# Kinetic Resolution of BINOLs and Biphenols by Atroposelective, Cu–H-Catalyzed Si–O Coupling with Hydrosilanes

**DOI:** 10.1021/acs.orglett.4c03557

**Published:** 2024-10-24

**Authors:** Lisa A. Böser, Martin Oestreich

**Affiliations:** Institut für Chemie, Technische Universität Berlin, Strasse des 17. Juni 115, 10623 Berlin, Germany

## Abstract

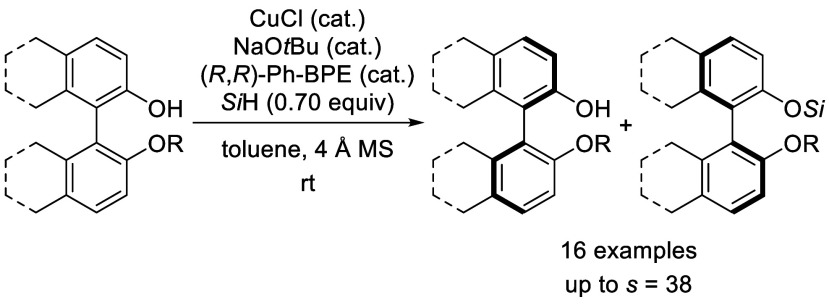

A nonenzymatic kinetic resolution of monoprotected BINOL and biphenol derivatives by atroposelective Si–O coupling with hydrosilanes is described. The reaction relies on a previously unprecedented Cu–H-catalyzed silylation of phenols. The catalyst system consisting of CuCl, (*R*,*R*)-Ph-BPE, and NaO*t*Bu enables the enantioselective coupling of the phenolic hydroxy group with a hydrosilane with moderate to good selectivity factors.

Over the past two decades, alcohol silylation strategies for their nonenzymatic kinetic resolution have evolved as a valuable tool in organic synthesis, thereby merging their protection with a stereocontrolled synthetic operation.^1^ Established methodologies often utilize chlorosilanes in combination with chiral nitrogen bases,^2^ while Song^3^ reported a kinetic resolution protocol using hexamethyldisilazane activated by chiral Brønsted acids. Furthermore, dehydrogenative coupling reactions can be carried out using transition-metal catalysts and hydrosilanes.^1b^ Our laboratory has had a long-standing interest in the kinetic resolution of alcohols by Cu–H-catalyzed, stereoselective Si–O couplings with monohydrosilanes, whereby we accomplished both reagent- and catalyst-controlled kinetic resolutions of donor-functionalized alcohols.^4^ In 2017, we introduced a catalyst-controlled kinetic resolution of simple secondary benzylic and allylic alcohols by enantioselective silylation.^5^ Since then, the procotol has been successfully extended to various other classes of alcohols,^1b^ and the inclusion of a racemization catalyst even enabled a dynamic kinetic resolution.^6^ However, our methodology has so far been confined to aliphatic alcohols with central chirality, which is why we have been aiming at expanding that resolution process to aromatic alcohols as part of axially chiral scaffolds. Until now, only one method for atroposelective silylation of phenols has been described in the literature, using List-type chiral Brønsted acid catalysts.^7^ Existing nonenzymatic methods^8^ for the kinetic resolution of BINOL and biphenol derivatives include esterification,^9^ alkylation^10^ and phosphorylation^11^ (Scheme 1, top), whereas a kinetic-resolution strategy by silylation remains unknown to the best of our knowledge. Herein, we present a kinetic resolution of biphenols by Cu–H-catalyzed, atroposelective Si–O coupling, employing a hydrosilane as the coupling partner (Scheme 1, bottom).

**Scheme 1:**
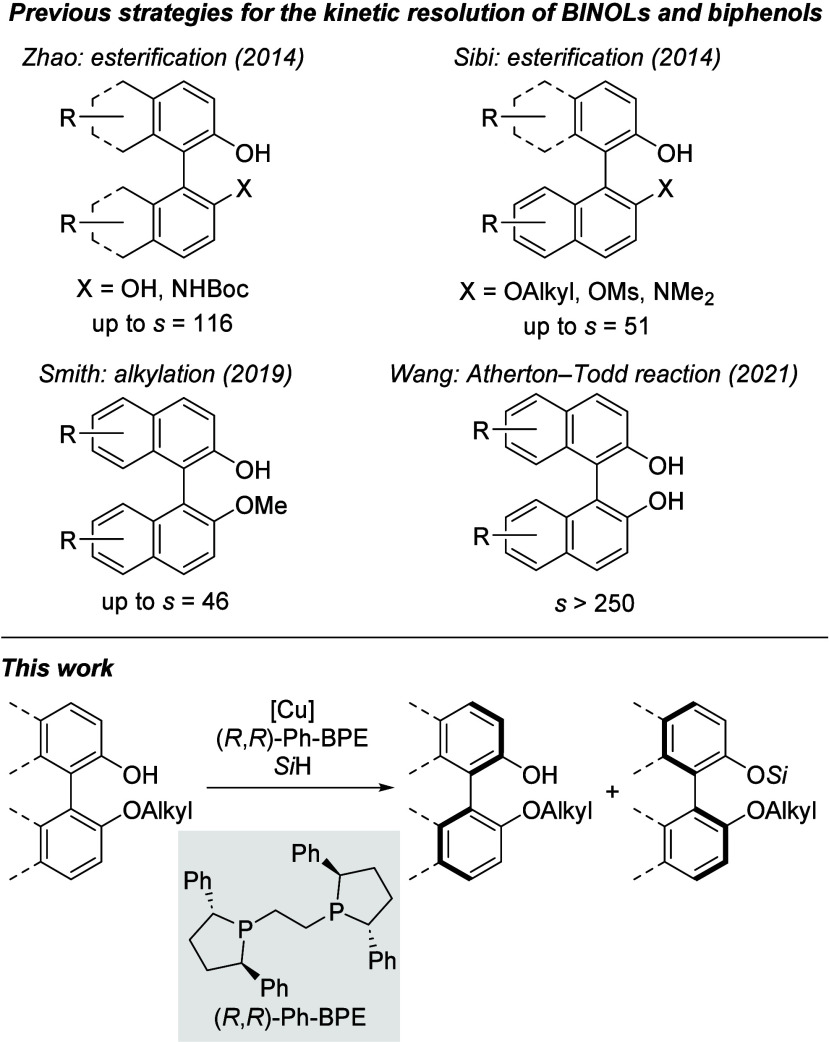
Previous Approaches of Kinetic Resolution of BINOL and Biphenol Derivatives and Planned Silylation

We commenced our study by subjecting racemic BINOL (**1a**) to our previously established catalytic system^5^ using MePh_2_SiH (**2a**) as the hydrosilane (Table 1, entry 1). Since **1a** showed poor solubility in toluene, these reaction conditions were not suitable. When conducted in dichloromethane, small quantitites of the silyl ether were isolated after 67 h but neither the silyl ether nor the unreacted alcohol **1a** showed any enantioenrichment. This prompted us to select monomethylated BINOL **1b** as the model substrate, where solubility in toluene was not an issue (entry 2). The dehydrogenative coupling with MePh_2_SiH (**2a**) was successful, yet with modest enantioselectivity (*s* = 7). The trialkyl-substituted hydrosilanes Et_3_SiH (**2b**) and Bn_3_SiH (**2c**) were too unreactive, and no reaction with **1b** was observed even at an elevated temperature of 50 °C (entries 3 and 4). When using sterically less demanding Me_2_PhSiH (**2d**), 12% of the desired silyl ether **3bd** formed after 22 h although the reaction mixture became increasingly complex over time (entry 5). Surprisingly, Ph_3_SiH (**2e**), which had been effective for silylating sterically demanding tertiary propargylic alcohols,^12^ did not lead to any silyl ether formation (entry 6). Given that MePh_2_SiH (**2a**) exhibited the most promising reactivity and selectivity, two electronically modified derivatives were tested. As anticipated, electron-deficient Me(3,5-(CF_3_)_2_C_6_H_3_)_2_SiH (**2f**) showed enhanced reactivity due to higher Lewis acidity at the silicon atom but the resulting silyl ether was unstable toward silica gel during purification (entry 7). Using electron-rich Me(3,5-Me_2_C_6_H_3_)_2_SiH (**2g**), the reaction was significantly slowed down relative to that of unsubstituted MePh_2_SiH (**2a**); the enantioselectivity was similar (*s* = 7) at a very low conversion of 9% after 74 h (entry 8). Since no synthetically useful selectivities were obtained with the model substrate **1b**, we decided to increase the steric demand by replacing the methyl by an isopropyl group (**1c**, entry 9). This modification led to a significantly better enantiodifferentiation in the silylation with MePh_2_SiH (**2a**, *s* = 12).

**Table 1:**
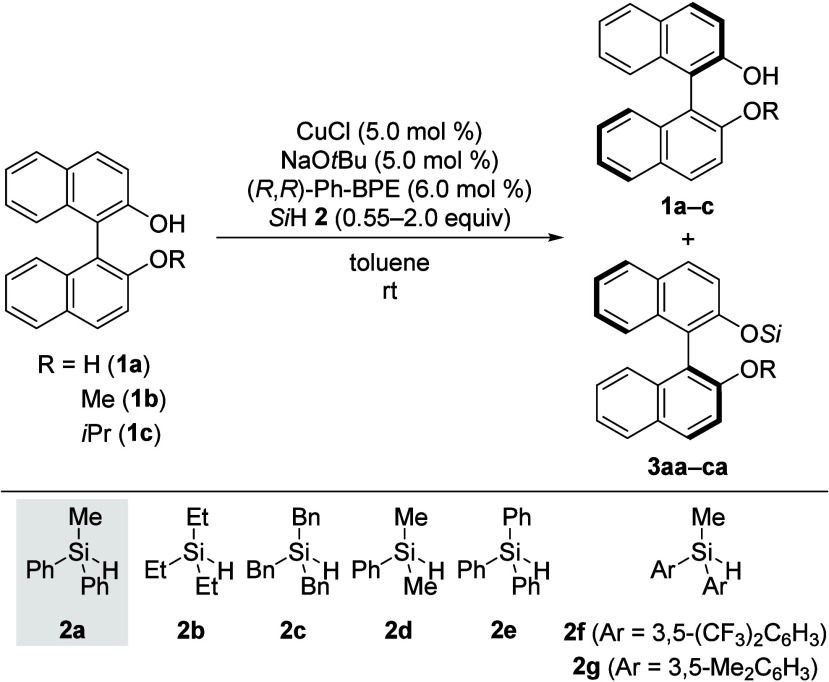
Substrate Identification and Hydrosilane Screening^a^

entry	R	*Si*H (equiv)	conv (%)^b^	time (h)	*s*^c^
1	**1a** (R = H)	**2a** (0.70)	n.r.		
2	**1b** (R = Me)	**2a** (0.55)	36	44	7
3^d^	**1b** (R = Me)	**2b** (0.55)	n.r.		
4^d^	**1b** (R = Me)	**2c** (0.55)	n.r.		
5	**1b** (R = Me)	**2d** (0.55)	12	22	
6	**1b** (R = Me)	**2e** (0.55)	n.r.		
7	**1b** (R = Me)	**2f** (0.55)	35	4	
8	**1b** (R = Me)	**2g** (0.55)	9	74	7
9	**1c** (R = *i*Pr)	**2a** (0.55)	38	75	12
10	**1c** (R = *i*Pr)	**2a** (0.70)	40	48	17
11	**1c** (R = *i*Pr)	**2a** (0.80)	44	29	16
12	**1c** (R = *i*Pr)	**2a** (1.0)	46	27	14
13	**1c** (R = *i*Pr)	**2a** (2.0)	64	24	11

a Unless otherwise noted, reactions were performed on a 0.2 mmol scale. n.r. = no reaction.

b Conversion was monitored by ^1^H NMR spectroscopy and calculated according to conversion = ee_unreacted alcohol_/(ee_silyl ether_ + ee_unreacted alcohol_) multiplied by 100.

c *s* = ln[(1 – *C*)(1 – ee)]/ln[(1 – *C*)(1 + ee)], where ee = ee_unreacted alcohol_/100 and *C* = conversion/100. The values are reported following published guidelines.^13^

d The reaction was performed at 50 °C.

We also serendipitously discovered that increasing the hydrosilane amount from 0.55 to 0.70 equiv further improved the selectivity (*s* = 17, entry 10). Using 0.80 equiv of **2a** maintained the same efficiency (entry 11) but further increasing to 1.0 or even 2.0 equiv led to a decrease in enantiodifferentiation (entries 12 and 13). The origin of this trend remains unclear. Of note and perhaps connected to this, a control experiment revealed that the formed silyl ether undergoes partial deprotection in the presence of the catalytic system, indicating that this might be a competing process to the forward reaction (see the Supporting Information for details).

Continuing with the optimized amount of the hydrosilane, we probed other (protecting) groups for the phenol function in the 2′-position (Scheme 2). Methyl and benzyl groups led to substantially diminished enantioselectivities compared to the isopropyl group (*s* = 5 for **1b** and *s* = 4 for **1d** vs *s* = 17 for **1c**). Since the increased steric demand from an isopropyl group seemed to be beneficial for the enantiodifferentiation, other bulky groups in this position were studied. However, larger steric congestion by introducing a benzhydryl moiety as in **1e** led to a complete loss of reactivity. A bulky silyl protecting group as in **1f** decreased the efficiency of the kinetic resolution drastically (*s* = 3). NOBIN congeners **1g** and **1h** with (partially) protected amine functions did not show any product formation, and a triflate group in **1i** was also not tolerated. Additionally, no reactivity was observed for **1j** when no heteroatom was present in the 2′-position.

**Scheme 2:**
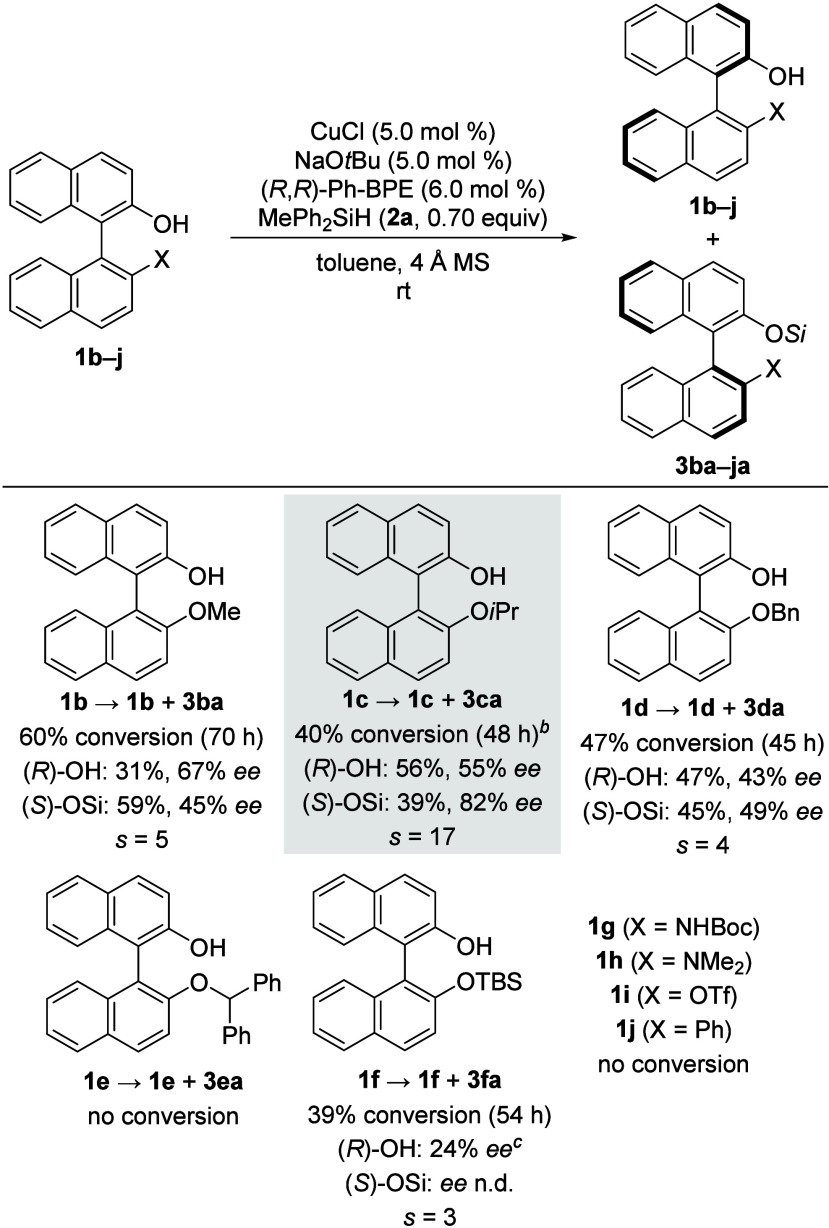
Substrate Scope I: Variation of Substituents in the 2′-Position of 2-Hydroxy-1,1′-binaphthalenes See caption of Table 1 for details. n.d. = not determined. No 4 Å MS was added to the catalyst mixture. The products were not isolated, and HPLC analysis was conducted of the crude reaction mixture.

Since the isopropyl group was providing the most valuable enantiodifferentation, we tested other BINOL and biphenol derivatives with an isopropoxy group in the 2′-position (Scheme 3). As anticipated, octahydro-BINOL-derived phenol **1k** exhibited good enantioselectivity (*s* = 15). Methylation of BINOL-derivative **1c** in the 4,4′-position was also tolerated (**1l**, *s* = 12) whereas phenyl or bromo substitution (**1m** and **1n**) resulted in no conversion. Methyl substitution in the 7,7′-position reduced enantiodifferentiation (**1o**, *s* = 6) and, as with **1n**, dibrominated **1p** did not undergo any silyl ether formation, suggesting that this is an electronic rather than a steric effect. In the 6,6′-position, selectivity decreased with the size of the attached substituent, yielding good selectivity factors in case of methyl (**1q**, *s* = 17) and isopropyl (**1r**, *s* = 13) while a moderate selectivity was obtained for phenyl (**1s**, *s* = 6). The observed alterations in enantioselectivity caused by substitution remote from the reaction site may result from changes in the dihedral angle of the binaphthyl system. For the example of **1c**, we also showed that the isopropyl group can easily be cleaved using boron trichloride, to deliver the free BINOL **1a** without racemization (see Supporting Information for details).

**Scheme 3:**
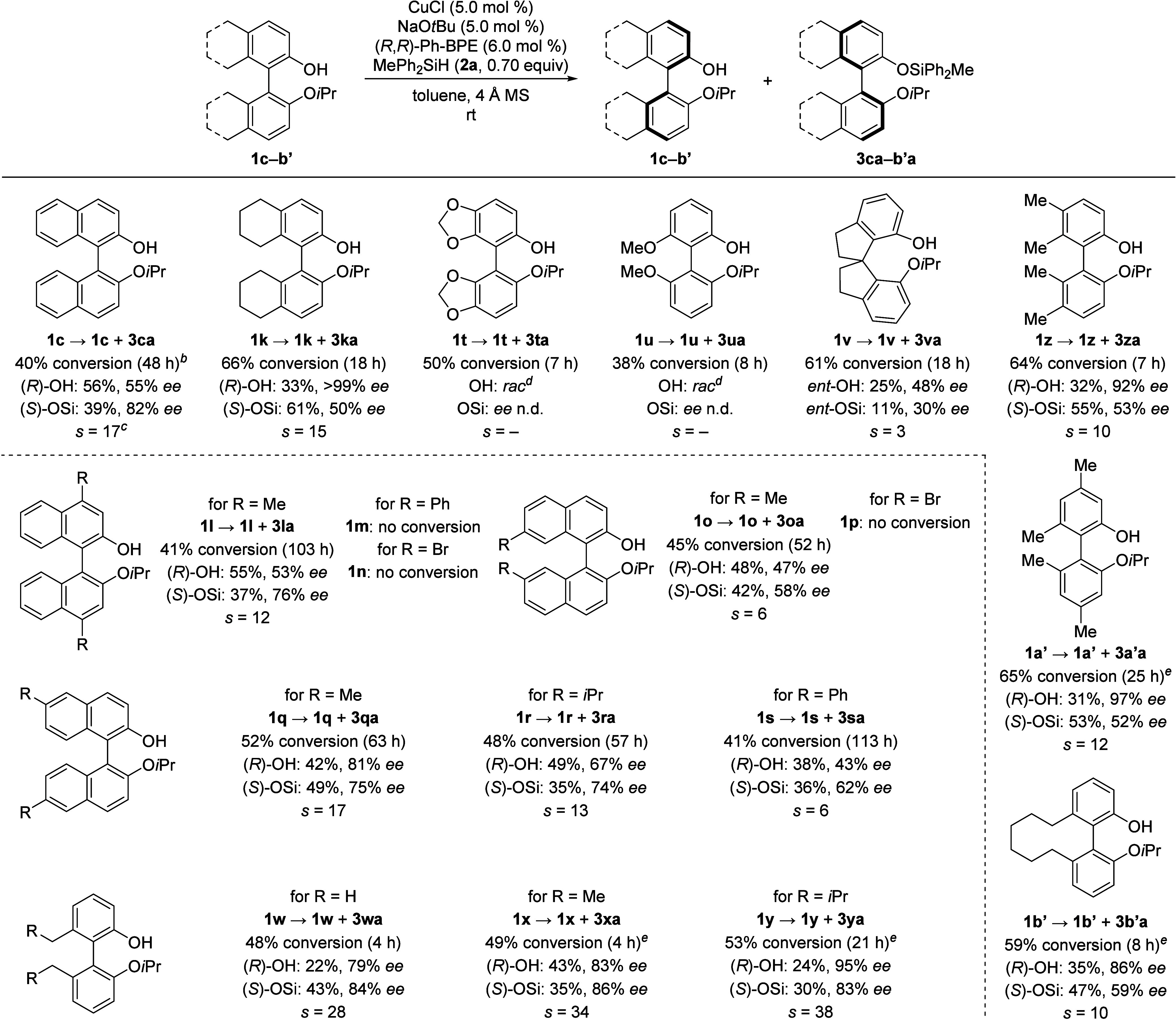
Substrate Scope II: Variations of the Biaryl Backbone See caption of Table 1 for details. No 4 Å MS was added to the catalyst mixture. A selectivity factor of *s* = 14 was obtained when the reaction was performed on a 1.0 mmol scale. The products were not isolated, and HPLC analysis was conducted of the crude reaction mixture. Purification was routinely done by flash-column chromatography, except for compounds **1x**, **1y**, **1a′**, and **1b′**, which were purified by preparative thin-layer chromatography.

Besides modifying the BINOL core, we also explored different backbones. Unfortunately, our catalyst system could neither achieve any enantiodifferentiation with compound **1t** featuring a benzo[*d*][1,3]dioxole scaffold nor with 6,6′-dimethoxy-biphenyl-based **1u**. Out of curiosity, we also tested a nonbiaryl backbone as seen in spirocyclic **1v**, however, the kinetic resolution occurred with poor efficiency (*s* = 3). Additionally, substrates with a biphenol scaffold were investigated. Among the substrates tested, phenol **1w** stood out in both reactivity and selectivity, and the atroposelective silylation was completed within 4 h with a selectivity factor of *s* = 28. Its analogue **1x** bearing an ethyl instead of a methyl group showed even higher selectivity (*s* = 34), and with an isobutyl group, the enantiodifferentiation slightly increased further (**1y**, *s* = 38). Tetramethylation in the 5,5′- and 6,6′-positions decreased the enantiodiscrimination, and the resolution process occurred with moderate selectivity of *s* = 10 for **1z**, indicating that steric hindrance in the 5,5′-positions is detrimental to the selectivity. Analogous to the BINOL derivatives, dimethylation in the 4,4′-positions as in **1a′** led to a decreased efficieny of the resolution (*s* = 12). Interestingly, the reaction of alkyl-bridged **1b′** showed diminished selectivity compared to nonbridged systems (*s* = 10), indicating again that changing the dihedral angle of the biaryl scaffold markedly impacts on the enantiodifferentiation.

In summary, we have disclosed the first example of a Cu–H-catalyzed Si–O coupling of phenols in general and atropisomeric phenols in particular. This work demonstrates that our previously established methodology for the kinetic resolution of centrally chiral aliphatic alcohols can also be applied to axially chiral aromatic alcohols. Moderate to good enantioselectivities were achieved for substrates with a BINOL core, and synthetically useful selectivity factors were obtained for 6,6′-dialkyl-substituted biphenols.

## Data Availability

The data underlying this study are available in the published article and its Supporting Information.

## Supporting Information Available

General procedures, experimental details, and characterization and spectral data for all new compounds (PDF)
