# Supplementary material for: Kinetic Resolution of BINOLs and Biphenols by Atroposelective, Cu–H-Catalyzed Si–O Coupling with Hydrosilanes
Source: Org Lett. 2024 Oct 24;26(44):9531–5. doi: 10.1021/acs.orglett.4c03557 (PMC11555675; doi:10.1021/acs.orglett.4c03557)
Supplement: Supplementary file 1 — ol4c03557_si_001.pdf [file ol4c03557_si_001.pdf]

Kinetic Resolution of BINOLs and Biphenols by Atroposelective, Cu–H-Catalyzed Si–O Coupling with Hydrosilanes

Lisa A. Böser and Martin Oestreich*

Institut für Chemie, Technische Universität Berlin,

Straße des 17. Juni 115, 10623 Berlin, Germany

martin.oestreich@tu-berlin.de

Supporting Information

Table of Contents

1 General Information S3

2 General Procedures....S6

2.1 General Procedure for the *O*-Alkylation of Phenols (GP1) S6

2.2 General Procedure for the Suzuki Cross Coupling (GP2) S6

2.3 General Procedure for the Methyl Ether Cleavage Using Boron Tribromide (GP3) S6

2.4 General Procedure for the Dehydrogenative Si–O Coupling of Phenols with Hydrosilanes (GP4) S7

2.5 General Procedure for the Deprotection of Enantioenriched Silyl Ethers (GP5) S7

3 Experimental Details for the Synthesis of the Racemic Substrates....S8

3.1 Modification of *rac*-BINOL (*rac*-**1a**) in the 2'-Position S8

3.2 Modification of the Backbone of *rac*-BINOL (*rac*-**1a**) S15

3.3 Synthesis of Spirocyclic *rac*-**1v**....S23

3.4 Synthesis of Biphenol-Substrates....S24

4 Experimental Details for the Kinetic Resolutions....S44

5 Optimization Studies and Control Experiments S70

5.1 Screening of Reaction Conditions S70

5.2 Investigation of the Silyl Ether Stability....S72

6 Scale-Up Experiment....S74

7 Removal of the Isopropyl-Group....S75

8 Determination of Absolute Configuration....S76

9 HPLC Traces S77

10 NMR Spectra S95

11 References S201

1 General Information

Unless otherwise stated, reactions were carried out under a nitrogen atmosphere using conventional Schlenk techniques or in a glovebox from *MBraun* under an argon atmosphere (H_2O < 0.5 ppm, O_2 < 0.5 ppm). Standard solvents and reagents were obtained from *ABCR*, *Acros Organics*, *Alfa Aesar*, *BLDpharm*, *Carbolution*, *Merck*, *Sigma-Aldrich* or *Tokyo Chemical Industry* (*TCI*). Glassware for reactions outside a glovebox was dried under a dynamic vacuum using a heat gun. Glassware for reactions inside the glovebox was oven-dried at 120 °C over night. Liquid reagents and solvents were transferred with disposable syringes, and all cannulas and syringes used in the glovebox were dried over night at 60 °C prior use. Reactions conducted at elevated temperatures were performed using a silicone oil bath for heating.

Solvents

The tetrahydrofurane, toluene and diethyl ether declared as anhydrous for reactions were refluxed under a nitrogen atmosphere over sodium with benzophenone as an indicator and distilled off when the indicator turned blue. Dichloromethane was refluxed over CaH_2 and then distilled under a nitrogen atmosphere. Solvents designated as degassed underwent three successive freeze-pump-thaw cycles, unless otherwise stated. The solvents used for reactions under non-anhydrous conditions were obtained in synthesis grade or technical grade, with technical grade solvents being distilled under reduced pressure before use. Solvents for glovebox reactions were always degassed and stored over thermally activated 4 Å molecular sieves. Solvents used for flash chromatography and extraction (cyclohexane, *n*-pentane, *tert*-butyl methyl ether, ethyl acetate and dichloromethane) were obtained in technical grade and distilled under reduced pressure before use. Deionized water was utilized. For analytical high-performance liquid chromatography (HPLC), solvents of corresponding purity from *Fisher Scientific* were employed.

Chromatography

Qualitative thin-layer chromatography (TLC) was performed on silica-coated aluminum plates with fluorescence indicator UV_{254} from *Macherey-Nagel*. Product spots were visualized by UV light (λ = 254 nm) and/or by staining with a ceric ammonium molybdate solution. Silica gel from *Grace* with a particle size of 30–63 µm was used as the stationary phase for flash column chromatography. Preparative TLC plates were prepared by applying a mixture of *Macherey-Nagel* silica gel with a UV_{254} fluorescence indicator and 2 wt.-% sodium carboxymethyl cellulose onto 20 x 20 cm glass plates. Automated column chromatography was carried out using an

Isolera One™ system from *Biotage* equipped with KP Sil columns. Analytical separations using high-performance liquid chromatography (HPLC) were carried out on a 1200 or 1200 Infinity HPLC system from *Agilent Technologies*. Solvent mixtures consisting of *n*-heptane/isopropanol were used, and the separations were performed on the indicated chiral stationary phase from *Daicel*.

Gas-Liquid Chromatography (GLC)

Gas-liquid chromatography (GLC) was conducted using an Agilent Technologies GC 8860 gas chromatograph, fitted with an *Agilent Technologies* J&W HP-5 capillary column (30 m x 0.32 mm, 0.25 µm film thickness). The analysis was performed under the following conditions: nitrogen was used as the carrier gas, the injection temperature was set to 250 °C, the detector temperature to 275 °C, and the flow rate was maintained at a constant pressure of 11 psi. The temperature program initiated at 40 °C, with a heating rate of 10 °C/min, and reached a final temperature of 280 °C, which was held for 10 minutes.

Nuclear Magnetic Resonance (NMR) Spectroscopy

^{1}H, ^{13}C and ^{29}Si NMR spectra were recorded on a *Bruker* AV 400 or AV 500 using $CDCl_3$, C_6D_6, CD_2Cl_2 or acetone-d_6 as the deuterated solvent. The chemical shifts are reported in parts per million (ppm) and are referenced to the residual protic solvent peaks in case of ^{1}H NMR and to the signals of the deuterated solvent carbon signals for ^{13}C NMR ($CHCl_3$: δ/ppm = 7.26 for ^{1}H NMR and $CDCl_3$ = 77.16 for ^{13}C NMR, C_6D_5H: δ/ppm = 7.16 for ^{1}H NMR and C_6D_6 = 128.06 for ^{13}C NMR, $CDHCl_2$: δ/ppm = 5.32 for ^{1}H NMR and CD_2Cl_2 = 53.84 for ^{13}C NMR, acetone-d_5: δ/ppm = 2.05 for ^{1}H NMR and acetone-d_6 = 29.84, 206.26 for ^{13}C NMR). Other measured nuclei (^{29}Si) were internally referenced relative to the resonance line of tetramethylsilane in the ^{1}H NMR spectrum using the unified chemical shift scale recommended by IUPAC. Data are reported in the following way: chemical shift, multiplicity (s = singlet, d = doublet, t = triplet, q = quartet, sept = septet, non = nonet, m = multiplet, m_c = centrosymmetric multiplet and combinations thereof), coupling constant (Hz), and integration. The specified multiplicities are phenomenological and do not necessarily correspond to the theoretically expected multiplicities. Structural assignments were made with additional information from gCOSY, gHSQC, and gHMBC experiments.

Mass Spectrometry

Mass spectrometric analyses were carried out by the *Department of Mass Spectrometry* at the *Institute of Chemistry* at the *Technical University of Berlin*. The spectra were recorded by chemical ionization at atmospheric pressure (APCI) on the LTQ Orbitrap XL device from *Thermo Scientific*. A detailed description of the fragmentation was omitted and only the molecular ion peak or a characteristic fragment was taken into account.

Infrared (IR) Spectroscopy

Infrared spectra were recorded using a Cary 630 FT-IR Fourier transform infrared spectrometer from *Agilent Technologies*. The characteristic absorption bands are reported in wavenumbers (cm^{-1}). The band intensities are described using the following abbreviations: w (weak), m (medium), s (strong), vs (very strong). Broad bands are additionally labeled with "br."

Optical Rotation

The specific rotation of the measured optically active substances was determined using an H532 polarimeter from *Schmidt+Haensch*. The compound was dissolved in the specified solvent and transferred to a 1 dm cuvette for measurement. The specific rotation values $[\alpha]_{\lambda}^{\vartheta}$ were calculated using the following equation:

$$[\alpha]_{\lambda}^{\vartheta} = \frac{[\alpha]\times 100}{c\times d}$$

In this equation, $[\alpha]_{\lambda}^{\vartheta}$ denotes the specific rotation value, with [α] denoting the measured rotation value, λ representing the wavelength of the light in nm and ϑ indicating the measurement temperature in °C. When the sodium D-line (λ = 589 nm) is used as the light source, it is indicated by the index "$_{D}$". The rotation value is calculated using the concentration c (in g/100 mL) and the cuvette length d (in dm).

Melting Points

Melting points (M.p.) were recorded with the SMP20 device from *Stuart* or on a Leica Galen III hot stage microscope from *Wagner & Munz* and are not corrected.

2 General Procedures

2.1 General Procedure for the *O*-Alkylation of Phenols (GP1)

Following a modified literature procedure,[1] the phenol (1.00 equiv) was dissolved in acetone (0.1 M) and potassium carbonate (1.25–1.35 equiv) was added (open to air). The suspension was stirred for 10 min at room temperature before adding the respective alkyl iodide (1.0–1.3 equiv). The reaction mixture was stirred at the specified temperature for the given duration, and workup was carried out as described.

2.2 General Procedure for the Suzuki Cross Coupling (GP2)

Following a modified literature procedure,[1] the indicated alcohol (1.00 equiv) and the indicated boronic acid (4.00 equiv) were added to a Schlenk tube together with caesium carbonate (4.00 equiv) and [1,1′-Bis(diphenylphosphino)ferrocene]dichloropalladium(II) dichloromethane complex ($Pd(dppf)Cl_2 \cdot CH_2Cl_2$, 10 mol %). The Schlenk tube was evacuated and backfilled with nitrogen for three times. Subsequently, 1,4-dioxane (which had been purged with nitrogen for 10 minutes) was added to the solid mixture, resulting in a 0.1 M solution with respect to the alcohol. The reaction was refluxed for the indicated time, cooled to room temperature, and filtered over silica. After evaporation of the solvents, flash column chromatography on silica gel afforded the pure cross coupling products.

2.3 General Procedure for the Methyl Ether Cleavage Using Boron Tribromide (GP3)

Following a modified literature procedure,[2] the indicated methyl ether (1.00 equiv) was dissolved in dry dichloromethane (0.5 M) in a dried Schlenk tube. The solution was cooled to 0 °C, followed by dropwise addition of a solution of boron tribromide in dichloromethane (1.0 M, 2.0 equiv). The reaction mixture was stirred for the indicated time while slowly being warmed to room temperature. Then, the solution was cooled back to 0 °C, and quenched by careful addition of water. The phases were separated, and the aqueous phase was extracted with dichloromethane. The combined organic layers were dried over Na_2SO_4, filtered and the solvent was removed under reduced pressure. The analytically pure alcohols were obtained by filtration over silica or column chromatography on silica gel.

2.4 General Procedure for the Dehydrogenative Si–O Coupling of Phenols with Hydrosilanes (GP4)

In an argon-filled glovebox, an oven-dried 1.5 mL vial was charged with sodium *tert*-butanolate (1.0 mg, 10 μmol, 5.0 mol %), copper(I) chloride (1.0 mg, 10 μmol, 5.0 mol %) and (*R*,*R*)-Ph-BPE (6.1 mg, 12 μmol, 6.0 mol %), a magnetic stir bar and grounded 4 Å molecular sieves (if indicated). Toluene (0.1 mL) was added, and the resulting mixture was stirred for 15 min at room temperature. In a second oven-dried 1.5 mL vial, the alcohol (0.200 mmol, 1.00 equiv) and the indicated hydrosilane (0.55–2.0 equiv for the optimization, 0.70 equiv for the substrate scope) were dissolved in toluene (0.5 mL). The solution containing the alcohol and the hydrosilane was added to the catalyst mixture and the reaction was stirred for the indicated time at the stated temperature, monitoring the reaction progress with ^{1}H NMR spectroscopy. The reaction was quenched by filtration over silica, and flash column chromatography on silica gel or preparative TLC afforded the analytically pure silyl ether and alcohol.

2.5 General Procedure for the Deprotection of Enantioenriched Silyl Ethers (GP5)

A 8 mL vial was charged with a stirring bar and 1.0–5.0 mg of the indicated silyl ether (open to air). A solution of tetra-*n*-butylammonium fluoride (1.0 mL, 1.0 M in THF) was added, and the solution was stirred for 10 min at room temperature. The reaction was quenched by addition of water (1 mL), diluted with *tert*-butyl methyl ether (1 mL), the phases were separated, and the aqueous phase was extracted with *tert*-butyl methyl ether (3 x 1 mL). The combined organic layers were dried over Na_2SO_4, filtered and the solvents were removed under reduced pressure. Flash column chromatography on silica gel using cyclohexane/ethyl acetate or cyclohexane/*tert*-butyl methyl ether solvent mixtures afforded the corresponding alcohol.

3 Experimental Details for the Synthesis of the Racemic Substrates

3.1 Modification of *rac*-BINOL (*rac*-1a) in the 2'-Position

rac-2'-Methoxy-[1,1'-binaphthalen]-2-ol (*rac*-1b)

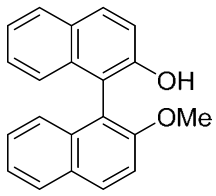

rac-**1b**
$C_{21}H_{16}O_2$
M = 300.36 g/mol

Prepared according to **GP1** from racemic BINOL (*rac*-**1a**, 580 mg, 2.03 mmol, 1.00 equiv), potassium carbonate (351 mg, 2.54 mmol, 1.25 equiv) and methyl iodide (0.13 mL, 2.0 mmol, 1.0 equiv) in acetone (25 mL). The reaction mixture was stirred for 48 h at room temperature, filtered over Celite® and the solvent was removed under reduced pressure. Flash column chromatography on silica gel using cyclohexane/*tert*-butyl methyl ether (10:1 → 7:1 → 4:1) afforded the title compound *rac*-**1b** (1.19 g, 1.39 mmol, 89%) as a pale yellow solid.

The analytical data are consistent with those reported in section 4 for the enantioenriched alcohol.

rac-2'-Isopropoxy-[1,1'-binaphthalen]-2-ol (*rac*-1c)

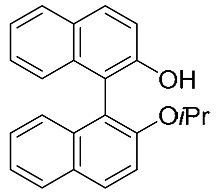

rac-**1c**
$C_{23}H_{20}O_2$
M = 328.41 g/mol

Prepared according to **GP1** from racemic BINOL (*rac*-**1a**, 573 mg, 2.00 mmol, 1.00 equiv), potassium carbonate (346 mg, 2.50 mmol, 1.25 equiv) and 2-iodopropane (0.20 mL, 2.0 mmol, 1.0 equiv) in acetone (20 mL). The reaction mixture was refluxed for 21 h, cooled to room temperature, filtered over Celite® and the solvent was removed under reduced pressure. Flash

column chromatography on silica gel using cyclohexane/dichloromethane (1:1 → 2:1) afforded the title compound *rac*-**1c** (439 mg, 1.34 mmol, 67%) as a yellow solid.

The analytical data are consistent with those reported in section 4 for the enantioenriched alcohol.

***rac*-2'-(Benzyloxy)-[1,1'-binaphthalen]-2-ol (*rac*-1d)**

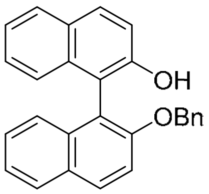

rac-**1d**
$C_{27}H_{20}O_2$
M = 376.46 g/mol

Prepared according to **GP1** from racemic BINOL (*rac*-**1a**, 1.43 g, 5.00 mmol, 1.00 equiv), potassium carbonate (864 mg, 6.25 mmol, 1.25 equiv) and benzyl bromide (0.59 mL, 5.0 mmol, 1.0 equiv) in acetone (50 mL). The reaction mixture was stirred for 20 h at room temperature, filtered over Celite® and the solvent was removed under reduced pressure. Flash column chromatography on silica gel using cyclohexane/dichloromethane (1:1 → 2:1) afforded the title compound *rac*-**1d** (1.19 g, 3.16 mmol, 63%) as a pale brown solid.

The analytical data are consistent with those reported in section 4 for the enantioenriched alcohol.

***rac*-2'-(Benzhydryloxy)-[1,1'-binaphthalen]-2-ol (*rac*-1e)**

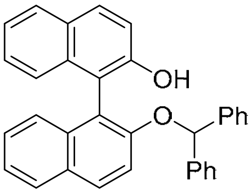

rac-**1e**
$C_{33}H_{24}O_2$
M = 452.55 g/mol

In a dried Schlenk tube, racemic BINOL (*rac*-**1a**, 859 mg, 3.00 mmol, 1.00 equiv) was dissolved in dry THF (6 mL) and the solution was cooled down to 0 °C. Sodium hydride (120 mg, 3.00 mmol, 1.00 equiv, 60 wt.% in paraffine oil) was added portionwise over 2 min and the resulting suspension was stirred at 0 °C for 45 min. Then, the mixture was warmed to room

temperature, benzhydryl bromide (741 mg, 3.00 mmol, 1.00 equiv) was added in one portion and the reaction mixture was heated to reflux. After refluxing for 24 h and cooling down to room temperature, water (20 mL) and ethyl acetate (10 mL) were added. The phases were separated, and the aqueous phase was extracted with ethyl acetate (3 x 20 mL). The combined organic layers were dried over Na_2SO_4, filtered and the solvent was removed under reduced pressure. Flash column chromatography on silica gel using cyclohexane/dichloromethane (3:1 → 2:1 → 1:1) afforded the title compound *rac*-**1e** (355 mg, 0.784 mmol, 26%) as a yellow solid.

^{1}H NMR (500 MHz, $CDCl_3$): δ/ppm = 7.94 (d, *J* = 8.9 Hz, 1H), 7.89 (d, *J* = 8.9 Hz, 2H), 7.84 (d, *J* = 8.2 Hz, 1H), 7.42 (d, *J* = 9.1 Hz, 1H), 7.31–7.36 (m, 2H), 7.27–7.29 (m, 2H), 7.14–7.25 (m, 5H), 7.12 (d, *J* = 6.9 Hz, 2H), 7.02–7.10 (m, 4H), 6.93 (d, *J* = 7.3 Hz, 2H), 6.25 (s, 1H), 4.94 (s, 1H). **^{13}C{^{1}H} NMR** (126 MHz, $CDCl_3$): δ/ppm = 154.1, 151.6, 141.4, 141.2, 134.2, 134.0, 130.7, 130.0, 129.8, 129.3, 128.6 (2C), 128.3 (2C), 128.2, 128.2, 127.7, 127.5, 127.4, 126.6 (2C), 126.5 (2C), 126.5, 125.4, 125.3, 124.6, 123.4, 117.8, 117.6, 117.1, 115.4, 82.4.

The NMR spectroscopic data are consistent with those reported in the literature.[3]

rac 2'-[(*tert*-Butyldimethylsilyl)oxy]-[1,1'-binaphthalen]-2-ol (*rac*-1f)

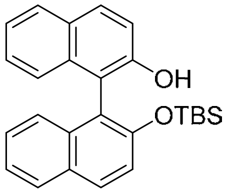

rac-**1f**
$C_{26}H_{28}O_2Si$
M = 400.59 g/mol

In a dried Schlenk tube, racemic BINOL (*rac*-**1a**, 859 mg, 3.00 mmol, 1.00 equiv) was dissolved in dry dichloromethane (15 mL). Imidazole (204 mg, 3.00 mg, 1.00 equiv) was added, followed by dropwise addition of a solution of *tert*-butylchlorodimethylsilane (3.0 mL, 3.0 mmol, 1.0 equiv, 1.0 M in dichloromethane). The solution was stirred for 17 h and quenched by addition of water (15 mL). The phases were separated, the aqueous phase was extracted with dichloromethane (3 x 15 mL) and the combined organic phases were dried over Na_2SO_4, filtered, and concentrated under reduced pressure. Flash column chromatography on silica gel using cyclohexane/*tert*-butyl methyl ether (15:1) afforded the title compound *rac*-**1f** (765 mg, 1.91 mmol, 64%) as a colorless resin.

^{1}**H NMR** (500 MHz, $CDCl_3$): δ/ppm = 7.93 (d, *J* = 9.0 Hz, 1H), 7.88 (t, *J* = 9.0 Hz, 2H), 7.83 (d, *J* = 8.1 Hz, 1H), 7.38 (m_c, 1H), 7.32 (d, *J* = 8.9 Hz, 1H), 7.31–7.26 (m, 3H), 7.25–7.20 (m, 2H), 7.08 (d, *J* = 8.3 Hz, 1H), 5.03 (s, br, 1H), 0.52 (s, 9H), 0.00 (s, 3H), –0.21 (s, 3H). ^{13}**C{^{1}H} NMR** (126 MHz, $CDCl_3$): δ/ppm = 152.5, 151.6, 134.3, 134.0, 130.7, 129.9, 129.8, 129.3, 128.3, 128.1, 127.2, 126.3, 125.4, 125.4, 124.4, 123.2, 121.4, 118.5, 117.7, 115.7, 25.1 (3C), 17.7, –4.3, –4.6. ^{1}**H/^{29}Si HMQC NMR** (500/99 MHz, $CDCl_3$, optimized for *J* = 7 Hz): δ/ppm = (0.52, 0.00, –0.21)/21.9.

The NMR spectroscopic data are consistent with those reported in the literature.[4]

***rac*-*tert*-Butyl-(2'-hydroxy-[1,1'-binaphthalen]-2-yl)carbamate (*rac*-1g)**

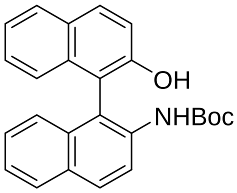

rac-**1g**
$C_{25}H_{23}NO_3$
M = 385.46 g/mol

Following a modified literature procedure,[5] racemic 2-amino-2'-hydroxy-1,1'-binaphthyl (300 mg, 1.05 mmol, 1.00 equiv) was suspended in benzene (6 mL) in a round bottom flask open to air. Di-*tert*-butyl dicarbonate (229 mg, 1.50 mmol, 1.00 equiv) was added and the resulting mixture was heated to reflux. After stirring for 48 h, the reaction mixture was cooled to room temperature and the solvent was removed under reduced pressure. Flash column chromatography on silica gel using cyclohexane/ethyl acetate (10:1) afforded the title compound *rac*-**1g** (119 mg, 0.309 mmol, 29%) as a pale yellow solid.

^{1}**H NMR** (500 MHz, $CDCl_3$): δ/ppm = 8.50 (d, *J* = 9.2 Hz, 1H), 8.03 (d, *J* = 9.1 Hz, 1H), 7.98 (d, *J* = 8.9 Hz, 1H), 7.91 (dd, *J* = 7.7, 5.0 Hz, 2H), 7.45–7.35 (m, 3H), 7.29–7.26 (m, 2H), 7.07 (d, *J* = 8.5 Hz, 1H), 7.02 (d, *J* = 8.5 Hz, 1H), 6.24 (s, 1H), 4.99 (s, br, 1H), 1.39 (s, 9H). ^{13}**C{^{1}H} NMR** (126 MHz, $CDCl_3$): δ/ppm = 153.2, 152.1, 136.6, 133.4, 133.1, 131.2, 130.8, 130.4, 129.5, 128.5, 128.4, 127.4, 127.4, 125.1 (2C), 124.5, 124.0, 120.2, 118.0, 116.4, 113.2, 81.1, 28.3 (3C).

The NMR spectroscopic data are consistent with those reported in the literature.[5]

rac-2'-(Dimethylamino)-[1,1'-binaphthalen]-2-ol (rac-1h)

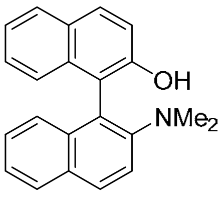

rac-**1h**
$C_{22}H_{19}NO$
M = 313.40 g/mol

Following a modified literature procedure,[6] a mixture of sulphuric acid (2.7 mL, 20 wt.% in water) and formaldehyde (1.5 mL, 20 mmol, 13 equiv, 37 wt.% in water) was placed in a round bottom flask open to air and diluted with water (15 mL). To this, a solution of racemic 2-amino-2'-hydroxy-1,1'-binaphthyl (428 mg, 1.50 mmol, 1.00 equiv) in THF (20 mL) and solid sodium borohydride (397 mg, 10.5 mmol, 7.00 equiv) were added alternately over 20 min. After complete addition, the mixture was stirred for another 10 min, and an aqueous solution of 2 M NaOH (20 mL) was added. The phases were separated, and the aqueous phase was extracted with *tert*-butyl methyl ether (3 x 30 mL). The combined organic phases were dried over Na_2SO_4, filtered and the solvent was removed under reduced pressure. Flash column chromatography on silica gel using cyclohexane/ethyl acetate (10:1 + 2% Et_3N) followed by subsequent recrystallization from *n*-heptane afforded the title compound *rac*-**1h** (217 mg, 0.692 mmol, 46%) as a pale yellow solid.

^{1}H NMR (500 MHz, $CDCl_3$): δ/ppm = 7.98 (d, *J* = 9.0 Hz, 1H), 7.91–7.83 (m, 3H), 7.59 (d, *J* = 9.0 Hz, 1H), 7.43 (d, *J* = 8.8 Hz, 1H), 7.36 (t, *J* = 7.5 Hz, 1H), 7.31 (t, *J* = 7.3 Hz, 1H), 7.23–7.13 (m, 2H), 7.09–7.00 (m, 2H), 2.71 (s, 6H). The resonance for the OH group was not observed. **^{13}C{^{1}H} NMR** (126 MHz, $CDCl_3$): δ/ppm = 151.8, 149.5, 134.2, 134.1, 130.2, 130.0, 129.8, 129.4, 128.3, 128.0, 126.7, 126.4, 126.0, 125.8, 124.3, 123.3, 122.2, 119.5, 118.5, 118.4, 43.7 (2C).

The NMR spectroscopic data are consistent with those reported in the literature.[6]

rac-2'-Hydroxy-[1,1'-binaphthalen]-2-yl trifluoromethanesulfonate (*rac*-1i)

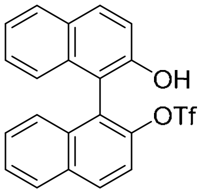

rac-**1i**
$C_{21}H_{13}F_3O_4S$
M = 418.39 g/mol

Following a modified literature procedure,[7] racemic BINOL (*rac*-**1a**, 2.86 g, 10.0 mmol, 1.00 equiv) was suspended in dry dichloromethane (50 mL) in a dried Schlenk tube. *N*,*N*-Ethyldiisopropylamine (1.9 mL, 11 mmol, 1.1 equiv) was added and the mixture was stirred for 10 min, until *rac*-**1a** was dissolved completely. Afterwards, the solution was cooled to 0 °C and triflic anhydride (1.9 mL, 11 mmol, 1.1 equiv) was added dropwise over 5 min. After slowly warming to room temperature for 18 h, the reaction was quenched by addition of 2 M HCl (20 mL). The phases were separated, and the aqueous phase was extracted with dichloromethane (3 x 40 mL). The combined organic phases were washed with saturated aqueous $NaHCO_3$ solution (100 mL), dried over Na_2SO_4, filtered and the solvent was removed under reduced pressure. Flash column chromatography on silica gel using cyclohexane/ethyl acetate (20:1 → 10:1) afforded the title compound *rac*-**1i** (1.80 g, 4.30 mmol, 43%) as a yellow resin.

^{1}H NMR (500 MHz, $CDCl_3$): δ/ppm = 8.13 (d, *J* = 9.1 Hz, 1H), 8.03 (d, *J* = 8.3 Hz, 1H), 7.98 (d, *J* = 9.0 Hz, 1H), 7.89 (d, *J* = 8.1 Hz, 1H), 7.62–7.59 (m, 2H), 7.47–7.43 (m, 2H), 7.39–7.33 (m, 2H), 7.29 (t, *J* = 7.2 Hz, 1H), 7.02 (d, *J* = 8.4 Hz, 1H), 4.88 (s, br, 1H). **^{13}C{^{1}H} NMR** (126 MHz, $CDCl_3$): δ/ppm = 151.9, 146.3, 133.4, 133.4, 133.1, 131.8, 131.6, 129.3, 128.6, 128.5, 128.4, 127.7, 127.2, 126.6, 125.3, 124.3, 124.0, 120.0, 118.4 (q, *J* = 321 Hz), 118.0, 112.2. **^{19}F NMR** (471 MHz, $CDCl_3$): δ/ppm = –74.4 (s).

The NMR spectroscopic data are consistent with those reported in the literature.[8]

rac-2'-Phenyl-[1,1'-binaphthalen]-2-ol (*rac*-1j)

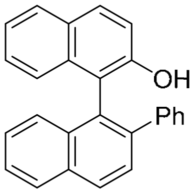

rac-**1j**
$C_{26}H_{18}O$
M = 346.43 g/mol

A dried Schlenk tube was charged with $NiCl_2$(dppe) (106 mg, 0.200 mmol, 4.00 mol%) and a solution of the *rac*-**1i** (719 mg, 1.72 mmol, 1.00 equiv) in dry THF (3.4 mL) was added. The mixture was cooled to 0 °C and a solution of phenylmagnesium bromide (5.4 mL, 8.6 mmol, 1.6 M in cyclopentyl methyl ether) was added dropwise over 5 min. The reaction mixture was warmed to room temperature and stirred for 14 h, followed by dropwise addition of saturated aqueous NH_4Cl solution (10 mL). The phases were separated, and the aqueous phase was extracted with *tert*-butyl methyl ether (3 x 15 mL). The combined organic layers were dried over Na_2SO_4, filtered and the solvent was removed under reduced pressure. Automatic flash column chromatography on silica gel using cyclohexane/ethyl acetate (100:0 → 13:1) afforded the title compound *rac*-**1j** (350 mg, 1.01 mmol, 59%) as a colorless solid.

^{1}H NMR (500 MHz, $CDCl_3$): δ/ppm = 8.10 (d, *J* = 8.5 Hz, 1H), 7.99 (d, *J* = 8.1 Hz, 1H), 7.78 (d, *J* = 8.7 Hz, 2H), 7.72 (d, *J* = 8.5 Hz, 1H), 7.36–7.27 (m, 3H), 7.16–7.12 (m, 3H), 7.12–7.02 (m, 4H), 7.22 (m_c, 1H), 7.52 (t, *J* = 7.5 Hz, 1H), 4.82 (s, br, 1H). **^{13}C{^{1}H} NMR** (126 MHz, $CDCl_3$): δ/ppm = 151.2, 141.7, 141.0, 134.3, 133.4, 133.3, 130.0, 129.5, 128.9, 128.7 (2C), 128.7 (2C), 128.3, 128.2, 127.8 (2C), 127.3, 127.1, 126.7, 126.5, 126.5, 125.2, 123.3, 117.9, 117.3.

The NMR spectroscopic data are consistent with those reported in the literature.[8]

3.2 Modification of the Backbone of *rac*-BINOL (*rac*-1a)

rac-2'-Isopropoxy-5,5',6,6',7,7',8,8'-octahydro-[1,1'-binaphthalen]-2-ol (*rac*-1k)

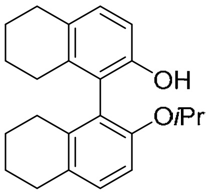

rac-**1k**
$C_{23}H_{28}O_2$
M = 336.48 g/mol

Prepared according to **GP1** from racemic 5,5',6,6',7,7',8,8'-octahydro-[1,1'-binaphthalene]-2,2'-diol (736 mg, 2.50 mmol, 1.00 equiv), potassium carbonate (432 mg, 2.50 mmol, 1.25 equiv) and 2-iodopropane (0.25 mL, 2.5 mmol, 1.0 equiv) in acetone (25 mL). The reaction mixture was refluxed for 20 h, cooled to room temperature and loaded on Celite®. Flash column chromatography on silica gel using cyclohexane/*tert*-butyl methyl ether (10:1) afforded the title compound *rac*-**1k** (140 mg, 0.416 mmol, 17%) as a brown resin.

The analytical data are consistent with those reported in section 4 for the enantioenriched alcohol.

Scheme S1. Synthesis of core-substituted BINOL derivatives.

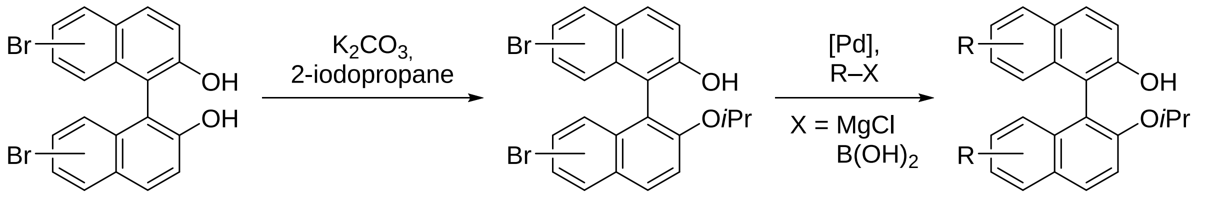

The dibrominated [1,1'-binaphthalene]-2,2'-diol derivatives were synthesized from the corresponding brominated naphthalen-2-ols following a literature procedure.[1]

***rac*-4,4'-Dibromo-2'-isopropoxy-[1,1'-binaphthalen]-2-ol (*rac*-1n)**

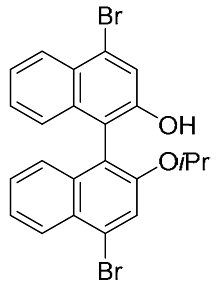

rac-**1n**
$C_{23}H_{18}Br_2O_2$
M = 486.20 g/mol

Prepared according to **GP1** from racemic 4,4'-dibromo-[1,1'-binaphthalene]-2,2'-diol (3.30 g, 7.43 mmol, 1.00 equiv), potassium carbonate (1.38 g, 10.0 mmol, 1.35 equiv) and 2-iodopropane (0.82 mL, 8.2 mmol, 1.1 equiv) in acetone (74 mL). The reaction mixture was refluxed for 38 h, cooled to room temperature, filtered over Celite® and the solvent was removed under reduced pressure. Flash column chromatography on silica gel using cyclohexane/dichloromethane (20:1 → 15:1 → 10:1 → 5:1 → 1:1) afforded the title compound *rac*-**1n** (1.99 g, 4.09 mmol, 55%) as a yellow solid.

M.p. = 49–52 °C (dichloromethane/*n*-pentane). **R_f** = 0.41 (cyclohexane/dichloromethane = 1:1). **^{1}H NMR** (500 MHz, $CDCl_3$): δ/ppm = 8.26 (m_c, 2H), 7.78 (s, 1H), 7.70 (s, 1H), 7.48 (t, *J* = 7.5 Hz, 1H), 7.41 (t, *J* = 7.3 Hz, 1H), 7.30 (t, *J* = 7.4 Hz, 1H), 7.26–7.22 (m, 1H), 7.15 (d, *J* = 8.5 Hz, 1H), 7.04 (d, *J* = 8.5 Hz, 1H), 4.95 (s, br, 1H), 4.51 (sept, *J* = 6.0 Hz, 1H), 1.16 (d, *J* = 6.0 Hz, 3H), 1.02 (d, *J* = 6.0 Hz, 3H). **^{13}C{^{1}H} NMR** (126 MHz, $CDCl_3$): δ/ppm = 154.3, 151.1, 134.8, 134.5, 128.5, 128.2, 127.8, 127.7, 127.4, 127.3, 125.9, 125.6, 125.5 (2C), 124.7, 123.9, 121.9, 121.6, 117.1, 115.3, 73.0, 22.3, 22.2. **HRMS** (APCI) calculated for $C_{23}H_{19}Br_2O_2^+$ [$(M+H)^+$]: 484.9746; found: 484.9747. **IR** (ATR): ṽ/cm^{-1} = 3517, 1066, 2975, 2929, 1577, 1496, 1453, 1369, 1314, 1256, 1232, 1008, 942, 906, 843, 756.

rac-2'-Isopropoxy-4,4'-dimethyl-[1,1'-binaphthalen]-2-ol (rac-1l)

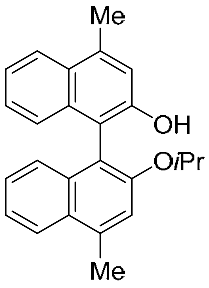

rac-**1l**
$C_{25}H_{24}O_2$
M = 356.47 g/mol

Prepared according to **GP2** from *rac*-**1n** (389 mg, 0.800 mmol, 1.00 equiv), methylboronic acid (192 mg, 3.20 mmol, 4.00 equiv), $Pd(dppf)Cl_2 \cdot CH_2Cl_2$ (65.3 mg, 80.0 µmol, 10.0 mol%) and caesium carbonate (1.04 g, 3.20 mmol, 4.00 equiv) in 1,4-dioxane (8 mL). The reaction mixture was refluxed for 26 h. Flash column chromatography on silica gel using cyclohexane/*tert*-butyl methyl ether (100:0 → 30:1 → 15:1 → 10:1 → 7:1) afforded the title compound *rac*-**1l** (242 mg, 0.679 mmol, 85%) as an orange solid.

The analytical data are consistent with those reported in section 4 for the enantioenriched alcohol.

rac-2'-Isopropoxy-4,4'-diphenyl-[1,1'-binaphthalen]-2-ol (rac-1m)

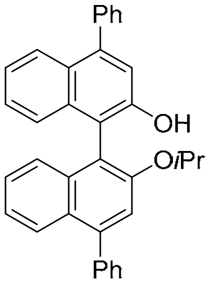

rac-**1m**
$C_{35}H_{28}O_2$
M = 480.61 g/mol

A Schlenk flask was charged with *rac*-**1n** (444 mg, 1.00 mmol, 1.00 equiv), phenylboronic acid (293 mg, 2.40 mmol, 2.40 equiv) and $Pd(PPh_3)_4$ (57.8 mg, 50.0 µmol, 5.00 mol%). The reaction vessel was evacuated and backfilled with nitrogen (3 x), and previously nitrogen-purged 1,2-dimethoxyethane (10 mL) and an aqueous 1.9 M solution of sodium carbonate (1.3 mL, nitrogen-purged) were added after one another. The reaction mixture was refluxed for 80 h,

cooled to room temperature, and filtered over silica. Flash column chromatography on silica gel using cyclohexane/ethyl acetate (30:1 → 15:1 → 10:1) afforded the title compound *rac*-**1m** (427 mg, 0.888 mmol, 89%) as a yellow solid.

M.p. = 82–86 °C (dichloromethane/*n*-pentane). **R$_f$** = 0.49 (cyclohexane/ethyl acetate = 15:1). **^{1}H NMR** (500 MHz, $CDCl_3$): δ/ppm = 7.92 (d, *J* = 7.1 Hz, 2H), 7.67–7.44 (m, 10H), 7.33–7.20 (m, 6H), 7.34 (s, 1H), 7.41 (s, 1H), 5.14 (s, br, 1H), 4.52 (sept, *J* = 6.1 Hz, 1H), 1.17 (d, *J* = 6.1 Hz, 3H), 1.05 (d, *J* = 6.1 Hz, 3H). **^{13}C{^{1}H} NMR** (126 MHz, $CDCl_3$): δ/ppm = 154.2, 150.9, 143.3, 142.2, 140.7, 140.6, 134.9, 134.6, 130.3 (2C), 130.2 (2C), 128.6 (2C), 128.4 (2C), 128.3, 127.8, 127.6, 127.5, 127.2, 126.6, 126.4, 126.2, 125.8, 125.8, 124.5, 123.3, 118.9, 118.7, 117.5, 115.3, 72.7, 22.5 (2C). **HRMS** (APCI) calculated for $C_{35}H_{29}O_2^+$ [(M+H)$^+$]: 481.2162; found: 481.2159. **IR** (ATR): ṽ/cm^{-1} = 3530, 3057, 2972, 1586, 1458, 1346, 1218, 1107, 875, 763, 702.

***rac*-7,7'-Dibromo-2'-isopropoxy-[1,1'-binaphthalen]-2-ol (*rac*-1p)**

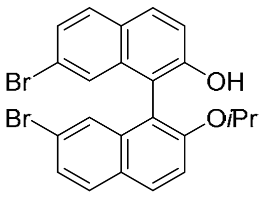

rac-**1p**
$C_{23}H_{18}Br_2O_2$
M = 486.20 g/mol

Prepared according to **GP1** from racemic 4,4'-dibromo-[1,1'-binaphthalene]-2,2'-diol (3.02 g, 6.80 mmol, 1.00 equiv), potassium carbonate (1.27 g, 9.18 mmol, 1.35 equiv) and 2-iodopropane (0.75 mL, 7.5 mmol, 1.1 equiv) in acetone (68 mL). The reaction mixture was refluxed for 38 h, cooled to room temperature, filtered over Celite® and the solvent was removed under reduced pressure. Flash column chromatography on silica gel using cyclohexane/dichloromethane (10:1 → 5:1 → 2:1 → 1:1 → 1:2) afforded the title compound *rac*-**1p** (1.30 g, 2.67 mmol, 39%) as a light yellow solid.

M.p. = 66–70 °C ($CHCl_3$). **R$_f$** = 0.24 (cyclohexane/dichloromethane = 1:1). **^{1}H NMR** (500 MHz, $CDCl_3$): δ/ppm = 7.97 (d, *J* = 9.1 Hz, 1H), 7.86 (d, *J* = 8.9 Hz, 1H), 7.75 (d, *J* = 8.8 Hz, 1H), 7.72 (d, *J* = 8.8 Hz, 1H), 7.45 (t, *J* = 8.8 Hz, 2H), 7.39 (dd, *J* = 8.6, 1.2 Hz, 1H), 7.34 (d, *J* = 8.9 Hz, 1H), 7.29 (s, 1H), 7.17 (s, 1H), 4.91 (s, br, 1H), 4.55 (sept, *J* = 6.0 Hz, 1H), 1.12 (d, *J* = 6.0 Hz, 3H), 1.05 (d, *J* = 6.0 Hz, 3H). **^{13}C{^{1}H} NMR** (126 MHz, $CDCl_3$): δ/ppm = 155.7, 152.2, 135.5, 135.1, 131.2, 130.0, 130.0, 129.9, 128.0, 128.0, 127.7, 127.0, 126.8, 126.8, 122.3, 121.1, 118.2, 117.1, 115.7, 114.3, 72.0, 22.3, 22.2. **HRMS** (APCI) calculated for $C_{23}H_{18}Br_2O_2^+$

[M+]: 483.9668; found: 483.9664. **IR** (ATR): ṽ/cm^{-1} = 3054, 3053, 2976, 1611, 1495, 1451, 1378, 1316, 1252, 1165, 1108, 1016, 908, 831, 734.

rac-2'-Isopropoxy-7,7'-dimethyl-[1,1'-binaphthalen]-2-ol (_rac_-1o)

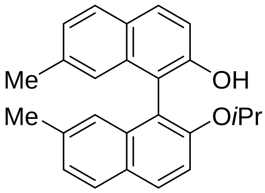

rac-**1o**
$C_{25}H_{24}O_2$
M = 356.47 g/mol

Prepared according to **GP2** from *rac*-**1p** (389 mg, 0.800 mmol, 1.00 equiv), methylboronic acid (192 mg, 3.20 mmol, 4.00 equiv), Pd(dppf)Cl_2·CH_2Cl_2 (65.3 mg, 80.0 µmol, 10.0 mol%) and caesium carbonate (1.04 g, 3.20 mmol, 4.00 equiv) in 1,4-dioxane (8 mL). The reaction mixture was refluxed for 26 h. Flash column chromatography on silica gel using cyclohexane/*tert*-butyl methyl ether (100:0 → 30:1 → 15:1 → 10:1) afforded the title compound *rac*-**1o** (199 mg, 0.558 mmol, 70%) as a pale yellow solid.

The analytical data are consistent with those reported in section 4 for the enantioenriched alcohol.

rac-6,6'-Dibromo-2'-isopropoxy-[1,1'-binaphthalen]-2-ol (_rac_-S1)

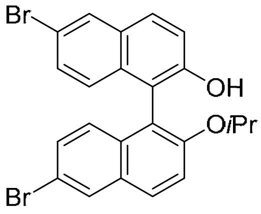

rac-**S1**
$C_{23}H_{18}Br_2O_2$
M = 486.20 g/mol

Prepared according to **GP1** from racemic 6,6'-dibromo-[1,1'-binaphthalene]-2,2'-diol (3.33 g, 7.50 mmol, 1.00 equiv), potassium carbonate (1.40 g, 10.1 mmol, 1.35 equiv) and 2-iodopropane (0.90 mL, 9.0 mmol, 1.1 equiv) in acetone (75 mL). The reaction mixture was refluxed for 17 h, cooled to room temperature, filtered over silica and loaded on Celite®. Flash column chromatography on silica gel using cyclohexane/dichloromethane (2:1 → 1:1) afforded the title compound *rac*-**S1** (2.51 g, 5.16 mmol, 69%) as a yellow solid.

M.p. = 66–68 °C (dichloromethane/*n*-pentane). $\mathbf{R_f}$ = 0.40 (cyclohexane/dichloromethane = 1:1). **^{1}H NMR** (500 MHz, $CDCl_3$): δ/ppm = 8.04 (d, *J* = 1.8 Hz, 1H), 8.00 (d, *J* = 1.7 Hz, 1H), 7.90 (d, *J* = 9.1 Hz, 1H), 7.80 (d, *J* = 8.9 Hz, 1H), 7.45 (d, *J* = 9.1 Hz, 1H), 7.35 (d, *J* = 8.9 Hz, 1H), 7.32 (dd, *J* = 9.1, 1.7 Hz, 1H), 7.28 (d, *J* = 1.8 Hz, 1H), 6.97 (d, *J* = 9.0 Hz, 1H), 6.87 (d, *J* = 9.0 Hz, 1H), 5.02 (s, 1H), 4.49 (sept, *J* = 6.1 Hz, 1H), 1.14 (d, *J* = 6.1 Hz, 3H), 1.00 (d, *J* = 6.1 Hz, 3H). **^{13}C{^{1}H} NMR** (126 MHz, $CDCl_3$): δ/ppm = 154.9, 151.8, 132.8, 132.4, 130.8, 130.6, 130.4, 130.2, 130.2, 130.1, 129.7, 129.1, 127.0, 126.9, 119.0, 118.4, 118.4, 117.5, 117.1, 115.4, 72.6, 22.3, 22.3. **HRMS** (APCI) calculated for $C_{23}H_{18}Br_2O_2^+$ [M$^+$]: 483.9668; found: 483.9669. **IR** (ATR): ṽ/cm^{-1} = 3503, 3057, 2974, 1583, 1490, 1378, 1348, 1260, 1107, 1005, 969, 875, 806, 766, 672.

***rac*-2'-Isopropoxy-6,6'-dimethyl-[1,1'-binaphthalen]-2-ol (*rac*-1q)**

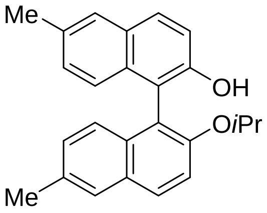

rac-**1q**
$C_{25}H_{24}O_2$
M = 356.47 g/mol

Prepared according to **GP2** from *rac*-**S1** (389 mg, 0.800 mmol, 1.00 equiv), methylboronic acid (192 mg, 3.20 mmol, 4.00 equiv), Pd(dppf)Cl_2·CH_2Cl_2 (65.3 mg, 80.0 µmol, 10.0 mol%) and caesium carbonate (1.04 g, 3.20 mmol, 4.00 equiv) in 1,4-dioxane (8 mL). The reaction mixture was refluxed for 16 h. Flash column chromatography on silica gel using cyclohexane/ethyl acetate (30:1 → 20:1 → 10:1 → 5:1) afforded the title compound *rac*-**1q** (209 mg, 0.586 mmol, 73%) as a pale yellow solid.

The analytical data are consistent with those reported in section 4 for the enantioenriched alcohol.

rac-2'-Isopropoxy-6,6'-diisopropyl-[1,1'-binaphthalen]-2-ol (rac-1r)

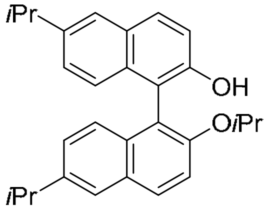

rac-**1r**
$C_{29}H_{32}O_2$
M = 412.57 g/mol

A dried Schlenk tube was charged with *rac*-**S1** (389 mg, 0.800 mmol, 1.00 equiv) and Pd(dppf)Cl_2·CH_2Cl_2 (65.3 mg, 80.0 µmol, 10.0 mol%) and dry THF (8 mL) was added. The mixture was cooled down to 0 °C, and a solution of isopropylmagnesium bromide (8.0 mL, 16 mmol, 20 equiv, 2.0 M in THF) was added dropwise over 15 min. After complete addition, the reaction mixture was heated to reflux and stirred for 25 h before being cooled to room temperature. A saturated aqueous solution of NH_4Cl (10 mL) and *tert*-butyl methyl ether (5 mL) were added, the phases were separated and the aqueous phase was extracted with *tert*-butyl methyl ether (3 x 50 mL). The combined organic layers were washed with saturated aqueous NaCl (150 mL), dried over Na_2SO_4, filtered and the solvent was removed under reduced pressure. Flash column chromatography on silica gel using cyclohexane/ethyl acetate (15:1 → 5:1) afforded the title compound *rac*-**1r** (206 mg, 0.499 mmol, 62%) as a yellow solid.

The analytical data are consistent with those reported in section 4 for the enantioenriched alcohol.

rac-2'-Isopropoxy-6,6'-diphenyl-[1,1'-binaphthalen]-2-ol (rac-1s)

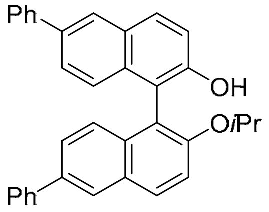

rac-**1s**
$C_{35}H_{28}O_2$
M = 480.61 g/mol

Prepared according to **GP2** from *rac*-**S1** (389 mg, 0.800 mmol, 1.00 equiv), phenylboronic acid (390 mg, 3.20 mmol, 4.00 equiv), Pd(dppf)Cl_2·CH_2Cl_2 (65.3 mg, 80.0 µmol, 10.0 mol%) and caesium carbonate (1.04 g, 3.20 mmol, 4.00 equiv) in 1,4-dioxane (8 mL). The reaction mixture

was refluxed for 20 h. Flash column chromatography on silica gel using cyclohexane/ethyl acetate (30:1 → 15:1 → 10:1 → 5:1) afforded the title compound *rac*-**1s** (336 mg, 0.699 mmol, 87%) as a pale yellow solid.

The analytical data are consistent with those reported in section 4 for the enantioenriched alcohol.

3.3 Synthesis of Spirocyclic *rac*-1v

rac-7'-Isopropoxy-2,2',3,3'-tetrahydro-1,1'-spirobi[inden]-7-ol (*rac*-1v)

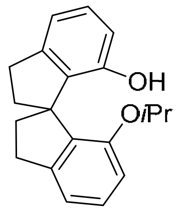

rac-**1v**
$C_{20}H_{22}O_2$
M = 294.39 g/mol

Prepared according to **GP1** from racemic 2,2',3,3'-tetrahydro-1,1'-spirobi[indene]-7,7'-diol (631 mg, 2.50 mmol, 1.00 equiv), potassium carbonate (432 mg, 2.50 mmol, 1.25 equiv) and 2-iodopropane (0.25 mL, 2.5 mmol, 1.0 equiv) in acetone (25 mL). The reaction mixture was refluxed for 20 h, cooled to room temperature and loaded on Celite®. Flash column chromatography on silica gel using cyclohexane/*tert*-butyl methyl ether (10:1) afforded the title compound *rac*-**1v** (252 mg, 0.856 mmol, 34%) as a colorless oil.

The analytical data are consistent with those reported in section 4 for the enantioenriched alcohol.

3.4 Synthesis of Biphenol-Substrates

Scheme S2. Synthesis of *rac*-**1t**.

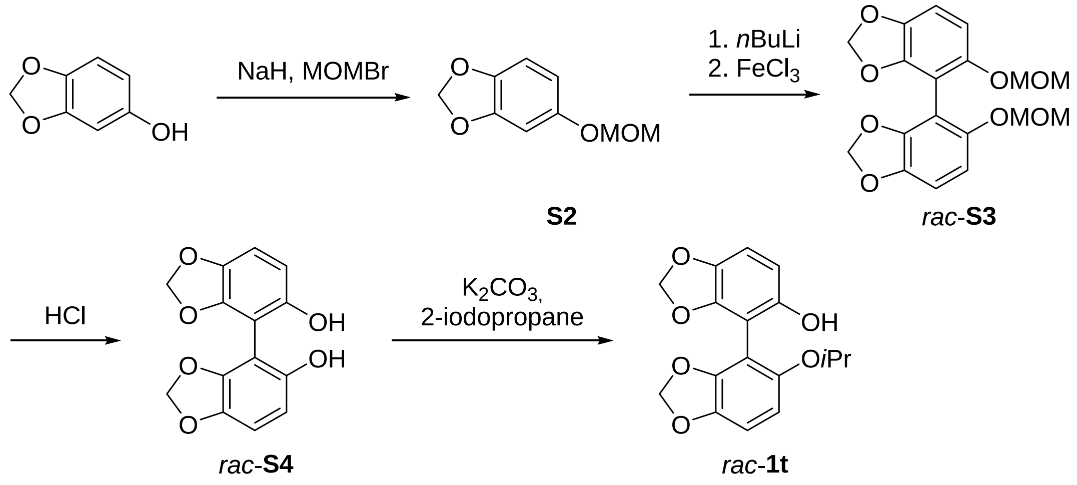

5-(Methoxymethoxy)benzo[*d*][1,3]dioxole (S2)

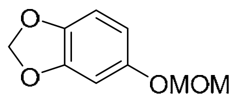

S2
$C_9H_{10}O_4$
M = 182.18 g/mol

In a dried Schlenk tube, sodium hydride (600 mg, 15.0 mmol, 1.5 equiv, 60 wt.% in paraffine oil) was suspended in dry THF (15 mL). The mixture was cooled to 0 °C, and a solution of benzo[*d*][1,3]dioxol-5-ol (1.38 g, 10.0 mmol, 1.0 equiv) in dry THF (15 mL) was added dropwise over 10 min. After stirring for 1.5 h, bromomethyl methyl ether (830 µL, 10.5 mmol, 1.05 equiv) was added in one portion. The reaction mixture was slowly warmed to room temperature and stirred for 24 h, then cooled to 0 °C again, followed by dropwise addition of water (10 mL). Then, it was diluted with water (20 mL) and *tert*-butyl methyl ether (20 mL), the phases were separated, and the aqueous phase was extracted with *tert*-butyl methyl ether (3 x 40 mL). The combined organic phases were dried over Na_2SO_4, filtered and the solvent was removed under reduced pressure. The crude product was applied on a plug of silica, first washed with cyclohexane and then eluted with dichloromethane. After evaporation of the solvent, the title compound **S2** (1.67 g, 9.17 mmol, 92%) was obtained a pale yellow oil.

^{1}H NMR (400 MHz, $CDCl_3$): δ/ppm = 6.71 (d, *J* = 8.5 Hz, 1H), 6.62 (d, *J* = 2.5 Hz, 1H), 6.49 (dd, *J* = 8.5, 2.5 Hz, 1H), 5.92 (s, 2H), 5.08 (s, 2H), 3.48 (s, 3H). **^{13}C{^{1}H} NMR** (101 MHz, $CDCl_3$): δ/ppm = 152.7, 148.3, 142.7, 108.6, 108.2, 101.3, 99.9, 95.7, 56.1.

The NMR spectroscopic data are consistent with those reported in the literature.[9]

rac-5,5'-Bis(methoxymethoxy)-4,4'-bibenzo[d][1,3]dioxole (rac-S3)

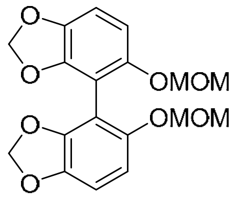

rac-**S3**
$C_{18}H_{18}O_8$
M = 362.33 g/mol

In a dried Schlenk tube, **S2** (1.16 g, 6.37 mmol, 1.00 equiv) was dissolved in dry diethylether (60 mL). The mixture was cooled to 0 °C, and *n*-butyllithium (2.5 mL, 6.4 mmol, 1.0 equiv, 2.5 M in *n*-hexane) was added dropwise over 10 min. The solution was stirred for 15 min at 0 °C, and then for another 2 h at room temperature. Subsequently, the reaction mixture was cooled to 0 °C again, and anhydrous $FeCl_3$ (1.14 g, 7.01 mmol, 1.10 equiv) was added in one portion. The solution was slowly warmed to room temperature and stirred for 16 h, and the reaction was quenched by addition of 2 M aqueous HCl (10 mL). The mixture was diluted with water (50 mL) and diethylether (20 mL), the phases were separated and the aqueous phase was extracted with diethyl ether (4 x 75 mL). The combined organic phases were dried over Na_2SO_4, filtered and the solvent was removed under reduced pressure. Flash column chromatography on silica gel using cyclohexane/ethyl acetate (5:1 → 3:1) afforded the title compound *rac*-**S3** (801 mg, 2.21 mmol, 69%) as a sticky yellow resin.

R_f = 0.31 (cyclohexane/ethyl acetate = 5:1). **^{1}H NMR** (500 MHz, $CDCl_3$): δ/ppm = 6.76 (d, *J* = 8.5 Hz, 2H), 6.69 (d, *J* = 8.5 Hz, 2H), 5.93 (d, *J* = 6.6 Hz, 4H), 5.00 (s, 4H), 3.35 (s, 6H). **^{13}C{^{1}H} NMR** (126 MHz, $CDCl_3$): δ/ppm = 150.8 (2C), 146.7 (2C), 142.7 (2C), 108.5 (2C), 107.8 (2C), 106.9 (2C), 101.5 (2C), 96.6 (2C), 56.0 (2C). **HRMS** (APCI) calculated for $C_{18}H_{19}O_8^+$ [(M+H)$^+$]: 363.1074; found: 363.1073. **IR** (ATR): ṽ/cm^{-1} = 2987, 1443, 1239, 1153, 1048, 920, 796.

rac-[4,4'-Bibenzo[_d_][1,3]dioxole]-5,5'-diol (_rac_-S4)

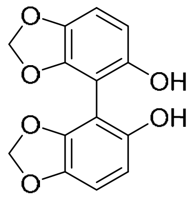

rac-**S4**
$C_{14}H_{10}O_6$
M = 274.23 g/mol

In a round bottom flask open to air, *rac*-**S3** (757 mg, 2.09 mmol, 1.00 equiv) was dissolved in methanol (20 mL) and concentrated aqueous HCl (0.3 mL) was added. The mixture was refluxed for 1.5 h and cooled to room temperature, followed by dilution with water (20 mL) and *tert*-butyl methyl ether (20 mL). The phases were separated, and the aqueous phase was extracted with *tert*-butyl methyl ether (4 x 40 mL). The combined organic phases were dried over Na_2SO_4, filtered and the solvents were removed under reduced pressure. Filtration over a plug of silica (eluted with ethyl acetate) afforded the title compound *rac*-**S4** (534 mg, 1.95 mmol, 93%) as a slightly brown solid.

^{1}H NMR (400 MHz, acetone-d_6): δ/ppm = 8.19 (s, br, 2H), 6.68 (d, *J* = 8.4 Hz, 2H), 6.42 (d, *J* = 8.4 Hz, 2H), 5.89 (d, *J* = 1.1 Hz, 2H), 5.88 (d, *J* = 1.1 Hz, 2H). **^{13}C{^{1}H} NMR** (101 MHz, acetone-d_6): δ/ppm = 151.1 (2C), 147.6 (2C), 141.4 (2C), 108.3 (2C), 107.7 (2C), 105.2 (2C), 101.8 (2C).

The NMR spectroscopic data are consistent with those reported in the literature.[10]

rac-5'-Isopropoxy-[4,4'-bibenzo[_d_][1,3]dioxol]-5-ol (_rac_-1t)

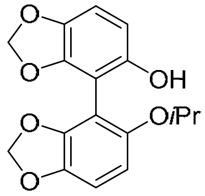

rac-**1t**
$C_{17}H_{16}O_6$
M = 316.31 g/mol

Prepared according to **GP1** from *rac*-**S4** (274 mg, 1.00 mmol, 1.00 equiv), potassium carbonate (187 mg, 1.35 mmol, 1.35 equiv) and 2-iodopropane (0.11 mL, 1.1 mmol, 1.1 equiv) in acetone (10 mL). The reaction mixture was refluxed for 19 h, cooled to room temperature and the solvent was evaporated under reduced pressure. The residue was suspended in

dichloromethane and filtered over Celite®. Flash column chromatography on silica gel using cyclohexane/dichloromethane (3:1 → 2:1 → 3:2 → 1:2 → 1:3) afforded the title compound *rac*-**1t** (163 mg, 0.515 mmol, 52%) as a sticky colorless resin.

R_f = 0.19 (cyclohexane/dichloromethane = 1:1). **^{1}H NMR** (400 MHz, C_6D_6): δ/ppm = 6.71 (d, *J* = 8.5 Hz, 1H), 6.65 (d, *J* = 8.4 Hz, 1H), 6.57 (d, *J* = 8.5 Hz, 1H), 6.48 (s, 1H), 6.15 (d, *J* = 8.4 Hz, 1H), 5.49 (d, *J* = 1.4 Hz, 1H), 5.43 (d, *J* = 1.4 Hz, 1H), 5.38 (d, *J* = 1.4 Hz, 1H), 5.20 (d, *J* = 1.4 Hz, 1H), 3.91 (sept, *J* = 6.1 Hz, 1H), 0.94 (d, *J* = 6.1 Hz, 3H), 0.87 (d, *J* = 6.1 Hz, 3H). **^{13}C{^{1}H} NMR** (101 MHz, C_6D_6): δ/ppm = 150.2, 149.8, 147.6, 146.9, 143.5, 141.8, 109.9, 109.4, 108.8, 108.2, 107.8, 106.0, 101.5, 101.1, 74.3, 21.8, 21.5. **HRMS** (APCI) calculated for $C_{17}H_{17}O_6^+$ [(M+H)$^+$]: 317.1020; found: 317.1018. **IR** (ATR): ṽ/cm^{-1} = 3365 (br), 2973, 2891, 1636, 1435, 1381, 1233, 1195, 1106, 1043, 927, 853, 791.

The racemic diol precursors for the synthesis of *rac*-**1m** (from *rac*-6,6'-dimethoxy-[1,1'-biphenyl]-2,2'-diol) and *rac*-**1p** (from *rac*-5,5',6,6'-tetramethyl-[1,1'-biphenyl]-2,2'-diol) were prepared according to literature procedures.[11,12]

rac-2'-Isopropoxy-6,6'-dimethoxy-[1,1'-biphenyl]-2-ol (_rac_-1u)

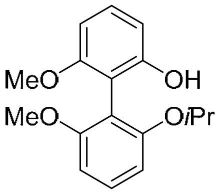

rac-**1u**
$C_{17}H_{20}O_4$
M = 288.34 g/mol

Prepared according to **GP1** from racemic 6,6'-dimethoxy-[1,1'-biphenyl]-2,2'-diol (263 mg, 1.07 mmol, 1.00 equiv), potassium carbonate (199 mg, 1.44 mmol, 1.35 equiv) and 2-iodopropane (0.12 mL, 1.2 mmol, 1.1 equiv) in acetone (11 mL). The reaction mixture was refluxed for 24 h, cooled to room temperature and loaded on Celite®. Flash column chromatography on silica gel using cyclohexane/dichloromethane (2:1 → 1:1 → 1:2) afforded the title compound *rac*-**1u** (163 mg, 0.565 mmol, 53%) as a colorless solid.

M.p. = 71–74 °C (dichloromethane/*n*-pentane). $\mathbf{R_f}$ = 0.08 (cyclohexane/dichloromethane = 1:1). **^{1}H NMR** (500 MHz, $CDCl_3$): δ/ppm = 7.32 (t, *J* = 8.3 Hz, 1H), 7.23 (t, *J* = 8.2 Hz, 1H), 6.71–6.66 (m, 3H), 6.57 (d, *J* = 8.2 Hz, 1H), 5.16 (s, 1H), 4.36 (sept, *J* = 6.1 Hz, 1H), 3.75 (s, 3H), 3.73 (s, 3H), 1.18 (d, *J* = 6.1 Hz, 3H), 1.14 (d, *J* = 6.0 Hz, 3H). **^{13}C{^{1}H} NMR** (126 MHz, $CDCl_3$): δ/ppm = 159.0, 158.5, 157.2, 154.5, 129.9, 129.0, 112.0, 111.0, 108.9, 108.9, 104.8, 103.5, 72.0, 56.2, 56.1, 22.2, 22.0. **HRMS** (APCI) calculated for $C_{17}H_{21}O_4^+$ [$(M+H)^+$]: 289.1434; found: 289.1431. **IR** (ATR): ṽ/cm^{-1} = 3489, 3425, 2971, 2927, 2834, 1583, 1461, 1305, 1246, 1201, 1169, 1081, 982, 841, 778, 726.

rac-6'-Isopropoxy-2',3',5,6-tetramethyl-[1,1'-biphenyl]-2-ol (_rac_-1z)

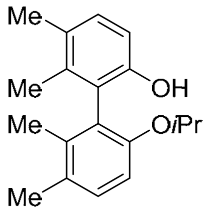

rac-**1z**
$C_{19}H_{24}O_2$
M = 284.40 g/mol

Prepared according to **GP1** from racemic 5,5',6,6'-tetramethyl-[1,1'-biphenyl]-2,2'-diol (197 mg, 0.813 mmol, 1.00 equiv), potassium carbonate (152 mg, 1.10 mmol, 1.35 equiv) and 2-iodopropane (90 µL, 0.89 mmol, 1.1 equiv) in acetone (8 mL). The reaction mixture was refluxed for 19 h, cooled to room temperature and loaded on Celite®. Flash column chromatography on silica gel using cyclohexane/dichloromethane (2:1 → 1:1) afforded the title compound *rac*-**1z** (136 mg, 0.478 mmol, 59%) as colorless crystals.

The analytical data are consistent with those reported in section 4 for the enantioenriched alcohol.

Scheme S3. Synthesis of *rac*-**1w** and *rac*-**1a'**.

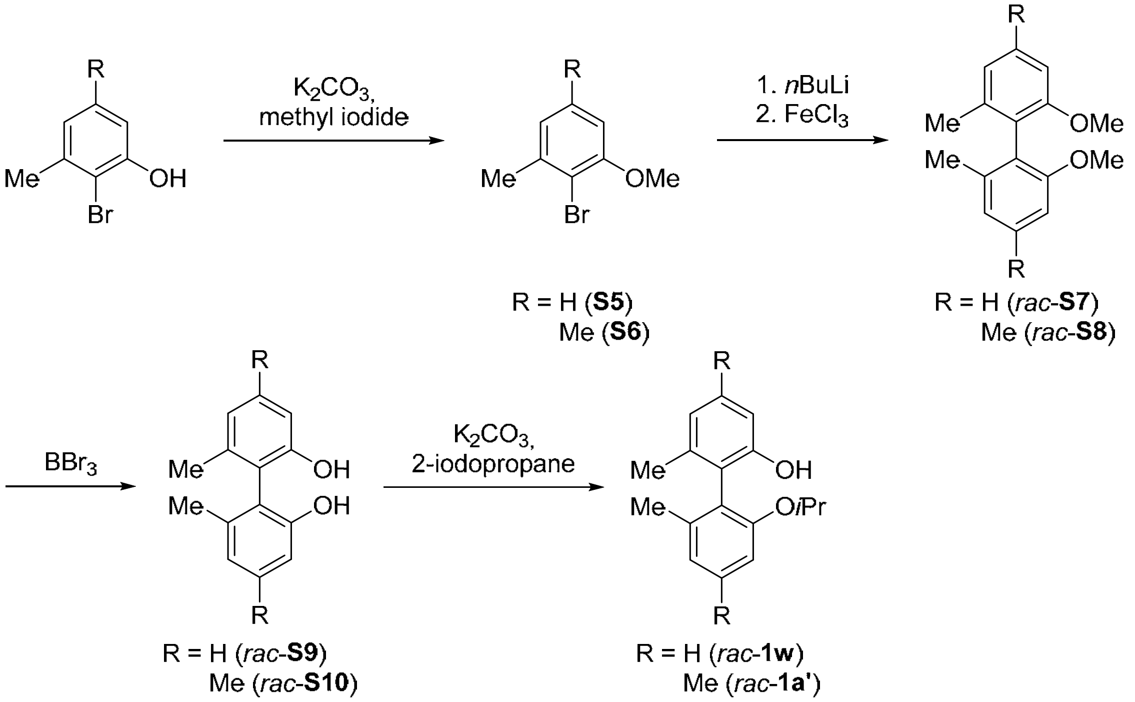

2-Bromo-1-methoxy-3-methylbenzene (S5)

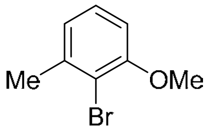

S5
C_8H_9BrO
M = 201.06 g/mol

Prepared according to **GP1** from 2-bromo-3-methylphenol (1.87 g, 10.0 mmol, 1.00 equiv), potassium carbonate (1.73 g, 12.5 mmol, 1.25 equiv) and methyl iodide (0.75 mL, 12 mmol, 1.2 equiv) in acetone (100 mL). The reaction mixture was stirred for 5 d at room temperature, and then the solvent was removed under reduced pressure. The residue was suspended in dichloromethane and filtered over Celite®. The solvent was removed under reduced pressure, afterwards the crude product was filtered over a plug of silica and eluted with dichloromethane. The title compound **S5** (1.90 g, 9.45 mmol, 94%) was obtained as a colorless oil that solidified upon standing.

^{1}H NMR (400 MHz, $CDCl_3$): δ/ppm = 7.16 (t, *J* = 7.9 Hz, 1H), 6.87 (d, *J* = 7.8 Hz, 1H), 6.75 (d, *J* = 8.0 Hz, 1H), 3.89 (s, 3H), 2.42 (s, 3H). **^{13}C{^{1}H} NMR** (101 MHz, $CDCl_3$): δ/ppm = 156.1, 139.9, 127.6, 123.1, 114.4, 109.3, 56.4, 23.4.

The NMR spectroscopic data are consistent with those reported in the literature.[13]

rac-2,2'-Dimethoxy-6,6'-dimethyl-1,1'-biphenyl (_rac_-S7)

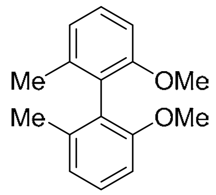

rac-**S7**
$C_{16}H_{18}O_2$
M = 242.32 g/mol

In a dried Schlenk tube, **S5** (1.54 g, 7.66 mmol, 1.00 equiv) was dissolved in dry THF (80 mL). The solution was cooled to –78 °C, and *n*-butyllithium (3.1 mL, 7.7 mmol, 1.0 equiv, 2.5 M in *n*-hexane) was added dropwise over 20 min. The solution was stirred for 40 min at –78 °C, and then anhydrous $FeCl_3$ (1.37 g, 8.43 mmol, 1.1 equiv) was added in one portion. The mixture was slowly warmed to room temperature over 12 h, and the reaction was quenched by addition of 2 M HCl (8 mL). The phases were separated, and the aqueous phase was extracted with *tert*-butyl methyl ether (3 x 100 mL). The combined organic layers were washed with saturated aqueous NaCl-solution (2 x 30 mL), dried over Na_2SO_4, filtered, and the solvents were removed under reduced pressure. Flash column chromatography on silica gel using cyclohexane/ethyl acetate (50:1) afforded the title compound *rac*-**S7** (679 mg, 2.80 mmol, 73%) as a pale yellow solid.

^{1}H NMR (400 MHz, $CDCl_3$): δ/ppm = 7.24 (t, *J* = 7.9 Hz, 2H), 6.91 (d, *J* = 7.7 Hz, 2H), 6.82 (d, *J* = 8.2 Hz, 2H), 3.70 (s, 6H), 1.94 (s, 6H). **^{13}C{^{1}H} NMR** (101 MHz, $CDCl_3$): δ/ppm = 157.1 (2C), 138.3 (2C), 128.0 (2C), 126.3 (2C), 122.3 (2C), 108.5 (2C), 55.9 (2C), 19.7 (2C).

The NMR spectroscopic data are consistent with those reported in the literature.[14]

rac-6,6'-Dimethyl-[1,1'-biphenyl]-2,2'-diol (_rac_-S9)

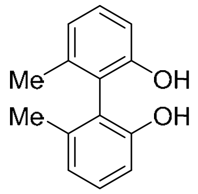

rac-**S9**
$C_{14}H_{14}O_2$
M = 214.26 g/mol

Prepared according to **GP3** from *rac*-**S7** (679 mg, 2.80 mmol, 1.00 equiv) and boron tribromide (5.6 mL, 5.6 mmol, 2.0 equiv, 1.0 M in dichloromethane) in dry dichloromethane (5.6 mL). After stirring for 14 h, the reaction was quenched by addition of water (20 mL), the phases were separated, and the aqueous phase was extracted with dichloromethane (3 x 30 mL). The combined organic layers were dried over Na_2SO_4, filtered, and the solvent was removed under reduced pressure. Flash column chromatography on silica gel using cyclohexane/ethyl acetate afforded the title compound *rac*-**S9** (531 mg, 2.48 mmol, 89%) as a pale yellow solid.

^{1}H NMR (400 MHz, $CDCl_3$): δ/ppm = 7.26 (t, *J* = 8.0 Hz, 2H), 6.93 (d, *J* = 7.7 Hz, 2H), 6.90 (d, *J* = 8.2 Hz, 2H), 4.67 (s, br, 2H), 2.01 (s, 6H). **^{13}C{^{1}H} NMR** (101 MHz, $CDCl_3$): δ/ppm = 154.0 (2C), 139.1 (2C), 130.3 (2C), 122.8 (2C), 119.6 (2C), 113.3 (2C), 19.6 (2C).

The NMR spectroscopic data are consistent with those reported in the literature.[14]

rac-2'-Isopropoxy-6,6'-dimethyl-[1,1'-biphenyl]-2-ol (_rac_-1w)

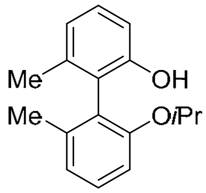

rac-**1w**
$C_{17}H_{20}O_2$
M = 256.35 g/mol

Prepared according to **GP1** from *rac*-**S9** (521 mg, 2.43 mmol, 1.00 equiv), potassium carbonate (453 mg, 3.28 mmol, 1.35 equiv) and 2-iodopropane (0.27 mL, 2.7 mmol, 1.1 equiv) in acetone (24 mL). The reaction mixture was refluxed for 21 h, cooled to room temperature and loaded on Celite®. Flash column chromatography on silica gel using cyclohexane/dichloromethane (1:1) afforded the title compound *rac*-**1w** (299 mg, 1.17 mmol, 48%) as a colorless resin.

The analytical data are consistent with those reported in section 4 for the enantioenriched alcohol.

2-Bromo-1-methoxy-3,5-dimethylbenzene (S6)

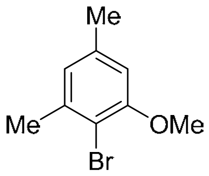

S6
$C_9H_{11}BrO$
M = 215.09 g/mol

Prepared according to **GP1** from 2-bromo-3,5-dimethylphenol (1.00 g, 4.97 mmol, 1.00 equiv), potassium carbonate (859 g, 6.22 mmol, 1.25 equiv) and methyl iodide (0.40 mL, 6.46 mmol, 1.3 equiv) in acetone (50 mL). The reaction mixture was stirred for 5 d at room temperature, and then filtered over a plug of silica. The solvents were evaporated and flash column chromatography on silica gel using cyclohexane afforded the title compound **S6** (1.07 g, 4.97 mmol, quant.) as a colorless solid.

M.p. = 40–42 °C (dichloromethane/*n*-pentane). **R**$_f$ = 0.14 (cyclohexane). ^{1}**H NMR** (500 MHz, $CDCl_3$): δ/ppm = 6.69 (s, 1H), 6.56 (s, 1H), 3.87 (s, 3H), 2.37 (s, 3H), 2.29 (s, 3H). ^{13}**C{**^{1}**H} NMR** (126 MHz, $CDCl_3$): δ/ppm = 155.9, 139.3, 137.7, 123.9, 111.0, 110.3, 59.4, 23.3, 21.4. **HRMS** (APCI) calculated for $C_9H_{12}BrO^+$ [$(M+H)^+$]: 215.0066; found: 215.0067. **IR** (ATR): ṽ/cm^{-1} = 2918, 2852, 1579, 1461, 1404, 1319, 1241, 1178, 1094, 1028, 828.

***rac*-2,2'-Dimethoxy-4,4',6,6'-tetramethyl-1,1'-biphenyl (*rac*-S8)**

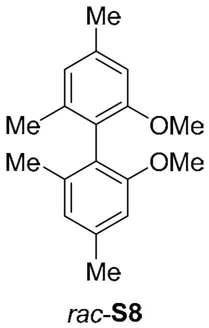

rac-**S8**
$C_{18}H_{22}O_2$
M = 270.37 g/mol

In a dried Schlenk tube, **S6** (1.10 g, 5.11 mmol, 1.00 equiv) was dissolved in dry THF (50 mL). The solution was cooled to –78 °C, and *n*-butyllithium (2.1 mL, 5.1 mmol, 1.0 equiv, 2.8 M in *n*-hexane) was added dropwise over 10 min. The solution was stirred for 40 min at –78 °C, and then anhydrous $FeCl_3$ (912 mg, 5.62 mmol, 1.10 equiv) was added in one portion. The mixture was slowly warmed to room temperature over 16 h, and the reaction was quenched

by addition of 2 M HCl (5 mL). After dilution with water (20 mL), the phases were separated and the aqueous phase was extracted with *tert*-butyl methyl ether (3 x 25 mL). The combined organic layers were washed with saturated aqueous NaCl-solution (3 x 25 mL), dried over Na_2SO_4, filtered, and the solvents were removed under reduced pressure. Flash column chromatography on silica gel using cyclohexane/ethyl acetate (50:1) afforded the title compound *rac*-**S8** (342 mg, 1.26 mmol, 49%) as a pale yellow solid.

M.p. = 86–88 °C (dichloromethane/*n*-pentane). $\mathbf{R_f}$ = 0.14 (cyclohexane/ethyl acetate = 50:1). **^{1}H NMR** (500 MHz, $CDCl_3$): δ/ppm = 6.73 (s, 2H), 6.63 (s, 2H), 3.68 (s, 6H), 2.36 (s, 6H), 1.91 (s, 6H). **^{13}C{^{1}H} NMR** (126 MHz, $CDCl_3$): δ/ppm = 157.1 (2C), 138.2 (2C), 137.6 (2C), 123.3 (2C), 123.1 (2C), 109.5 (2C), 55.9 (2C), 21.8 (2C), 19.7 (2C). **HRMS** (APCI) calculated for $C_{18}H_{23}O_2^+$ [(M+H)$^+$]: 271.1693; found: 271.1691. **IR** (ATR): $\tilde{\nu}$/cm^{-1} = 2919, 2855, 1575, 1461, 1312, 1235, 1171, 1096, 1001, 921, 828.

***rac*-4,4',6,6'-Tetramethyl-[1,1'-biphenyl]-2,2'-diol (*rac*-S10)**

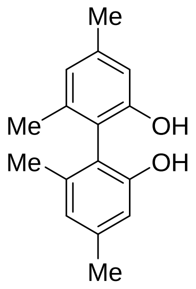

rac-**S10**
$C_{16}H_{18}O_2$
M = 242.32 g/mol

Prepared according to **GP3** from *rac*-**S8** (334 mg, 1.24 mmol, 1.00 equiv) and boron tribromide (2.5 mL, 2.5 mmol, 2.0 equiv, 1.0 M in dichloromethane) in dry dichloromethane (2.5 mL). After stirring for 26 h, the reaction was quenched by addition of water (3 mL), the phases were separated, and the aqueous phase was extracted with dichloromethane (3 x 4 mL). The combined organic layers were dried over Na_2SO_4, filtered, and the solvent was removed under reduced pressure. Filtration over a plug of silica using *tert*-butyl methyl ether afforded the diol *rac*-**S10** (300 mg, 1.24 mmol, quant.) as a pale yellow solid.

^{1}H NMR (500 MHz, $CDCl_3$): δ/ppm = 6.75 (s, 2H), 6.72 (s, 2H), 4.64 (s, 2H), 2.33 (s, 6H), 1.97 (s, 6H). **^{13}C{^{1}H} NMR** (126 MHz, $CDCl_3$): δ/ppm = 154.0 (2C), 140.3 (2C), 138.9 (2C), 123.7 (2C), 116.6 (2C), 113.8 (2C), 21.5 (2C), 19.6 (2C).

The NMR spectroscopic data are consistent with those reported in the literature.[15]

rac-2'-Isopropoxy-4,4',6,6'-tetramethyl-[1,1'-biphenyl]-2-ol (rac-1a')

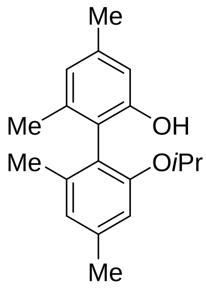

rac-**1a'**
$C_{19}H_{24}O_2$
M = 284.40 g/mol

Prepared according to **GP1** from *rac*-**S10** (314 mg, 1.24 mmol, 1.00 equiv), potassium carbonate (231 mg, 1.67 mmol, 1.35 equiv) and 2-iodopropane (0.14 mL, 1.7 mmol, 1.1 equiv) in acetone (15 mL). The reaction mixture was refluxed for 5 d, cooled to room temperature and filtered over a plug of silica. Flash column chromatography on silica gel using cyclohexane/ethyl acetate (70:1 → 50:1) afforded *rac*-**1a’** (176 mg, 0.619 mmol, 50%) together with some unknown impurities. Preparative TLC (cyclohexane/ethyl acetate = 5:1) afforded the title compound *rac*-**1a’** (90.6 mg, 0.319 mmol, 26%) in an analytically pure form as a yellow oil.

The analytical data are consistent with those reported in section 4 for the enantioenriched alcohol.

Scheme S4. Synthesis of biphenols *rac*-**1x**, *rac*-**1y** and *rac*-**1b'**.

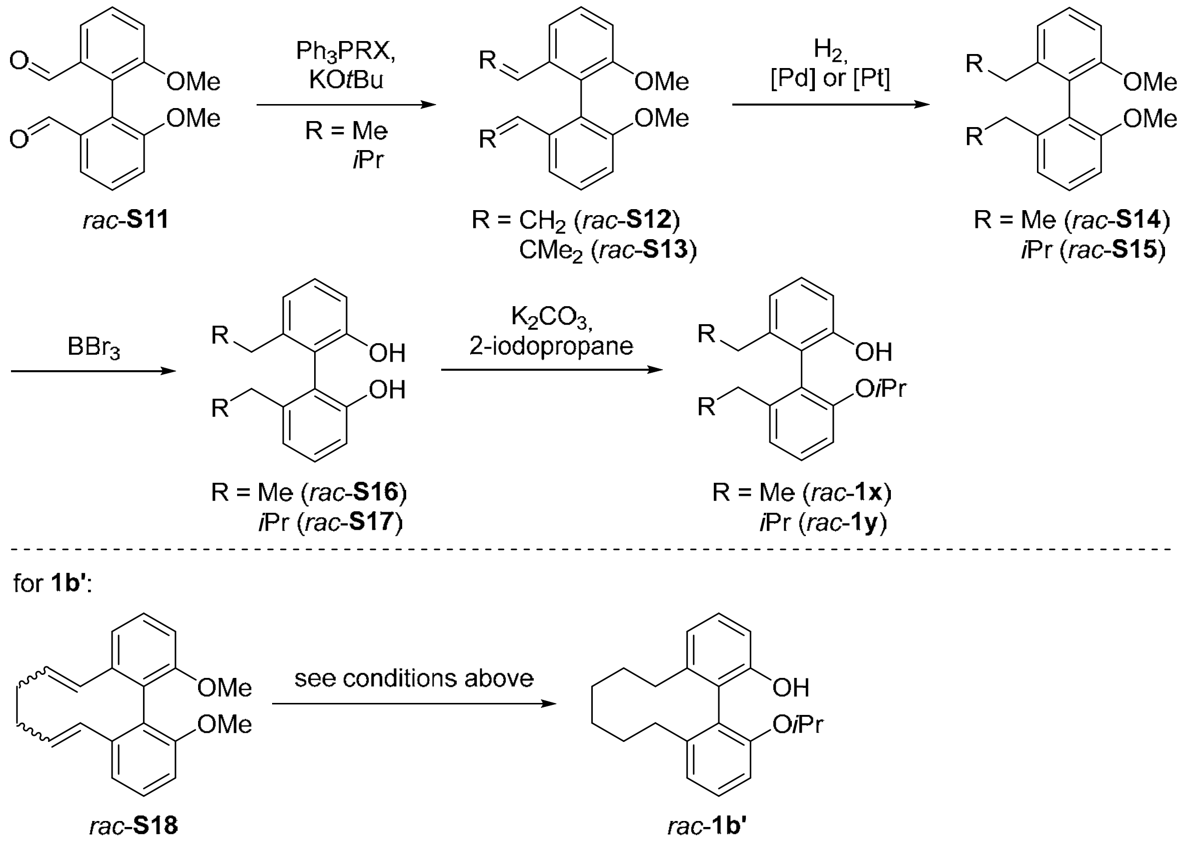

The precursors *rac*-**S11** and *rac*-**S18** were prepared according to literature procedures.[16,17]

rac-2,2'-Dimethoxy-6,6'-divinyl-1,1'-biphenyl (*rac*-S12)

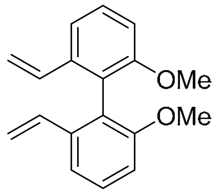

rac-**S12**
$C_{18}H_{18}O_2$
M = 266.34 g/mol

In a dried Schlenk tube, triphenylmethylphosphonium bromide (1.43 g, 4.00 mmol, 4.00 equiv) was suspended in dry THF (4 mL), followed by addition of potassium *tert*-butanolate (449 mg, 4.00 mmol, 4.00 equiv). The mixture was stirred for 4 h at room temperature, and afterwards a solution of the bisaldehyde *rac*-**S11** (270 mg, 1.00 mmol, 1.00 equiv) in dry THF (1 mL) was added. After stirring for 16 h, the suspension was filtered over silica and eluted with diethyl ether. The solvent was removed under removed pressure, and flash column chromatography

on silica gel using cyclohexane/ethyl acetate (10:1) afforded the title compound *rac*-**S12** (209 mg, 0.785 mmol, 79%) as a pale yellow solid.

^{1}H NMR (500 MHz, $CDCl_3$): δ/ppm = 7.36–7.29 (m, 4H), 6.90 (dd, *J* = 7.8, 1.3 Hz, 2H), 6.25 (dd, *J* = 17.5, 11.0 Hz, 2H), 5.62 (dd, *J* = 17.5, 1.3 Hz, 2H), 5.04 (dd, *J* = 11.0, 1.3 Hz, 2H), 3.69 (s, 6H). **$^{13}C\{^1H\}$ NMR** (126 MHz, $CDCl_3$): δ/ppm = 157.6 (2C), 138.2 (2C), 135.1 (2C), 128.5 (2C), 124.8 (2C), 117.3 (2C), 114.8 (2C), 110.3 (2C), 56.2 (2C).

***rac*-2,2'-Diethyl-6,6'-dimethoxy-1,1'-biphenyl (*rac*-S14)**

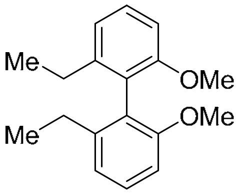

rac-**S14**
$C_{18}H_{22}O_2$
M = 270.37 g/mol

In a Schlenk flask, *rac*-**S12** (189 mg, 0.710 mmol, 1.00 equiv) and palladium on carbon (90.0 mg, 84.6 µmol, 12 mol %, 10 wt.% Pd) were suspended in dichloromethane/methanol (v/v = 2:1, 15 mL), and the mixture was degassed with three freeze-pump-thaw cycles. Then, the mixture was purged with hydrogen for 15 min and equipped with a hydrogen balloon. After stirring for 15 h at room temperature, GLC indicated full conversion, and the suspension was filtered over Celite®. The solvents were removed under reduced pressure, and the crude product was used for the next step without further purification.

***rac*-6,6'-Diethyl-[1,1'-biphenyl]-2,2'-diol (*rac*-S16)**

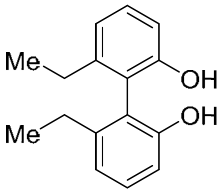

rac-**S16**
$C_{16}H_{18}O_2$
M = 242.32 g/mol

Prepared according to **GP3** from *rac*-**S14** (192 mg, 0.710 mmol, 1.00 equiv) and boron tribromide (1.4 mL, 1.4 mmol, 2.0 equiv, 1.0 M in dichloromethane) in dry dichloromethane (1.4 mL). After stirring for 17 h, the reaction was quenched by addition of water (3 mL), the phases were separated, and the aqueous phase was extracted with dichloromethane (4 x 4

mL). The combined organic layers were dried over Na_2SO_4, filtered, and the solvent was removed under reduced pressure. Filtration over a plug of silica using *tert*-butyl methyl ether afforded *rac*-**S16** (172 mg, 0.710 mmol, quant.) as a colorless solid.

^{1}H NMR (500 MHz, $CDCl_3$): δ/ppm = 7.33 (t, *J* = 8.0 Hz, 2H), 6.98 (d, *J* = 7.6 Hz, 2H), 6.91 (d, *J* = 8.2 Hz, 2H), 4.65 (s, br, 2H), 2.30 (q, *J* = 7.6 Hz, 4H), 1.07 (t, *J* = 7.6 Hz, 6H). **^{13}C{^{1}H} NMR** (126 MHz, $CDCl_3$): δ/ppm = 154.0 (2C), 145.2 (2C), 130.6 (2C), 121.0 (2C), 118.8 (2C), 113.3 (2C), 26.3 (2C), 14.8 (2C).

The NMR spectroscopic data are consistent with those reported in the literature.[18]

rac-2',6-Diethyl-6'-isopropoxy-[1,1'-biphenyl]-2-ol (*rac*-1x)

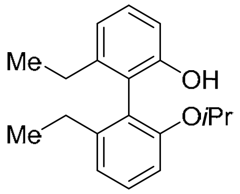

rac-**1x**
$C_{19}H_{24}O_2$
M = 284.40 g/mol

Prepared according to **GP1** from racemic diol **S14** (183 mg, 0.755 mmol, 1.00 equiv), potassium carbonate (141 mg, 1.02 mmol, 1.35 equiv) and 2-iodopropane (83 µL, 0.83 mmol, 1.1 equiv) in acetone (7.5 mL). The reaction mixture was refluxed for 20 h, cooled to room temperature and loaded on Celite®. Flash column chromatography on silica gel using cyclohexane/dichloromethane (2:1 → 1:1) afforded the title compound *rac*-**1x** (92.5 mg, 0.325 mmol, 43%) as a yellow oil.

The analytical data are consistent with those reported in section 4 for the enantioenriched alcohol.

rac-2,2'-Dimethoxy-6,6'-bis(2-methylprop-1-en-1-yl)-1,1'-biphenyl (rac-S13)

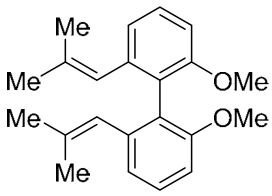

rac-**S13**
$C_{22}H_{26}O_2$
M = 322.45 g/mol

In a dried Schlenk tube, isopropyltriphenylphosphonium iodide (1.73 g, 4.00 mmol, 4.00 equiv) was suspended in dry THF (4 mL), followed by addition of potassium *tert*-butanolate (449 mg, 4.00 mmol, 4.00 equiv). The mixture was stirred for 4 h at room temperature, and afterwards a solution of the bisaldehyde *rac*-**S11** (270 mg, 1.00 mmol, 1.00 equiv) in dry THF (1 mL) was added. After stirring for 16 h, the suspension was filtered over silica and eluted with diethyl ether. The solvent was removed under reduced pressure, and flash column chromatography on silica gel using cyclohexane/ethyl acetate (20:1) afforded the title compound *rac*-**S13** (295 mg, 0.915 mmol, 92%) as a sticky yellow resin.

^{1}H NMR (500 MHz, $CDCl_3$): δ/ppm = 7.26 (t, *J* = 8.0 Hz, 2H), 6.88 (d, *J* = 7.9 Hz, 2H), 6.82 (d, *J* = 8.1 Hz, 2H), 5.64 (s, br, 2H), 3.68 (s, 6H), 1.68 (d, *J* = 1.4 Hz, 6H), 1.62 (d, *J* = 1.4 Hz, 6H). **^{13}C{^{1}H} NMR** (126 MHz, $CDCl_3$): δ/ppm = 157.1 (2C), 139.8 (2C), 134.2 (2C), 127.5 (2C), 126.0 (2C), 124.6 (2C), 122.3 (2C), 108.9 (2C), 56.0 (2C), 26.2 (2C), 19.3 (2C).

rac-2,2'-Diisobutyl-6,6'-dimethoxy-1,1'-biphenyl (rac-S15)

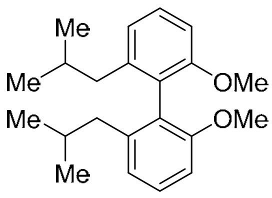

rac-**S15**
$C_{22}H_{30}O_2$
M = 326.48 g/mol

In a round bottom flask, *rac*-**S13** (251 mg, 0.778 mmol, 1.00 equiv) and platinum(IV) dioxide (17.7 mg, 78 µmol, 10 mol %) were suspended in glacial acetic acid (8 mL). The mixture was purged with hydrogen for 10 min and the flask was equipped with an hydrogen balloon. After stirring at room temperature for 22 h, the mixture was filtered over Celite®. The filtrate was diluted with saturated aqueous Na_2CO_3 solution (20 mL) and *tert*-butyl methyl ether (20 mL).

The phases were separated, and the organic phase was washed with saturated aqueous Na_2CO_3 solution (3 x 20 mL) and 2 M NaOH (20 mL), dried over Na_2SO_4, and filtered. After evaporation of the solvent, the title compound *rac*-**S15** (244 mg, 0.747 mmol, 96%) was obtained as a pale brown oil.

^{1}H NMR (500 MHz, $CDCl_3$): δ/ppm = 7.26 (t, *J* = 7.9 Hz, 2H), 6.88 (d, *J* = 7.7 Hz, 2H), 6.79 (d, *J* = 8.2 Hz, 2H), 3.66 (s, 6H), 2.08 (m_c, 4H), 1.69 (non, *J* = 6.6 Hz, 2H), 0.77 (d, *J* = 6.6 Hz, 6H), 0.68 (d, *J* = 6.6 Hz, 6H). **^{13}C{^{1}H} NMR** (126 MHz, $CDCl_3$): δ/ppm = 157.4 (2C), 141.7 (2C), 127.7 (2C), 126.4 (2C), 121.8 (2C), 108.1 (2C), 55.6 (2C), 42.6 (2C), 28.3 (2C), 22.8 (2C), 22.8 (2C). **HRMS** (APCI) calculated for $C_{22}H_{31}O_2^+$ [$(M+H)^+$]: 327.2319; found: 327.2314.

***rac*-6,6'-Diisobutyl-[1,1'-biphenyl]-2,2'-diol (*rac*-S17)**

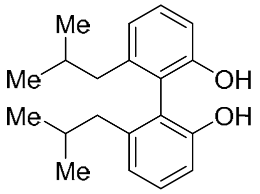

rac-**S17**
$C_{20}H_{26}O_2$
M = 298.43 g/mol

Prepared according to **GP3** from *rac*-**S15** (230 mg, 0.704 mmol, 1.00 equiv) and boron tribromide (1.4 mL, 1.4 mmol, 2.0 equiv, 1.0 M in dichloromethane) in dry dichloromethane (1.4 mL). After stirring for 16 h, the reaction was quenched by addition of water (1.5 mL), the phases were separated, and the aqueous phase was extracted with dichloromethane (4 x 3 mL). The combined organic layers were dried over Na_2SO_4, filtered, and the solvent was removed under reduced pressure. Filtration over a plug of silica using *tert*-butyl methyl ether afforded the diol *rac*-**S17** (210 mg, 0.704 mmol, quant.) as a colorless solid.

M.p. = 63–68 °C (dichloromethane/*n*-pentane). **R_f** = 0.29 (cyclohexane/ethyl acetate = 5:1). **^{1}H NMR** (500 MHz, $CDCl_3$): δ/ppm = 7.30 (t, *J* = 7.9 Hz, 2H), 6.93 (d, *J* = 7.8 Hz, 2H), 6.90 (d, *J* = 8.4 Hz, 2H), 4.63 (s, br, 2H), 2.18 (dd, *J* = 14.0, 7.5 Hz, 2H), 2.12 (dd, *J* = 14.0, 7.3 Hz, 2H), 1.71 (non, *J* = 6.7 Hz, 2H), 0.80 (d, *J* = 6.6 Hz, 6H), 0.73 (d, *J* = 6.6 Hz, 6H). **^{13}C{^{1}H} NMR** (126 MHz, $CDCl_3$): δ/ppm = 154.0 (2C), 142.8 (2C), 130.1 (2C), 122.5 (2C), 119.5 (2C), 113.3 (2C), 42.6 (2C), 28.3 (2C), 22.8 (2C), 22.6 (2C). **HRMS** (APCI) calculated for $C_{20}H_{27}O_2^+$ [$(M+H)^+$]: 299.2006; found: 299.2000. **IR** (ATR): $\tilde{\nu}$/cm^{-1} = 3479, 3064, 2953, 1573, 1456, 1327, 1277, 1176, 1105, 991, 937, 752.

rac-2',6-Diisobutyl-6'-isopropoxy-[1,1'-biphenyl]-2-ol (rac-1y)

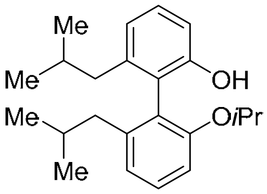

rac-**1y**
$C_{23}H_{32}O_2$
M = 340.51 g/mol

Prepared according to **GP1** from racemic diol **S17** (223 mg, 0.747 mmol, 1.00 equiv), potassium carbonate (140 mg, 1.01 mmol, 1.35 equiv) and 2-iodopropane (89 µL, 0.90 mmol, 1.2 equiv) in acetone (7.5 mL). The reaction mixture was refluxed for 20 h, cooled to room temperature and loaded on Celite®. Flash column chromatography on silica gel using cyclohexane/dichloromethane (2:1 → 1:1) afforded the title compound *rac*-**1y** (125 mg, 0.367 mmol, 49%) as a yellow oil.

The analytical data are consistent with those reported in section 4 for the enantioenriched alcohol.

***rac*-1,14-Dimethoxy-5,6,7,8,9,10-hexahydrodibenzo[*a*,*c*][10]annulene (*rac*-S19)**

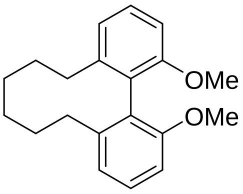

rac-**S19**
$C_{20}H_{24}O_2$
M = 296.41 g/mol

In a round bottom flask, *rac*-**S18** (403 mg, 1.38 mmol, 1.00 equiv) and platinum(IV) dioxide (35 mg, 0.15 mmol, 11 mol %) were suspended in glacial acetic acid (16 mL). The mixture was purged with hydrogen for 10 min and the flask was equipped with a hydrogen balloon. After stirring at room temperature for 20 h, the mixture was filtered over Silica®. The filtrate was diluted with 2 M NaOH (30 mL) and *tert*-butyl methyl ether (30 mL). The phases were separated and the organic phase was washed with 2 M NaOH (2 x 50 mL), dried over Na_2SO_4, and filtered. After evaporation of the solvent, the title compound *rac*-**S19** (319 mg, 1.07 mmol, 78%) was obtained as a pale yellow solid.

^{1}H NMR (400 MHz, $CDCl_3$): δ/ppm = 7.30 (t, *J* = 7.9 Hz, 2H), 6.93 (d, *J* = 7.8 Hz, 2H), 6.79 (d, *J* = 8.0 Hz, 2H), 3.66 (s, 6H), 2.53–2.46 (m, 2H), 2.33 (dt, *J* = 13.4, 4.3 Hz, 2H), 1.74–1.63 (m, 2H), 1.43–1.33 (m, 2H), 1.21–1.15 (m, 2H), 0.75–0.64 (m, 2H). **$^{13}C\{^1H\}$ NMR** (101 MHz, $CDCl_3$): δ/ppm = 157.1 (2C), 143.0 (2C), 128.3 (2C), 127.4 (2C), 120.8 (2C), 108.0 (2C), 56.0 (2C), 29.0 (2C), 28.4 (2C), 21.3 (2C).

The NMR spectroscopic data are consistent with those reported in the literature.[17]

***rac*-5,6,7,8,9,10-Hexahydrodibenzo[*a*,*c*][10]annulene-1,14-diol (*rac*-S20)**

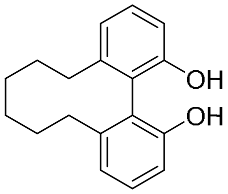

rac-**S20**
$C_{18}H_{20}O_2$
M = 268.36 g/mol

Prepared according to **GP3** from *rac*-**S16** (278 mg, 0.938 mmol, 1.00 equiv) and boron tribromide (1.9 mL, 1.89 mmol, 2.0 equiv, 1.0 M in dichloromethane) in dry dichloromethane (1.9 mL). After stirring for 16 h, the reaction was quenched by addition of water (4 mL), the phases were separated, and the aqueous phase was extracted with dichloromethane (4 x 4 mL). The combined organic layers were dried over Na_2SO_4, filtered, and the solvent was removed under reduced pressure. Filtration over a plug of silica using *tert*-butyl methyl ether afforded the diol *rac*-**S17** (252 mg, 0.938 mmol, quant.) as a brown solid.

^{1}H NMR (400 MHz, $CDCl_3$): δ/ppm = 7.33 (t, *J* = 7.9 Hz, 2H), 6.95 (d, *J* = 7.7 Hz, 2H), 6.90 (d, *J* = 8.0 Hz, 2H), 4.59 (s, br, 2H), 2.62–2.55 (m, 2H), 2.36 (dt, *J* = 13.3, 4.4 Hz, 2H), 1.74–1.63 (m, 2H), 1.49–1.38 (m, 2H), 1.25–1.14 (m, 2H), 0.71–0.61 (m, 2H). **$^{13}C\{^1H\}$ NMR** (101 MHz, $CDCl_3$): δ/ppm = 153.9 (2C), 143.6 (2C), 130.7 (2C), 121.5 (2C), 120.7 (2C), 113.0 (2C), 29.3 (2C), 28.4 (2C), 20.9 (2C).

The NMR spectroscopic data are consistent with those reported in the literature.[17]

rac-14-Isopropoxy-5,6,7,8,9,10-hexahydrodibenzo[_a_,_c_][10]annulen-1-ol (_rac_-1b')

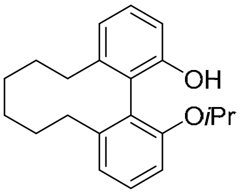

rac-**1b'**
$C_{21}H_{26}O_2$
M = 310.44 g/mol

Prepared according to **GP1** from *rac*-**S20** (249 mg, 0.928 mmol, 1.00 equiv), potassium carbonate (173 mg, 1.25 mmol, 1.35 equiv) and 2-iodopropane (0.10 mL, 1.0 mmol, 1.1 equiv) in acetone (9.3 mL). The reaction mixture was refluxed for 16 h, cooled to room temperature and the solvent was removed under reduced pressure. Flash column chromatography on silica gel using cyclohexane/dichloromethane (3:1 → 2:1 → 1:1) afforded *rac*-**1b'** (76.6 mg, 0.247 mmol, 27%) together with some unknown impurities. Preparative TLC (cyclohexane/ethyl acetate = 20:1) afforded the title compound *rac*-**1b'** (52.7 mg, 0.170 mmol, 18%) in an analytically pure form as a colorless solid.

The analytical data are consistent with those reported in section 4 for the enantioenriched alcohol.

4 Experimental Details for the Kinetic Resolutions

(*S*)-[(2'-Methoxy-[1,1'-binaphthalen]-2-yl)oxy](methyl)diphenylsilane [(*S*)-3ba] and (*R*)-2'-Methoxy-[1,1'-binaphthalen]-2-ol [(*R*)-1b]

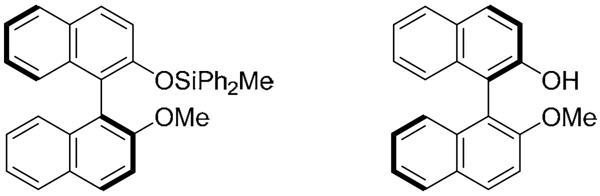

(*S*)-**3ba** $C_{34}H_{28}O_2Si$ M = 496.68 g/mol

(*R*)-**1b** $C_{21}H_{16}O_2$ M = 300.36 g/mol

Prepared according to **GP4** from *rac*-**1b** (60.1 mg, 0.200 mmol, 1.00 equiv) and methyldiphenylsilane (**2a**, 27.8 mg, 0.140 mmol, 0.700 equiv). 4 Å molecular sieves were added to the catalyst mixture, and the reaction mixture was stirred for 70 h at room temperature. Flash column chromatography on silica gel using cyclohexane/*tert*-butyl methyl ether (40:1 → 15:1 → 10:1 → 5:1) afforded silyl ether (*S*)-**3ba** (58.6 mg, 0.118 mmol, 59%, 45% *ee*) and alcohol (*R*)-**1b** (18.7 mg, 62.3 µmol, 31%, 67% *ee*) as colorless solids.

Analytical data for silyl ether (*S*)-**3ba**:

M.p. = 56–58 °C (dichloromethane/*n*-pentane). $\mathbf{R_f}$ = 0.36 (cyclohexane/*tert*-butyl methyl ether = 50:1). **^{1}H NMR** (500 MHz, CD_2Cl_2): δ/ppm = 8.07 (d, *J* = 9.1 Hz, 1H), 7.96 (d, *J* = 8.2 Hz, 1H), 7.87 (d, *J* = 8.2 Hz, 1H), 7.80 (d, *J* = 8.8 Hz, 1H), 7.46 (d, *J* = 9.1 Hz, 1H), 7.41–7.32 (m, 4H), 7.30–7.15 (m, 13H), 3.71 (s, 3H), 0.58 (s, 3H). **^{13}C{^{1}H} NMR** (126 MHz, CD_2Cl_2): δ/ppm = 155.5, 151.2, 136.3, 136.0, 134.8, 134.5, 134.4 (4C), 130.2, 130.2, 129.9, 129.9, 129.7, 129.3, 128.4 (2C), 128.1 (4C), 126.8, 126.6, 125.8, 125.7, 124.2, 123.9, 122.4, 121.2, 119.7, 114.1, 56.6, –2.5. **^{1}H/^{29}Si HMQC NMR** (500/99 MHz, CD_2Cl_2, optimized for *J* = 7 Hz): δ/ppm = (7.20, 0.58)/–4.5. **HRMS** (APCI) calculated for $C_{34}H_{28}O_2Si^+$ [M^+]: 496.1853; found: 496.1846. **IR** (ATR): ṽ/cm^{-1} = 3048, 2929, 2835, 2503, 1474, 1332, 1240, 1116, 996, 951, 808, 726, 698. **Optical Rotation:** $[\alpha]_D^{20}$ = –21.7 (*c* 2.3, dichloromethane). The enantiomeric excess of **3ba** was determined by HPLC analysis on a chiral stationary phase after cleavage of the silyl ether according to **GP5** (Daicel Chiralpak® IA column, column temperature: 20 °C, *n*-heptane/isopropanol = 90:10, flow rate: 0.60 mL/min, λ = 230 nm): t_R = 19.2 min (major), t_R = 29.7 min (minor).

Analytical data for alcohol (*R*)-**1b**:

M.p. = 139–140 °C ($CHCl_3$/*n*-pentane). $\mathbf{R_f}$ = 0.14 (cyclohexane/*tert*-butyl methyl ether = 7:1). **^{1}H NMR** (500 MHz, $CDCl_3$): δ/ppm = 8.06 (d, *J* = 9.1 Hz, 1H), 7.91 (dd, *J* = 8.6, 3.2 Hz, 2H), 7.87 (d, *J* = 8.1 Hz, 1H), 7.49 (d, *J* = 9.1 Hz, 1H), 7.31 (m_c, 2H), 7.34–7.27 (m, 2H), 7.25–7.17 (m, 2H), 7.06 (d, *J* = 8.4 Hz, 1H), 4.93 (s, br, 1H), 3.81 (s, 3H). **^{13}C{^{1}H} NMR** (126 MHz, $CDCl_3$): δ/ppm = 156.2, 151.4, 134.2, 133.9, 131.2, 129.9, 129.6, 129.3, 128.3, 128.3, 127.5, 126.5, 125.0, 125.0, 124.3, 123.4, 117.6, 115.5, 115.2, 114.0, 56.8. **HRMS** (APCI) calculated for $C_{21}H_{17}O_2^+$ $[(M+H)^+]$: 301.1223; found: 301.1220. **IR** (ATR): ṽ/cm^{-1} = 3499, 3056, 2932, 2839, 1592, 1506, 1461, 1379, 1263, 1206, 1146, 1082, 972, 906, 812, 749. **Optical Rotation:** $[\alpha]_D^{20}$ = +32.2 (*c* 0.75, acetone). The enantiomeric excess of **1b** was determined by HPLC analysis on a chiral stationary phase (Daicel Chiralpak® IA column, column temperature: 20 °C, *n*-heptane/isopropanol = 90:10, flow rate: 0.60 mL/min, λ = 230 nm): t_R = 19.2 min for (minor), t_R = 29.6 min (major).

(*S*)-[(2'-Isopropoxy-[1,1'-binaphthalen]-2-yl)oxy](methyl)diphenylsilane [(*S*)-3ca] and (*R*)-2'-Isopropoxy-[1,1'-binaphthalen]-2-ol [(*R*)-1c]

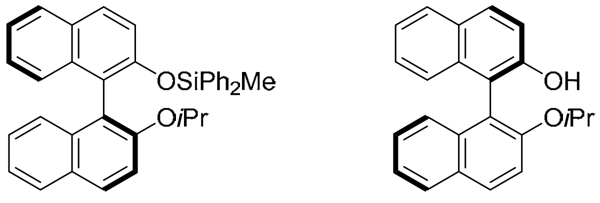

(*S*)-**3ca** $C_{36}H_{32}O_2Si$ M = 524.74 g/mol

(*R*)-**1c** $C_{23}H_{20}O_2$ M = 328.41 g/mol

Prepared according to **GP4** from *rac*-**1c** (65.7 mg, 0.200 mmol, 1.00 equiv) and methyldiphenylsilane (**2a**, 27.8 mg, 0.140 mmol, 0.700 equiv). The reaction mixture was stirred for 48 h at room temperature. Flash column chromatography on silica gel using cyclohexane/*tert*-butyl methyl ether (100:0 → 70:1 → 50:1 → 20:1) afforded silyl ether (*S*)-**3ca** (40.6 mg, 77.4 µmol, 39%, 82% *ee*) and alcohol (*R*)-**1c** (36.9 mg, 0.112 mmol, 56%, 55% *ee*) as yellow solids.

Analytical data for silyl ether (*S*)-**3ca**:

M.p. = 51–53 °C (dichloromethane/*n*-pentane). $\mathbf{R_f}$ = 0.60 (cyclohexane/*tert*-butyl methyl ether = 50:1). **^{1}H NMR** (500 MHz, CD_2Cl_2): δ/ppm = 8.01 (d, *J* = 9.0 Hz, 1H), 7.93 (d, *J* = 8.2 Hz, 1H), 7.85 (d, *J* = 8.2 Hz, 1H), 7.77 (d, *J* = 8.9 Hz, 1H), 7.45 (d, *J* = 9.1 Hz, 1H), 7.37–7.29 (m,

4H), 7.27–7.17 (m, 9H), 7.17–7.11 (m, 4H), 4.51 (sept, *J* = 6.1 Hz, 1H), 1.05 (d, *J* = 6.1 Hz, 3H), 1.02 (d, *J* = 6.0 Hz, 3H), 0.56 (s, 3H). **^{13}C{^{1}H} NMR** (126 MHz, CD_2Cl_2): δ/ppm = 154.5, 151.1, 136.3, 136.1, 134.8, 134.8, 134.5 (2C), 134.4 (2C), 130.2, 130.1, 129.9, 129.8, 129.5, 129.1, 128.3, 128.2, 128.1 (2C), 128.1 (2C), 126.6, 126.4, 126.1, 125.8, 124.0, 124.0, 122.8, 121.8, 120.9, 117.8, 72.3, 22.7, 22.6, –2.5. **^{1}H/^{29}Si HMQC NMR** (500/99 MHz, CD_2Cl_2, optimized for *J* = 7 Hz): δ/ppm = (7.24, 7.21, 0.56)/–4.7. **HRMS** (APCI) calculated for $C_{36}H_{32}O_2Si^+$ [M^+]: 524.2166; found: 524.2164. **IR** (ATR): ṽ/cm^{-1} = 3050, 2974, 2925, 1591, 1504, 1472, 1353, 1241, 1113, 997, 809, 727. **Optical Rotation:** $[\alpha]_D^{20}$ = –44.0 (*c* 1.5, dichloromethane). The enantiomeric excess of **3ca** was determined by HPLC analysis on a chiral stationary phase after cleavage of the silyl ether according to **GP5** (Daicel Chiralpak® IA column, column temperature: 20 °C, *n*-heptane/isopropanol = 90:10, flow rate: 0.60 mL/min, λ = 254 nm): t_R = 10.1 min (major), t_R = 30.5 min (minor).

Analytical data for alcohol (*R*)-**1c**:

M.p. = 116–117 °C (dichloromethane/*n*-pentane). **R_f** = 0.20 (cyclohexane/*tert*-butyl methyl ether = 50:1). **^{1}H NMR** (500 MHz, $CDCl_3$): δ/ppm = 8.00 (d, *J* = 9.0 Hz, 1H), 7.92–7.85 (m, 3H), 7.45 (d, *J* = 9.1 Hz, 1H), 7.40–7.35 (m, 2H), 7.34–7.25 (m, 2H), 7.25–7.17 (m, 2H), 7.08 (d, *J* = 8.5 Hz, 1H), 5.08 (s, br, 1H), 4.45 (sept, *J* = 6.2 Hz, 1H), 1.14 (d, *J* = 6.1 Hz, 3H), 1.00 (d, *J* = 6.0 Hz, 3H). **^{13}C{^{1}H} NMR** (126 MHz, $CDCl_3$): δ/ppm = 154.8, 151.4, 134.4, 134.0, 130.8, 129.9, 129.7, 129.2, 128.2, 128.1, 127.2, 126.3, 125.4, 125.3, 124.5, 123.2, 118.3, 118.1, 117.7, 115.7, 72.7, 22.4, 22.4. **HRMS** (APCI) calculated for $C_{23}H_{21}O_2^+$ [$(M+H)^+$]: 329.1536; found: 329.1534. **IR** (ATR): ṽ/cm^{-1} = 3503, 3423, 3055, 2984, 2924, 1591, 1505, 1462, 1378, 1325, 1240, 1109, 1000. 906, 811, 749, 683. **Optical Rotation:** $[\alpha]_D^{20}$ = –40.9 (*c* 1.6, $CHCl_3$). The enantiomeric excess of **1c** was determined by HPLC analysis on a chiral stationary phase (Daicel Chiralpak ® IA column, column temperature: 20 °C, *n*-heptane/isopropanol = 90:10, flow rate: 0.60 mL/min, λ = 254 nm): t_R = 10.1 min (minor), t_R = 30.9 min (major).

(*S*)-[(2'-(Benzyloxy)-[1,1'-binaphthalen]-2-yl)oxy](methyl)diphenylsilane [(*S*)-3da] and (*R*)-2'-(Benzyloxy)-[1,1'-binaphthalen]-2-ol [(*R*)-1d]

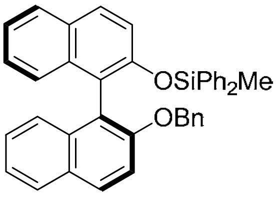

(*S*)-**3da**
$C_{40}H_{32}O_2Si$
M = 572.78 g/mol

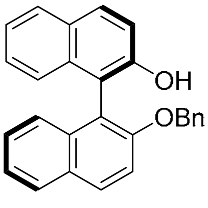

(*R*)-**1d**
$C_{27}H_{20}O_2$
M = 376.46 g/mol

Prepared according to **GP4** from *rac*-**1d** (75.3 mg, 0.200 mmol, 1.00 equiv) and methyldiphenylsilane (27.8 mg, 0.140 mmol, 0.700 equiv). The reaction mixture was stirred for 45 h at room temperature. Flash column chromatography on silica gel using cyclohexane/*tert*-butyl methyl ether (100:1 → 70:1 → 50:1 → 30:1 → 20:1) afforded silyl ether (*S*)-**3da** (51.7 mg, 90.3 µmol, 45%, 49% *ee*) as a colorless solid and alcohol (*R*)-**1d** (35.4 mg, 94.0 µmol, 47%, 43% *ee*) as a pale brown solid.

Analytical data for silyl ether (*S*)-**3da**:

M.p. = 58–60 °C (dichloromethane/*n*-pentane). $\mathbf{R_f}$ = 0.43 (cyclohexane/*tert*-butyl methyl ether = 50:1). **^{1}H NMR** (500 MHz, CD_2Cl_2): δ/ppm = 8.01 (d, *J* = 9.0 Hz, 1H), 7.95 (d, *J* = 8.2 Hz, 1H), 7.88 (d, *J* = 8.1 Hz, 1H), 7.82 (d, *J* = 8.9 Hz, 1H), 7.45 (d, *J* = 9.1 Hz, 1H), 7.41–7.30 (m, 4H), 7.29–7.25 (m, 3H), 7.25–7.08 (m, 13H), 7.04 (d, *J* = 7.1 Hz, 2H), 5.06 (s, 2H), 0.53 (s, 3H). **^{13}C{^{1}H} NMR** (126 MHz, CD_2Cl_2): δ/ppm = 154.8, 151.2, 138.0, 136.1, 136.0, 134.9, 134.6, 134.5 (2C), 134.4 (2C), 130.2, 130.2, 130.0, 130.0, 129.7, 129.4, 128.6 (2C), 128.4 (2C), 128.1 (2C), 128.1 (2C), 127.8, 127.2 (2C), 126.8, 126.7, 125.9, 125.9, 124.2 (2C), 122.5, 121.1, 121.0, 116.1, 71.3, –2.4. **^{1}H/^{29}Si HMQC NMR** (500/99 MHz, CD_2Cl_2, optimized for *J* = 7 Hz): δ/ppm = (7.23, 7.12, 0.53)/–4.3. **HRMS** (APCI) calculated for $C_{40}H_{32}O_2Si^+$ [M^+]: 572.2166; found: 572.2162. **IR** (ATR): $\tilde{v}$/cm^{-1} = 3049, 2918, 1589, 1503, 1458, 1353, 1240, 1114, 996, 951, 806, 727, 696. **Optical Rotation:** $[\alpha]_D^{20}$ = –19.3 (*c* 2.1, dichloromethane). The enantiomeric excess of **3da** was determined by HPLC analysis on a chiral stationary phase after cleavage of the silyl ether according to **GP5** (Daicel Chiralpak® AD-H column, column temperature: 20 °C, *n*-heptane/isopropanol = 80:20, flow rate: 0.80 mL/min, λ = 230 nm): t_R = 24.4 min (major), t_R = 43.1 min (minor).

Analytical data for alcohol (*R*)-**1d**:

M.p. = 57–58 °C ($CHCl_3$/*n*-pentane). $\mathbf{R_f}$ = 0.16 (cyclohexane/*tert*-butyl methyl ether = 30:1). **^{1}H NMR** (500 MHz, $CDCl_3$): δ/ppm = 7.99 (d, *J* = 9.1 Hz, 1H), 7.94 (d, *J* = 8.8 Hz, 1H), 7.89 (d, *J* = 8.2 Hz, 2H), 7.47 (d, *J* = 9.1 Hz, 1H), 7.41–7.36 (m, 2H), 7.35–7.28 (m, 2H), 7.27–7.22 (m, 2H), 7.21–7.17 (m, 3H), 7.10 (d, *J* = 8.4 Hz, 1H), 7.07–7.03 (m, 2H), 5.10 (m_c, 2H), 4.94 (s, 1H). **^{13}C{^{1}H} NMR** (126 MHz, $CDCl_3$): δ/ppm = 155.1, 151.5, 137.1, 134.2, 134.0, 131.0, 130.0, 129.9, 129.3, 128.5 (2C), 128.3, 128.2, 127.8, 127.4, 127.0 (2C), 126.5, 125.2, 125.1, 124.6, 123.4, 117.7, 117.0, 116.1, 115.3, 71.3. **HRMS** (APCI) calculated for $C_{27}H_{21}O_2^+$ [(M+H)$^+$]: 377.1536; found: 377.1535. **IR** (ATR): $\tilde{\nu}$/cm^{-1} = 3056, 2921, 2851, 1590, 1505, 1455, 1379, 1328, 1263, 1215, 1144, 1083, 1047, 1017, 906, 809, 730. **Optical Rotation:** $[\alpha]_D^{20}$ = –2.6 (*c* 1.2, $CHCl_3$). The enantiomeric excess of **1d** was determined by HPLC analysis on a chiral stationary phase (Daicel Chiralpak® AD-H column, column temperature: 20 °C, *n*-heptane/isopropanol = 80:20, flow rate: 0.80 mL/min, λ = 230 nm): t_R = 24.4 min (minor), t_R = 43.1 min (major).

(*S*)-*tert*-Butyldimethyl[(2'-[(methyldiphenylsilyl)oxy]-[1,1'-binaphthalen]-2-yl)oxy]silane [(*S*)-3fa] and (*R*)-2'-[(*tert*-Butyldimethylsilyl)oxy]-[1,1'-binaphthalen]-2-ol [(*R*)-1f]

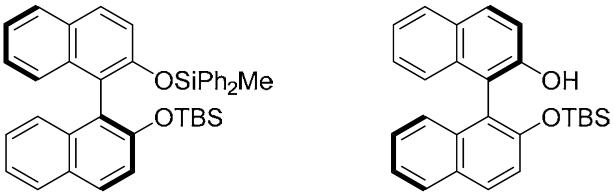

(*S*)-**3fa** $C_{39}H_{40}O_2Si_2$ M = 596.92 g/mol

(*R*)-**1f** $C_{26}H_{28}O_2Si$ M = 400.59 g/mol

The kinetic resolution was performed according to **GP4** using *rac*-**1e** (80.1 mg, 0.200 mmol, 1.00 equiv) and methyldiphenylsilane (**2a**, 27.8 mg, 0.140 mmol, 0.700 equiv). 4 Å molecular sieves were added to the catalyst mixture, and the reaction mixture was stirred for 54 h at room temperature. The reaction was quenched by filtration over silica, and the HPLC analysis was carried out of the crude mixture, affording an enantiomeric excess of only 24% for the unreacted alcohol. Thus, no isolation of the pure silyl ether and the alcohol was carried out.

(*S*)-[(2'-Isopropoxy-5,5',6,6',7,7',8,8'-octahydro-[1,1'-binaphthalen]-2-yl)oxy](methyl)diphenylsilane [(*S*)-3ka] and (*R*)-2'-Isopropoxy-5,5',6,6',7,7',8,8'-octahydro-[1,1'-binaphthalen]-2-ol [(*R*)-1k]

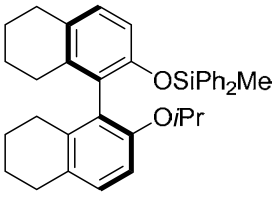

(*S*)-**3ka**
$C_{36}H_{40}O_2Si$
M = 532.80 g/mol

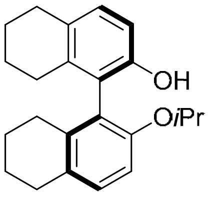

(*R*)-**1k**
$C_{23}H_{28}O_2$
M = 336.48 g/mol

Prepared according to **GP4** from *rac*-**1k** (67.3 mg, 0.200 mmol, 1.00 equiv) and methyldiphenylsilane (**2a**, 27.8 mg, 0.140 mmol, 0.700 equiv). 4 Å molecular sieves were added to the catalyst mixture, and the reaction mixture was stirred for 18 h at room temperature. Flash column chromatography on silica gel using cyclohexane/*tert*-butyl methyl ether (70:1 → 60:1 → 50:1 → 20:1) afforded silyl ether (*S*)-**3ka** (65.1 mg, 0.122 mmol, 61%, 50% *ee*) as a colorless sticky resin and alcohol (*R*)-**1k** (22.2 mg, 66.0 µmol, 33%, >99% *ee*) as a brown resin.

Analytical data for silyl ether (*S*)-**3ka**:

R_f = 0.72 (cyclohexane/*tert*-butyl methyl ether = 50:1). **^{1}H NMR** (500 MHz, CD_2Cl_2): δ/ppm = 7.40–7.32 (m, 4H), 7.32–7.23 (m, 6H), 7.10 (d, *J* = 8.4 Hz, 1H), 6.85 (d, *J* = 8.2 Hz, 1H), 6.80 (d, *J* = 8.4 Hz, 1H), 6.55 (d, *J* = 8.2 Hz, 1H), 4.33 (sept, *J* = 6.0 Hz, 1H), 2.82–2.71 (m, 4H), 2.41 (m_c, 2H), 2.21–2.09 (m, 2H), 1.79–1.57 (m, 8H), 1.15 (d, *J* = 6.1 Hz, 3H), 1.04 (d, *J* = 6.0 Hz, 3H), 0.64 (s, 3H). **^{13}C{^{1}H} NMR** (126 MHz, CD_2Cl_2): δ/ppm = 153.7, 150.1, 137.7, 137.5, 137.0, 136.7, 134.6 (2C), 134.5 (2C), 130.5, 130.2, 130.2, 130.0, 129.3, 128.8, 128.5, 128.2 (2C), 128.1, 128.1 (2C), 116.0, 112.6, 70.7, 29.9, 29.8, 27.8, 27.8, 23.8, 23.7, 23.7, 23.7, 22.5, 22.4, –2.5. **^{1}H/^{29}Si HMQC NMR** (500/99 MHz, CD_2Cl_2, optimized for *J* = 7 Hz): δ/ppm = (7.38, 7.27, 0.64)/–6.6. **HRMS** (APCI) calculated for $C_{36}H_{40}O_2Si^+$ [M$^+$]: 532.2792; found: 532.2787. **IR** (ATR): ṽ/cm^{-1} = 3046, 2921, 1588, 1467, 1251, 1112, 1072, 973, 791, 724. **Optical Rotation:** $[\alpha]_D^{20}$ = –31.4 (*c* 1.8, dichloromethane).The enantiomeric excess of (*R*)-**3ka** was determined by HPLC analysis on a chiral stationary phase after cleavage of the silyl ether according to **GP5** (Daicel Chiralpak® AD-H column, column temperature: 20 °C, *n*-heptane/isopropanol = 90:10, flow rate: 0.60 mL/min, λ = 273 nm): t_R = 6.5 min (major), t_R = 18.0 min (minor).

Analytical data for alcohol (*R*)-**1k**:

R$_f$ = 0.22 (cyclohexane/*tert*-butyl methyl ether = 50:1). **^{1}H NMR** (500 MHz, $CDCl_3$): δ/ppm = 7.08 (d, *J* = 8.4 Hz, 1H), 6.98 (d, *J* = 8.3 Hz, 1H), 6.81 (d, *J* = 8.4 Hz, 1H), 6.75 (d, *J* = 8.2 Hz, 1H), 4.41 (s, br, 1H), 4.27 (sept, *J* = 6.1 Hz, 1H), 2.36–2.27 (m, 2H), 2.21–2.07 (m, 2H), 2.76 (m$_c$, 4H), 1.76–1.70 (m, 4H), 1.69–1.61 (m, 4H), 1.13 (d, *J* = 6.1 Hz, 3H), 1.08 (d, *J* = 6.1 Hz, 3H). **^{13}C{^{1}H} NMR** (126 MHz, $CDCl_3$): δ/ppm = 154.2, 150.2, 138.3, 136.3, 130.8, 130.2, 129.1, 129.0, 124.3, 123.3, 113.4, 112.0, 71.1, 29.5 (2C), 27.4, 27.3, 23.4 (2C), 23.3, 23.2, 22.3, 22.2. **HRMS** (APCI) calculated for $C_{23}H_{29}O_2^+$ [(M+H)$^+$]: 337.2162; found: 337.2160. **IR** (ATR): ṽ/cm^{-1} = 3537, 2915, 2855, 1591, 1473, 1260, 1188, 1113, 981, 805. **Optical Rotation:** $[\alpha]_D^{20}$ = +40.5 (*c* 0.67, $CHCl_3$). The enantiomeric excess of (*S*)-**1k** was determined by HPLC analysis on a chiral stationary phase (Daicel Chiralpak® AD-H column, column temperature: 20 °C, *n*-heptane/isopropanol = 90:10, flow rate: 0.60 mL/min, λ = 273 nm): t_R = 6.5 min (minor), t_R = 18.1 min (major).

(*S*)-[(2'-Isopropoxy-4,4'-dimethyl-[1,1'-binaphthalen]-2-yl)oxy](methyl)diphenylsilane [(*S*)-3la] and (*R*)-2'-Isopropoxy-4,4'-dimethyl-[1,1'-binaphthalen]-2-ol [(*R*)-1l]

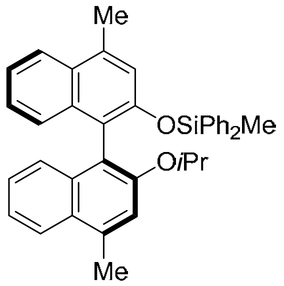

(*S*)-**3la**
$C_{38}H_{36}O_2Si$
M = 552.79 g/mol

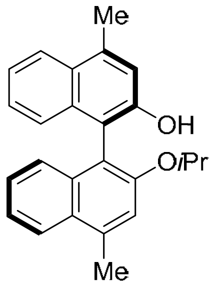

(*R*)-**1l**
$C_{25}H_{24}O_2$
M = 356.47 g/mol

Prepared according to **GP4** from *rac*-**1l** (71.3 mg, 0.200 mmol, 1.00 equiv) and methyldiphenylsilane (**2a**, 27.8 mg, 0.140 mmol, 0.700 equiv). 4 Å molecular sieves were added to the catalyst mixture, and the reaction mixture was stirred for 103 h at room temperature. Flash column chromatography on silica gel using cyclohexane/*tert*-butyl methyl ether (100:0 → 80:1 → 70:1 → 30:1) afforded silyl ether (*S*)-**3la** (40.9 mg, 74.0 µmol, 37%, 76% *ee*) as a sticky yellow resin and alcohol (*R*)-**1l** (39.2 mg, 0.110 mmol, 55%, 53% *ee*) as a yellow solid.

Analytical data for silyl ether (*S*)-**3la**:

R$_f$ = 0.20 (cyclohexane/ethyl acetate = 80:1). **^{1}H NMR** (500 MHz, CD_2Cl_2): δ/ppm = 8.06 (d, *J* = 8.5 Hz, 1H), 8.00 (d, *J* = 8.3 Hz, 1H), 7.40–7.11 (m, 17H), 7.04 (s, 1H), 2.86 (s, 3H), 2.67 (s, 3H), 4.50 (sept, *J* = 6.1 Hz, 1H), 1.06 (d, *J* = 6.1 Hz, 3H), 1.03 (d, *J* = 6.1 Hz, 3H), 0.57 (s, 3H). **^{13}C{^{1}H} NMR** (126 MHz, CD_2Cl_2): δ/ppm = 154.1, 150.7, 136.5, 136.3, 136.1, 135.8, 135.2, 135.1, 134.5 (2C), 134.4 (2C), 130.1, 130.0, 129.2, 128.7, 128.0 (2C), 128.0 (2C), 126.7, 126.5, 126.3, 126.1, 124.5, 124.5, 123.8, 123.7, 121.9, 121.1, 120.0, 118.8, 72.1, 22.8, 22.7, 20.1, 19.7, –2.5. **^{1}H/^{29}Si HMQC NMR** (500/99 MHz, CD_2Cl_2, optimized for *J* = 7 Hz): δ/ppm = (7.22, 7.13, 0.57)/–5.0. **HRMS** (APCI) calculated for $C_{38}H_{36}O_2Si^+$ [M$^+$]: 552.2479; found: 552.2477. **IR** (ATR): ṽ/cm^{-1} = 3064, 2972, 1592, 1508, 1462, 1348, 1237, 1203, 1109, 1006, 830, 735, 720. **Optical Rotation:** $[\alpha]_D^{20}$ = –36.6 (*c* 1.7, dichloromethane). The enantiomeric excess of **3la** was determined by HPLC analysis on a chiral stationary phase after cleavage of the silyl ether according to **GP5** (Daicel Chiralpak® IA column, column temperature: 20 °C, *n*-heptane/isopropanol = 90:10, flow rate: 0.60 mL/min, λ = 230 nm): t_R = 9.3 min (major), t_R = 40.4 min (minor).

Analytical data for alcohol (*R*)-**1l**:

M.p. = 43–46 °C (dichloromethane/*n*-pentane). **R$_f$** = 0.18 (cyclohexane/ethyl acetate = 50:1). **^{1}H NMR** (500 MHz, $CDCl_3$): δ/ppm = 8.02 (d, *J* = 8.6 Hz, 1H), 8.00 (d, *J* = 8.5 Hz, 1H), 7.39 (t, *J* = 7.6 Hz, 1H), 7.33 (t, *J* = 7.8 Hz, 1H), 7.30 (s, 1H), 7.23–7.25 (m, 1H), 7.21 (s, 2H), 7.18–7.24 (m, 1H), 7.10 (d, *J* = 8.4 Hz, 1H), 5.02 (s, 1H), 4.42 (sept, *J* = 6.1 Hz, 1H), 2.81 (s, 3H), 2.78 (s, 3H), 1.12 (d, *J* = 6.1 Hz, 3H), 0.98 (d, *J* = 6.1 Hz, 3H). **^{13}C{^{1}H} NMR** (126 MHz, $CDCl_3$): δ/ppm = 154.4, 151.0, 137.4, 136.3, 134.7, 134.4, 129.3, 128.5, 126.8, 126.2, 126.0 (2C), 124.4, 124.3 (2C), 122.9, 119.3, 118.5, 116.6, 113.9, 72.7, 22.5 (2C), 20.0, 19.7. **HRMS** (APCI) calculated for $C_{25}H_{25}O_2^+$ [(M+H)$^+$]: 357.1849; found: 357.1849. **IR** (ATR): ṽ/cm^{-1} = 3531, 3064, 2974, 2925, 1594, 1509, 1465, 1378, 1348, 1266, 1217, 1183, 1109, 1014, 907, 758. **Optical Rotation:** $[\alpha]_D^{20}$ = –25.5 (*c* 1.1, $CHCl_3$): The enantiomeric excess of **1l** was determined by HPLC analysis on a chiral stationary phase (Daicel Chiralpak® IA column, column temperature: 20 °C, *n*-heptane/isopropanol = 90:10, flow rate: 0.60 mL/min, λ = 230 nm): t_R = 9.3 min (minor), t_R = 40.6 min (major).

(*S*)-[(2'-Isopropoxy-7,7'-dimethyl-[1,1'-binaphthalen]-2-yl)oxy](methyl)diphenylsilane [(*S*)-3oa] and (*R*)-2'-Isopropoxy-7,7'-dimethyl-[1,1'-binaphthalen]-2-ol [(*R*)-1o]

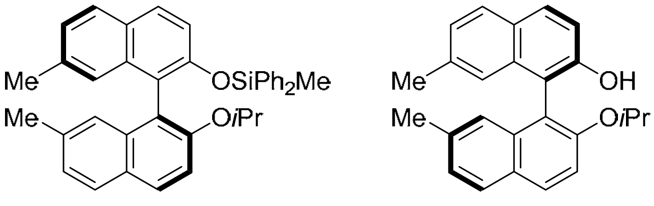

(*S*)-**3oa** $C_{38}H_{36}O_2Si$ M = 552.79 g/mol

(*R*)-**1o** $C_{25}H_{24}O_2$ M = 356.47 g/mol

Prepared according to **GP4** from *rac*-**1o** (71.3 mg, 0.200 mmol, 1.00 equiv) and methyldiphenylsilane (**2a**, 27.8 mg, 0.140 mmol, 0.700 equiv). 4 Å molecular sieves were added to the catalyst mixture, and the reaction mixture was stirred for 52 h at room temperature. Flash column chromatography on silica gel using cyclohexane/*tert*-butyl methyl ether (100:0 → 80:1 → 70:1 → 30:1 → 20:1) afforded silyl ether (*S*)-**3oa** (46.5 mg, 84.1 µmol, 42%, 58% *ee*) as a sticky yellow resin and alcohol (*R*)-**1o** (34.4 mg, 96.5 µmol, 48%, 47% *ee*) as a yellow solid.

Analytical data for silyl ether (*S*)-**3oa**:

R_f = 0.11 (cyclohexane/*tert*-butyl methyl ether = 80:1). **^{1}H NMR** (500 MHz, CD_2Cl_2): δ/ppm = 7.97 (d, *J* = 9.0 Hz, 1H), 7.84 (d, *J* = 8.4 Hz, 1H), 7.76 (d, *J* = 8.3 Hz, 1H), 7.72 (d, *J* = 8.8 Hz, 1H), 7.40 (d, *J* = 9.1 Hz, 1H), 7.36–7.30 (m, 2H), 7.24–7.12 (m, 10H), 7.08 (d, *J* = 8.8 Hz, 1H), 7.06 (s, 1H), 6.99 (s, 1H), 4.52 (sept, *J* = 6.2 Hz, 1H), 2.29 (s, 3H), 2.27 (s, 3H), 1.05 (d, *J* = 6.2 Hz, 3H), 1.04 (d, *J* = 6.2 Hz, 3H), 0.58 (s, 3H). **^{13}C{^{1}H} NMR** (126 MHz, CD_2Cl_2): δ/ppm = 154.6, 151.1, 136.4, 136.3, 136.3, 136.1, 135.1, 134.5 (2C), 134.4 (2C), 130.1, 130.1, 129.1, 128.3, 128.2 (2C), 128.1 (3C), 128.1 (2C), 128.1 (2C), 126.3, 126.3, 125.0, 124.8, 122.4, 121.6, 119.9, 116.9, 72.2, 22.7, 22.6, 22.1, 22.0, –2.5. **^{1}H/^{29}Si HMQC NMR** (500/99 MHz, CD_2Cl_2, optimized for *J* = 7 Hz): δ/ppm = (7.22, 7.14, 0.58)/–5.1. **HRMS** (APCI) calculated for $C_{38}H_{36}O_2Si^+$ [M^+]: 552.2479; found: 552.2482. **IR** (ATR): ṽ/cm^{-1} = 3045, 2971, 2918, 1624, 1506, 1447, 1354, 1323, 1236, 1110, 1036, 990, 886, 827, 792, 734. **Optical Rotation:** $[\alpha]_D^{20}$ = –30.5 (*c* 2.3, dichloromethane). The enantiomeric excess of **3oa** was determined by HPLC analysis on a chiral stationary phase after cleavage of the silyl ether according to **GP5** (Daicel Chiralpak® AD-H column, column temperature: 20 °C, *n*-heptane/isopropanol = 95:5, flow rate: 0.60 mL/min, λ = 250 nm): t_R = 10.8 min (major), t_R = 17.8 min (minor).

Analytical data for alcohol (*R*)-**1o**:

M.p. = 49–52 °C (dichloromethane/*n*-pentane). $\mathbf{R_f}$ = 0.14 (cyclohexane/*tert*-butyl methyl ether = 30:1). **^{1}H NMR** (500 MHz, $CDCl_3$): δ/ppm = 7.95 (d, *J* = 9.0 Hz, 1H), 7.85 (d, *J* = 8.8 Hz, 1H), 7.80 (d, *J* = 8.3 Hz, 1H), 7.76 (d, *J* = 8.3 Hz, 1H), 7.37 (d, *J* = 8.9 Hz, 1H), 7.29 (d, *J* = 8.8 Hz, 1H), 7.22 (d, *J* = 8.2 Hz, 1H), 7.15 (d, *J* = 8.2 Hz, 1H), 6.99 (s, 1H), 6.87 (s, 1H), 4.93 (s, br, 1H), 4.43 (sept, *J* = 6.1 Hz, 1H), 2.28 (s, 3H), 2.26 (s, 3H), 1.09 (d, *J* = 6.1 Hz, 3H), 1.01 (d, *J* = 6.1 Hz, 3H). **^{13}C{^{1}H} NMR** (126 MHz, $CDCl_3$): δ/ppm = 155.1, 151.3, 137.1, 135.8, 134.6, 134.2, 130.4, 129.3, 128.1, 128.1, 127.9, 127.5, 126.8, 125.5, 124.3, 124.1, 117.5, 117.0, 116.6, 115.3, 72.4, 22.4, 22.3, 22.1, 22.0. **HRMS** (APCI) calculated for $C_{25}H_{25}O_2^+$ [(M+H)$^+$]: 357.1849; found: 357.1851. **IR** (ATR): ṽ/cm^{-1} = 3424, 3045, 2973, 2919, 1623, 1509, 1449, 1377, 1316, 1239, 1181, 1109, 991, 917, 830, 732, 682. **Optical Rotation:** $[\alpha]_D^{20}$ = –38.4 (*c* 1.2, $CHCl_3$). The enantiomeric excess of **1o** was determined by HPLC analysis on a chiral stationary phase (Daicel Chiralpak® AD-H column, column temperature: 20 °C, *n*-heptane/isopropanol = 95:5, flow rate: 0.60 mL/min, λ = 250 nm): t_R = 10.8 min (minor), t_R = 17.9 min (major).

(*S*)-[(2'-Isopropoxy-6,6'-dimethyl-[1,1'-binaphthalen]-2-yl)oxy](methyl)diphenylsilane [(*S*)-3qa] and (*R*)-2'-Isopropoxy-6,6'-dimethyl-[1,1'-binaphthalen]-2-ol [(*R*)-1q]

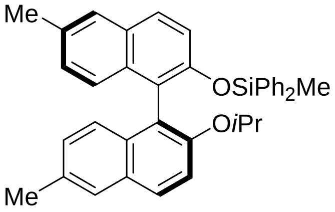

(*S*)-**3qa**
$C_{38}H_{36}O_2Si$
M = 552.79 g/mol

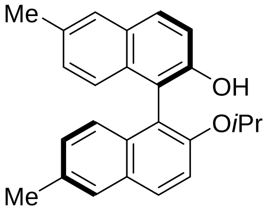

(*R*)-**1q**
$C_{25}H_{24}O_2$
M = 356.47 g/mol

Prepared according to **GP4** from *rac*-**1q** (71.3 mg, 0.200 mmol, 1.00 equiv) and methyldiphenylsilane (**2a**, 27.8 mg, 0.140 mmol, 0.700 equiv). 4 Å molecular sieves were added to the catalyst mixture, and the reaction mixture was stirred for 64 h at room temperature. Flash column chromatography on silica gel using cyclohexane/*tert*-butyl methyl ether (80:1 → 20:1 → 10:1) afforded silyl ether (*S*)-**3qa** (53.8 mg, 97.3 µmol, 49%, 75% *ee*) and alcohol (*R*)-**1q** (30.0 mg, 84.2 µmol, 42%, 81% *ee*) as yellow solids.

Analytical data for silyl ether (*S*)-**3qa**:

M.p. = 33–35 °C (dichloromethane/*n*-pentane). **R$_f$** = 0.17 (cyclohexane/ethyl acetate = 80:1). **^{1}H NMR** (500 MHz, CD_2Cl_2): δ/ppm = 7.91 (d, *J* = 9.0 Hz, 1H), 7.70 (s, 1H), 7.67 (d, *J* = 8.9 Hz, 1H), 7.62 (s, 1H), 7.41 (d, *J* = 9.0 Hz, 1H), 7.36–7.29 (m, 2H), 7.24–7.09 (m, 11H), 7.07 (d, *J* = 9.0 Hz, 1H), 7.01 (d, *J* = 8.6 Hz, 1H), 4.46 (sept, *J* = 6.1 Hz, 1H), 2.47 (s, 3H), 2.46 (s, 3H), 1.05 (d, *J* = 6.1 Hz, 3H), 1.01 (d, *J* = 6.1 Hz, 3H), 0.56 (s, 3H). **^{13}C{^{1}H} NMR** (126 MHz, CD_2Cl_2): δ/ppm = 153.9, 150.4, 136.4, 136.2, 134.5 (2C), 134.4 (2C), 133.5 (2C), 133.0, 133.0, 130.2, 130.1, 130.1, 130.0, 128.8, 128.7 (2C), 128.3, 128.1 (2C), 128.0 (2C), 127.2, 127.1, 125.9, 125.8, 122.8, 122.1, 120.9, 118.1, 72.4, 22.7, 22.6, 21.5, 21.5, –2.4. **^{1}H/^{29}Si HMQC NMR** (500/99 MHz, CD_2Cl_2, optimized for *J* = 7 Hz): δ/ppm = (7.23, 7.16, 0.56)/–4.9. **HRMS** (APCI) calculated for $C_{38}H_{36}O_2Si^+$ [M$^+$]: 552.2479; found: 552.2476. **IR** (ATR): ṽ/cm^{-1} = 3047, 2972, 2917, 1594, 1474, 1428, 1349, 1242, 1112, 1080, 1015, 956, 883, 841, 810, 736, 698. **Optical Rotation:** $[\alpha]_D^{20}$ = –19.3 (*c* 0.86, dichloromethane). The enantiomeric excess of **3qa** was determined by HPLC analysis on a chiral stationary phase after cleavage of the silyl ether according to **GP5** (Daicel Chiralpak® AD-H column, column temperature: 20 °C, *n*-heptane/isopropanol = 90:10, flow rate: 0.60 mL/min, λ = 254 nm): t_R = 9.6 min (major), t_R = 13.5 min (minor).

Analytical data for alcohol (*R*)-**1q**:

M.p. = 50–52 °C (dichloromethane/*n*-pentane). **R$_f$** = 0.18 (cyclohexane/ethyl actetate = 50:1). **^{1}H NMR** (500 MHz, $CDCl_3$): δ/ppm = 7.89 (d, *J* = 9.0 Hz, 1H), 7.81 (d, *J* = 8.9 Hz, 1H), 7.66 (s, 1H), 7.63 (s, 1H), 7.40 (d, *J* = 9.0 Hz, 1H), 7.32 (d, *J* = 8.9 Hz, 1H), 7.12–7.04 (m, 3H), 6.98 (d, *J* = 8.6 Hz, 1H), 5.05 (s, 1H), 4.37 (sept, *J* = 6.1 Hz, 1H), 2.47 (s, 3H), 2.46 (s, 3H), 1.12 (d, *J* = 6.1 Hz, 3H), 0.98 (d, *J* = 6.1 Hz, 3H). **^{13}C{^{1}H} NMR** (126 MHz, $CDCl_3$): δ/ppm = 154.1, 150.8, 134.1, 132.5 (2C), 132.1, 130.2, 129.9, 129.4 (2C), 129.0, 128.5, 127.2 (2C), 125.4, 125.2, 118.7, 118.6, 117.8, 115.8, 73.0, 22.4 (2C), 21.5, 21.5. **HRMS** (APCI) calculated for $C_{25}H_{25}O_2^+$ [(M+H)$^+$]: 357.1849; found: 357.1846. **IR** (ATR): ṽ/cm^{-1} = 3538, 3504, 3437, 2973, 2918, 1725, 1597, 1500, 1476, 1379, 1354, 1327, 1262, 1241, 1205, 1165, 1145, 1110, 1073, 1011, 947, 878, 817. **Optical Rotation:** $[\alpha]_D^{20}$ = –110 (*c* 1.2, $CHCl_3$). The enantiomeric excess of **1q** was determined by HPLC analysis on a chiral stationary phase (Daicel Chiralpak® AD-H column, column temperature: 20 °C, *n*-heptane/isopropanol = 90:10, flow rate: 0.60 mL/min, λ = 254 nm): t_R = 9.5 min (minor), t_R = 13.1 min (major).

(*S*)-[(2'-Isopropoxy-6,6'-diisopropyl-[1,1'-binaphthalen]-2-yl)oxy](methyl)diphenylsilane [(*S*)-3ra] and (*R*)-2'-Isopropoxy-6,6'-diisopropyl-[1,1'-binaphthalen]-2-ol [(*R*)-1r]

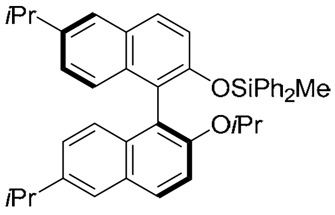

(*S*)-**3ra**
$C_{42}H_{44}O_2Si$
M = 608.90 g/mol

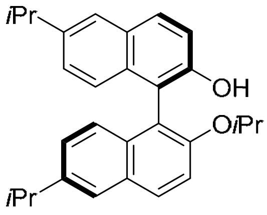

(*R*)-**1r**
$C_{29}H_{32}O_2$
M = 412.57 g/mol

Prepared according to **GP4** from *rac*-**1r** (82.5 mg, 0.200 mmol, 1.00 equiv) and methyldiphenylsilane (**2a**, 27.8 mg, 0.140 mmol, 0.700 equiv). 4 Å molecular sieves were added to the catalyst mixture, and the reaction mixture was stirred for 57 h at room temperature. Flash column chromatography on silica gel using cyclohexane/*tert*-butyl methyl ether (80:1 → 30:1 → 20:1) afforded silyl ether (*S*)-**3ra** (42.9 mg, 70.5 µmol, 35%, 74% *ee*) as a pale brown resin and alcohol (*R*)-**1r** (40.0 mg, 97.0 µmol, 49%, 67% *ee*) as a yellow solid.

Analytical data for silyl ether (*S*)-**3ra**:

$\mathbf{R_f}$ = 0.30 (cyclohexane/ethyl acetate = 80:1). ^{1}**H NMR** (500 MHz, CD_2Cl_2): δ/ppm = 7.94 (d, *J* = 9.0 Hz, 1H), 7.74–7.70 (m, 2H), 7.66 (s, 1H), 7.42 (d, *J* = 9.0 Hz, 1H), 7.36–7.29 (m, 2H), 7.23–7.11 (m, 12H), 7.05 (d, *J* = 8.6 Hz, 1H), 4.48 (sept, *J* = 6.1 Hz, 1H), 3.04 (m_c, 2H), 1.35–1.31 (m, 12H), 1.05 (d, *J* = 6.1 Hz, 3H), 1.03 (d, *J* = 6.1 Hz, 3H), 0.56 (s, 3H). ^{13}**C{^{1}H} NMR** (126 MHz, CD_2Cl_2): δ/ppm = 153.9, 150.5, 144.5, 144.4, 136.4, 136.2, 134.5 (2C), 134.5 (2C), 133.4, 133.4, 130.1 (2C), 130.1, 130.0, 129.0, 128.6, 128.1 (2C), 128.0 (2C), 126.5, 126.4, 126.1, 125.9, 124.4, 124.4, 122.8, 121.9, 120.9, 117.9, 72.4, 34.3 (2C), 24.2, 24.1 (2C), 24.1, 22.7, 22.6, –2.4. ^{1}**H/^{29}Si HMQC NMR** (500/99 MHz, CD_2Cl_2, optimized for *J* = 7 Hz): δ/ppm = (7.22, 7.16, 0.56)/–4.8. **HRMS** (APCI) calculated for $C_{42}H_{45}O_2Si^+$ [(M+H)$^+$]: 609.3183; found: 609.3184. **IR** (ATR): ṽ/cm^{-1} = 3047, 2958, 1593, 1477, 1242, 1179, 1113, 1013, 807, 732. **Optical Rotation:** $[\alpha]_D^{20}$ = –21.8 (*c* 1.5, dichloromethane). The enantiomeric excess of **3ra** was determined by HPLC analysis on a chiral stationary phase after cleavage of the silyl ether according to **GP5** (Daicel Chiralpak® IA column, column temperature: 20 °C, *n*-heptane/isopropanol = 90:10, flow rate: 0.60 mL/min, λ = 230 nm): t_R = 7.5 min (major), t_R = 8.6 min (minor).

Analytical data for alcohol (*R*)-**1r**:

M.p. = 48–50 °C (dichloromethane). **R**$_f$ = 0.25 (cyclohexane/ethyl acetate = 50:1). **^{1}H NMR** (500 MHz, $CDCl_3$): δ/ppm = 7.94 (d, *J* =9.0 Hz, 1H), 7.85 (d, *J* = 8.9 Hz, 1H), 7.70 (s, 1H), 7.66 (s, 1H), 7.41 (d, *J* = 9.0 Hz, 1H), 7.33 (d, *J* = 8.9 Hz, 1H), 7.19 (dd, *J* = 8.8, 1.6 Hz, 1H), 7.16–7.12 (m, 2H), 7.04 (d, *J* = 8.7 Hz, 1H), 5.01 (s, 1H), 4.39 (sept, *J* = 6.1 Hz, 1H), 3.03 (m_c, 2H), 1.35–1.31 (m, 12H), 1.11 (d, *J* = 6.1 Hz, 3H), 1.01 (d, *J* = 6.1 Hz, 3H). **^{13}C{^{1}H} NMR** (126 MHz, $CDCl_3$): δ/ppm = 154.2, 150.8, 144.9, 143.4, 132.9, 132.5, 130.3, 130.2, 129.3 (2C), 127.1, 126.2, 125.5, 125.3, 124.3, 124.3, 118.6, 118.4, 117.5, 115.8, 72.8, 34.0, 33.9, 24.1, 24.1, 24.0, 23.9, 22.4, 22.4. **HRMS** (APCI) calculated for $C_{29}H_{33}O_2^+$ [(M+H)$^+$]: 413.2475; found: 413.2471. **IR** (ATR): ṽ/cm^{-1} = 3541, 2959, 1597, 1462, 1380, 1318, 1241, 1151, 1109, 1007, 971, 884, 826, 690. **Optical Rotation:** $[\alpha]_D^{20}$ = –69.9 (*c* 1.8, $CHCl_3$). The enantiomeric excess of **1r** was determined by HPLC analysis on a chiral stationary phase (Daicel Chiralpak® IA column, column temperature: 20 °C, *n*-heptane/isopropanol = 90:10, flow rate: 0.60 mL/min, λ = 230 nm): t_R = 7.5 min (minor), t_R = 8.6 min (major).

(*S*)-[(2'-Isopropoxy-6,6'-diphenyl-[1,1'-binaphthalen]-2-yl)oxy](methyl)diphenylsilane [(*S*)-3sa] and (*R*)-2'-Isopropoxy-6,6'-diphenyl-[1,1'-binaphthalen]-2-ol [(*R*)-1s]

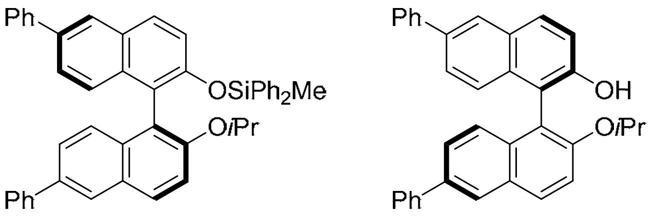

(*S*)-**3sa**
$C_{48}H_{40}O_2Si$
M = 676.93 g/mol

(*R*)-**1s**
$C_{35}H_{28}O_2$
M = 480.61 g/mol

Prepared according to **GP4** from *rac*-**1s** (96.1 mg, 0.200 mmol, 1.00 equiv) and methyldiphenylsilane (**2a**, 27.8 mg, 0.140 mmol, 0.700 equiv). 4 Å molecular sieves were added to the catalyst mixture, and the reaction mixture was stirred for 113 h at room temperature. Flash column chromatography on silica gel using cyclohexane/*tert*-butyl methyl ether (100:0 → 100:1 → 30:1 → 20:1 → 10:1) afforded silyl ether (*S*)-**3sa** (48.9 mg, 72.2 µmol, 36%, 62% *ee*) as a pale yellow solid and alcohol (*R*)-**1s** (36.2 mg, 75.3 µmol, 38%, 43% *ee*) as a colorless solid.

Analytical data for silyl ether (*S*)-**3sa**:

M.p. = 54–60 °C (dichloromethane). **R$_f$** = 0.15 (cyclohexane/ethyl acetate = 50:1). **^{1}H NMR** (500 MHz, CD_2Cl_2): δ/ppm = 8.17 (d, *J* = 1.4 Hz, 1H), 8.12–8.08 (m, 2H), 7.87 (d, *J* = 8.9 Hz, 1H), 7.75–7.71 (m, 4H), 7.59–7.45 (m, 7H), 7.39–7.30 (m, 5H), 7.28–7.19 (m, 10H), 5.57 (sept, *J* = 6.1 Hz, 1H), 1.11 (d, *J* = 6.1 Hz, 3H), 1.08 (d, *J* = 6.1 Hz, 3H), 0.62 (s, 3H). **^{13}C{^{1}H} NMR** (126 MHz, CD_2Cl_2): δ/ppm = 154.7, 151.3, 141.5, 141.4, 136.6, 136.6, 136.2, 136.0, 134.5 (2C), 134.5 (2C), 134.1, 134.0, 130.2, 130.2, 130.1, 129.9, 129.5, 129.2 (4C), 128.1 (2C), 128.1 (2C), 127.5 (5C), 127.5 (2C), 126.7, 126.5, 126.2, 126.2, 126.1, 126.0, 122.7, 121.5 (2C), 118.1, 72.3, 22.7, 22.6, –2.4. **^{1}H/^{29}Si HMQC NMR** (500/99 MHz, CD_2Cl_2, optimized for *J* = 7 Hz): δ/ppm = (7.27, 7.21, 0.62)/–4.4. **HRMS** (APCI) calculated for $C_{48}H_{40}O_2Si^+$ [M$^+$]: 676.2792; found: 676.2790. **IR** (ATR): ṽ/cm^{-1} = 3050, 2974, 1592, 1490, 1428, 1352, 1274, 1244, 1111, 949, 888, 862, 806, 758, 733, 697. **Optical Rotation:** $[\alpha]_D^{20}$ = +38.1 (*c* 1.5, dichloromethane). The enantiomeric excess of **3sa** was determined by HPLC analysis on a chiral stationary phase after cleavage of the silyl ether according to **GP5** (Daicel Chiralpak® IA column, column temperature: 20 °C, *n*-heptane/isopropanol = 90:10, flow rate: 0.60 mL/min, λ = 260 nm): t_R = 16.8 min (major), t_R = 18.8 min (minor).

Analytical data for alcohol (*R*)-**1r**:

M.p. = 79–84 °C (dichloromethane/*n*-pentane). **R$_f$** = 0.18 (cyclohexane/ethyl acetate = 50:1). **^{1}H NMR** (500 MHz, $CDCl_3$): δ/ppm = 8.12 (s, 1H), 8.11 (s, 1H), 8.08 (d, *J* = 9.1 Hz, 1H), 7.99 (d, *J* = 8.9 Hz, 1H), 7.71 (t, *J* = 7.8 Hz, 4H), 7.59–7.44 (m, 7H), 7.42 (d, *J* = 8.8 Hz, 1H), 7.40–7.33 (m, 2H), 7.31 (d, *J* = 8.8 Hz, 1H), 7.21 (d, *J* = 8.8 Hz, 1H), 5.16 (s, 1H), 4.51 (sept, *J* = 6.1 Hz, 1H), 1.18 (d, *J* = 6.1 Hz, 3H), 1.06 (d, *J* = 6.1 Hz, 3H). **^{13}C{^{1}H} NMR** (126 MHz, $CDCl_3$): δ/ppm = 154.9, 151.6, 141.3, 141.0, 137.3, 135.9, 133.5, 133.2, 131.1, 130.2, 130.1, 129.5, 129.0 (2C), 128.9 (2C), 127.4 (2C), 127.4, 127.3 (2C), 127.1, 126.9, 126.1 (2C), 126.0 (2C), 125.9, 118.4, 118.2, 118.0, 115.6, 72.8, 22.4 (2C). **HRMS** (APCI) calculated for $C_{35}H_{28}O_2^+$ [M$^+$]: 480.2084; found: 480.2077. **IR** (ATR): ṽ/cm^{-1} = 3525, 3428, 3057, 3026, 2974, 2928, 1592, 1490, 1444, 1380, 1356, 1329, 1272, 1242, 1212, 1182, 1150, 1107, 1006, 941, 889. **Optical Rotation:** $[\alpha]_D^{20}$ = –98.1 (*c* 1.4, $CHCl_3$). The enantiomeric excess of **1s** was determined by HPLC analysis on a chiral stationary phase (Daicel Chiralpak® IA column, column temperature: 20 °C, *n*-heptane/isopropanol = 90:10, flow rate: 0.60 mL/min, λ = 260 nm): t_R = 16.8 min (minor), t_R = 18.7 min (major).

rac-2' [(5'-Isopropoxy-[4,4'-bibenzo-[_d_][1,3]dioxol]-5-yl)oxy](methyl)diphenylsilane (_rac_-3ta) and _rac_-5'-Isopropoxy-[4,4'-bibenzo[_d_][1,3]dioxol]-5-ol (_rac_-1t)

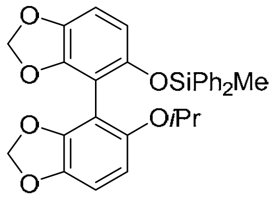

rac-**3ta**
$C_{30}H_{28}O_6Si$
M = 512.63 g/mol

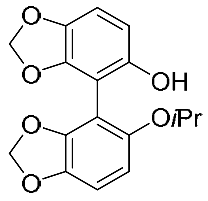

rac-**1t**
$C_{17}H_{16}O_6$
M = 316.31 g/mol

The kinetic resolution was performed according to **GP4** using *rac*-**1t** (63.3 mg, 0.200 mmol, 1.00 equiv) and methyldiphenylsilane (**2a**, 27.8 mg, 0.140 mmol, 0.700 equiv). 4 Å molecular sieves were added to the catalyst mixture, and the reaction mixture was stirred for 7 h at room temperature. The reaction was quenched by filtration over silica, and the HPLC analysis was carried out of the crude mixture, affording no enantiomeric excess for the unreacted alcohol. Thus, no isolation of the pure silyl ether and the alcohol was carried out.

rac-[(2'-Isopropoxy-6,6'-dimethoxy-[1,1'-biphenyl]-2-yl)oxy](methyl)diphenylsilane (_rac_-3ua) and _rac_-2'-Isopropoxy-6,6'-dimethoxy-[1,1'-biphenyl]-2-ol (_rac_-1u)

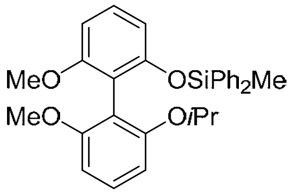

rac-**3ua**
$C_{30}H_{32}O_4Si$
M = 484.67 g/mol

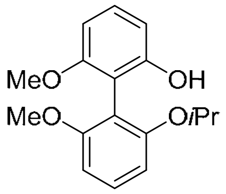

rac-**1u**
$C_{17}H_{20}O_4$
M = 288.34 g/mol

The kinetic resolution was performed according to **GP4** using *rac*-**1u** (57.7 mg, 0.200 mmol, 1.00 equiv) and methyldiphenylsilane (**2a**, 27.8 mg, 0.140 mmol, 0.700 equiv). 4 Å molecular sieves were added to the catalyst mixture, and the reaction mixture was stirred for 8 h at room temperature. The reaction was quenched by filtration over silica, and the HPLC analysis was carried out of the crude mixture, affording no enantiomeric excess for the unreacted alcohol. Thus, no isolation of the pure silyl ether and the alcohol was carried out.

ent-[(7'-Isopropoxy-2,2',3,3'-tetrahydro-1,1'-spirobi[inden]-7-yl)oxy](methyl)diphenyl-silane (*ent*-3va) and *ent*-7'-Isopropoxy-2,2',3,3'-tetrahydro-1,1'-spirobi[inden]-7-ol (*ent*-1v)

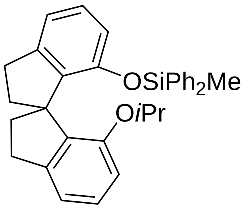

ent-**3va**
$C_{33}H_{34}O_2Si$
M = 490.72 g/mol

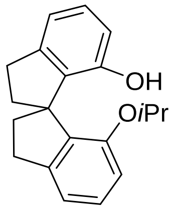

ent-**1v**
$C_{20}H_{22}O_2$
M = 294.39 g/mol

Prepared according to **GP4** from *rac*-**1v** (58.9 mg, 0.200 mmol, 1.00 equiv) and methyldiphenylsilane (**2a**, 27.8 mg, 0.140 mmol, 0.700 equiv). 4 Å molecular sieves were added to the catalyst mixture, and the reaction mixture was stirred for 18 h at room temperature. Flash column chromatography on silica gel using cyclohexane/*tert*-butyl methyl ether (100:1 → 50:1 → 20:1 → 10:1) afforded silyl ether *ent*-**3va** (10.8 mg, 22.0 μmol, 11%, 30% *ee*) and alcohol *ent*-**1v** (14.6 mg, 49.6 μmol, 25%, 48% *ee*) as colorless oils.

Note: Some material was lost during the purification process, explaining the low yields of **3va** and **1v**. However, considering the low selectivity of the reaction, the experiment was not repeated. No determination of the absolute configuration of **3va** and **1v** was carried out. Due to the structural uniqueness of **3va** and **1v** compared to the other substrates, an assignment by analogy seemed inappropriate.

Analytical data for silyl ether *ent*-**3va**:

R_f = 0.71 (cyclohexane/*tert*-butyl methyl ether = 50:1). **^{1}H NMR** (500 MHz, CD_2Cl_2): δ/ppm = 7.37–7.32 (m, 2H), 7.29–7.22 (m, 8H), 7.12 (t, J = 7.8 Hz, 1H), 6.85 (t, J = 7.8 Hz, 1H), 6.79 (t, J = 7.7 Hz, 2H), 6.62 (d, J = 8.1 Hz, 1H), 6.39 (d, J = 7.9 Hz, 1H), 4.34 (sept, J = 6.0 Hz, 1H), 3.04–2.94 (m, 3H), 2.87–2.80 (m, 1H), 2.46 (m_c, 1H), 2.30–2.10 (m, 3H), 1.03 (d, J = 6.0 Hz, 3H), 0.75 (d, J = 6.0 Hz, 3H), 0.54 (s, 3H). **^{13}C{^{1}H} NMR** (126 MHz, CD_2Cl_2): δ/ppm = 154.8, 152.3, 146.6, 145.7, 139.2, 138.2, 136.5, 136.2, 134.5 (2C), 134.5 (2C), 130.1, 130.0, 128.1 (2C), 128.1 (2C), 127.9, 127.3, 117.5, 116.8, 116.1, 110.3, 68.7, 59.8, 38.8, 38.5, 32.0, 31.7, 21.9, 21.8, –2.6. **^{1}H/^{29}Si HMQC NMR** (500/99 MHz, CD_2Cl_2, optimized for J = 7 Hz): δ/ppm = (7.28, 7.23, 0.54)/–6.0. **HRMS** (APCI) calculated for $C_{27}H_{29}O_2Si^+$ [(M–C_6H_5)$^+$]: 413.1931; found: 413.1927. **IR** (ATR): ṽ/cm^{-1} = 3049, 2932, 1585, 1471, 1262, 1118, 1026,

842, 777, 736. **Optical Rotation:** $[\alpha]_D^{20}$ = –7.4 (*c* 0.54, dichloromethane). The enantiomeric excess of **3va** was determined by HPLC analysis on a chiral stationary phase after cleavage of the silyl ether according to **GP5** (Daicel Chiralpak® AD-H column, column temperature: 20 °C, *n*-heptane/isopropanol = 95:5, flow rate: 0.60 mL/min, λ = 280 nm): t_R = 9.6 min (major), t_R = 10.8 min (minor).

Analytical data for alcohol *ent*-**1v**:

R_f = 0.37 (cyclohexane/*tert*-butyl methyl ether = 20:1). **^{1}H NMR** (500 MHz, $CDCl_3$): δ/ppm = 7.20 (t, *J* = 7.8 Hz, 1H), 7.06 (t, *J* = 7.7 Hz, 1H), 6.86 (d, *J* = 7.4 Hz, 1H), 6.80 (d, *J* = 7.3 Hz, 1H), 6.67 (d, *J* = 8.1 Hz, 1H), 6.57 (d, *J* = 8.0 Hz, 1H), 4.41 (sept, *J* = 6.0 Hz, 1H), 3.09–2.91 (m, 4H), 2.42–2.26 (m, 2H), 2.24–2.12 (m, 2H), 1.04 (d, *J* = 6.0 Hz, 3H), 0.91 (d, *J* = 6.0 Hz, 3H). The signal of the OH-group could not be detected. **^{13}C{^{1}H} NMR** (126 MHz, $CDCl_3$): δ/ppm = 155.0, 152.6, 146.6, 144.8, 134.6, 133.4, 129.6, 128.0, 117.1, 116.7, 113.3, 110.4, 68.9, 58.5, 37.9, 37.6, 31.5, 31.5, 22.1, 21.1. **HRMS** (APCI) calculated for $C_{20}H_{23}O_2^+$ [$(M+H)^+$]: 295.1693; found: 295.1690. **IR** (ATR): ṽ/cm^{-1} = 3525, 3033, 2934, 1587, 1470, 1383, 1264, 1181, 1116, 776. **Optical Rotation:** $[\alpha]_D^{20}$ = +28.1 (*c* 0.73, $CHCl_3$). The enantiomeric excess of **1v** was determined by HPLC analysis on a chiral stationary phase (Daicel Chiralpak® AD-H column, column temperature: 20 °C, *n*-heptane/isopropanol = 95:5, flow rate: 0.60 mL/min, λ = 280 nm): t_R = 9.6 min (minor), t_R = 10.8 min (major).

(*S*)-[(2'-Isopropoxy-6,6'-dimethyl-[1,1'-biphenyl]-2-yl)oxy](methyl)diphenylsilane [(*S*)-3wa] and (*R*)-2'-Isopropoxy-6,6'-dimethyl-[1,1'-biphenyl]-2-ol [(*R*)-1w]

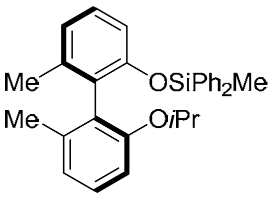

(*S*)-**3wa**
$C_{30}H_{32}O_2Si$
M = 452.67 g/mol

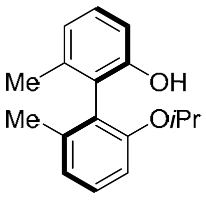

(*R*)-**1w**
$C_{17}H_{20}O_2$
M = 256.35 g/mol

Prepared according to **GP4** from *rac*-**1w** (51.3 mg, 0.200 mmol, 1.00 equiv) and methyldiphenylsilane (**2a**, 27.8 mg, 0.140 mmol, 0.700 equiv). 4 Å molecular sieves were added to the catalyst mixture, and the reaction mixture was stirred for 4 h at room temperature. Flash column chromatography on silica gel using cyclohexane/*tert*-butyl methyl ether (90:1 → 50:1) afforded silyl ether (*S*)-**3wa** (38.8 mg, 85.7 µmol, 43%, 84% *ee*) as a colorless oil and alcohol (*R*)-**1w** (11.5 mg, 44.9 µmol, 22%, 79% *ee*) as a colorless resin.

Analytical data for silyl ether (*S*)-**3wa**:

R$_f$ = 0.32 (cyclohexane/ethyl acetate = 80:1). **^{1}H NMR** (500 MHz, CD_2Cl_2): δ/ppm = 7.40–7.32 (m, 4H), 7.33–7.22 (m, 7H), 7.02 (t, *J* = 7.8 Hz, 1H), 6.94 (d, *J* = 7.6 Hz, 1H), 6.90 (d, *J* = 7.6 Hz, 1H), 6.86 (d, *J* = 8.3 Hz, 1H), 6.61 (d, *J* = 8.1 Hz, 1H), 4.39 (sept, *J* = 6.1 Hz, 1H), 2.01 (s, 3H), 1.98 (s, 3H), 1.16 (d, *J* = 6.1 Hz, 3H), 1.05 (d, *J* = 6.1 Hz, 3H), 0.63 (s, 3H). **^{13}C{^{1}H} NMR** (126 MHz, CD_2Cl_2): δ/ppm = 156.0, 152.7, 139.1, 139.0, 136.7, 136.4, 134.5 (2C), 134.5 (2C), 130.2, 130.1, 129.9, 128.4, 128.2 (2C), 128.1 (2C), 128.0, 127.7, 123.0, 122.5, 116.2, 111.9, 70.5, 22.4, 22.2, 20.0, 20.0, –2.6. **^{1}H/^{29}Si HMQC NMR** (500/99 MHz, CD_2Cl_2, optimized for *J* = 7 Hz): δ/ppm = (7.37, 7.26, 0.63)/–5.9. **HRMS** (APCI) calculated for $C_{30}H_{32}O_2Si^+$ [M$^+$]: 452.2166; found: 452.2160. **IR** (ATR): ṽ/cm^{-1} = 3049, 2973, 1577, 1459, 1372, 1253, 1117, 1048, 971, 837, 791, 732. **Optical Rotation:** $[\alpha]_D^{20}$ = –19.9 (*c* 1.4, dichloromethane). The enantiomeric excess of **3wa** was determined by HPLC analysis on a chiral stationary phase after cleavage of the silyl ether according to **GP5** (Daicel Chiralpak® IA column, column temperature: 20 °C, *n*-heptane/isopropanol = 90:10, flow rate: 0.60 mL/min, λ = 280 nm): t_R = 7.5 min (major), t_R = 12.5 min (minor).

Analytical data for alcohol (*R*)-**1w**:

R$_f$ = 0.13 (cyclohexane/ethyl acetate = 50:1). **^{1}H NMR** (500 MHz, $CDCl_3$): δ/ppm = 7.25–7.22 (m, 1H), 7.13 (t, *J* = 7.8 Hz, 1H), 6.92 (d, *J* = 7.6 Hz, 1H), 6.86–6.78 (m, 3H), 4.53 (s, 1H), 4.34 (sept, *J* = 6.1 Hz, 1H), 1.98 (s, 3H), 1.94 (s, 3H), 1.13 (d, *J* = 6.1 Hz, 3H), 1.10 (d, *J* = 6.1 Hz, 3H). **^{13}C{^{1}H} NMR** (126 MHz, $CDCl_3$): δ/ppm = 156.4, 152.8, 139.9, 137.9, 129.4, 128.3, 124.4, 124.0, 123.0, 121.8, 112.4, 112.2, 70.8, 22.1, 22.1, 19.8, 19.7. **HRMS** (APCI) calculated for $C_{17}H_{21}O_2^+$ [(M+H)$^+$]: 257.1536; found: 257.1535. **IR** (ATR): ṽ/cm^{-1} = 3498, 3439, 3029, 2975, 2922, 1577, 1460, 1373, 1255, 1179, 1118, 1046, 974, 778. **Optical Rotation:** $[\alpha]_D^{20}$ = –19.7 (*c* 0.47, $CHCl_3$). The enantiomeric excess of **1w** was determined by HPLC analysis on a chiral stationary phase (Daicel Chiralpak® IA column, column temperature: 20 °C, *n*-heptane/isopropanol = 90:10, flow rate: 0.60 mL/min, λ = 280 nm): t_R = 7.5 min (minor), t_R = 12.5 min (major).

(*S*)-[(2',6-Diethyl-6'-isopropoxy-[1,1'-biphenyl]-2-yl)oxy](methyl)diphenylsilane [(*S*)-3xa] and (*R*)-2',6-Diethyl-6'-isopropoxy-[1,1'-biphenyl]-2-ol [(*R*)-1x]

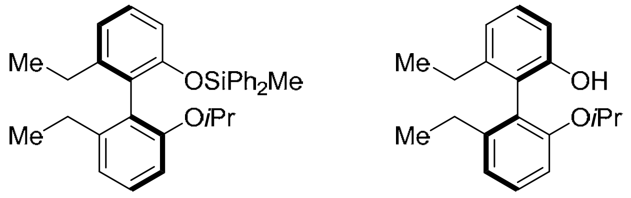

(*S*)-**3xa**
$C_{32}H_{36}O_2Si$
M = 480.72 g/mol

(*R*)-**1x**
$C_{19}H_{24}O_2$
M = 284.40 g/mol

Prepared according to **GP4** from *rac*-**1x** (56.9 mg, 0.200 mmol, 1.00 equiv) and methyldiphenylsilane (**2a**, 27.8 mg, 0.140 mmol, 0.700 equiv). 4 Å molecular sieves were added to the catalyst mixture, and the reaction mixture was stirred for 4 h at room temperature. Preparative TLC (cyclohexane/*tert*-butyl methyl ether = 100:1) afforded silyl ether (*S*)-**3xa** (33.9 mg, 70.5 µmol, 35%, 86% *ee*) and alcohol (*R*)-**1x** (24.4 mg, 85.8 µmol, 43%, 83% *ee*) as yellow oils.

Analytical data for silyl ether (*S*)-**3xa**:

$\mathbf{R_f}$ = 0.51 (cyclohexane/ethyl acetate = 80:1). **^{1}H NMR** (500 MHz, CD_2Cl_2): δ/ppm = 7.39–7.31 (m, 5H), 7.30–7.26 (m, 2H), 7.25–7.20 (m, 4H), 7.07 (t, *J* = 7.8 Hz, 1H), 6.99 (d, *J* = 7.6 Hz, 1H), 6.93 (d, *J* = 7.6 Hz, 1H), 6.83 (d, *J* = 8.2 Hz, 1H), 6.59 (d, *J* = 8.0 Hz, 1H), 4.38 (sept, *J* = 6.1 Hz, 1H), 2.37–2.27 (m, 4H), 1.14 (d, *J* = 8.0 Hz, 3H), 1.06 (t, *J* = 7.7 Hz, 3H), 1.04–0.98 (m, 6H), 0.63 (s, 3H). **^{13}C{^{1}H} NMR** (126 MHz, CD_2Cl_2): δ/ppm = 156.0, 152.7, 145.2, 145.0, 136.8, 136.5, 134.5 (4C), 130.1, 130.0, 129.1, 128.2 (2C), 128.2, 128.1 (2C), 127.8, 127.5, 121.0, 120.5, 115.9, 111.4, 70.2, 26.7, 26.6, 22.3, 22.2, 14.7 (2C), –2.6. **^{1}H/^{29}Si HMQC NMR** (500/99 MHz, CD_2Cl_2, optimized for *J* = 7 Hz): δ/ppm = (7.36, 7.23, 0.63)/–6.4. **HRMS** (APCI) calculated for $C_{32}H_{36}O_2Si^+$ [M$^+$]: 480.2479; found: 480.2472. **IR** (ATR): $\tilde{\nu}$/cm^{-1} = 3050, 2967, 1574, 1460, 1382, 1252, 1118, 1056, 1016, 954, 793, 736. **Optical Rotation:** $[\alpha]_D^{20}$ = –18.5 (*c* 1.4, dichloromethane). The enantiomeric excess of **3xa** was determined by HPLC analysis on a chiral stationary phase after cleavage of the silyl ether according to **GP5** (Daicel Chiralpak® IA column, column temperature: 20 °C, *n*-heptane/isopropanol = 98:2, flow rate: 0.60 mL/min, λ = 210 nm): t_R = 9.9 min (major), t_R = 20.5 min (minor).

Analytical data for alcohol (*R*)-**1x**:

R$_f$ = 0.13 (cyclohexane/ethyl acetate = 50:1). **^{1}H NMR** (500 MHz, $CDCl_3$): δ/ppm = 7.33 (t, *J* = 8.0 Hz, 1H), 7.22 (t, *J* = 7.8 Hz, 1H), 6.98 (d, *J* = 7.6 Hz, 1H), 6.89 (d, *J* = 7.5 Hz, 1H), 6.86 (d, *J* = 8.2 Hz, 1H), 6.82 (d, *J* = 8.0 Hz, 1H), 4.53 (s, br, 1H), 4.39 (sept, *J* = 6.1 Hz, 1H), 2.30 (q, *J* = 7.6 Hz, 2H), 2.26 (q, *J* = 7.6 Hz, 2H), 1.15 (d, *J* = 6.1 Hz, 3H), 1.12 (d, *J* = 6.1 Hz, 3H), 1.04 (t, *J* = 7.6 Hz, 6H). **^{13}C{^{1}H} NMR** (126 MHz, $CDCl_3$): δ/ppm = 156.4, 152.9, 146.1, 143.9, 129.7, 128.4, 123.3, 123.1, 121.1, 119.8, 112.1, 111.8, 70.5, 26.4, 26.3, 22.1, 22.0, 14.8, 14.6. **HRMS** (APCI) calculated for $C_{19}H_{25}O_2^+$ [(M+H)$^+$]: 285.1849; found: 285.1845. **IR** (ATR): ṽ/cm^{-1} = 3540, 3061, 2967, 2930, 1574, 1458, 1372, 1332, 1254, 1177, 1118, 1056, 1014, 959, 795, 744. **Optical Rotation:** $[\alpha]_D^{20}$ = +13.7 (*c* 0.69, $CHCl_3$).The enantiomeric excess of **1x** was determined by HPLC analysis on a chiral stationary phase (Daicel Chiralpak® IA column, column temperature: 20 °C, *n*-heptane/isopropanol = 98:2, flow rate: 0.60 mL/min, λ = 210 nm): t_R = 10.0 min (minor), t_R = 20.7 min (major).

(*S*)-[(2',6-Diisobutyl-6'-isopropoxy-[1,1'-biphenyl]-2-yl)oxy](methyl)diphenylsilane [(*S*)-3ya] and (*R*)-2',6-Diisobutyl-6'-isopropoxy-[1,1'-biphenyl]-2-ol [(*R*)-1y]

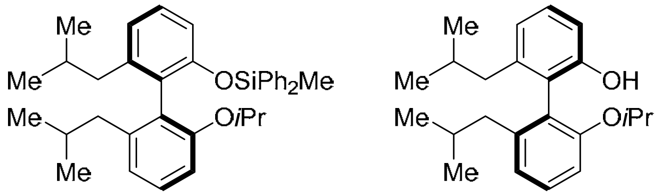

(*S*)-**3ya**
$C_{36}H_{44}O_2Si$
M = 536.83 g/mol

(*R*)-**1y**
$C_{23}H_{32}O_2$
M = 340.51 g/mol

Prepared according to **GP4** from *rac*-**1y** (68.1 mg, 0.200 mmol, 1.00 equiv) and methyldiphenylsilane (**2a**, 27.8 mg, 0.140 mmol, 0.700 equiv). 4 Å molecular sieves were added to the catalyst mixture, and the reaction mixture was stirred for 21 h at room temperature. Preparative TLC (cyclohexane/*tert*-butyl methyl ether = 100:1) afforded silyl ether (*S*)-**3ya** (32.7 mg, 60.9 μmol, 30%, 83% *ee*) as a yellow resin and alcohol (*R*)-**1y** (16.0 mg, 47.0 μmol, 24%, 95% *ee*) as a yellow oil.

Analytical data for silyl ether (*S*)-**3ya**:

R$_f$ = 0.49 (cyclohexane/ethyl acetate = 80:1). **^{1}H NMR** (500 MHz, CD_2Cl_2): δ/ppm = 7.38–7.31 (m, 5H), 7.30–7.21 (m, 6H), 7.03 (t, *J* = 7.8 Hz, 1H), 6.91 (d, *J* = 7.6 Hz, 1H), 6.86 (d, *J* = 7.6 Hz, 1H), 6.81 (d, *J* = 8.2 Hz, 1H), 6.58 (d, *J* = 8.0 Hz, 1H), 4.37 (sept, *J* = 6.0 Hz, 1H), 2.26–

2.10 (m, 4H), 1.78 (non, *J* = 6.8 Hz, 2H), 1.12 (d, *J* = 6.0 Hz, 3H), 0.98 (d, *J* = 6.0 Hz, 3H), 0.79 (d, *J* = 6.6 Hz, 3H), 0.73 (m_c, 6H), 0.69–0.64 (m, 6H). **^{13}C{^{1}H} NMR** (126 MHz, CD_2Cl_2): δ/ppm = 142.4, 142.2, 136.9, 136.6, 134.5 (2C), 134.5 (2C), 130.1, 130.0, 130.0, 129.6, 128.2 (2C), 128.2, 128.1 (2C), 127.9, 127.6, 127.3, 122.4, 121.8, 115.6, 110.6, 69.6, 43.3 (2C), 28.2, 28.2, 23.2, 23.1, 22.7, 22.6, 22.2 (2C), –2.7. **^{1}H/^{29}Si HMQC NMR** (500/99 MHz, CD_2Cl_2, optimized for *J* = 7 Hz): δ/ppm = (7.36, 7.29, 0.66)/–7.2. **HRMS** (APCI) calculated for $C_{36}H_{45}O_2Si^+$ [(M+H)$^+$]: 537.3183; found: 537.3177. **IR** (ATR): ṽ/cm^{-1} = 3051, 2952, 1573, 1458, 1382, 1252, 1117, 1024, 947, 848, 790, 735. **Optical Rotation:** $[\alpha]_D^{20}$ = +4.3 (*c* 1.3, dichloromethane). The enantiomeric excess of **3ya** was determined by HPLC analysis on a chiral stationary phase after cleavage of the silyl ether according to **GP5** (Daicel Chiralpak® IA column, column temperature: 20 °C, *n*-heptane/isopropanol = 98:2, flow rate: 0.60 mL/min, λ = 210 nm): t_R = 7.8 min (major), t_R = 10.4 min (minor).

Analytical data for alcohol (*R*)-**1y**:

R_f = 0.27 (cyclohexane/ethyl acetate = 50:1). **^{1}H NMR** (500 MHz, $CDCl_3$): δ/ppm = 7.29 (t, *J* = 8.0 Hz, 1H), 7.17 (t, *J* = 7.8 Hz, 1H), 6.91 (d, *J* = 7.7 Hz, 1H), 6.84–6.78 (m, 3H), 4.56 (s, br, 1H), 4.43 (sept, *J* = 6.0 Hz, 1H), 2.11 (d, *J* = 7.3 Hz, 2H), 2.11 (d, *J* = 7.3 Hz, 2H), 1.67 (non, *J* = 6.7 Hz, 2H), 1.17 (d, *J* = 6.0 Hz, 3H), 1.14 (d, *J* = 6.0 Hz, 3H), 0.78 (d, *J* = 6.6 Hz, 3H), 0.75 (d, *J* = 6.7 Hz, 3H), 0.67 (m_c, 6H). **^{13}C{^{1}H} NMR** (126 MHz, $CDCl_3$): δ/ppm = 156.1, 153.3, 143.7, 141.3, 129.1, 128.0, 123.9, 123.7, 122.4, 121.6, 112.3, 110.7, 69.8, 43.1, 42.7, 28.6, 28.3, 22.9, 22.7, 22.7, 22.6, 22.0, 21.9. **HRMS** (APCI) calculated for $C_{23}H_{33}O_2^+$ [(M+H)$^+$]: 341.2475; found: 341.2471. **IR** (ATR): ṽ/cm^{-1} = 3543, 3058, 2954, 1574, 1457, 1383, 1256, 1174, 1117, 1022, 948, 749. **Optical Rotation:** $[\alpha]_D^{20}$ = –30.8 (*c* 0.74, $CHCl_3$). The enantiomeric excess of **1y** was determined by HPLC analysis on a chiral stationary phase (Daicel Chiralpak® IA column, column temperature: 20 °C, *n*-heptane/isopropanol = 98:2, flow rate: 0.60 mL/min, λ = 210 nm): t_R = 7.9 min (minor), t_R = 10.5 min (major).

(*S*)-[(6'-Isopropoxy-2',3',5,6-tetramethyl-[1,1'-biphenyl]-2-yl)oxy](methyl)diphenylsilane [(*S*)-3za] and (*R*)-6'-Isopropoxy-2',3',5,6-tetramethyl-[1,1'-biphenyl]-2-ol [(*R*)-1z]

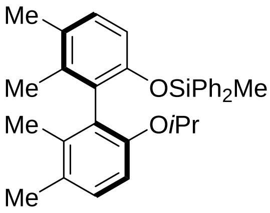

(*S*)-**3za**
$C_{32}H_{36}O_2Si$
M = 480.72 g/mol

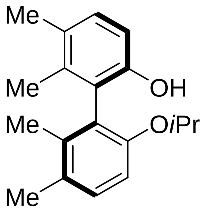

(*R*)-**1z**
$C_{19}H_{24}O_2$
M = 284.40 g/mol

Prepared according to **GP4** from *rac*-**1z** (56.9 mg, 0.200 mmol, 1.00 equiv) and methyldiphenylsilane (**2a**, 27.8 mg, 0.140 mmol, 0.700 equiv). 4 Å molecular sieves were added to the catalyst mixture, and the reaction mixture was stirred for 7 h at room temperature. Flash column chromatography on silica gel using cyclohexane/ethyl acetate (100:0 → 80:1 → 50:1 → 20:1) afforded silyl ether (*S*)-**3za** (52.6 mg, 0.109 mmol, 55%, 53% *ee*) as a colorless oil and alcohol (*R*)-**1z** (18.4 mg, 64.7 µmol, 32%, 92% *ee*) as a colorless solid.

Analytical data for silyl ether (*S*)-**3za**:

R_f = 0.35 (cyclohexane/ethyl acetate = 80:1). **^{1}H NMR** (500 MHz, CD_2Cl_2): δ/ppm = 7.40–7.32 (m, 4H), 7.32–7.22 (m, 6H), 7.17 (d, *J* = 8.2 Hz, 1H), 6.92 (d, *J* = 8.0 Hz, 1H), 6.78 (d, *J* = 8.2 Hz, 1H), 6.53 (s, *J* = 8.0 Hz, 1H), 4.31 (sept, *J* = 6.0 Hz, 1H), 2.28 (s, 3H), 2.25 (s, 3H), 1.92 (s, 3H), 1.86 (s, 3H), 1.13 (d, *J* = 6.0 Hz, 3H), 1.04 (d, *J* = 6.0 Hz, 3H), 0.61 (s, 3H). **^{13}C{^{1}H} NMR** (126 MHz, CD_2Cl_2): δ/ppm = 154.3, 150.8, 137.3, 137.3, 136.9, 136.7, 134.5 (2C), 134.5 (2C), 130.4, 130.1, 130.0, 129.6, 129.3, 129.2, 129.0, 128.8, 128.1 (2C), 128.0 (2C), 115.6, 112.2, 70.7, 22.4, 22.3, 20.0 (2C), 16.9, 16.7, –2.6. **^{1}H/^{29}Si HMQC NMR** (500/99 MHz, CD_2Cl_2, optimized for *J* = 7 Hz): δ/ppm = (7.35, 7.26, 0.61)/–6.3. **HRMS** (APCI) calculated for $C_{32}H_{36}O_2Si^+$ [M$^+$]: 480.2479; found: 480.2470. **IR** (ATR): ṽ/cm^{-1} = 3048, 2973, 2929, 1590, 1465, 1382, 1253, 1117, 1047, 973, 870, 794, 727. **Optical Rotation:** $[\alpha]_D^{20}$ = –29.6 (*c* 0.59, dichloromethane). The enantiomeric excess of **3za** was determined by HPLC analysis on a chiral stationary phase after cleavage of the silyl ether according to **GP5** (Daicel Chiralpak® IA column, column temperature: 20 °C, *n*-heptane/isopropanol = 90:10, flow rate: 0.60 mL/min, λ = 230 nm): t_R = 8.1 min (major), t_R = 30.4 min (minor).

Analytical data for alcohol (*R*)-**1z**:

M.p. = 87–90 °C (dichloromethane). **R$_f$** = 0.17 (cyclohexane/ethyl acetate = 50:1). **^{1}H NMR** (500 MHz, $CDCl_3$): δ/ppm = 7.15 (d, *J* = 8.3 Hz, 1H), 7.04 (d, *J* = 8.2 Hz, 1H), 6.79 (d, *J* = 8.4 Hz, 1H), 6.74 (d, *J* = 8.2 Hz, 1H), 4.44 (s, 1H), 4.24 (sept, *J* = 6.1 Hz, 1H), 2.27 (s, 3H), 2.25 (s, 3H), 1.89 (s, 3H), 1.85 (s, 3H), 1.10 (d, *J* = 6.1 Hz, 3H), 1.08 (d, *J* = 6.1 Hz, 3H). **^{13}C{^{1}H} NMR** (126 MHz, $CDCl_3$): δ/ppm = 154.5, 150.9, 138.1, 136.0, 130.4, 130.0, 129.4, 128.1, 125.6, 124.7, 113.1, 111.7, 71.2, 22.2, 22.1, 20.1, 20.0, 16.7, 16.4. **HRMS** (APCI) calculated for $C_{19}H_{25}O_2^+$ [(M+H)$^+$]: 285.1849; found: 285.1846. **IR** (ATR): ṽ/cm^{-1} = 3539, 3432, 2974, 2928, 1590, 1468, 1382, 1327, 1268, 1186, 1115, 1042, 914, 809, 735. **Optical Rotation:** $[\alpha]_D^{20}$ = +21.4 (*c* 0.76, $CHCl_3$). The enantiomeric excess of **1z** was determined by HPLC analysis on a chiral stationary phase (Daicel Chiralpak® IA column, column temperature: 20 °C, *n*-heptane/isopropanol = 90:10, flow rate: 0.60 mL/min, λ = 230 nm): t_R = 8.1 min (minor), t_R = 30.5 min (major).

(*S*)-[(2'-Isopropoxy-4,4',6,6'-tetramethyl-[1,1'-biphenyl]-2-yl)oxy](methyl)diphenylsilane [(*S*)-3a'a] and (*R*)-2'-Isopropoxy-4,4',6,6'-tetramethyl-[1,1'-biphenyl]-2-ol [(*R*)-1a']

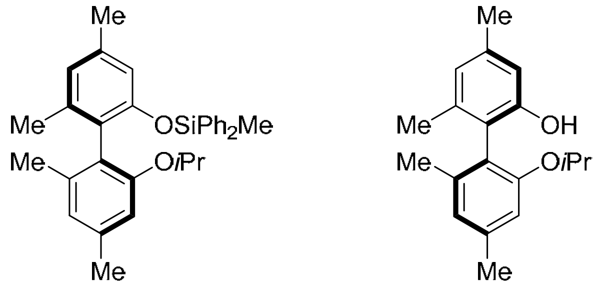

(*S*)-**3a'a** $C_{32}H_{36}O_2Si$ M = 480.72 g/mol

(*R*)-**1a'** $C_{19}H_{24}O_2$ M = 284.40 g/mol

Prepared according to **GP4** from *rac*-**1a'** (55.0 mg, 0.193 mmol, 1.00 equiv) and methyldiphenylsilane (**2a**, 26.8 mg, 0.135 mmol, 0.700 equiv). 4 Å molecular sieves were added to the catalyst mixture, and the reaction mixture was stirred for 25 h at room temperature. Preparative TLC (cyclohexane/ethyl acetate = 50:1) afforded silyl ether (*S*)-**3a'a** (49.3 mg, 0.103 mmol, 53%, 52% *ee*) as a pale yellow oil and alcohol (*R*)-**1a'** (16.8 mg, 59.1 µmol, 31%, 97% *ee*) as a yellow oil.

Analytical data for silyl ether (*S*)-**3a'a**:

R$_f$ = 0.58 (cyclohexane/ ethyl acetate = 50:1). **^{1}H NMR** (500 MHz, CD_2Cl_2): δ/ppm = 7.38–7.25 (m, 6H), 7.25–7.19 (m, 4H), 6.75 (s, 1H), 6.72 (s, 1H), 6.65 (s, 1H), 6.43 (s, 1H), 4.34 (sept, *J* = 6.0 Hz, 1H), 2.41 (s, 3H), 2.20 (s, 3H), 1.95 (s, 3H), 1.91 (s, 3H), 1.14 (d, *J* = 6.0 Hz, 3H), 1.03 (d, *J* = 6.0 Hz, 3H), 0.61 (s, 3H). **^{13}C{^{1}H} NMR** (126 MHz, CD_2Cl_2): δ/ppm = 156.1, 152.7, 138.8, 138.8, 137.7, 137.4, 137.0, 136.6, 134.5 (4C), 130.1, 130.0, 128.1 (2C), 128.0 (2C), 126.9, 125.4, 123.9, 123.3, 117.1, 112.8, 70.4, 22.4, 22.3, 21.8, 21.4, 20.0 (2C), –2.6. **^{1}H/^{29}Si HMQC NMR** (500/99 MHz, CD_2Cl_2, optimized for *J* = 7 Hz): δ/ppm = (7.34, 7.22, 0.61)/–6.2. **HRMS** (APCI) calculated for $C_{26}H_{31}O_2Si^+$ [$(M–C_6H_5)^+$]: 403.2088; found: 403.2081. **IR** (ATR): ṽ/cm^{-1} = 2973, 2919, 1570, 1427, 1314, 1166, 1117, 1073, 972, 829, 793, 736. **Optical Rotation:** $[\alpha]_D^{20}$ = –3.8 (*c* 1.2, dichloromethane). The enantiomeric excess of **3a'a** was determined by HPLC analysis on a chiral stationary phase after cleavage of the silyl ether according to **GP5** (Daicel Chiralpak® AD-H column, column temperature: 20 °C, *n*-heptane/isopropanol = 97:3, flow rate: 0.60 mL/min, λ = 230 nm): t_R = 10.4 min (major), t_R = 36.1 min (minor).

Analytical data for alcohol (*R*)-**1a'**:

R$_f$ = 0.19 (cyclohexane/ethyl acetate = 50:1). **^{1}H NMR** (500 MHz, $CDCl_3$): δ/ppm = 6.76 (s, 1H), 6.67 (s, 1H), 6.65 (s, 1H), 6.63 (s, 1H), 4.53 (s, 1H), 4.33 (sept, *J* = 6.0 Hz, 1H), 2.35 (s, 3H), 2.31 (s, 3H), 1.95 (s, 3H), 1.91 (s, 3H), 1.14 (d, *J* = 6.0 Hz, 3H), 1.11 (d, *J* = 6.0 Hz, 3H). **^{13}C{^{1}H} NMR** (126 MHz, $CDCl_3$): δ/ppm = 156.5, 152.7, 139.7, 139.2, 138.0, 137.7, 123.9, 122.7, 121.5, 121.0, 113.5, 112.8, 70.9, 22.2, 22.1, 21.7, 21.4, 19.8, 19.7. **HRMS** (APCI) calculated for $C_{19}H_{25}O_2^+$ [$(M+H)^+$]: 285.1849; found: 285.1847. **IR** (ATR): ṽ/cm^{-1} = 3538, 2974, 2922, 1727, 1570, 1453, 1381, 1301, 1179, 1115, 1070, 972, 908, 836, 749. **Optical Rotation:** $[\alpha]_D^{20}$ = +14.4 (*c* 0.57, $CHCl_3$). The enantiomeric excess of **1a'** was determined by HPLC analysis on a chiral stationary phase (Daicel Chiralpak® AD-H column, column temperature: 20 °C, *n*-heptane/isopropanol = 97:3, flow rate: 0.60 mL/min, λ = 230 nm): t_R = 10.4 min (minor), t_R = 36.4 min (major).

(*S*)-[(14-Isopropoxy-5,6,7,8,9,10-hexahydrodibenzo[*a*,*c*][10]annulen-1-yl)oxy](methyl)-diphenylsilane [(*S*)-3b'a] and (*R*)-14-Isopropoxy-5,6,7,8,9,10-hexahydrodibenzo-[*a*,*c*][10]annulen-1-ol [(*R*)-1b']

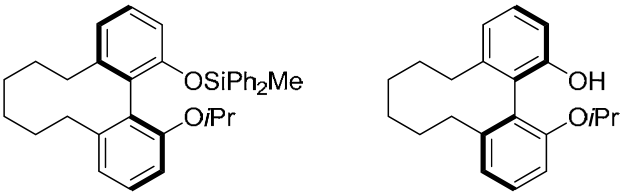

(*S*)-**3b'a** $C_{34}H_{38}O_2Si$ M = 506.76 g/mol

(*R*)-**1b'** $C_{21}H_{26}O_2$ M = 310.44 g/mol

Prepared according to **GP4** from *rac*-**1b'** (52.4 mg, 0.169 mmol, 1.00 equiv) and methyldiphenylsilane (**2a**, 22.5 mg, 0.118 mmol, 0.700 equiv). The catalyst system consisting of sodium *tert*-butanolate (0.8 mg, 8 µmol, 5 mol %), copper(I) chloride (0.8 mg, 8 µmol, 5 mol %) and (*R*,*R*)-Ph-BPE (5.1 mg, 10 µmol, 6.0 mol %) was scaled down accordingly. 4 Å molecular sieves were added to the catalyst mixture, and the reaction mixture was stirred for 8 h at room temperature. Preparative TLC (cyclohexane/ethyl acetate = 50:1) afforded silyl ether (*S*)-**3b'a** (40.4 mg, 79.7 µmol, 47%, 59% *ee*) as a sticky yellow resin and alcohol (*R*)-**1b'** (18.2 mg, 58.6 µmol, 35%, 86% *ee*) as a colorless solid.

Analytical data for silyl ether (*S*)-**3b'a**:

R_f = 0.37 (cyclohexane/ethyl acetate = 50:1). **^{1}H NMR** (500 MHz, CD_2Cl_2): δ/ppm = 7.39–7.31 (m, 5H), 7.29 (d, *J* = 7.2 Hz, 2H), 7.27–7.21 (m, 4H), 7.06 (t, *J* = 7.8 Hz, 1H), 6.95 (d, *J* = 7.7 Hz, 1H), 6.90 (d, *J* = 7.7 Hz, 1H), 6.83 (d, *J* = 8.2 Hz, 1H), 6.55 (d, *J* = 7.9 Hz, 1H), 4.37 (sept, *J* = 6.1 Hz, 1H), 2.54–2.46 (m, 2H), 2.45–2.37 (m, 2H), 1.68 (m_c, 2H), 1.39 (m_c, 2H), 1.19 (m_c, 2H), 1.12 (d, *J* = 6.1 Hz, 3H), 1.02 (d, *J* = 6.1 Hz, 3H), 0.73 (m_c, 2H), 0.61 (s, 3H). **^{13}C{^{1}H} NMR** (126 MHz, CD_2Cl_2): δ/ppm = 156.0, 152.6, 143.8, 143.7, 136.8, 136.5, 134.5 (2C), 134.5 (2C), 130.8, 130.1, 130.0, 129.6, 128.3, 128.1 (2C), 128.0 (2C), 127.9, 121.5, 121.2, 115.5, 111.5, 70.4, 29.5, 29.4, 29.1, 29.0, 22.4, 22.2, 21.5, 21.5, –2.7. **^{1}H/^{29}Si HMQC NMR** (500/99 MHz, CD_2Cl_2, optimized for *J* = 7 Hz): δ/ppm = (7.34, 7.24, 0.61)/–6.2. **HRMS** (APCI) calculated for $C_{28}H_{33}O_2Si^+$ [$(M–C_6H_5)^+$]: 429.2244; found: 429.2236. **IR** (ATR): ṽ/cm^{-1} = 3050, 2928, 2860, 1575, 1459, 1371, 1251, 1118, 1046, 1008, 939, 828, 795, 737. **Optical Rotation:** $[\alpha]_D^{20}$ = –11.5 (*c* 1.2, dichloromethane). The enantiomeric excess of **3b'a** was determined by HPLC analysis on a chiral stationary phase after cleavage of the silyl ether according to **GP5** (Daicel Chiralpak® IA column, column temperature: 20 °C, *n*-heptane/isopropanol = 90:10, flow rate: 0.60 mL/min, λ = 210 nm): t_R = 7.9 min (major), t_R = 15.8 min (minor).

Analytical data for alcohol (*R*)-**1b'**:

M.p. = 56–60 °C (dichloromethane). **R_f** = 0.11 (cyclohexane/ethyl acetate = 50:1). **^{1}H NMR** (500 MHz, $CDCl_3$): δ/ppm = 7.33 (t, *J* = 8.0 Hz, 1H), 7.22 (t, *J* = 7.9 Hz, 1H), 6.95 (d, *J* = 7.7 Hz, 1H), 6.84 (m_c, 2H), 6.79 (d, *J* = 7.7 Hz, 1H), 4.42 (s, 1H), 4.33 (sept, *J* = 6.0 Hz, 1H), 2.56–2.49 (m, 2H), 2.42 (dt, *J* = 13.3, 4.3 Hz, 1H), 2.29 (dt, *J* = 13.5, 4.3 Hz, 1H), 1.67 (m_c, 2H), 1.40 (m_c, 2H), 1.19 (m_c, 2H), 1.13 (d, *J* = 6.0 Hz, 3H), 1.06 (d, *J* = 6.0 Hz, 3H), 0.69 (m_c, 2H). **^{13}C{^{1}H} NMR** (126 MHz, $CDCl_3$): δ/ppm = 156.5, 152.6, 144.4, 142.7, 129.8, 128.6, 125.4, 124.8, 121.6, 120.3, 112.1, 111.5, 70.7, 29.3, 29.1, 28.8, 28.3, 22.1, 22.1, 21.2, 21.0. **HRMS** (APCI) calculated for $C_{21}H_{27}O_2^+$ [(M+H)$^+$]: 311.2006; found: 311.2000. **IR** (ATR): $\tilde{\nu}$/cm^{-1} = 3534, 2929, 2859, 1575, 1459, 1372, 1252, 1173, 1117, 1013. **Optical Rotation:** $[\alpha]_D^{20}$ = +35.1 (*c* 0.68, $CHCl_3$). The enantiomeric excess of **1b'** was determined by HPLC analysis on a chiral stationary phase (Daicel Chiralpak® IA column, column temperature: 20 °C, *n*-heptane/isopropanol = 90:10, flow rate: 0.60 mL/min, λ = 210 nm): t_R = 7.9 min (minor), t_R = 15.8 min (major).

5 Optimization Studies and Control Experiments

5.1. Screening of Reaction Conditions

Table S1. Optimization of Reaction Conditions.[a]

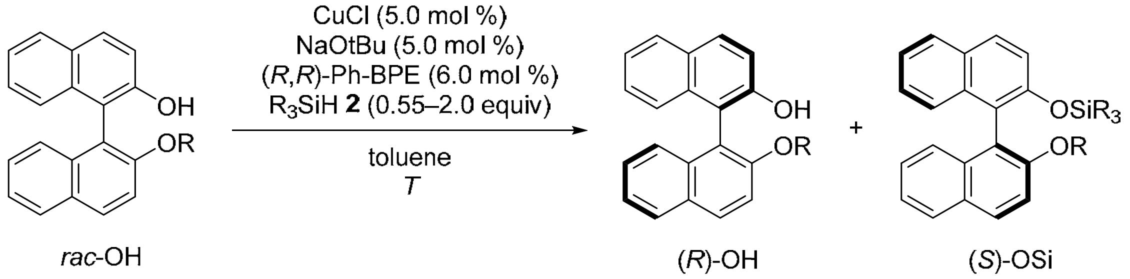

entry	R	R_3SiH, equiv	*T* [°C]	conversion [%][b]	time [h]	*ee*$_{OH}$ [%][c]	*ee*$_{OSi}$ [%][d]	*s*[e]
1[f]	H (**1a**)	$MePh_2SiH$ (**2a**), 0.70 equiv	rt	n.r.	–	–	–	–
2[g]	H (**1a**)	$MePh_2SiH$ (**2a**), 0.70 equiv	rt	n.d.	67	*rac*	*rac*	–
3	Me (**1b**)	$MePh_2SiH$ (**2a**), 0.55 equiv	rt	36	44	37	66	7.0
4	Me (**1b**)	Et_3SiH (**2b**), 0.55 equiv	50	n.r.	–	–	–	–
5	Me (**1b**)	Bn_3SiH (**2c**), 0.55 equiv	50	n.r.	–	–	–	–
6	Me (**1b**)	Me_2PhSiH (**2d**), 0.55 equiv	rt	12[h]	22	–	–	–
7	Me (**1b**)	Ph_3SiH (**2e**), 0.55 equiv	rt	n.r.	–	–	–	–
8	Me (**1b**)	Me[3,5-$(CF_3)_2C_6H_3]_2SiH$ (**2f**), 0.55 equiv	rt	35[i]	4	–	–	–
9	Me (**1b**)	Me(3,5-$Me_2C_6H_3)_2SiH$ (**2g**), 0.55 equiv	rt	9	74	8	73	6.8
10	*i*Pr (**1c**)	$MePh_2SiH$ (**2a**), 0.55 equiv	rt	38	75	47	77	12
11	*i*Pr (**1c**)	$MePh_2SiH$ (**2a**), 0.70 equiv	rt	40	48	55	82	17

12	*i*Pr (**1c**)	$MePh_2SiH$ (**2a**), 0.80 equiv	rt	44	29	62	79	16
13	*i*Pr (**1c**)	$MePh_2SiH$ (**2a**), 1.0 equiv	rt	46	27	64	77	14
14	*i*Pr (**1c**)	$MePh_2SiH$ (**2a**), 2.0 equiv	rt	64	24	94	52	11
15[j]	*i*Pr (**1c**)	$MePh_2SiH$ (**2a**), 0.70 equiv	rt	54	135	49	41	3.8
16[j]	*i*Pr (**1c**)	$MePh_2SiH$ (**2a**), 1.0 equiv	rt	52	42	61	57	6.6

[a]All reactions were performed on a 0.2 mmol scale according to **GP4**. [b]Conversion was monitored by ^{1}H NMR spectroscopy and calculated according to: conversion = $ee_{\text{unreacted alcohol}}/(ee_{\text{silyl ether}} + ee_{\text{unreacted alcohol}})$ multiplied by 100. [c]Determined by HPLC analysis on a chiral stationary phase. [d]Determined by HPLC analysis on a chiral stationary phase after cleavage of the silyl ether according to **GP5**. [e]s = ln[(1–C)(1–*ee*)]/ln[(1–C)(1+*ee*)], where *ee* = $ee_{\text{unreacted alcohol}}$/100 and C = conversion/100. [f]The alcohol *rac*-**1a** did not dissolve in toluene. [g]Reaction was perfomed in dichloromethane. [h]Complex reaction mixture. [i]Unstable silyl ether. [j]Instead of using CuCl and NaO*t*Bu, mesitylcopper(I) (5.0 mol %) was used as both the copper(I) source and the base. *n.r.* = no reaction. *n.d.* = not determined.

5.2. Investigation of the Silyl Ether Stability

For the silyl ether (*S*)-**3ba** from the reaction between 2'-methoxy-substituted phenol *rac*-**1b** and methyldiphenylsilane (**2a**), desilylation occurred already during storage at room temperature under air, and 70% of the deprotected alcohol were founf by ^{1}H NMR spectroscopy after two weeks. In the case of 2'-isopropoxy-substituted (*S*)-**3ca**, no desilylation was observed after this time, and also the silyl ethers of the other 2'-isopropoxy-substituted derivatives synthesized in this study did not show any signs of decomposition after storing for several months. However, desilylation was observed when a sample of **3ca** was subjected to the catalytic system consisting of CuCl, NaO*t*Bu, and (*R*,*R*)-Ph-BPE. After 22 hours, 12% of the deprotected alcohol **1c** were detected by ^{1}H NMR spectroscopy (Scheme S5, top). In the presence of an excess of phenol, the decomposition process was even more pronounced (68% deprotection after 22 h, Scheme S5, bottom). These results indicate that the formed silyl ether exhibits limited stability under the reaction conditions, and that the decomposition process might be competing with the forward reaction.

Scheme S5. Examination of silyl ether stability under the kinetic resolution conditions.

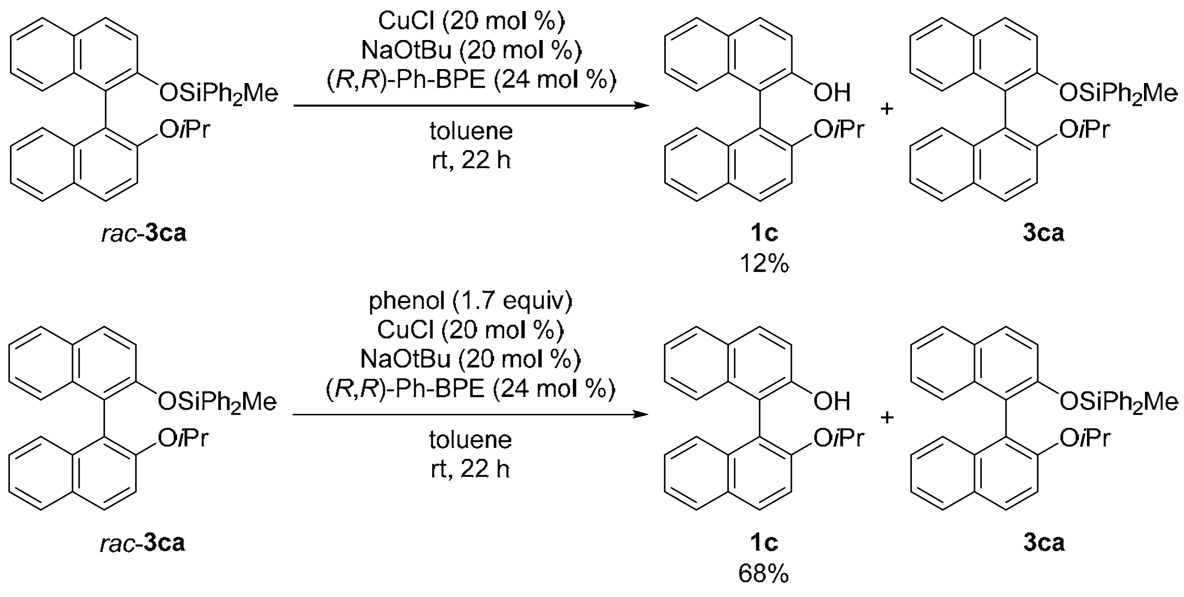

We then examined the effect on the stereochemical integrity of the desilylation reaction (Scheme S6), for which we treated the silyl ether **3ca** with our catalytic system using two samples: one with an initial enantiomeric excess of 77% from the scale-up reaction, and the other as a racemic mixture. In both cases, 14% of the desilylated alcohol **1c** were detected by ^{1}H NMR spectroscopy after 23 h. After separation by flash-column chromatography and deprotection of the silyl ether following **GP5**, HPLC analysis revealed no change in the enantiomeric excess in both cases. However, for the desilylated alcohol, slight variations in

the *ee* value were observed. For the enantioenriched (*S*)-**3ca**, the *ee* of the alcohol (*S*)-**1c** was measured at 72%, while for the racemic substrate, it was 3%.

Scheme S6. Examination of the stereochemical course of the desilylation reaction.

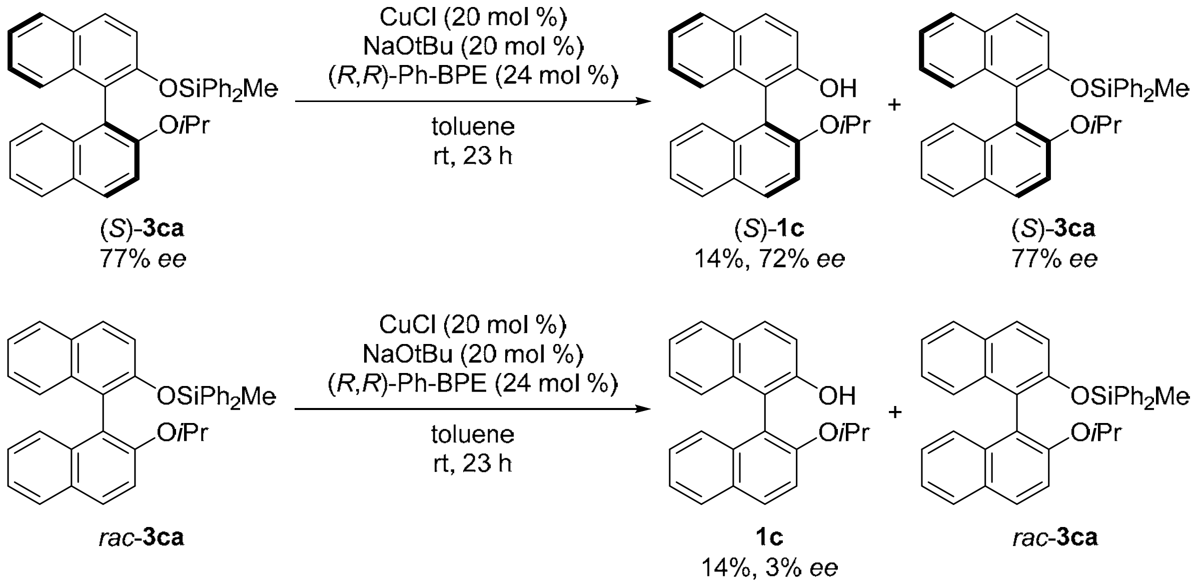

6 Scale-Up Experiment

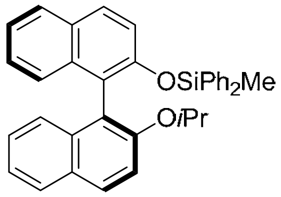

(*S*)-**3ca**
$C_{36}H_{32}O_2Si$
M = 524.74 g/mol

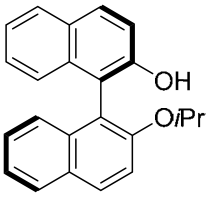

(*R*)-**1c**
$C_{23}H_{20}O_2$
M = 328.41 g/mol

In an argon-filled glovebox, an oven-dried 8 mL vial was charged with sodium *tert*-butanolate (4.8 mg, 50 µmol, 5.0 mol %), copper(I) chloride (5.0 mg, 50 µmol, 5.0 mol %), (*R*,*R*)-Ph-BPE (30.4 mg, 60.0 µmol, 6.00 mol %) and a magnetic stir bar. Toluene (0.5 mL) was added, and the resulting mixture was stirred for 15 min at room temperature. In a second oven-dried 8 mL vial, the alcohol *rac*-**1c** (328 mg, 1.00 mmol, 1.00 equiv) and methyldiphenylsilane (**2a**, 139 mg, 0.700 mmol, 0.700 equiv) were dissolved in toluene (4.5 mL). The solution containing *rac*-**1c** and the hydrosilane **2a** was added to the catalyst mixture, and the reaction was stirred for 49 h at room temperature and monitored with ^{1}H NMR spectroscopy. The reaction was quenched by filtration over silica, and flash column chromatography on silica gel using cyclohexane/ethyl acetate (100:0 → 80:1 → 40:1 → 20:1) afforded silyl ether (*S*)-**3ca** (232 mg, 0.442 mmol, 44%, 77% *ee*) and alcohol (*R*)-**1c** (173 mg, 0.527 mmol, 53%, 61% *ee*) as yellow solids.

The spectroscopic data of (*S*)-**3ca** and (*R*)-**1c** are consistent with those reported in section 4 for the small-scale reaction.

7 Removal of the Isopropyl-Group

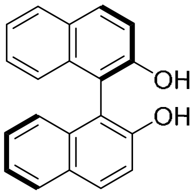

(*R*)-**1a**
$C_{20}H_{14}O_2$
M = 286.33 g/mol

In a dried Schlenk flask, the (*R*)-**1c** (0.200 mmol, 57.3 mg, 1.00 equiv, 61% *ee*) from the scale-up experiment was dissolved in dry dichloromethane (2 mL). The solution was cooled to 0 °C, and then boron trichloride (0.60 mL, 0.60 mmol, 3.00 equiv, 1.0 M in dichloromethane) was added dropwise *via* syringe. The solution was stirred for 17 h while slowly being warmed to room temperature. Then, water (3 mL) was added carefully, the phases were separated, and the aqueous phase was extracted with dichloromethane (4 x 3 mL). The combined organic phases were dried over Na_2SO_4 and the solvent was removed under reduced pressure. Filtration over a plug of silica using *tert*-butyl methyl ether as a solvent afforded the title compound (*R*)-**1a** (56.3 mg, 0.197 mmol, 99%, 64% *ee*) as a colorless solid.

^{1}H NMR (400 MHz, $CDCl_3$): δ/ppm = 7.97 (d, *J* = 8.9 Hz, 2H), 7.89 (d, *J* = 7.6 Hz, 2H), 7.41–7.35 (m, 4H), 7.31 (td, *J* = 7.0, 1.4 Hz, 2H), 7.16 (d, *J* = 8.3 Hz, 2H), 5.05 (s, br, 2H). **^{13}C{^{1}H} NMR** (101 MHz, $CDCl_3$): δ/ppm = 152.9 (2C), 133.5 (2C), 131.6 (2C), 129.6 (2C), 128.5 (2C), 127.6 (2C), 124.3 (2C), 124.2 (2C), 117.9 (2C), 111.0 (2C). **Optical Rotation:** $[\alpha]_D^{20}$ = +20.3 (*c* 0.90, THF).

The enantiomeric excess of **1a** was determined by HPLC analysis on a chiral stationary phase (Daicel Chiralpak® IA column, column temperature: 20 °C, *n*-heptane/isopropanol = 75:25, flow rate: 0.60 mL/min, λ = 230 nm): t_R = 19.9 min (major), t_R = 22.9 min (minor).

The NMR spectroscopic data are consistent with those reported in the literature.[19]

8 Determination of Absolute Configuration

The absolute configuration of the enantioenriched alcohols **1b** and **1c** was determined by comparison of the optical rotation value with literature values.[1] Additionally, the isopropoxy group in **1c** was cleaved (see section 7 for details) and the optical rotation value and the retention times on the chiral HPLC were compared with those of commercially obtained enantiopure (*R*)- and (*S*)-BINOL [(*R*)-**1a** and (*S*)-**1a**]. Similarly, the benzyloxy group in **1d** was removed following the same procedure, and the resulting product was analyzed *via* chiral HPLC. These analyses consistently pointed to enrichment of the (*R*)-alcohol. Based on these findings, the absolute configurations of the other compounds were assigned by analogy (with the exception of spirocyclic **1v**), assuming that the (*S*)-enantiomer undergoes silylation more rapidly in all cases.

9 HPLC Traces

(*S*)-[(2'-Methoxy-[1,1'-binaphthalen]-2-yl)oxy](methyl)diphenylsilane [(*S*)-3ba] and (*R*)-2'-Methoxy-[1,1'-binaphthalen]-2-ol [(*R*)-1b]

Figure S1. *rac*-2'-Methoxy-[1,1'-binaphthalen]-2-ol (*rac*-**1b**).

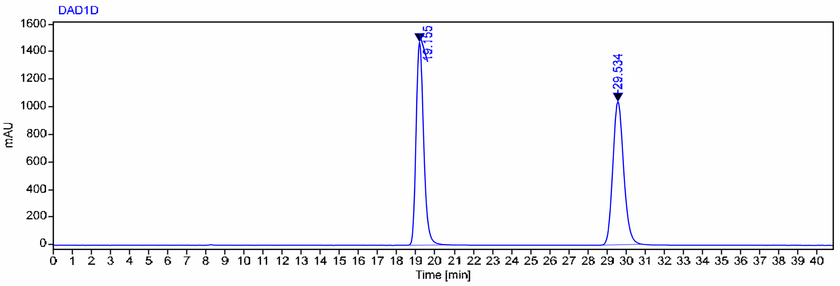

Signal: DAD1D

RT [min]	Width [min]	Area	Height	Area%
19.155	2.30	40404.05	1470.35	49.68
29.534	2.34	40919.50	1035.93	50.32
	**Sum**	**81323.55**		

Figure S2. (*S*)-2'-Methoxy-[1,1'-binaphthalen]-2-ol [(*S*)-**1b**, 45% *ee*].

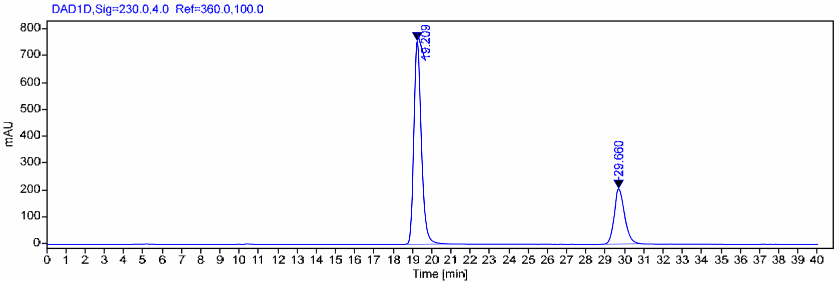

Signal: DAD1D,Sig=230.0,4.0 Ref=360.0,100.0

RT [min]	Width [min]	Area	Height	Area%
19.209	2.45	20350.06	755.37	72.71
29.660	1.98	7636.58	204.91	27.29
	**Sum**	**27986.64**		

Figure S3. (*R*)-2'-Methoxy-[1,1'-binaphthalen]-2-ol [(*R*)-**1b**, 67% *ee*].

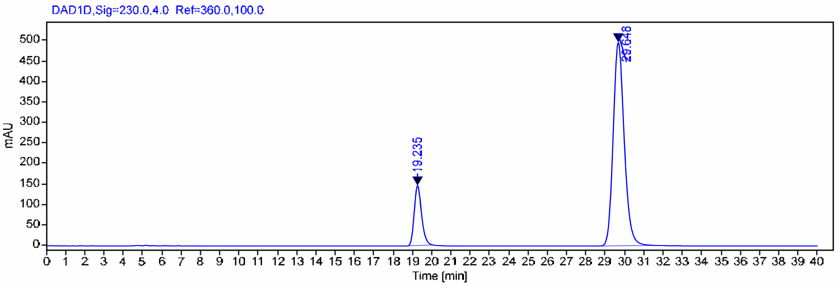

Signal: DAD1D,Sig=230.0,4.0 Ref=360.0,100.0

RT [min]	Width [min]	Area	Height	Area%
19.235	1.32	3865.43	146.03	16.36
29.648	2.85	19762.02	496.24	83.64
	**Sum**	**23627.45**		

(*S*)-[(2'-Isopropoxy-[1,1'-binaphthalen]-2-yl)oxy](methyl)diphenylsilane [(*S*)-3ca] and (*R*)-2'-Isopropoxy-[1,1'-binaphthalen]-2-ol [(*R*)-1c]

Figure S4. *rac*-2'-Isopropoxy-[1,1'-binaphthalen]-2-ol (*rac*-**1c**).

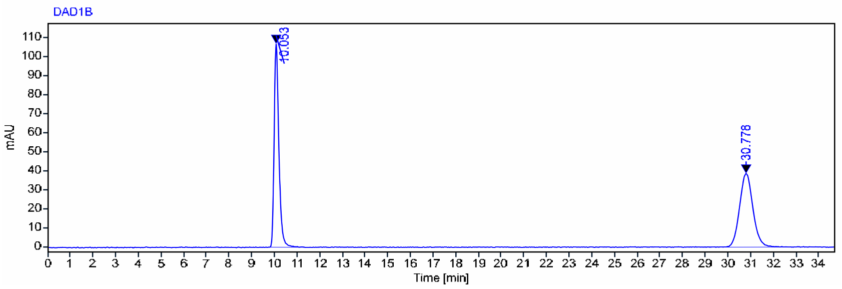

Signal:	DAD1B			
**RT [min]**	**Width [min]**	**Area**	**Height**	**Area%**
10.053	1.83	1544.02	106.74	49.36
30.778	3.16	1584.30	38.59	50.64
	**Sum**	**3128.32**		

Figure S5. (*S*)-2'-Isopropoxy-[1,1'-binaphthalen]-2-ol [(*S*)-**1c**, 82% *ee*].

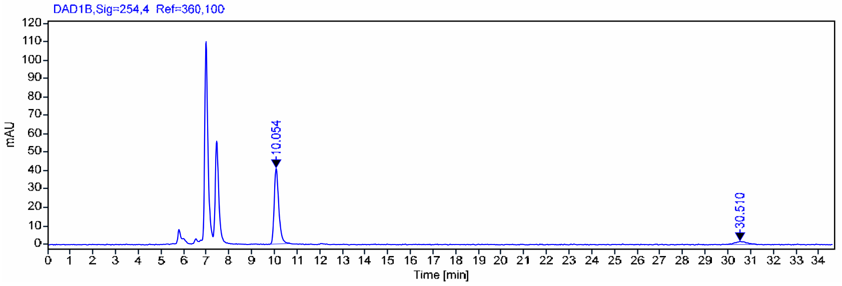

Signal:	DAD1B,Sig=254,4 Ref=360,100			
**RT [min]**	**Width [min]**	**Area**	**Height**	**Area%**
10.054	0.96	579.13	41.05	91.00
30.510	1.36	57.28	1.59	9.00
	**Sum**	**636.41**		

Figure S6. (*R*)-2'-Isopropoxy-[1,1'-binaphthalen]-2-ol [(*R*)-**1c**, 55% *ee*].

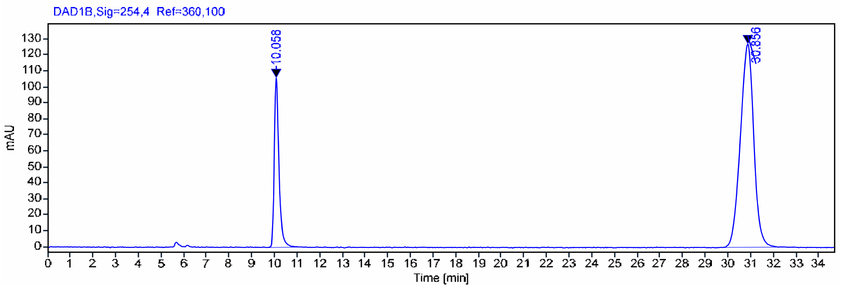

Signal:	DAD1B,Sig=254,4 Ref=360,100			
**RT [min]**	**Width [min]**	**Area**	**Height**	**Area%**
10.058	1.40	1529.45	105.86	22.60
30.856	2.61	5238.99	127.05	77.40
	**Sum**	**6768.44**		

(*S*)-[(2'-(Benzyloxy)-[1,1'-binaphthalen]-2-yl)oxy](methyl)diphenylsilane [(*S*)-3da] and (*R*)-2'-(Benzyloxy)-[1,1'-binaphthalen]-2-ol [(*R*)-1d]

Figure S7. *rac*-2'-(Benzyloxy)-[1,1'-binaphthalen]-2-ol (*rac*-**1d**).

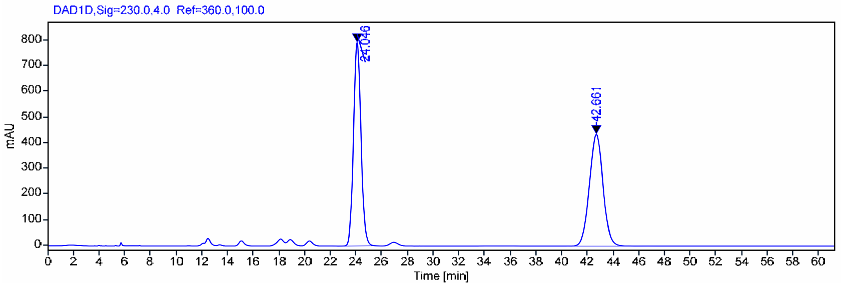

Signal:	DAD1D,Sig=230.0,4.0 Ref=360.0,100.0			
**RT [min]**	**Width [min]**	**Area**	**Height**	**Area%**
24.046	2.62	31971.98	791.18	49.94
42.661	4.58	32045.25	434.46	50.06
	**Sum**	**64017.23**		

Figure S8. (*S*)-2'-(Benzyloxy)-[1,1'-binaphthalen]-2-ol [(*S*)-**1d**, 49% *ee*].

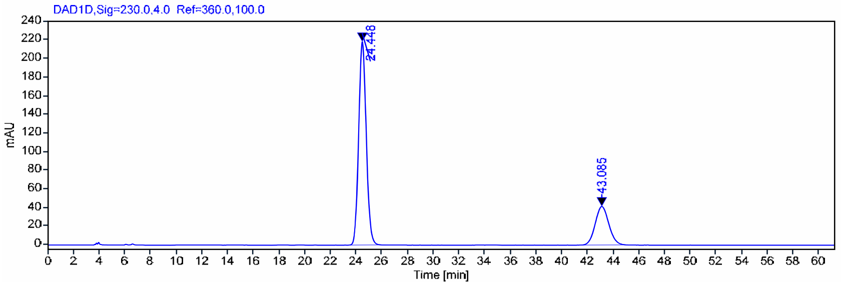

Signal:	DAD1D,Sig=230.0,4.0 Ref=360.0,100.0			
**RT [min]**	**Width [min]**	**Area**	**Height**	**Area%**
24.448	2.57	8937.32	218.43	74.55
43.085	3.63	3051.03	41.42	25.45
	**Sum**	**11988.35**		

Figure S9. (*R*)-2'-(Benzyloxy)-[1,1'-binaphthalen]-2-ol [(*R*)-**1d**, 43% *ee*].

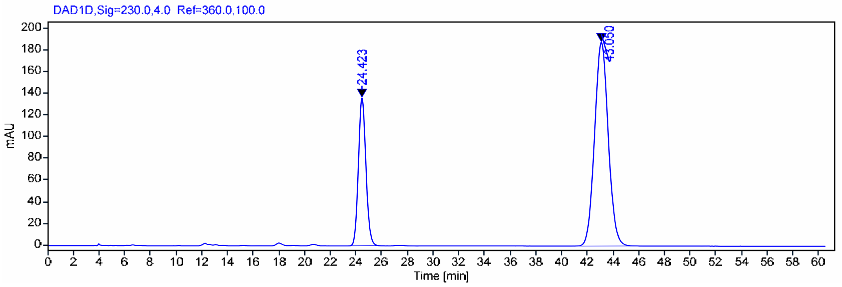

Signal:	DAD1D,Sig=230.0,4.0 Ref=360.0,100.0			
**RT [min]**	**Width [min]**	**Area**	**Height**	**Area%**
24.423	2.31	5539.70	136.32	28.49
43.050	4.64	13907.41	187.76	71.51
	**Sum**	**19447.11**		

(*S*)-[(2'-Isopropoxy-5,5',6,6',7,7',8,8'-octahydro-[1,1'-binaphthalen]-2-yl)oxy](methyl)diphenylsilane [(*S*)-3ka] and (*R*)-2'-Isopropoxy-5,5',6,6',7,7',8,8'-octahydro-[1,1'-binaphthalen]-2-ol [(*R*)-1k]

Figure S10. *rac*-2'-Isopropoxy-5,5',6,6',7,7',8,8'-octahydro-[1,1'-binaphthalen]-2-ol (*rac*-**1k**).

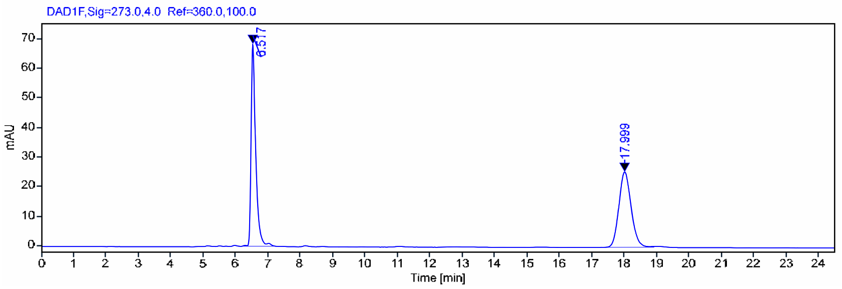

Signal:	DAD1F,Sig=273.0,4.0 Ref=360.0,100.0			
**RT [min]**	**Width [min]**	**Area**	**Height**	**Area%**
6.517	1.01	675.79	68.21	50.23
17.999	1.53	669.50	25.44	49.77
	**Sum**	**1345.28**		

Figure S11. (*S*)-2'-Isopropoxy-5,5',6,6',7,7',8,8'-octahydro-[1,1'-binaphthalen]-2-ol [(*S*)-**1k**, 50% *ee*].

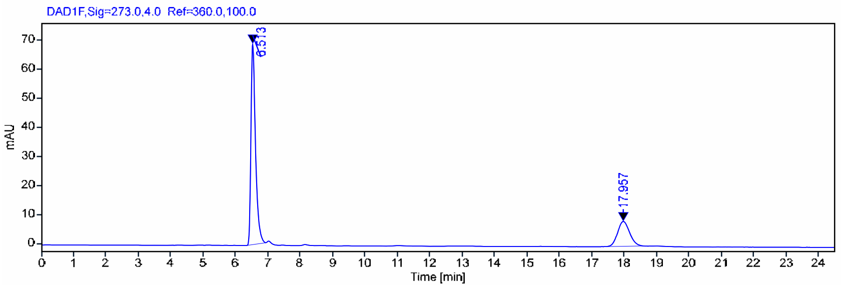

Signal:	DAD1F,Sig=273.0,4.0 Ref=360.0,100.0			
**RT [min]**	**Width [min]**	**Area**	**Height**	**Area%**
6.513	0.59	662.11	68.88	75.16
17.957	1.13	218.78	8.53	24.84
	**Sum**	**880.89**		

Figure S12. (*R*)-2'-Isopropoxy-5,5',6,6',7,7',8,8'-octahydro-[1,1'-binaphthalen]-2-ol [(*R*)-**1k**, >99% *ee*].

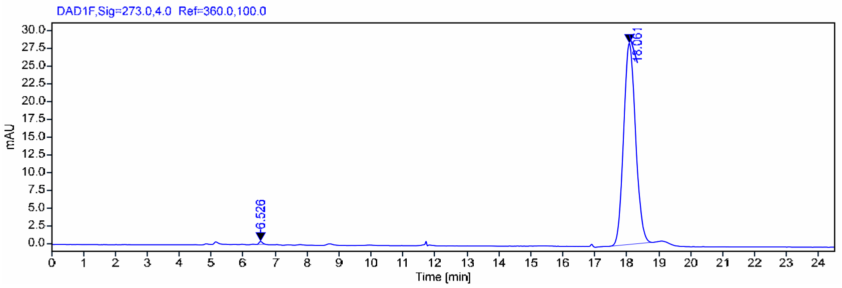

Signal:	DAD1F,Sig=273.0,4.0 Ref=360.0,100.0			
**RT [min]**	**Width [min]**	**Area**	**Height**	**Area%**
6.526	0.27	3.05	0.40	0.42
18.061	1.33	730.69	28.30	99.58
	**Sum**	**733.74**		

(*S*)-[(2'-Isopropoxy-4,4'-dimethyl-[1,1'-binaphthalen]-2-yl)oxy](methyl)diphenylsilane [(*S*)-3la] and (*R*)-2'-Isopropoxy-4,4'-dimethyl-[1,1'-binaphthalen]-2-ol [(*R*)-1l]

Figure S13. *rac*-2'-Isopropoxy-4,4'-dimethyl-[1,1'-binaphthalen]-2-ol (*rac*-**1l**).

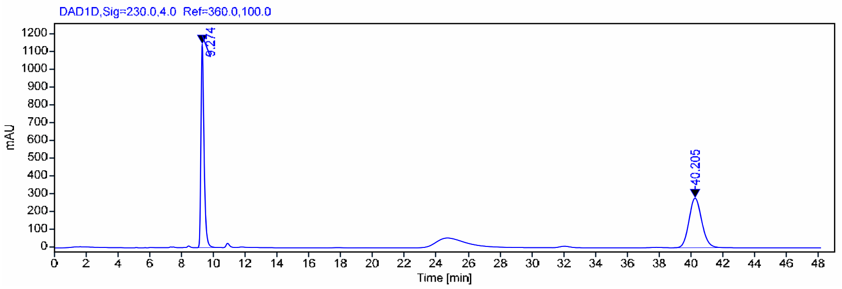

Signal: DAD1D,Sig=230.0,4.0 Ref=360.0,100.0

RT [min]	Width [min]	Area	Height	Area%
9.274	1.30	15335.52	1143.05	49.92
40.205	3.34	15384.38	278.01	50.08
	**Sum**	**30719.90**		

Figure S14. (*S*)-2'-Isopropoxy-4,4'-dimethyl-[1,1'-binaphthalen]-2-ol [(*S*)-**1l**, 76% *ee*].

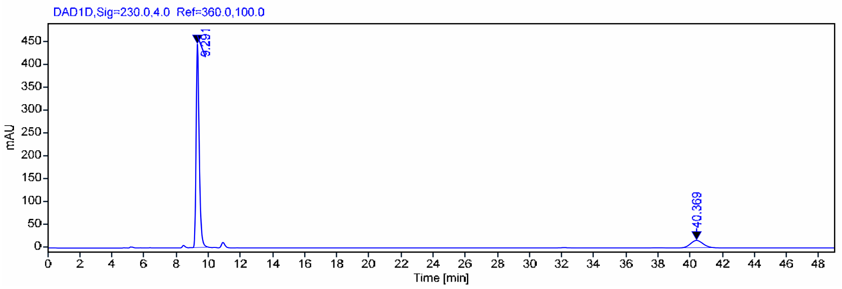

Signal: DAD1D,Sig=230.0,4.0 Ref=360.0,100.0

RT [min]	Width [min]	Area	Height	Area%
9.291	0.95	5927.82	443.94	87.78
40.369	1.97	825.04	15.78	12.22
	**Sum**	**6752.86**		

Figure S15. (*R*)-2'-Isopropoxy-4,4'-dimethyl-[1,1'-binaphthalen]-2-ol [(*R*)-**1l**, 53% *ee*].

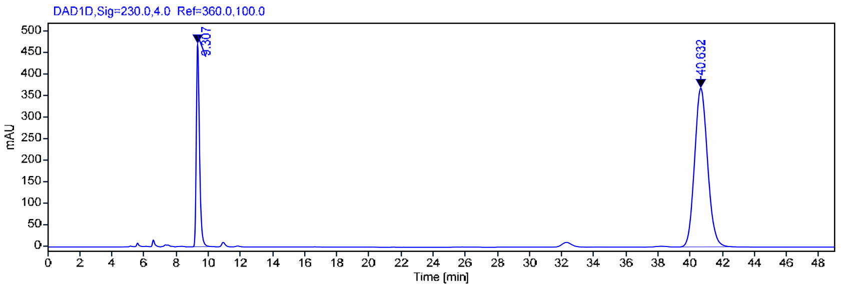

Signal: DAD1D,Sig=230.0,4.0 Ref=360.0,100.0

RT [min]	Width [min]	Area	Height	Area%
9.307	1.07	6352.91	470.27	23.54
40.632	3.51	20631.60	367.78	76.46
	**Sum**	**26984.51**		

(*S*)-[(2'-Isopropoxy-7,7'-dimethyl-[1,1'-binaphthalen]-2-yl)oxy](methyl)diphenylsilane [(*S*)-3oa] and (*R*)-2'-Isopropoxy-7,7'-dimethyl-[1,1'-binaphthalen]-2-ol [(*R*)-1o]

Figure S16. *rac*-2'-Isopropoxy-7,7'-dimethyl-[1,1'-binaphthalen]-2-ol (*rac*-**1o**).

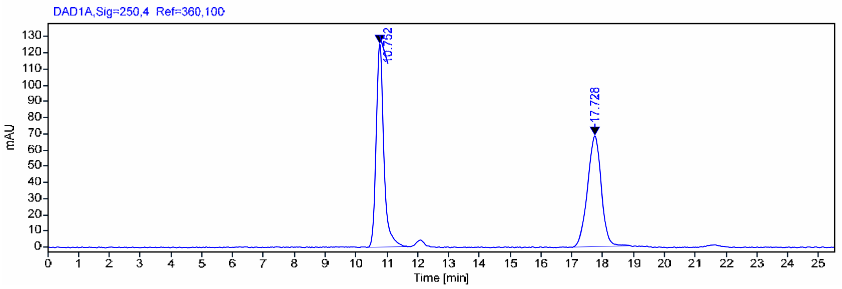

**Signal:**	DAD1A,Sig=250,4 Ref=360,100			
**RT [min]**	**Width [min]**	**Area**	**Height**	**Area%**
10.752	1.36	2159.80	125.53	50.39
17.728	1.96	2126.67	68.47	49.61
	**Sum**	**4286.47**		

Figure S17. (*S*)-2'-Isopropoxy-7,7'-dimethyl-[1,1'-binaphthalen]-2-ol [(*S*)-**1o**, 58% *ee*].

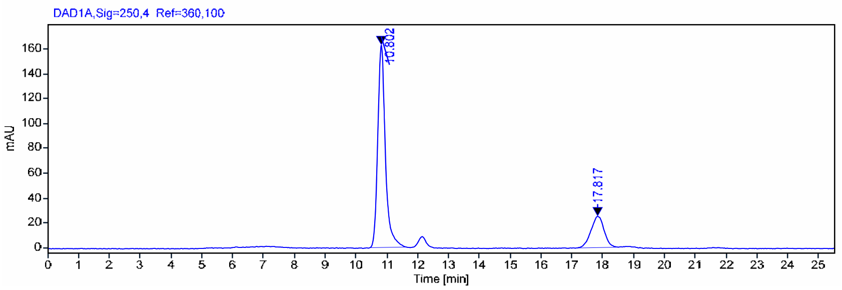

**Signal:**	DAD1A,Sig=250,4 Ref=360,100			
**RT [min]**	**Width [min]**	**Area**	**Height**	**Area%**
10.802	1.16	2783.98	162.32	78.93
17.817	1.34	743.30	25.01	21.07
	**Sum**	**3527.28**		

Figure S18. (*R*)-2'-Isopropoxy-7,7'-dimethyl-[1,1'-binaphthalen]-2-ol [(*R*)-**1o**, 47% *ee*].

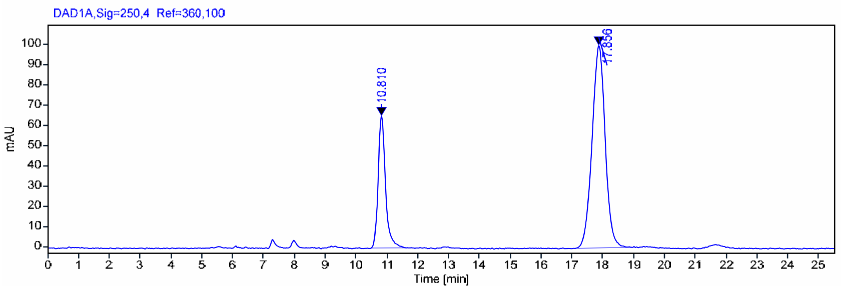

**Signal:**	DAD1A,Sig=250,4 Ref=360,100			
**RT [min]**	**Width [min]**	**Area**	**Height**	**Area%**
10.810	1.15	1073.21	64.78	26.72
17.856	1.88	2942.87	99.42	73.28
	**Sum**	**4016.08**		

(*S*)-[(2'-Isopropoxy-6,6'-dimethyl-[1,1'-binaphthalen]-2-yl)oxy](methyl)diphenylsilane [(*S*)-3qa] and (*R*)-2'-Isopropoxy-6,6'-dimethyl-[1,1'-binaphthalen]-2-ol [(*R*)-1q]

Figure S19. *rac*-2'-Isopropoxy-6,6'-dimethyl-[1,1'-binaphthalen]-2-ol (*rac*-**1q**).

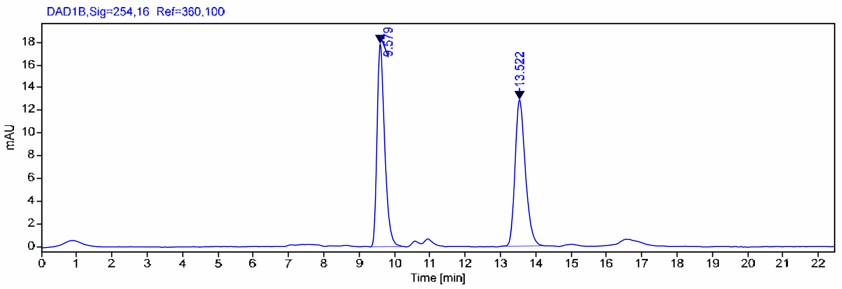

Signal:	DAD1B,Sig=254,16 Ref=360,100			
**RT [min]**	**Width [min]**	**Area**	**Height**	**Area%**
9.579	0.98	267.58	17.88	50.58
13.522	1.31	261.41	12.88	49.42
	**Sum**	**528.99**		

Figure S20. (*S*)-2'-Isopropoxy-6,6'-dimethyl-[1,1'-binaphthalen]-2-ol [(*S*)-**1q**, 75% *ee*].

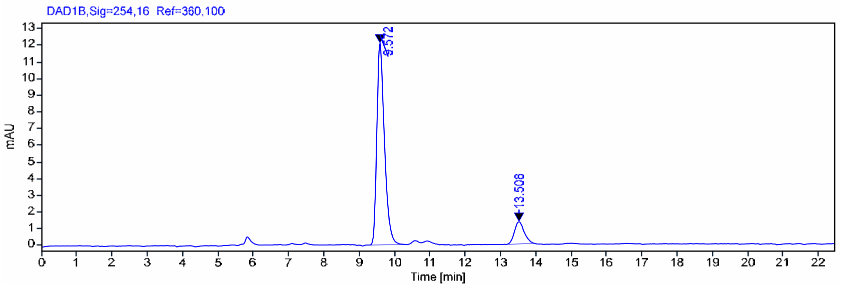

Signal:	DAD1B,Sig=254,16 Ref=360,100			
**RT [min]**	**Width [min]**	**Area**	**Height**	**Area%**
9.572	1.02	180.49	12.07	87.66
13.508	0.80	25.40	1.32	12.34
	**Sum**	**205.90**		

Figure S21. (*R*)-2'-Isopropoxy-6,6'-dimethyl-[1,1'-binaphthalen]-2-ol [(*R*)-**1q**, 81% *ee*].

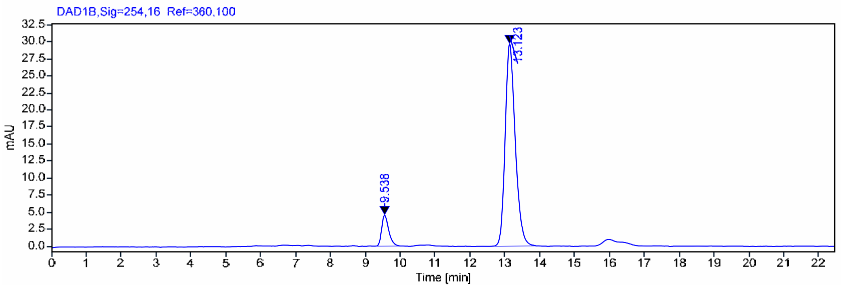

Signal:	DAD1B,Sig=254,16 Ref=360,100			
**RT [min]**	**Width [min]**	**Area**	**Height**	**Area%**
9.538	0.71	63.57	4.50	9.65
13.123	1.30	595.48	29.56	90.35
	**Sum**	**659.05**		

(*S*)-[(2'-Isopropoxy-6,6'-diisopropyl-[1,1'-binaphthalen]-2-yl)oxy](methyl)diphenyl-silane [(*S*)-3ra] and (*R*)-2'-Isopropoxy-6,6'-diisopropyl-[1,1'-binaphthalen]-2-ol [(*R*)-1r]

Figure S22. *rac*-2'-Isopropoxy-6,6'-diisopropyl-[1,1'-binaphthalen]-2-ol (*rac*-**1r**).

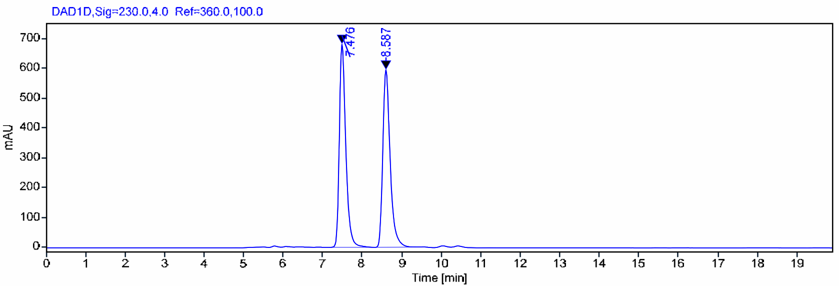

Signal:	DAD1D,Sig=230.0,4.0 Ref=360.0,100.0			
**RT [min]**	**Width [min]**	**Area**	**Height**	**Area%**
7.476	1.10	7757.52	683.05	50.27
8.587	1.08	7673.15	596.42	49.73
	**Sum**	**15430.67**		

Figure S23. (*S*)-2'-Isopropoxy-6,6'-diisopropyl-[1,1'-binaphthalen]-2-ol [(*S*)-**1r**, 74% *ee*].

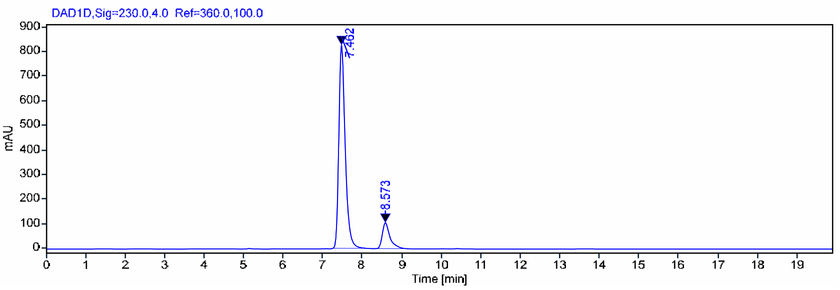

Signal:	DAD1D,Sig=230.0,4.0 Ref=360.0,100.0			
**RT [min]**	**Width [min]**	**Area**	**Height**	**Area%**
7.462	0.85	9258.81	825.09	86.75
8.573	0.76	1413.85	105.09	13.25
	**Sum**	**10672.66**		

Figure S24. (*R*)-2'-Isopropoxy-6,6'-diisopropyl-[1,1'-binaphthalen]-2-ol [(*R*)-**1r**, 67% *ee*].

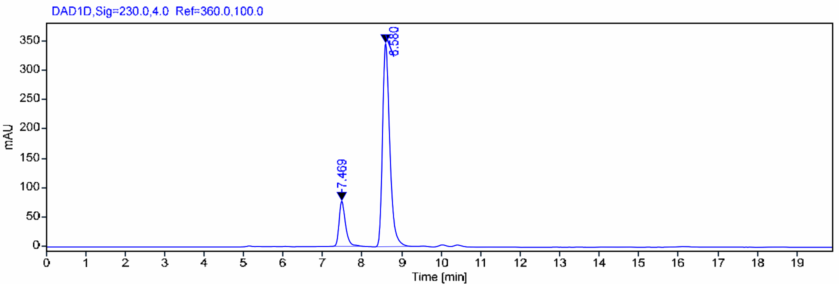

Signal:	DAD1D,Sig=230.0,4.0 Ref=360.0,100.0			
**RT [min]**	**Width [min]**	**Area**	**Height**	**Area%**
7.469	0.81	872.95	77.00	16.52
8.580	0.93	4411.88	346.35	83.48
	**Sum**	**5284.83**		

(*S*)-[(2'-Isopropoxy-6,6'-diphenyl-[1,1'-binaphthalen]-2-yl)oxy](methyl)diphenylsilane [(*S*)-3sa] and (*R*)-2'-Isopropoxy-6,6'-diphenyl-[1,1'-binaphthalen]-2-ol [(*R*)-1s]

Figure S25. *rac*-2'-Isopropoxy-6,6'-diphenyl-[1,1'-binaphthalen]-2-ol (*rac*-**1s**).

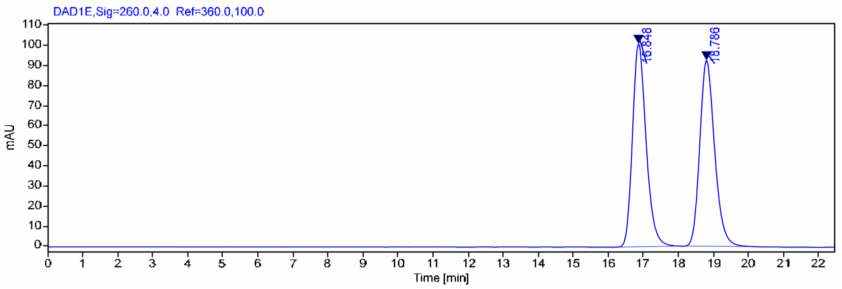

Signal: DAD1E,Sig=260.0,4.0 Ref=360.0,100.0

RT [min]	Width [min]	Area	Height	Area%
16.848	1.84	2739.84	101.03	50.08
18.786	1.90	2730.92	92.49	49.92
	**Sum**	**5470.76**		

Figure S26. (*S*)-2'-Isopropoxy-6,6'-diphenyl-[1,1'-binaphthalen]-2-ol [(*S*)-**1s**, 62% *ee*].

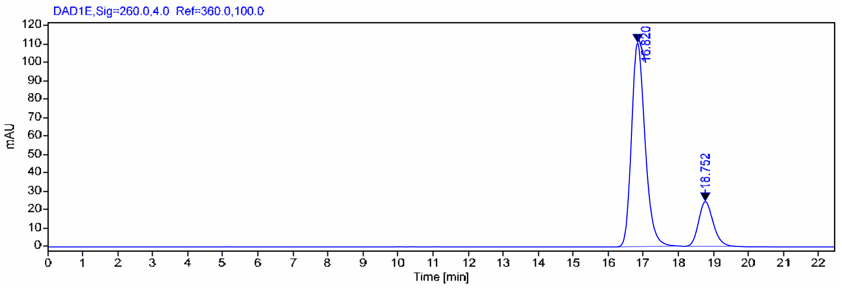

Signal: DAD1E,Sig=260.0,4.0 Ref=360.0,100.0

RT [min]	Width [min]	Area	Height	Area%
16.820	1.73	2979.41	110.68	80.97
18.752	1.42	700.41	24.24	19.03
	**Sum**	**3679.82**		

Figure S27. (*R*)-2'-Isopropoxy-6,6'-diphenyl-[1,1'-binaphthalen]-2-ol [(*R*)-**1s**, 43% *ee*].

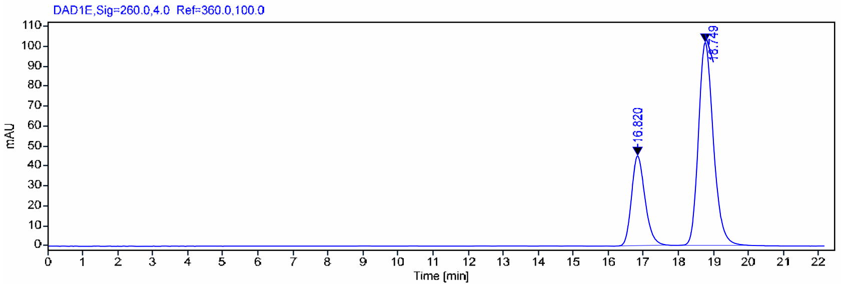

Signal: DAD1E,Sig=260.0,4.0 Ref=360.0,100.0

RT [min]	Width [min]	Area	Height	Area%
16.820	1.38	1198.74	44.91	28.56
18.749	2.01	2998.56	101.64	71.44
	**Sum**	**4197.30**		

ent-[(7'-Isopropoxy-2,2',3,3'-tetrahydro-1,1'-spirobi[inden]-7-yl)oxy](methyl)diphenyl-silane (*ent*-3va) and *ent*-7'-Isopropoxy-2,2',3,3'-tetrahydro-1,1'-spirobi[inden]-7-ol (*ent*-1v)

Figure S28. *rac*-7'-Isopropoxy-2,2',3,3'-tetrahydro-1,1'-spirobi[inden]-7-ol (*rac*-**1v**).

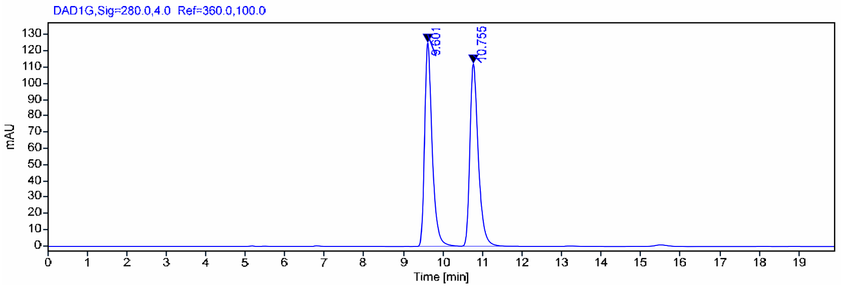

Signal:	DAD1G,Sig=280.0,4.0 Ref=360.0,100.0			
**RT [min]**	**Width [min]**	**Area**	**Height**	**Area%**
9.601	1.13	1659.79	125.24	50.03
10.755	1.23	1657.59	112.30	49.97
	**Sum**	**3317.38**		

Figure S29. *ent*-7'-Isopropoxy-2,2',3,3'-tetrahydro-1,1'-spirobi[inden]-7-ol (*ent*-**1v**, 30% *ee*).

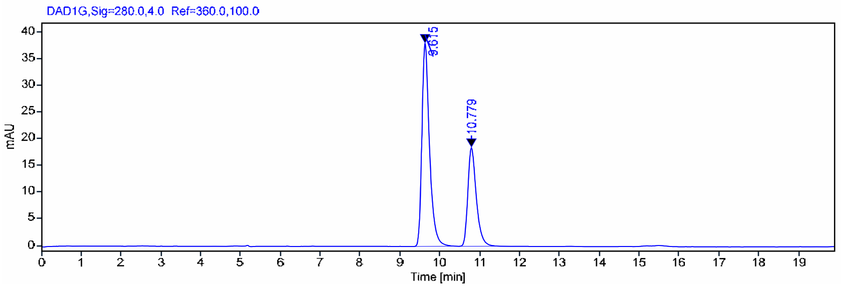

Signal:	DAD1G,Sig=280.0,4.0 Ref=360.0,100.0			
**RT [min]**	**Width [min]**	**Area**	**Height**	**Area%**
9.615	1.02	503.65	37.91	65.11
10.779	0.97	269.86	18.42	34.89
	**Sum**	**773.51**		

Figure S30. *ent*-7'-Isopropoxy-2,2',3,3'-tetrahydro-1,1'-spirobi[inden]-7-ol (*ent*-**1v**, 48% *ee*).

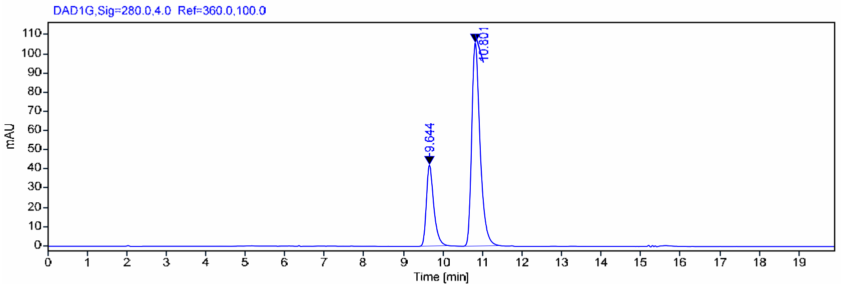

Signal:	DAD1G,Sig=280.0,4.0 Ref=360.0,100.0			
**RT [min]**	**Width [min]**	**Area**	**Height**	**Area%**
9.644	0.75	550.53	42.16	26.17
10.801	0.92	1552.98	105.84	73.83
	**Sum**	**2103.52**		

(*S*)-[(2'-Isopropoxy-6,6'-dimethyl-[1,1'-biphenyl]-2-yl)oxy](methyl)diphenylsilane [(*S*)-3wa] and (*R*)-2'-Isopropoxy-6,6'-dimethyl-[1,1'-biphenyl]-2-ol [(*R*)-1w]

Figure S31. *rac*-2'-Isopropoxy-6,6'-dimethyl-[1,1'-biphenyl]-2-ol (*rac*-**1w**).

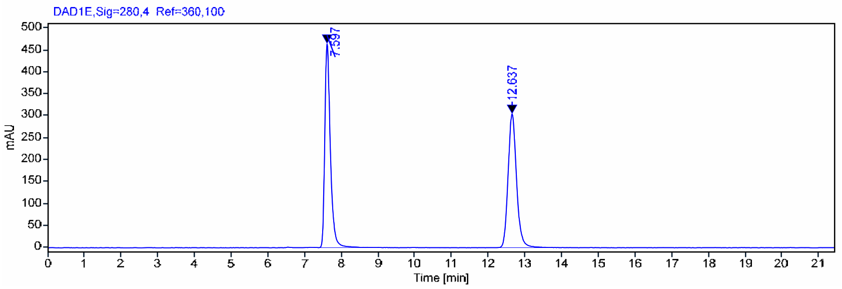

Signal:	DAD1E,Sig=280,4 Ref=360,100			
**RT [min]**	**Width [min]**	**Area**	**Height**	**Area%**
7.597	1.48	4880.40	464.63	49.94
12.637	1.74	4892.01	304.95	50.06
	**Sum**	**9772.41**		

Figure S32. (*S*)-2'-Isopropoxy-6,6'-dimethyl-[1,1'-biphenyl]-2-ol [(*S*)-**1w**, 84% *ee*].

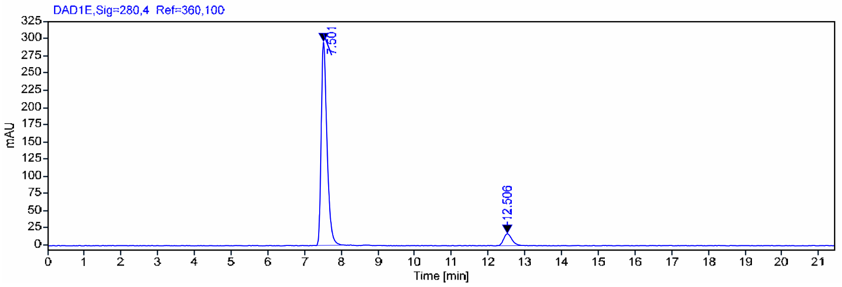

Signal:	DAD1E,Sig=280,4 Ref=360,100			
**RT [min]**	**Width [min]**	**Area**	**Height**	**Area%**
7.501	0.84	3241.46	295.61	92.20
12.506	0.79	274.12	17.15	7.80
	**Sum**	**3515.58**		

Figure S33. (*R*)-2'-Isopropoxy-6,6'-dimethyl-[1,1'-biphenyl]-2-ol [(*R*)-**1w**, 79% *ee*].

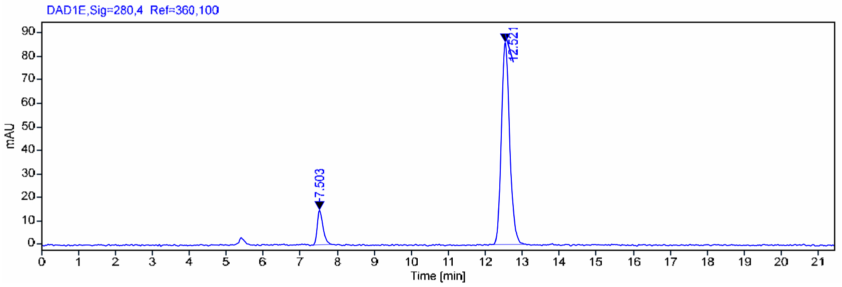

Signal:	DAD1E,Sig=280,4 Ref=360,100			
**RT [min]**	**Width [min]**	**Area**	**Height**	**Area%**
7.503	0.52	157.20	14.64	10.48
12.521	0.95	1342.56	85.85	89.52
	**Sum**	**1499.76**		

(*S*)-[(2',6-Diethyl-6'-isopropoxy-[1,1'-biphenyl]-2-yl)oxy](methyl)diphenylsilane [(*S*)-3xa] and (*R*)-2',6-Diethyl-6'-isopropoxy-[1,1'-biphenyl]-2-ol [(*R*)-1x]

Figure S34. *rac*-2',6-Diethyl-6'-isopropoxy-[1,1'-biphenyl]-2-ol (*rac*-**1x**).

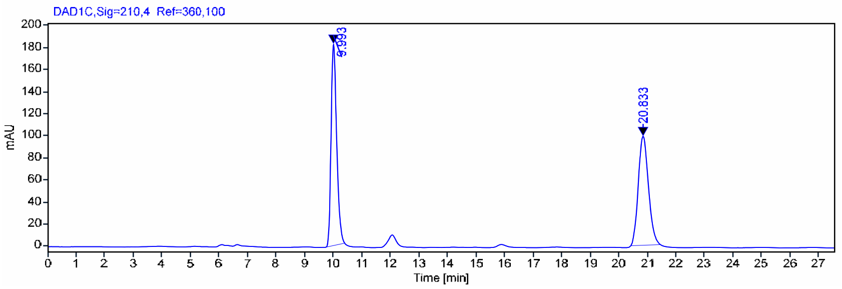

Signal:	DAD1C,Sig=210,4 Ref=360,100			
**RT [min]**	**Width [min]**	**Area**	**Height**	**Area%**
9.993	0.69	2474.95	182.81	50.38
20.833	1.03	2437.86	98.92	49.62
	**Sum**	**4912.81**		

Figure S35. (*S*)-2',6-Diethyl-6'-isopropoxy-[1,1'-biphenyl]-2-ol [(*S*)-**1x**, 86% *ee*].

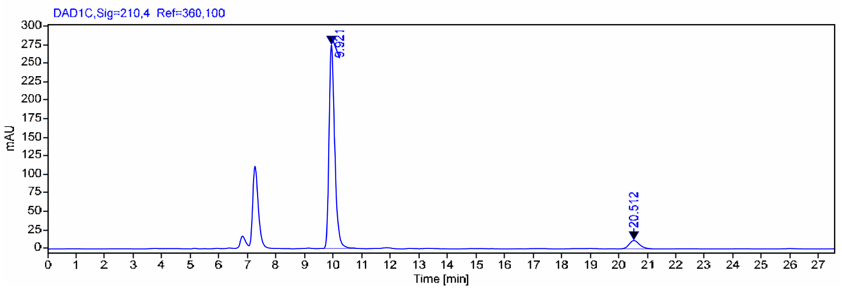

Signal:	DAD1C,Sig=210,4 Ref=360,100			
**RT [min]**	**Width [min]**	**Area**	**Height**	**Area%**
9.921	1.26	3789.81	274.71	93.00
20.512	1.22	285.15	11.27	7.00
	**Sum**	**4074.96**		

Figure S36. (*R*)-2',6-Diethyl-6'-isopropoxy-[1,1'-biphenyl]-2-ol [(*R*)-**1x**, 83% *ee*].

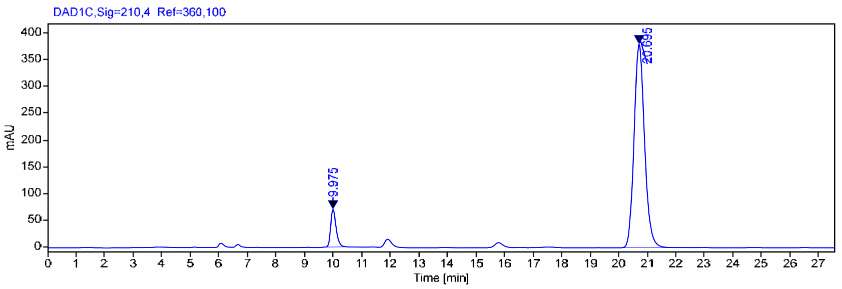

Signal:	DAD1C,Sig=210,4 Ref=360,100			
**RT [min]**	**Width [min]**	**Area**	**Height**	**Area%**
9.975	0.78	925.07	70.05	8.68
20.695	1.97	9734.11	380.15	91.32
	**Sum**	**10659.19**		

(*S*)-[(2',6-Diisobutyl-6'-isopropoxy-[1,1'-biphenyl]-2-yl)oxy](methyl)diphenylsilane [(*S*)-3ya] and (*R*)-2',6-Diisobutyl-6'-isopropoxy-[1,1'-biphenyl]-2-ol [(*R*)-1y]

Figure S37. *rac*-2',6-Diisobutyl-6'-isopropoxy-[1,1'-biphenyl]-2-ol (*rac*-**1y**).

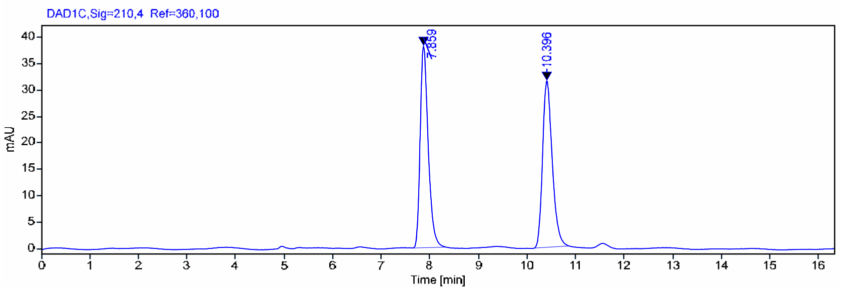

Signal:	DAD1C,Sig=210,4 Ref=360,100			
**RT [min]**	**Width [min]**	**Area**	**Height**	**Area%**
7.859	0.70	437.45	38.19	50.14
10.396	0.71	435.06	31.50	49.86
	**Sum**	**872.51**		

Figure S38. (*S*)-2',6-Diisobutyl-6'-isopropoxy-[1,1'-biphenyl]-2-ol [(*S*)-**1y**, 83% *ee*].

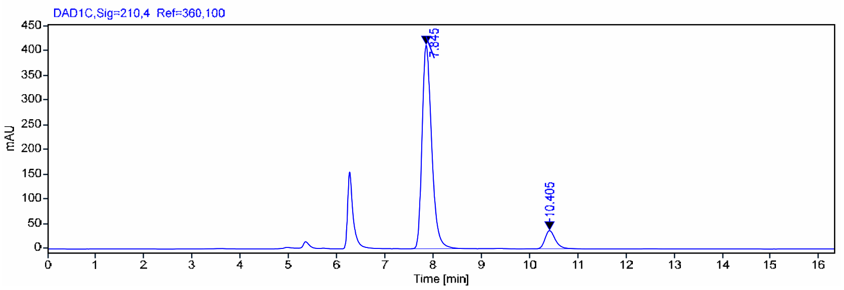

Signal:	DAD1C,Sig=210,4 Ref=360,100			
**RT [min]**	**Width [min]**	**Area**	**Height**	**Area%**
7.845	1.10	5684.35	411.26	91.28
10.405	0.89	543.23	36.69	8.72
	**Sum**	**6227.58**		

Figure S39. (*R*)-2',6-Diisobutyl-6'-isopropoxy-[1,1'-biphenyl]-2-ol [(*R*)-**1y**, 95% *ee*].

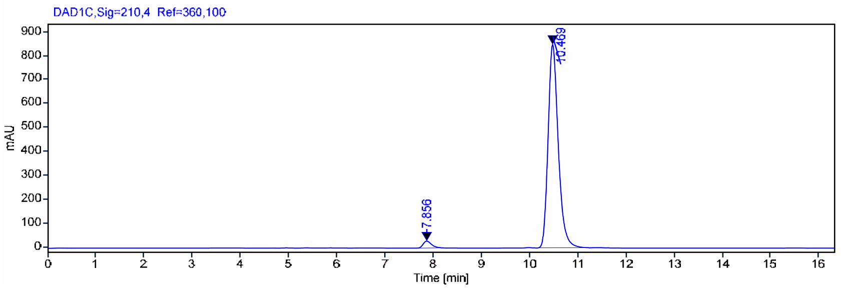

Signal:	DAD1C,Sig=210,4 Ref=360,100			
**RT [min]**	**Width [min]**	**Area**	**Height**	**Area%**
7.856	0.59	348.45	28.69	2.74
10.469	1.03	12366.83	846.47	97.26
	**Sum**	**12715.27**		

(*S*)-[(6'-Isopropoxy-2',3',5,6-tetramethyl-[1,1'-biphenyl]-2-yl)oxy](methyl)diphenylsilane [(*S*)-3za] and (*R*)-6'-Isopropoxy-2',3',5,6-tetramethyl-[1,1'-biphenyl]-2-ol [(*R*)-1z]

Figure S40. *rac*-6'-Isopropoxy-2',3',5,6-tetramethyl-[1,1'-biphenyl]-2-ol (*rac*-**1z**).

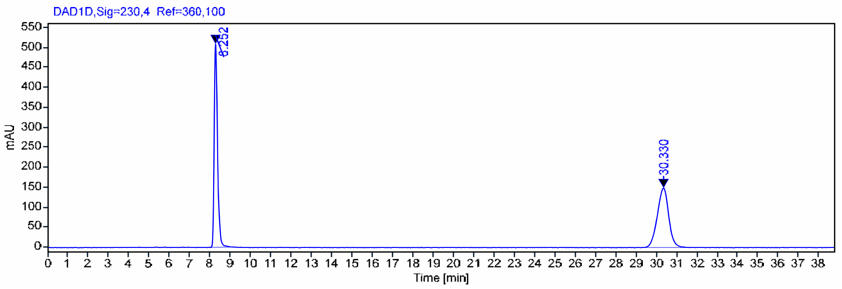

Signal:	DAD1D,Sig=230,4 Ref=360,100			
**RT [min]**	**Width [min]**	**Area**	**Height**	**Area%**
8.252	1.16	5730.67	508.89	49.68
30.330	2.54	5803.85	148.94	50.32
	**Sum**	**11534.52**		

Figure S41. (*S*)-6'-Isopropoxy-2',3',5,6-tetramethyl-[1,1'-biphenyl]-2-ol [(*S*)-**1z**, 53% *ee*].

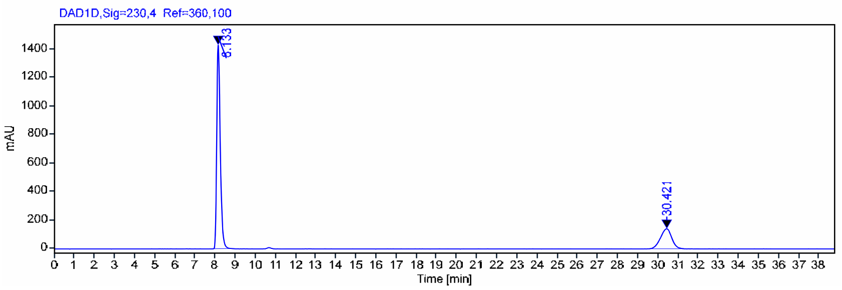

Signal:	DAD1D,Sig=230,4 Ref=360,100			
**RT [min]**	**Width [min]**	**Area**	**Height**	**Area%**
8.133	0.97	17464.55	1428.20	76.40
30.421	1.74	5393.50	139.77	23.60
	**Sum**	**22858.05**		

Figure S42. (*R*)-6'-Isopropoxy-2',3',5,6-tetramethyl-[1,1'-biphenyl]-2-ol [(*R*)-**1z**, 92% *ee*].

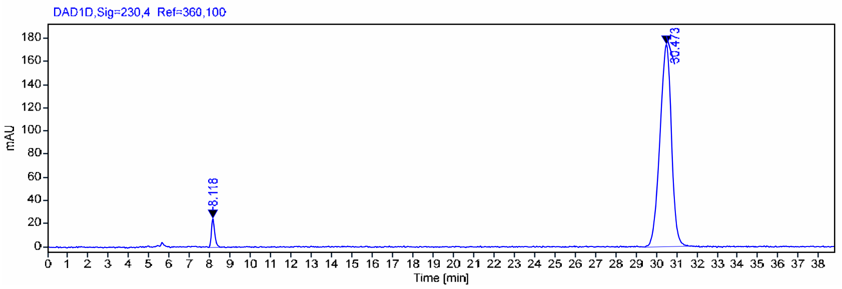

Signal:	DAD1D,Sig=230,4 Ref=360,100			
**RT [min]**	**Width [min]**	**Area**	**Height**	**Area%**
8.118	0.55	285.26	24.64	3.98
30.473	2.42	6890.89	174.26	96.02
	**Sum**	**7176.15**		

(*S*)-[(2'-Isopropoxy-4,4',6,6'-tetramethyl-[1,1'-biphenyl]-2-yl)oxy](methyl)diphenylsilane [(*S*)-3a'a] and (R)-2'-Isopropoxy-4,4',6,6'-tetramethyl-[1,1'-biphenyl]-2-ol [(*R*)-1a']

Figure S43. *rac*-2'-Isopropoxy-4,4',6,6'-tetramethyl-[1,1'-biphenyl]-2-ol (*rac*-**1a'**).

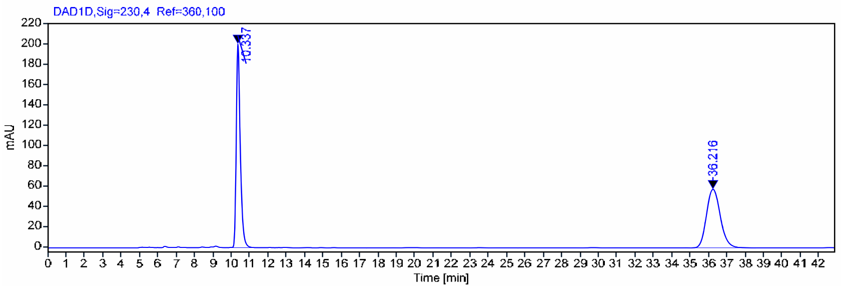

Signal:	DAD1D,Sig=230,4 Ref=360,100			
**RT [min]**	**Width [min]**	**Area**	**Height**	**Area%**
10.337	1.62	3072.33	200.21	50.43
36.216	3.52	3020.13	57.49	49.57
	**Sum**	**6092.46**		

Figure S44. (*S*)-2'-Isopropoxy-4,4',6,6'-tetramethyl-[1,1'-biphenyl]-2-ol [(*S*)-**1a'**, 52% *ee*].

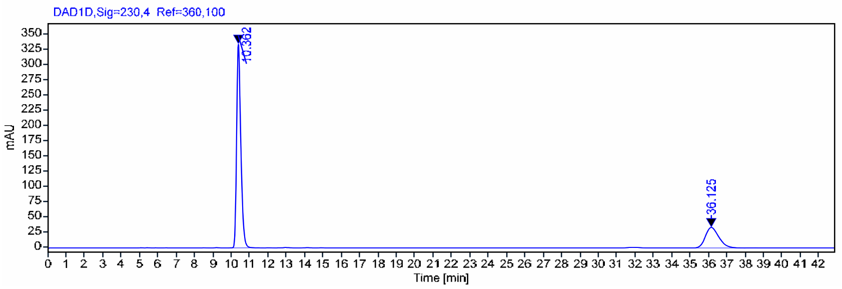

Signal:	DAD1D,Sig=230,4 Ref=360,100			
**RT [min]**	**Width [min]**	**Area**	**Height**	**Area%**
10.362	1.76	5340.27	333.66	75.76
36.125	3.19	1708.36	33.60	24.24
	**Sum**	**7048.63**		

Figure S45. (*R*)-2'-Isopropoxy-4,4',6,6'-tetramethyl-[1,1'-biphenyl]-2-ol [(*R*)-**1a'**, 97% *ee*].

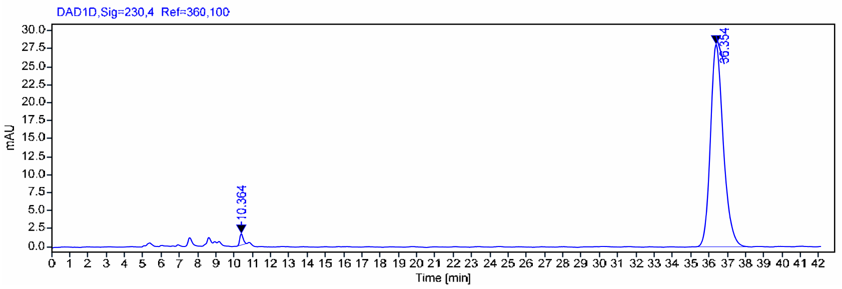

Signal:	DAD1D,Sig=230,4 Ref=360,100			
**RT [min]**	**Width [min]**	**Area**	**Height**	**Area%**
10.364	0.54	20.09	1.55	1.44
36.354	2.82	1371.95	28.10	98.56
	**Sum**	**1392.04**		

(*S*)-[(14-Isopropoxy-5,6,7,8,9,10-hexahydrodibenzo[*a*,*c*][10]annulen-1-yl)oxy](methyl)-diphenylsilane [(*S*)-3b'a] and (*R*)-14-Isopropoxy-5,6,7,8,9,10-hexahydrodibenzo[*a*,*c*]-[10]annulen-1-ol [(*R*)-1b']

Figure S46. *rac*-14-Isopropoxy-5,6,7,8,9,10-hexahydrodibenzo[*a*,*c*][10]annulen-1-ol (*rac*-**1b'**).

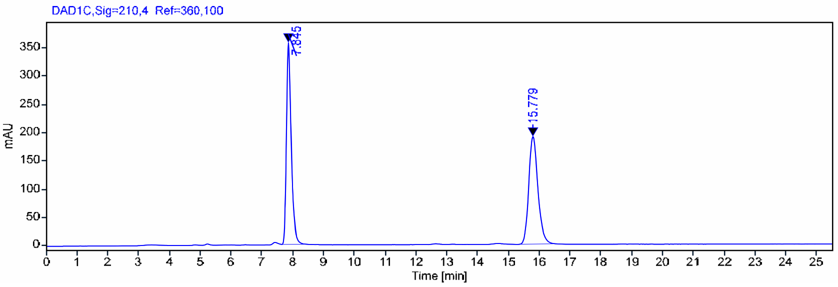

Signal:	DAD1C,Sig=210,4 Ref=360,100			
**RT [min]**	**Width [min]**	**Area**	**Height**	**Area%**
7.845	0.81	3900.85	356.28	49.90
15.779	1.18	3916.33	189.79	50.10
	**Sum**	**7817.18**		

Figure S47. (*S*)-14-Isopropoxy-5,6,7,8,9,10-hexahydrodibenzo[*a*,*c*][10]annulen-1-ol [(*S*)-**1b'**, 59% *ee*].

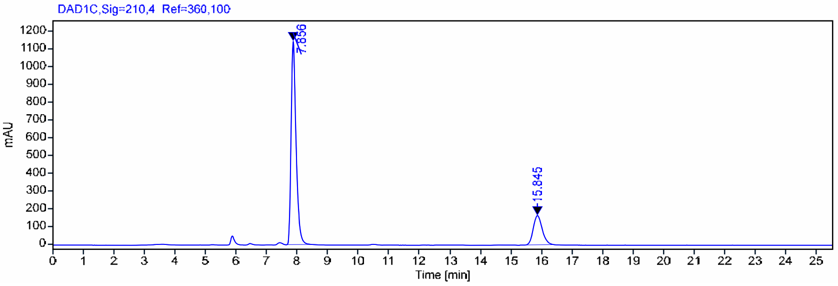

Signal:	DAD1C,Sig=210,4 Ref=360,100			
**RT [min]**	**Width [min]**	**Area**	**Height**	**Area%**
7.856	0.86	12899.28	1144.65	79.43
15.845	0.91	3340.10	164.59	20.57
	**Sum**	**16239.38**		

Figure S48. (*R*)-14-Isopropoxy-5,6,7,8,9,10-hexahydrodibenzo[*a*,*c*][10]annulen-1-ol [(*R*)-**1b'**, 86% *ee*].

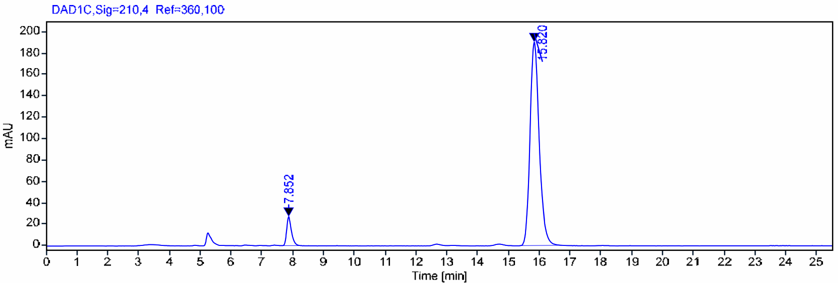

Signal:	DAD1C,Sig=210,4 Ref=360,100			
**RT [min]**	**Width [min]**	**Area**	**Height**	**Area%**
7.852	0.59	291.65	27.05	6.89
15.820	1.37	3943.19	190.10	93.11
	**Sum**	**4234.84**		

(*S*)-[(2'-Isopropoxy-[1,1'-binaphthalen]-2-yl)oxy](methyl)diphenylsilane [(*S*)-3ca] and (*R*)-2'-Isopropoxy-[1,1'-binaphthalen]-2-ol [(*R*)-1c] from the Scale-Up Reaction

Figure S49. *rac*-2'-Isopropoxy-[1,1'-binaphthalen]-2-ol (*rac*-**1c**).

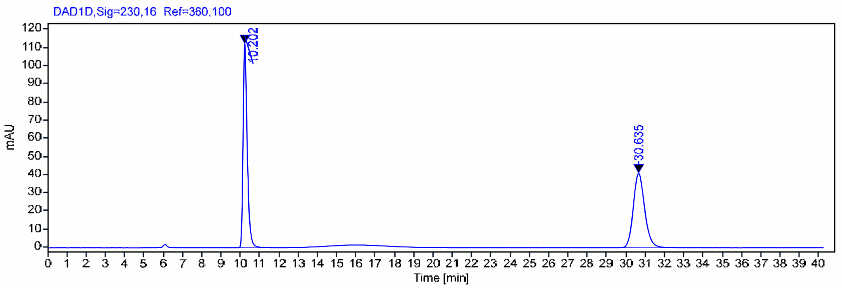

Signal:	DAD1D,Sig=230,16 Ref=360,100			
**RT [min]**	**Width [min]**	**Area**	**Height**	**Area%**
10.202	1.36	1667.88	112.18	50.01
30.635	2.76	1667.03	40.85	49.99
	**Sum**	**3334.91**		

Figure S50. (*S*)-2'-Isopropoxy-[1,1'-binaphthalen]-2-ol [(*S*)-**1c**, 77% *ee*].

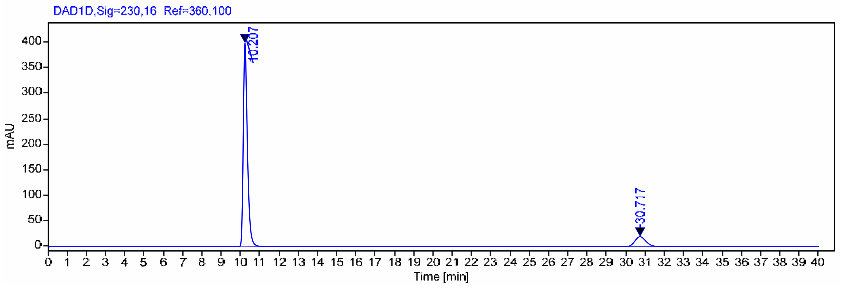

Signal:	DAD1D,Sig=230,16 Ref=360,100			
**RT [min]**	**Width [min]**	**Area**	**Height**	**Area%**
10.207	1.38	5932.99	399.16	88.40
30.717	1.79	778.59	19.55	11.60
	**Sum**	**6711.58**		

Figure S51. (*R*)-2'-Isopropoxy-[1,1'-binaphthalen]-2-ol [(*R*)-**1c**, 61% *ee*].

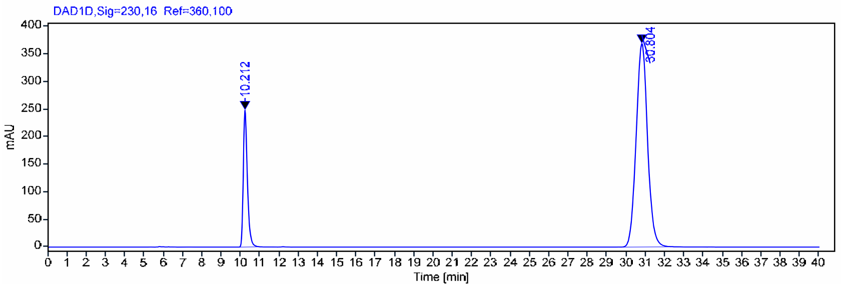

Signal:	DAD1D,Sig=230,16 Ref=360,100			
**RT [min]**	**Width [min]**	**Area**	**Height**	**Area%**
10.212	1.20	3680.98	248.74	19.49
30.804	3.04	15201.53	369.63	80.51
	**Sum**	**18882.51**		

(*R*)-[1,1'-Binaphthalene]-2,2'-diol [(*R*)-1a] from the Reaction of (*R*)-2'-Isopropoxy-[1,1'-binaphthalen]-2-ol [(*R*)-1c] with Boron Trichloride

Figure S52. *rac*-[1,1'-Binaphthalene]-2,2'-diol (*rac*-**1a**).

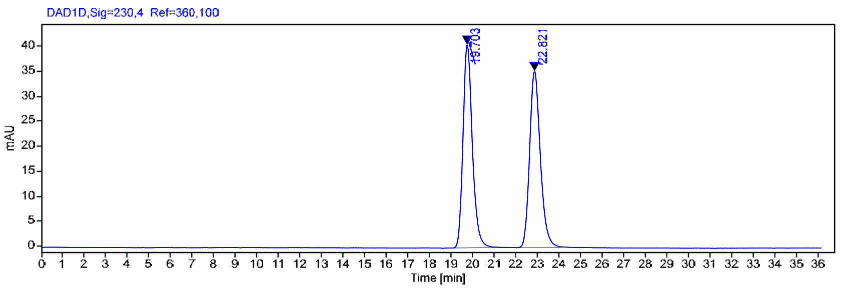

Signal:	DAD1D,Sig=230,4 Ref=360,100			
RT [min]	Width [min]	Area	Height	Area%
19.703	2.50	1223.45	40.57	50.18
22.821	2.28	1214.91	35.21	49.82
	**Sum**	**2438.35**		

Figure S53. (*R*)-[1,1'-Binaphthalene]-2,2'-diol [(*R*)-**1a**, 64% *ee*].

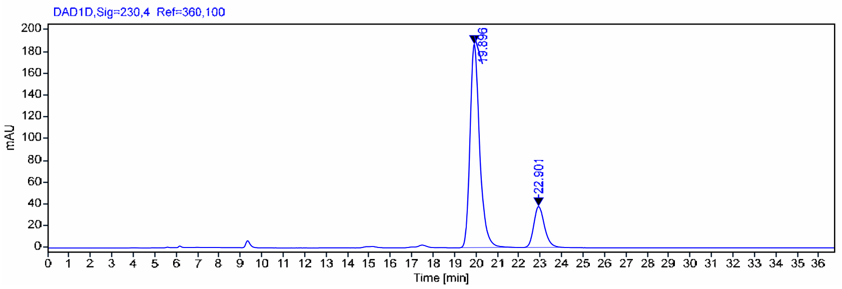

Signal:	DAD1D,Sig=230,4 Ref=360,100			
RT [min]	Width [min]	Area	Height	Area%
19.896	2.55	5845.48	186.71	81.80
22.901	1.84	1300.39	37.54	18.20
	**Sum**	**7145.87**		

9 NMR Spectra

Figure S54. ^{1}H NMR (500 MHz, $CDCl_3$) of *rac*-4,4'-Dibromo-2'-isopropoxy-[1,1'-binaphthalen]-2-ol (*rac*-**1n**).

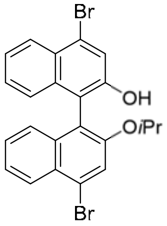

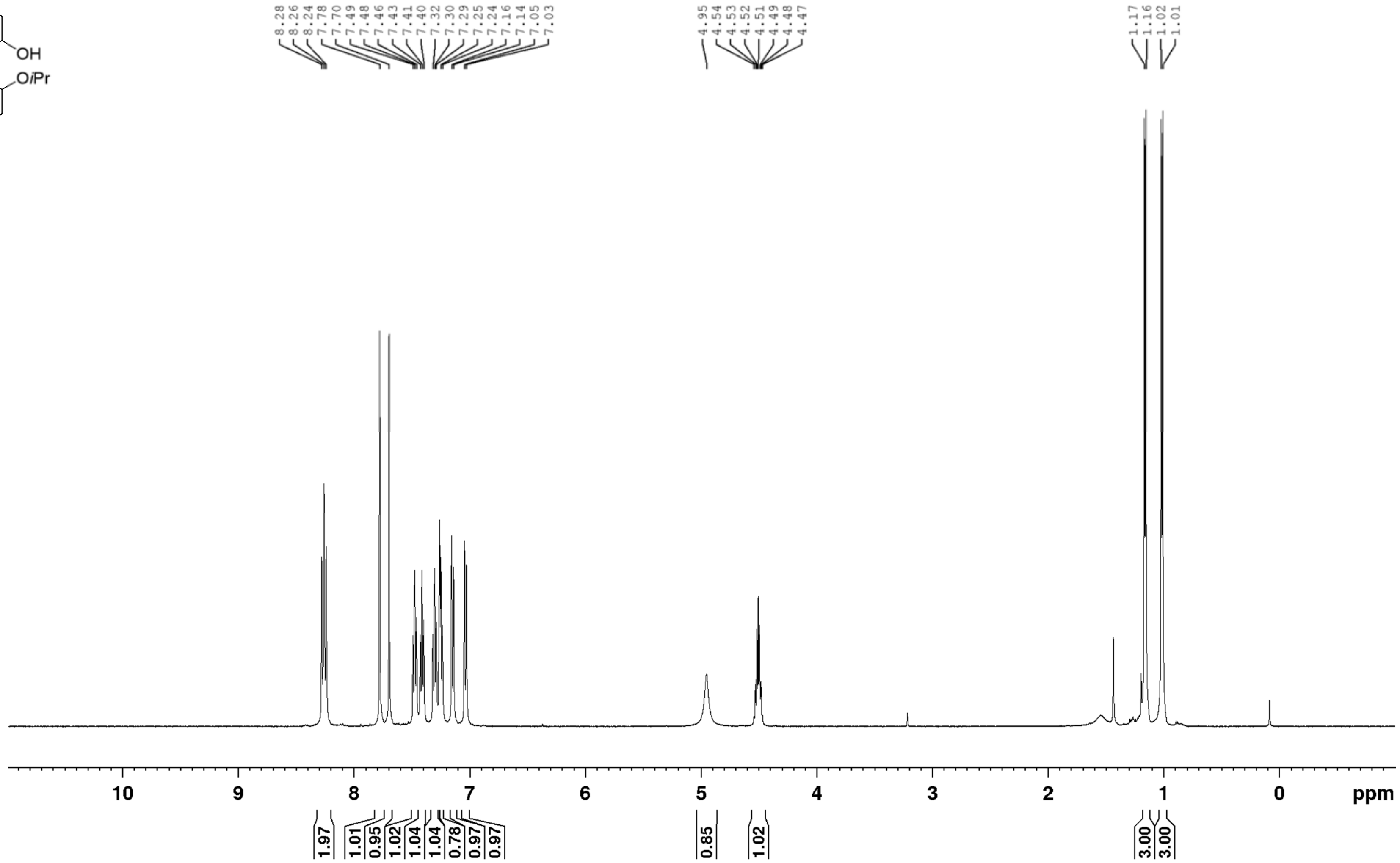

Figure S55. $^{13}C\{^{1}H\}$ NMR (126 MHz, $CDCl_3$) of *rac*-4,4'-Dibromo-2'-isopropoxy-[1,1'-binaphthalen]-2-ol (*rac*-**1n**).

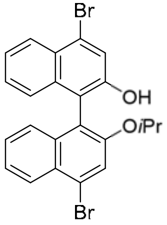

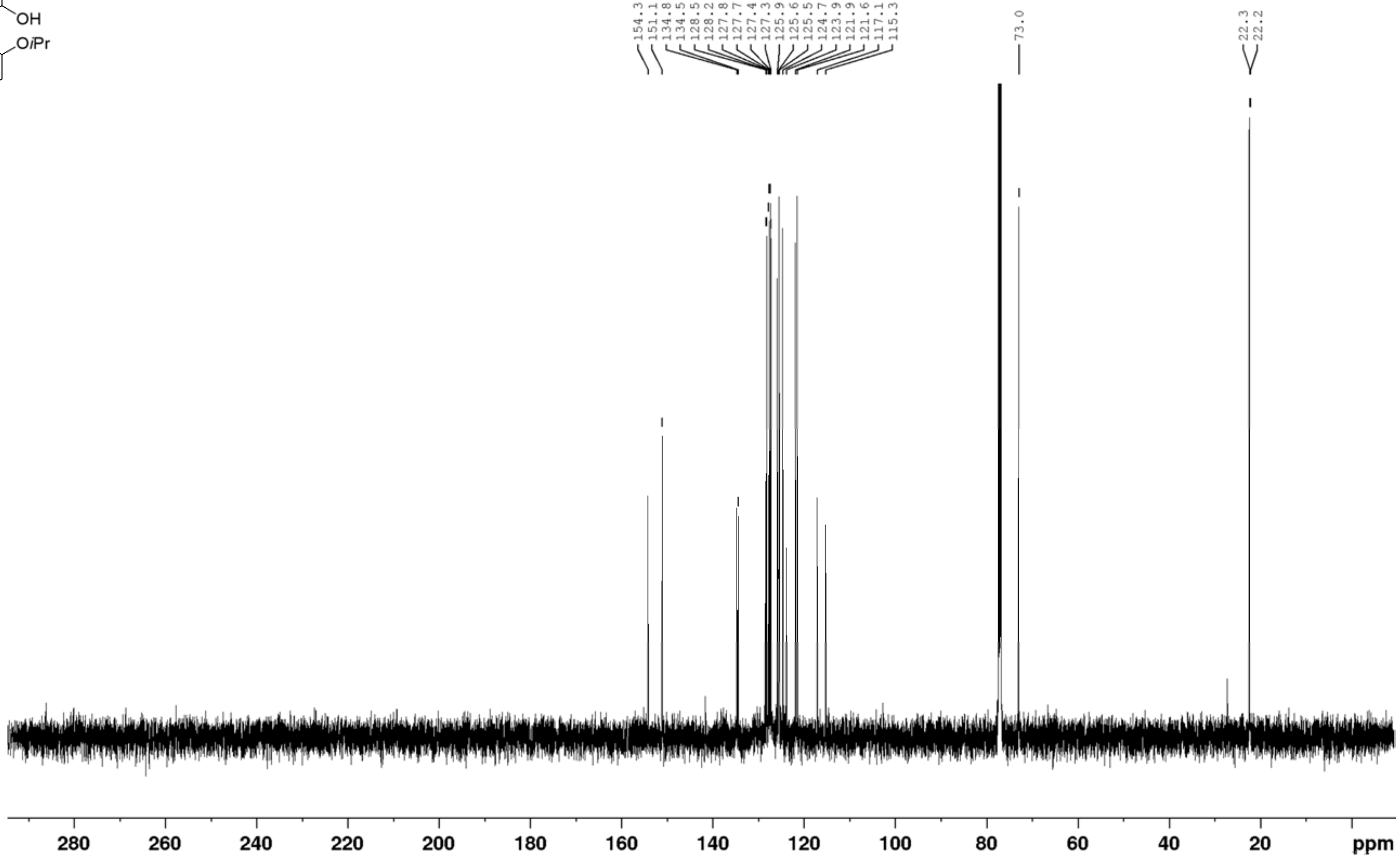

Figure S56. ^{1}H NMR (500 MHz, $CDCl_3$) of *rac*-2'-Isopropoxy-4,4'-diphenyl-[1,1'-binaphthalen]-2-ol (*rac*-**1m**).

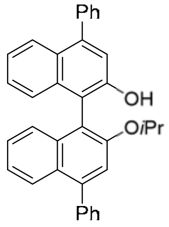

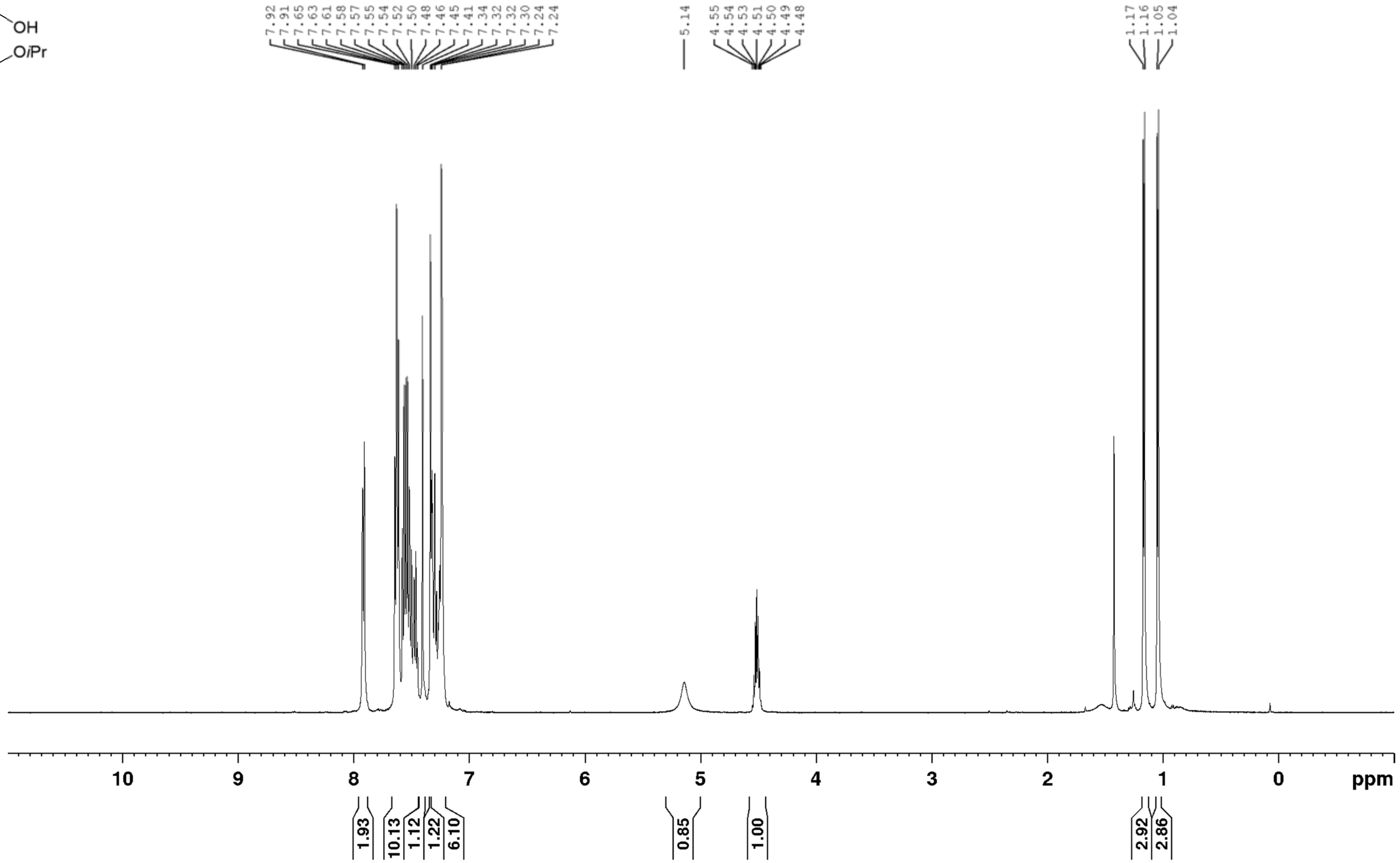

Figure S57. $^{13}C\{^{1}H\}$ NMR (126 MHz, $CDCl_3$) of *rac*-2'-Isopropoxy-4,4'-diphenyl-[1,1'-binaphthalen]-2-ol (*rac*-**1m**).

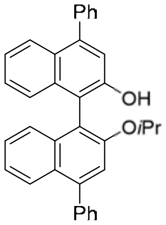

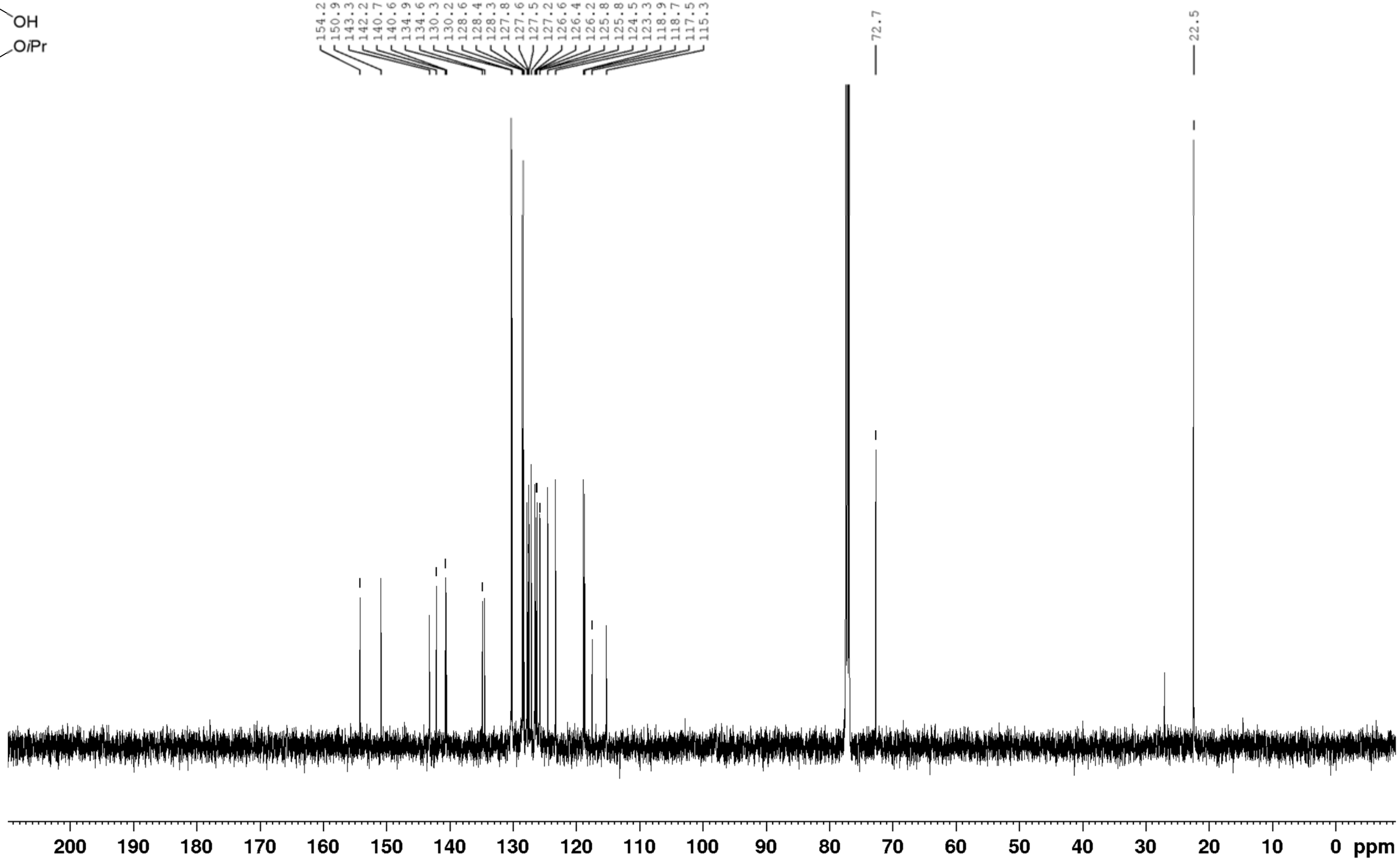

Figure S58. ^{1}H NMR (500 MHz, $CDCl_3$) of *rac*-7,7'-Dibromo-2'-isopropoxy-[1,1'-binaphthalen]-2-ol (*rac*-**1p**).

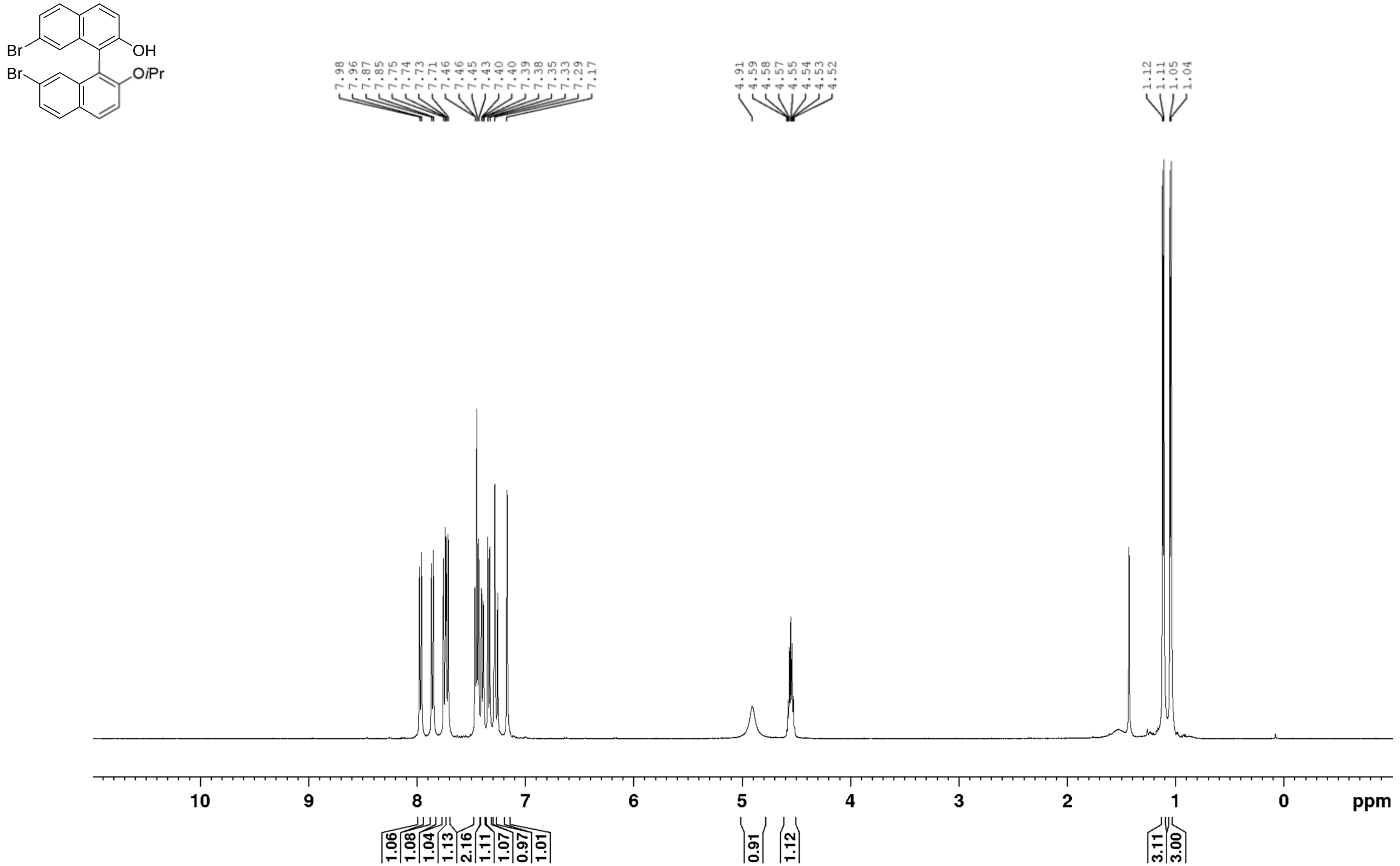

Figure S59. $^{13}C\{^{1}H\}$ NMR (126 MHz, $CDCl_3$) of *rac*-7,7'-Dibromo-2'-isopropoxy-[1,1'-binaphthalen]-2-ol (*rac*-**1p**).

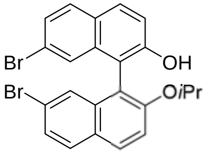

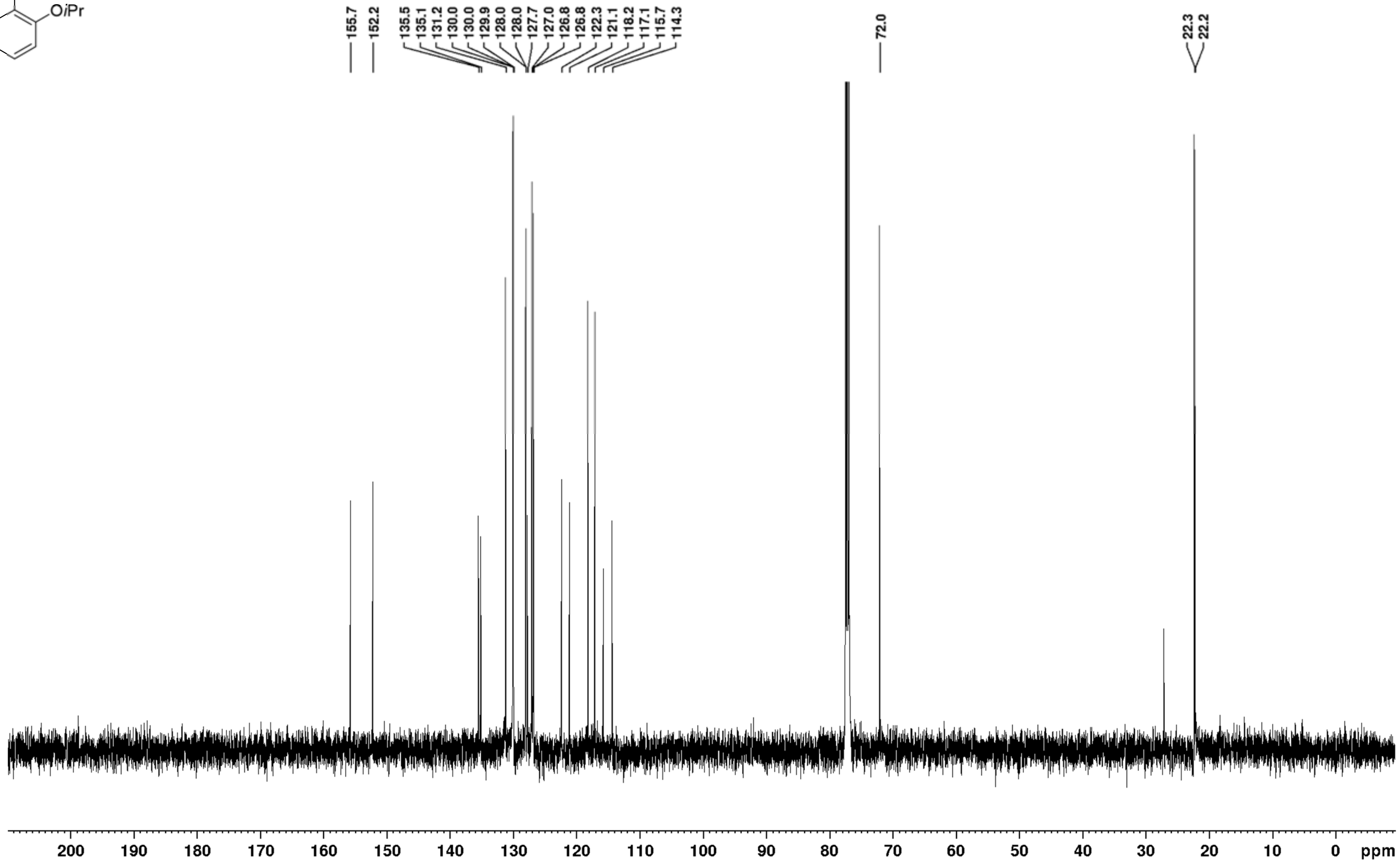

Figure S60. ^{1}H NMR (500 MHz, $CDCl_3$) of *rac*-6,6'-Dibromo-2'-isopropoxy-[1,1'-binaphthalen]-2-ol (*rac*-**S1**).

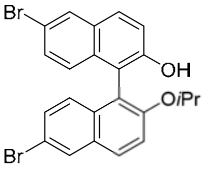

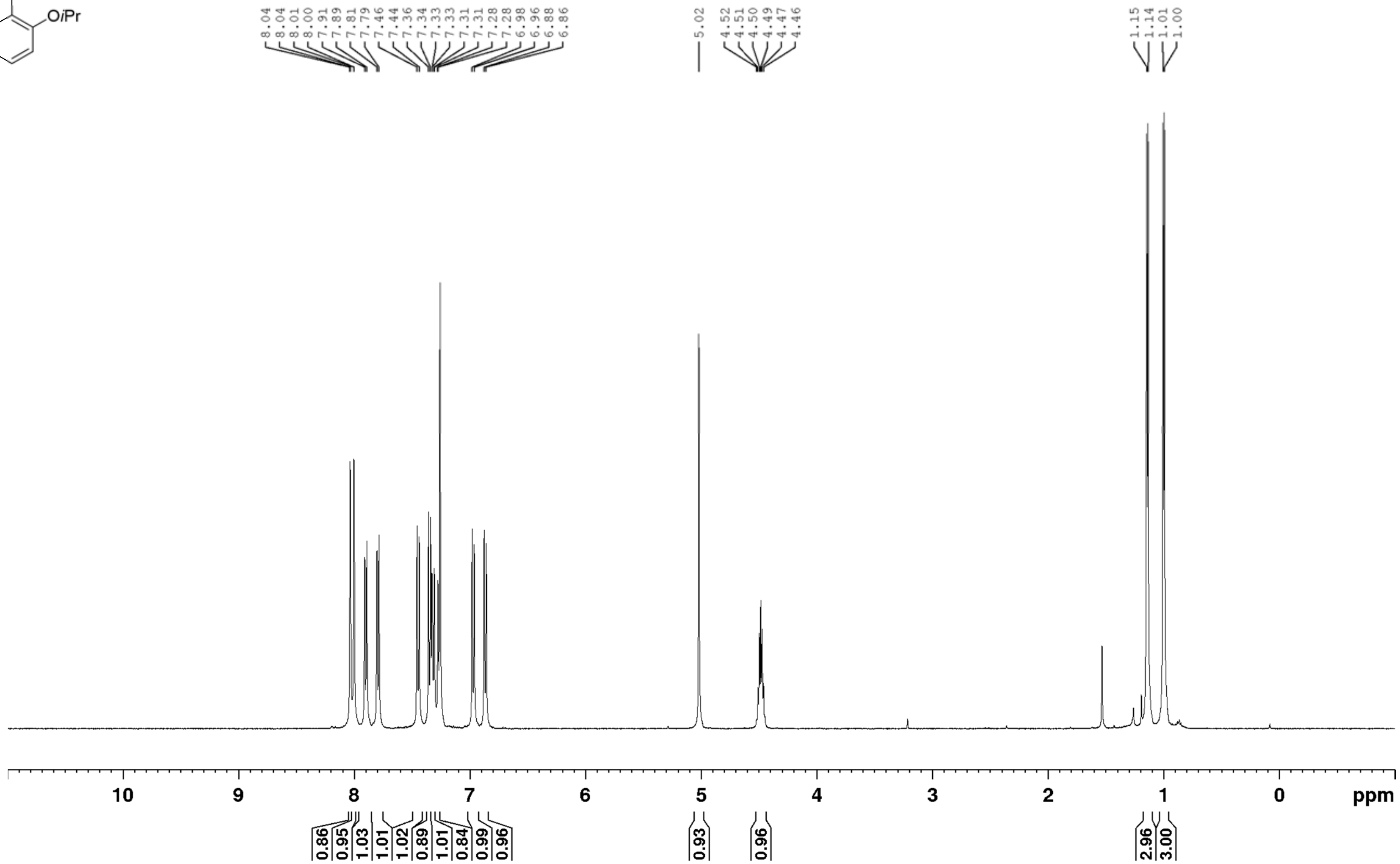

Figure S61. $^{13}C\{^{1}H\}$ NMR (126 MHz, $CDCl_3$) of *rac*-6,6'-Dibromo-2'-isopropoxy-[1,1'-binaphthalen]-2-ol (*rac*-**S1**).

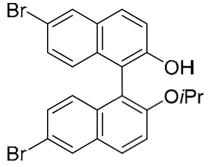

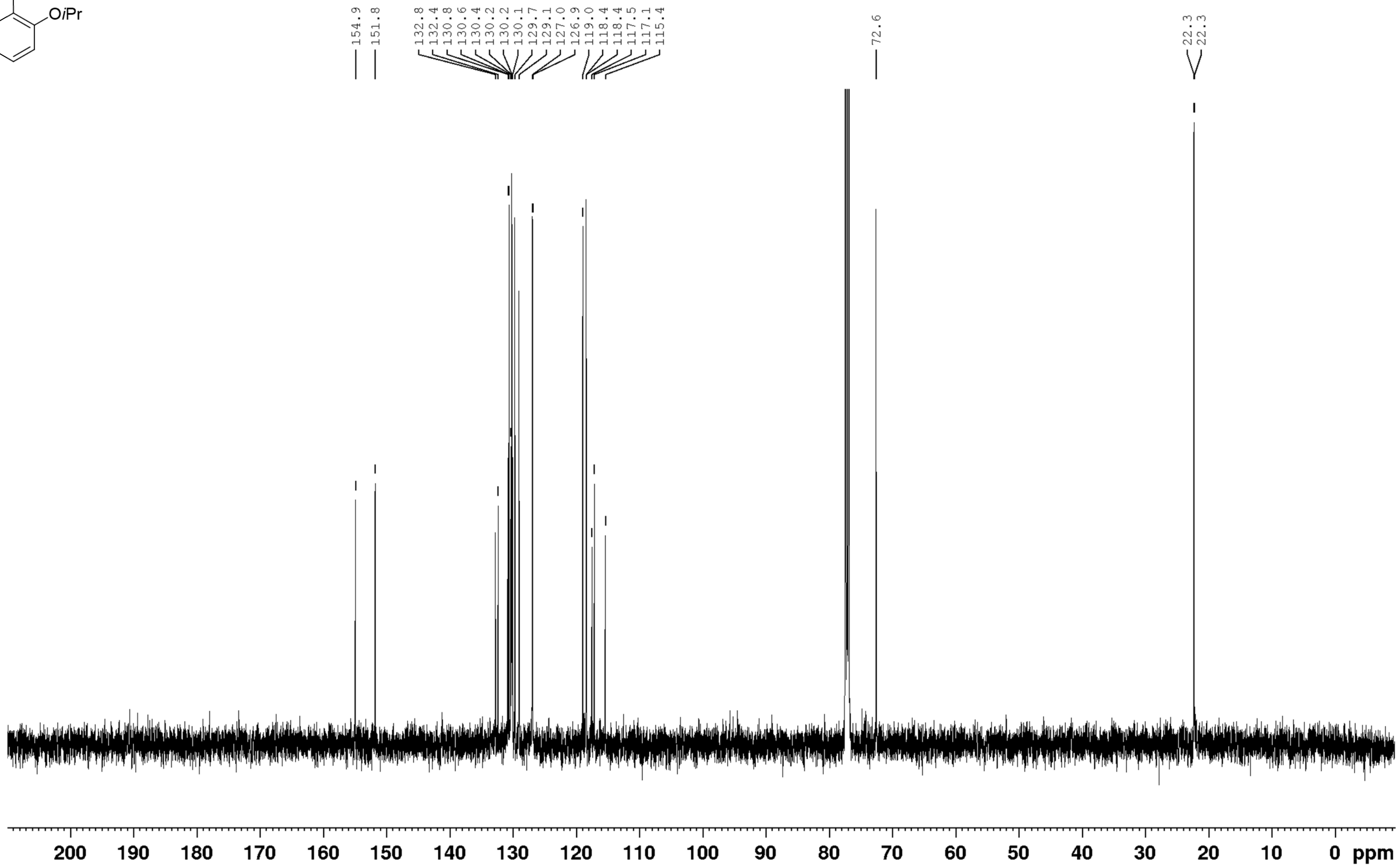

Figure S62. ^1H NMR (500 MHz, $CDCl_3$) of *rac*-5,5'-Bis(methoxymethoxy)-4,4'-bibenzo[*d*][1,3]dioxole (*rac*-**S3**).

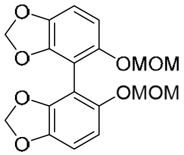

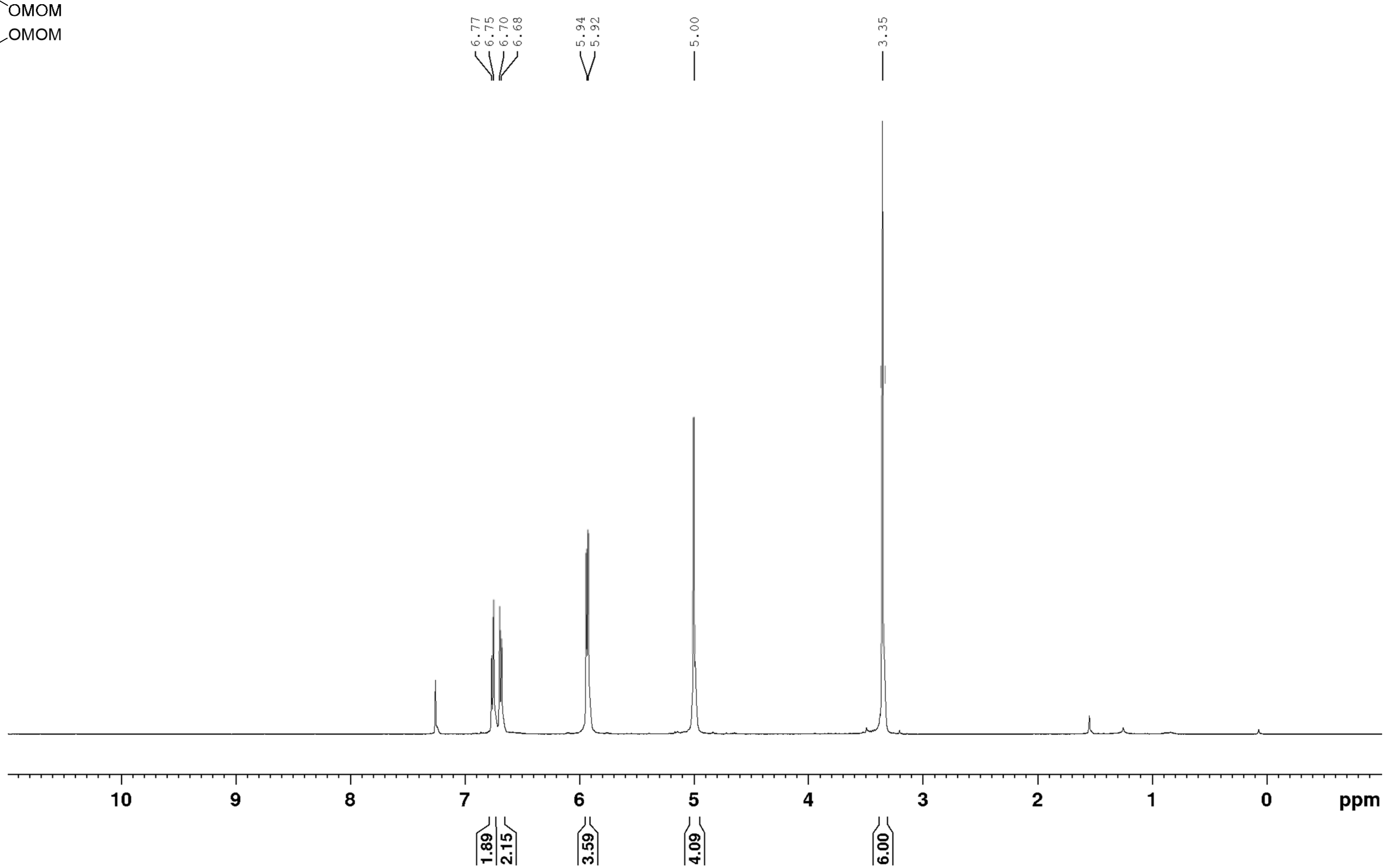

Figure S63. $^{13}C\{^{1}H\}$ NMR (126 MHz, $CDCl_3$) of *rac*-5,5'-Bis(methoxymethoxy)-4,4'-bibenzo[*d*][1,3]dioxole (*rac*-**S3**).

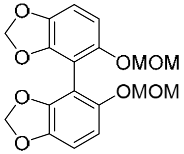

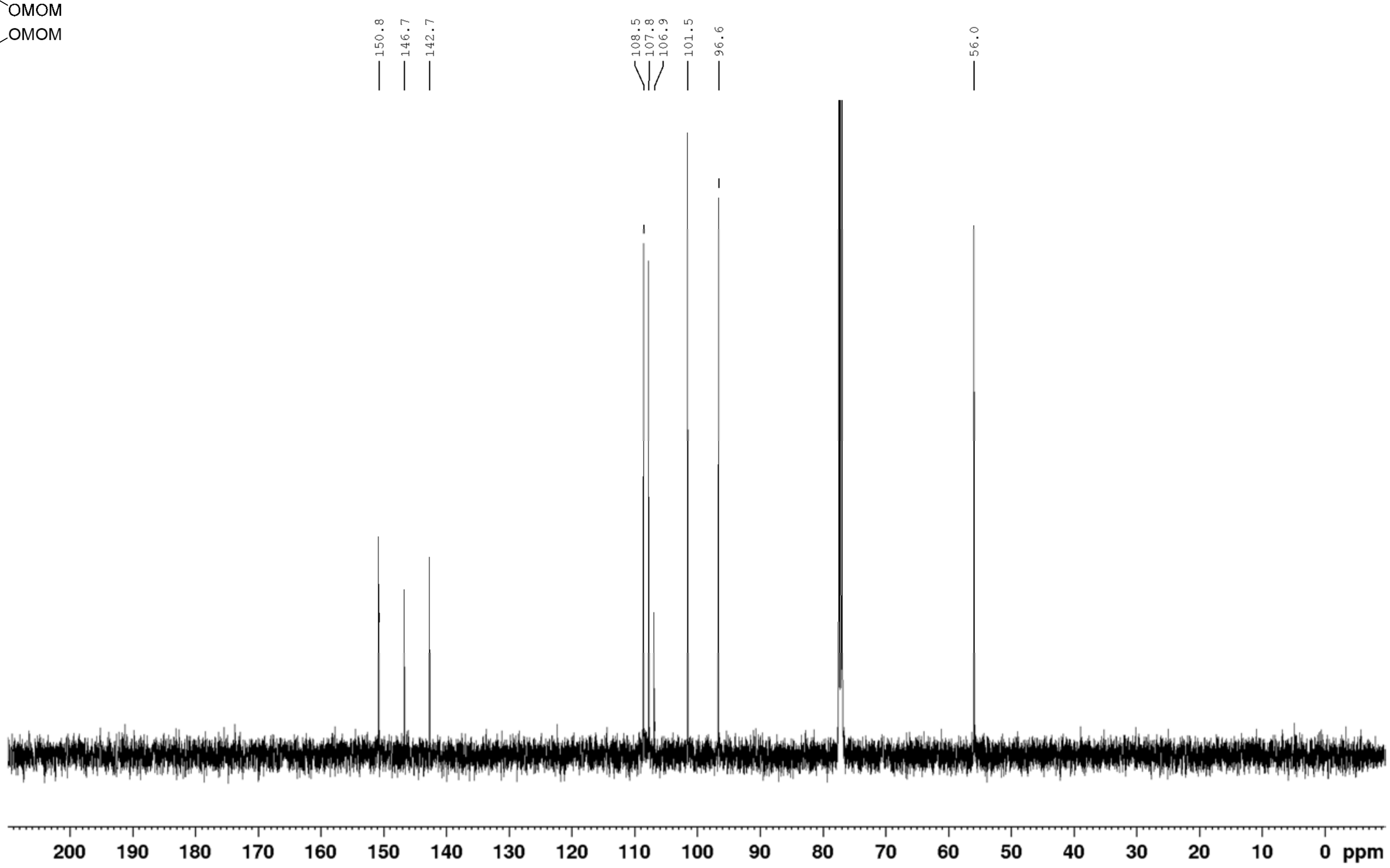

Figure S64. ^{1}H NMR (400 MHz, C_6D_6) of *rac*-5'-Isopropoxy-[4,4'-bibenzo[*d*][1,3]dioxol]-5-ol (*rac*-**1t**).

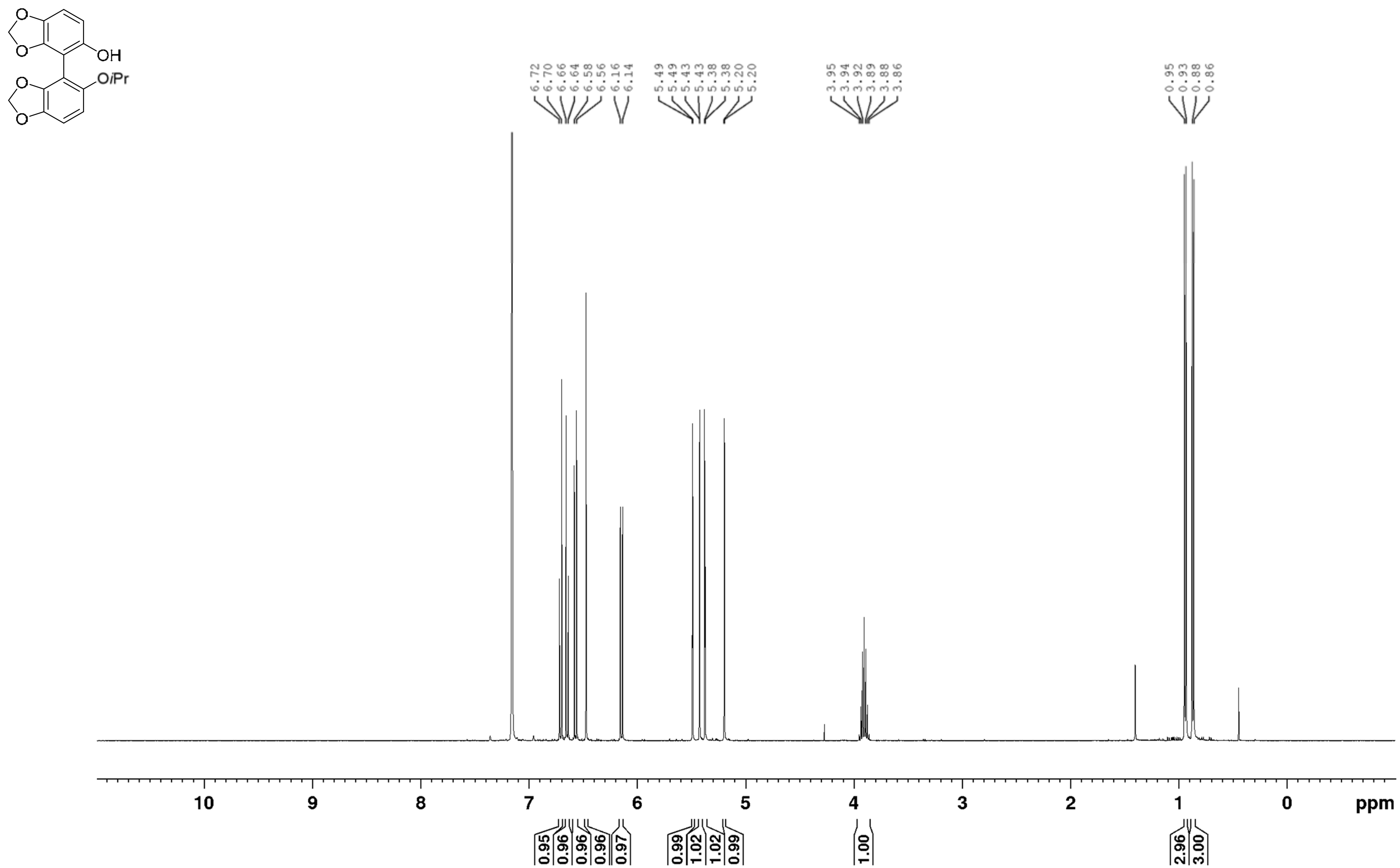

Figure S65. $^{13}C\{^{1}H\}$ NMR (101 MHz, C_6D_6) of *rac*-5'-Isopropoxy-[4,4'-bibenzo[*d*][1,3]dioxol]-5-ol (*rac*-**1t**).

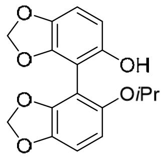

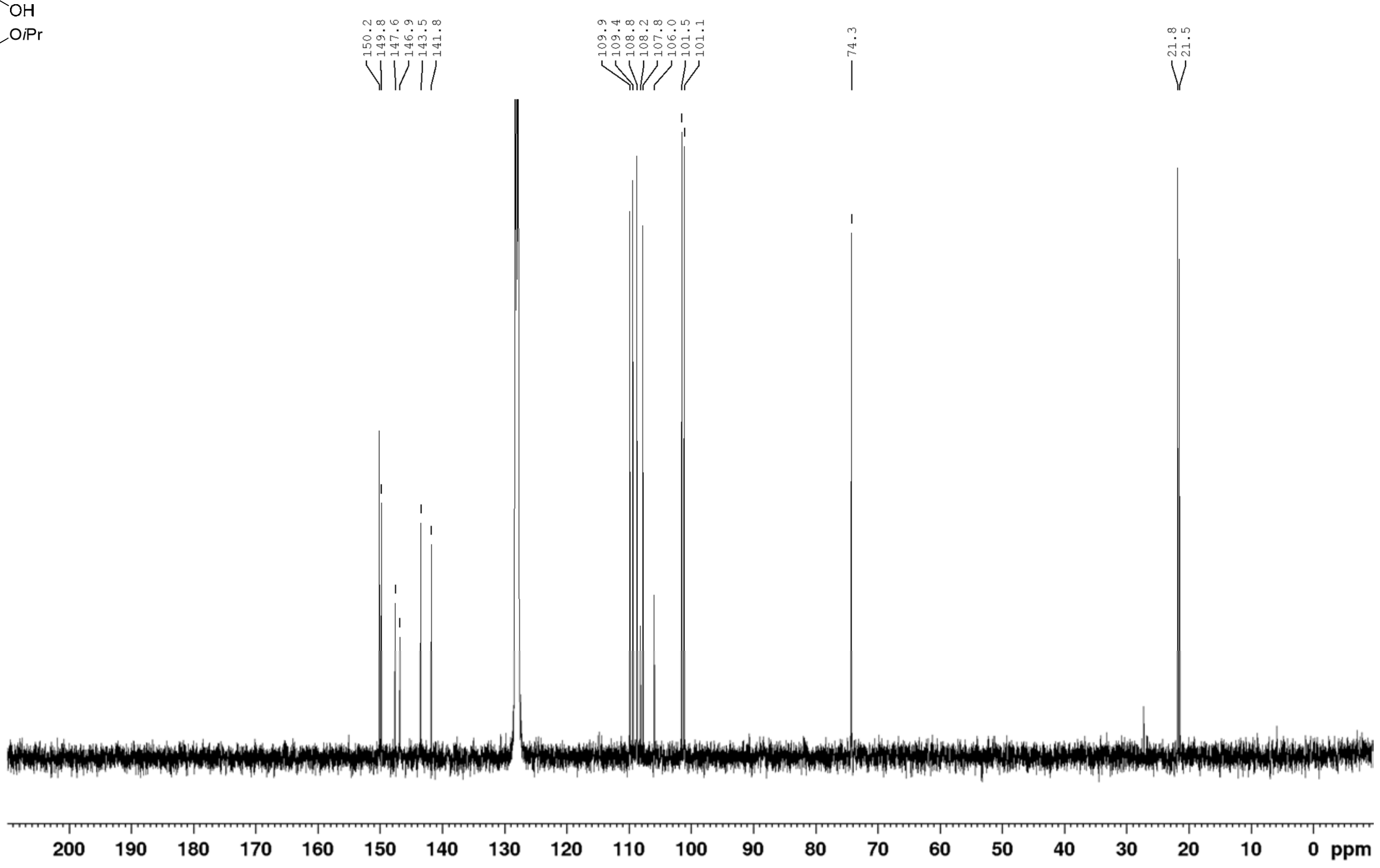

Figure S66. ^{1}H NMR (500 MHz, $CDCl_3$) of *rac*-2'-Isopropoxy-6,6'-dimethoxy-[1,1'-biphenyl]-2-ol (*rac*-**1u**).

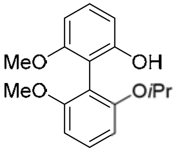

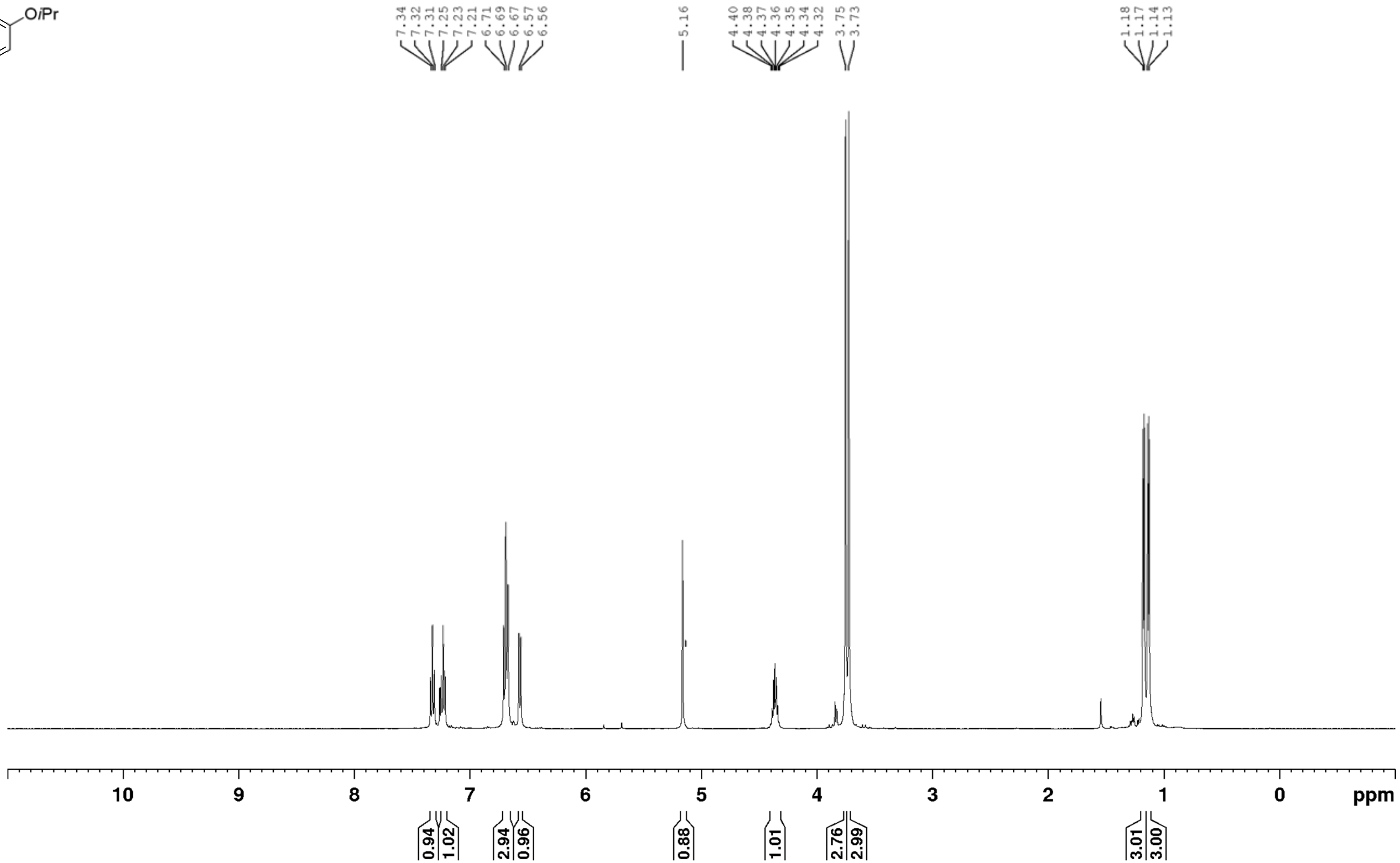

Figure S67. $^{13}C\{^1H\}$ NMR (126 MHz, $CDCl_3$) of *rac*-2'-Isopropoxy-6,6'-dimethoxy-[1,1'-biphenyl]-2-ol (*rac*-**1u**).

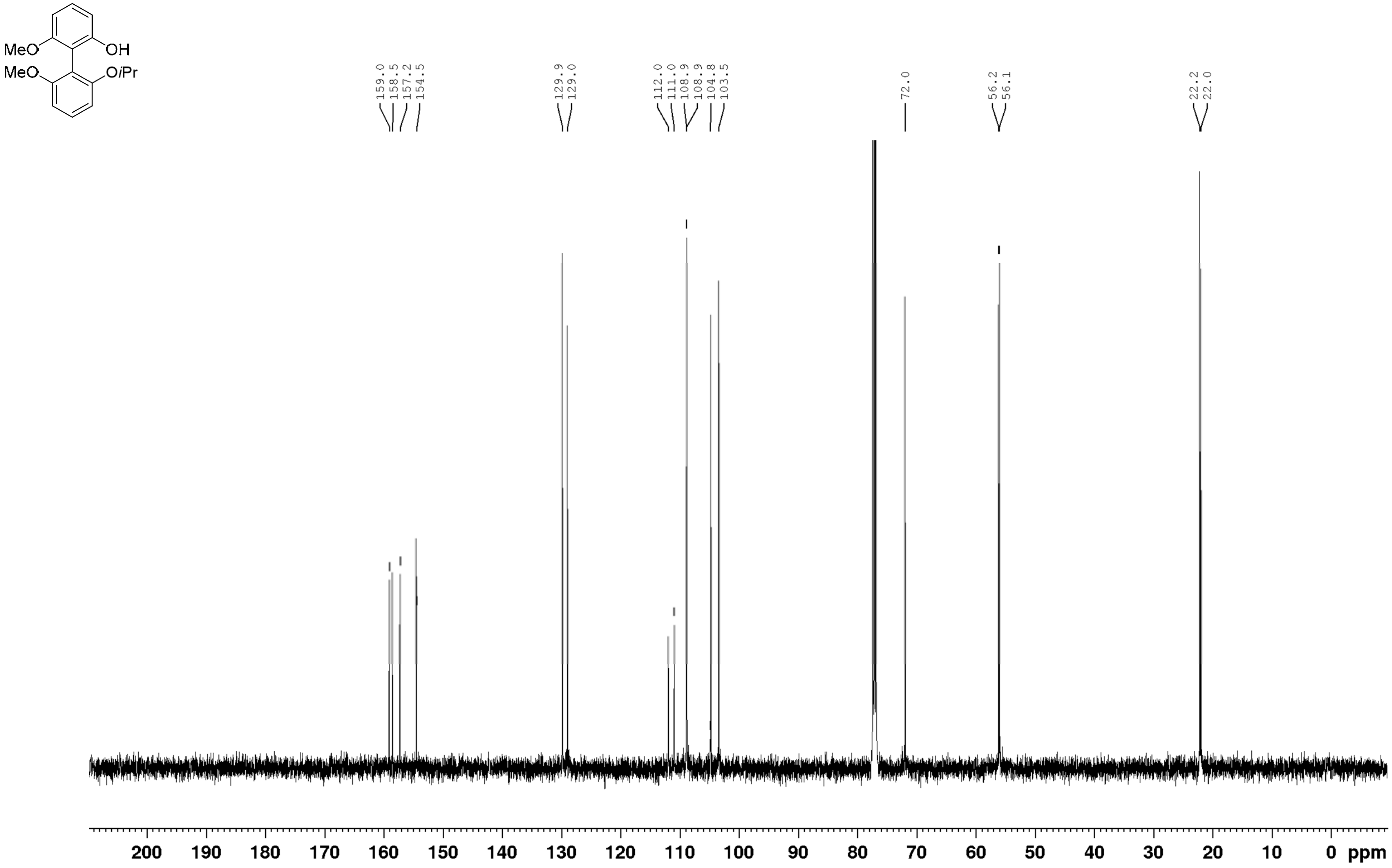

Figure S68. ^{1}H NMR (500 MHz, $CDCl_3$) of 2-Bromo-1-methoxy-3,5-dimethylbenzene (**S6**).

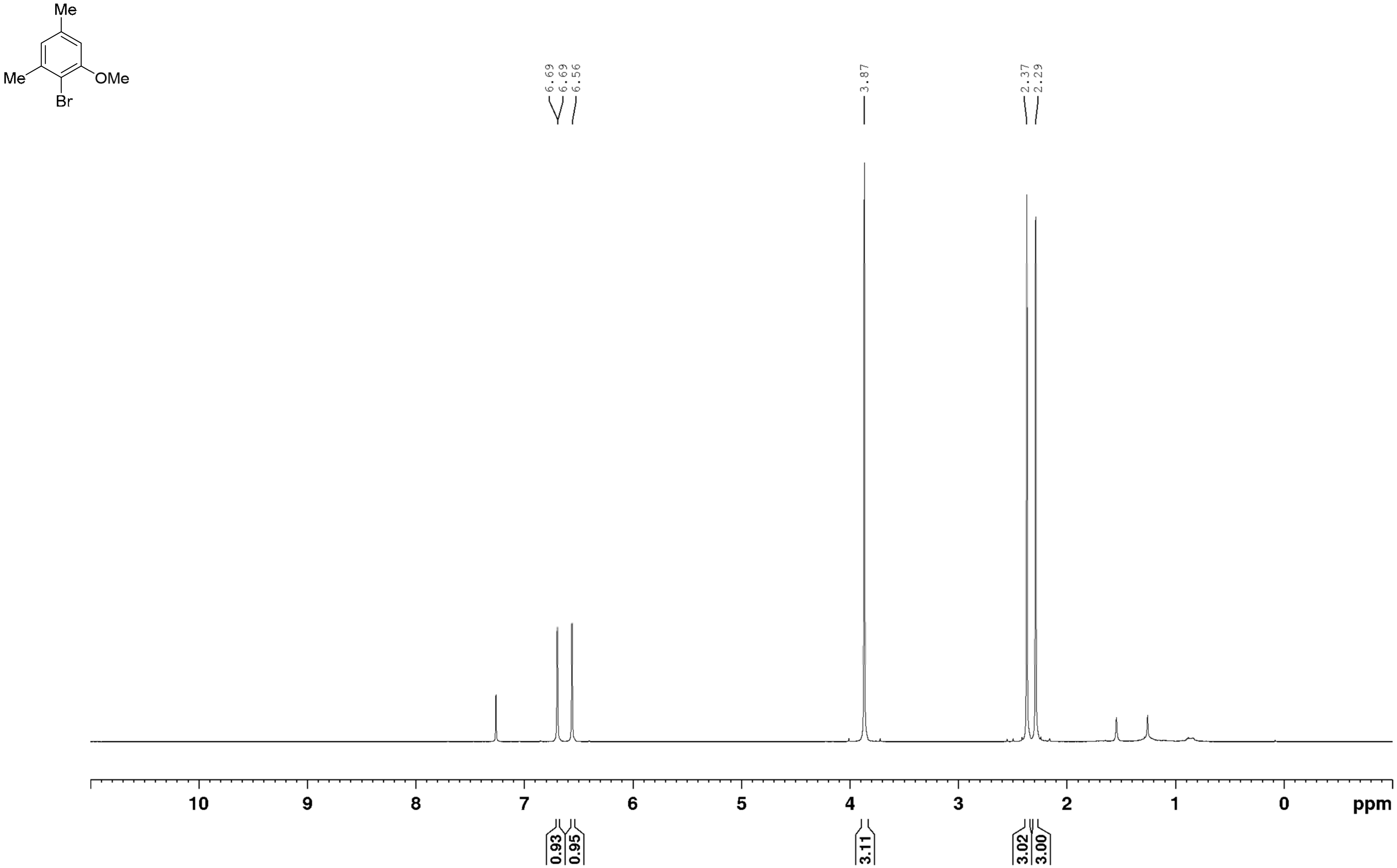

Figure S69. $^{13}C\{^1H\}$ NMR (126 MHz, $CDCl_3$) of 2-Bromo-1-methoxy-3,5-dimethylbenzene (**S6**).

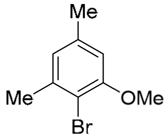

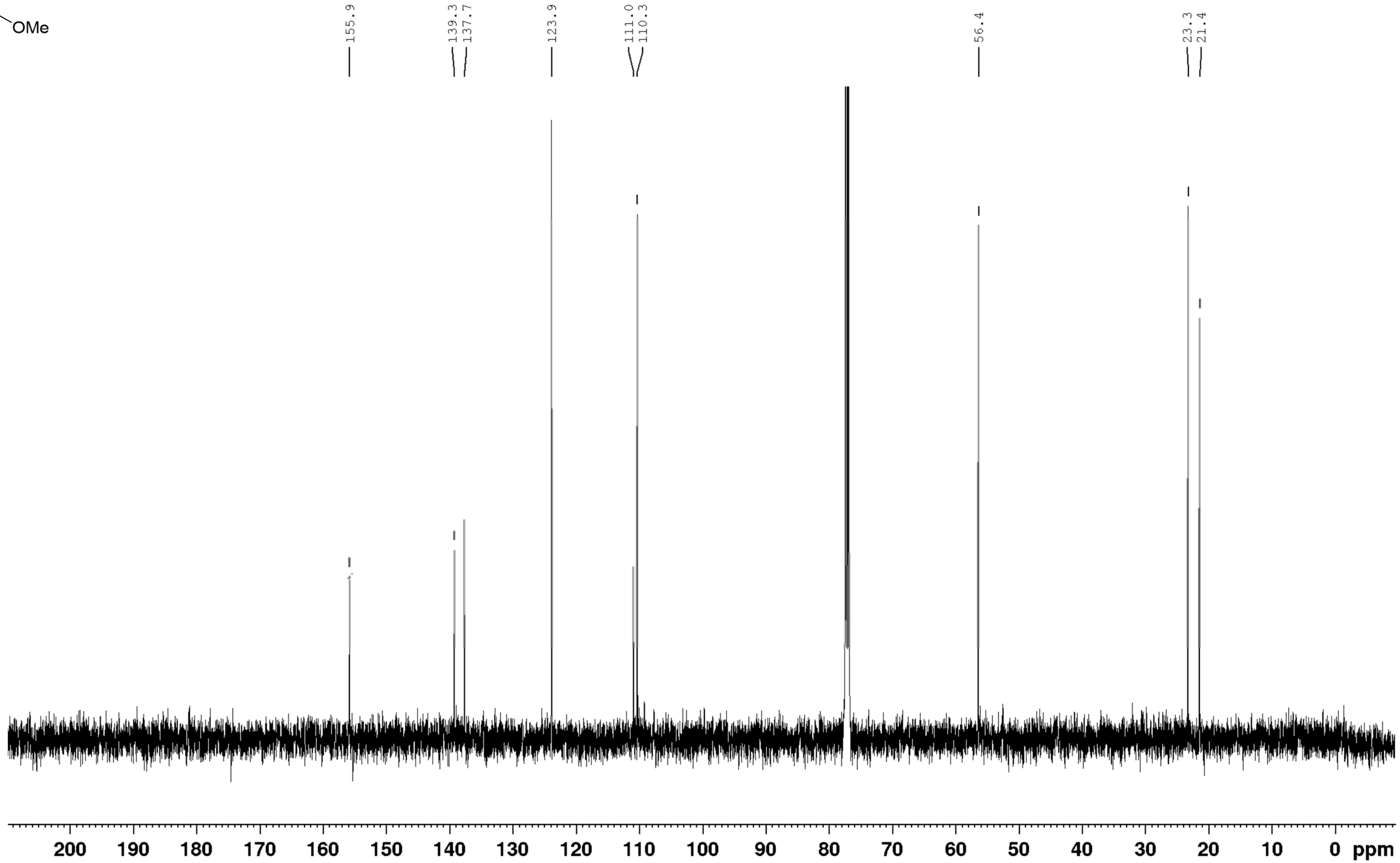

Figure S70. ^{1}H NMR (500 MHz, $CDCl_3$) of *rac*-2,2'-Dimethoxy-4,4',6,6'-tetramethyl-1,1'-biphenyl (*rac*-**S8**).

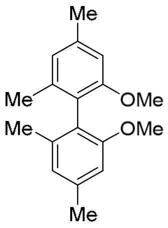

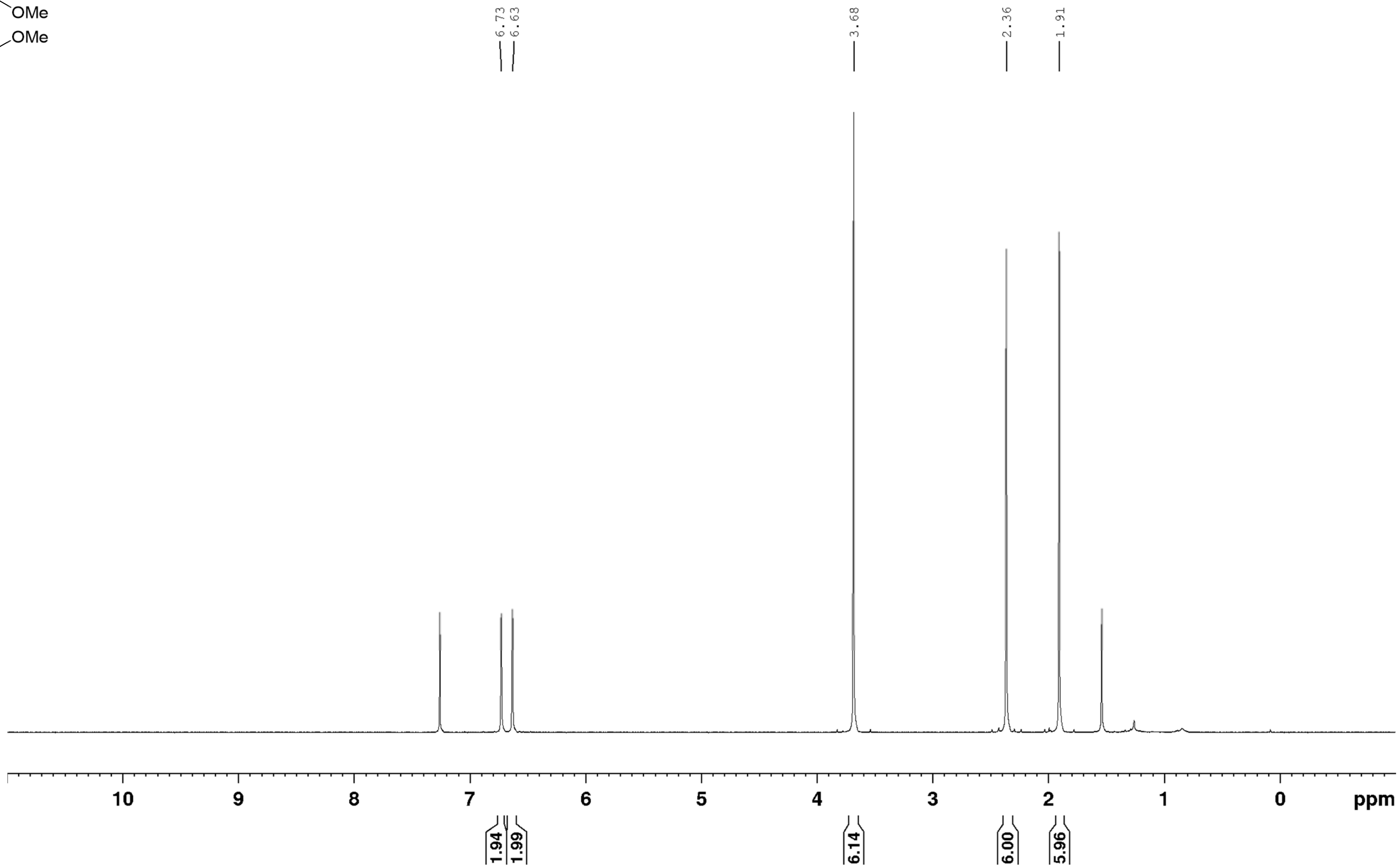

Figure S71. $^{13}C\{^{1}H\}$ NMR (126 MHz, $CDCl_3$) of *rac*-2,2'-Dimethoxy-4,4',6,6'-tetramethyl-1,1'-biphenyl (*rac*-**S8**).

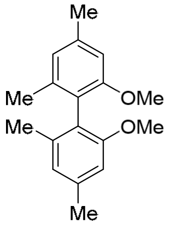

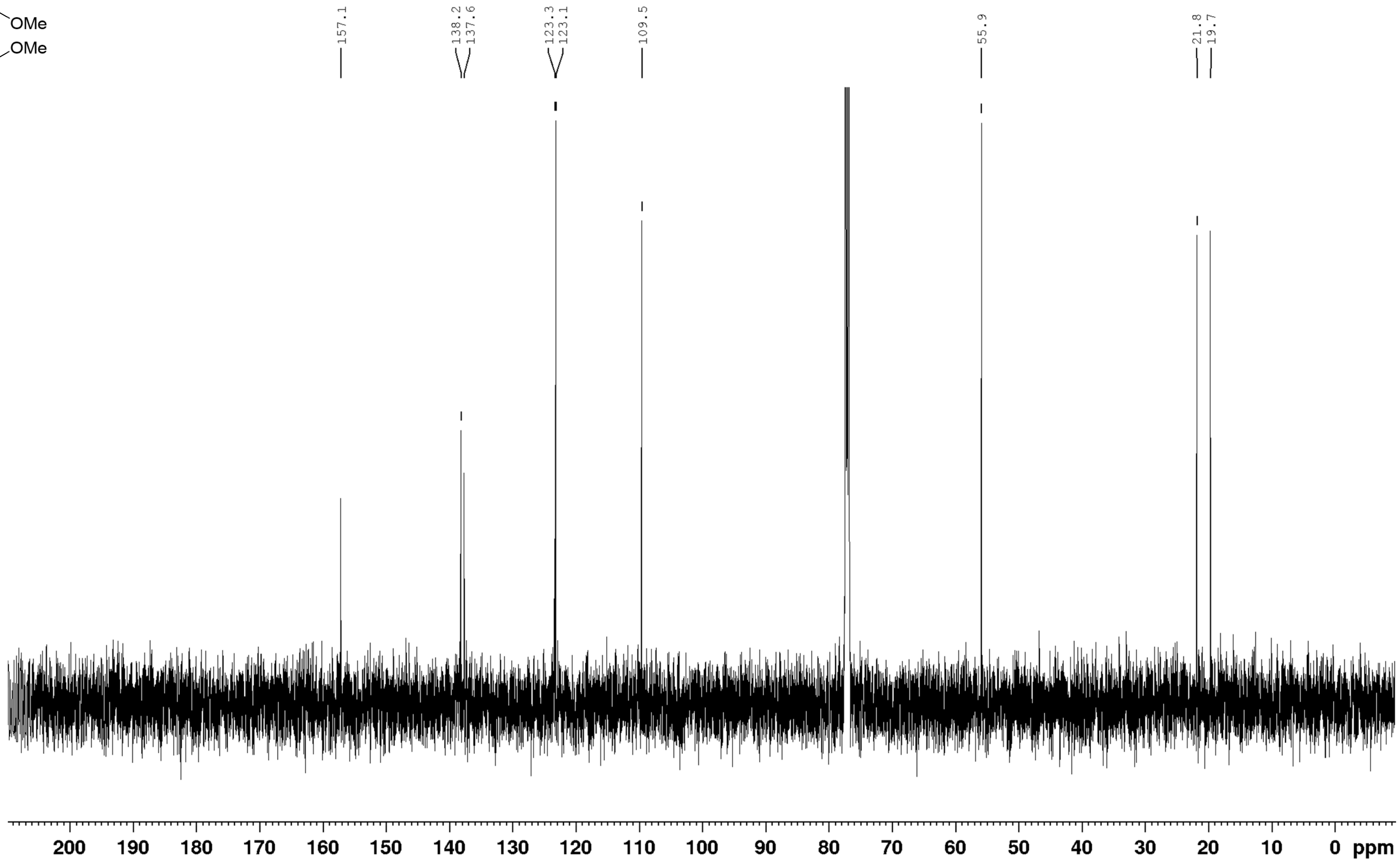

Figure S72. ^{1}H NMR (500 MHz, $CDCl_3$) of *rac*-2,2'-Dimethoxy-6,6'-divinyl-1,1'-biphenyl (*rac*-**S12**).

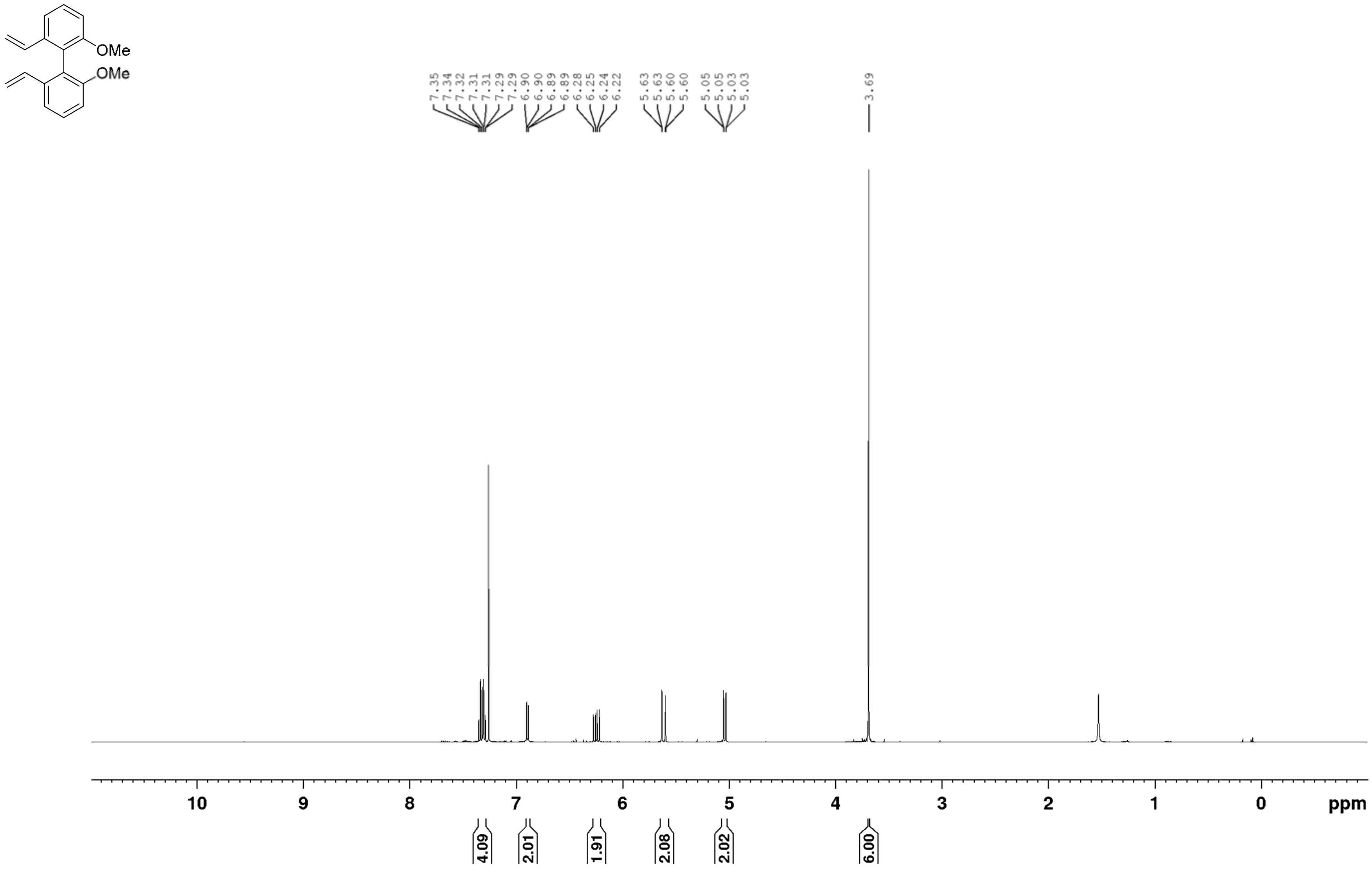

Figure S73. $^{13}C\{^{1}H\}$ NMR (126 MHz, $CDCl_3$) of *rac*-2,2'-Dimethoxy-6,6'-divinyl-1,1'-biphenyl (*rac*-**S12**).

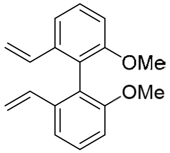

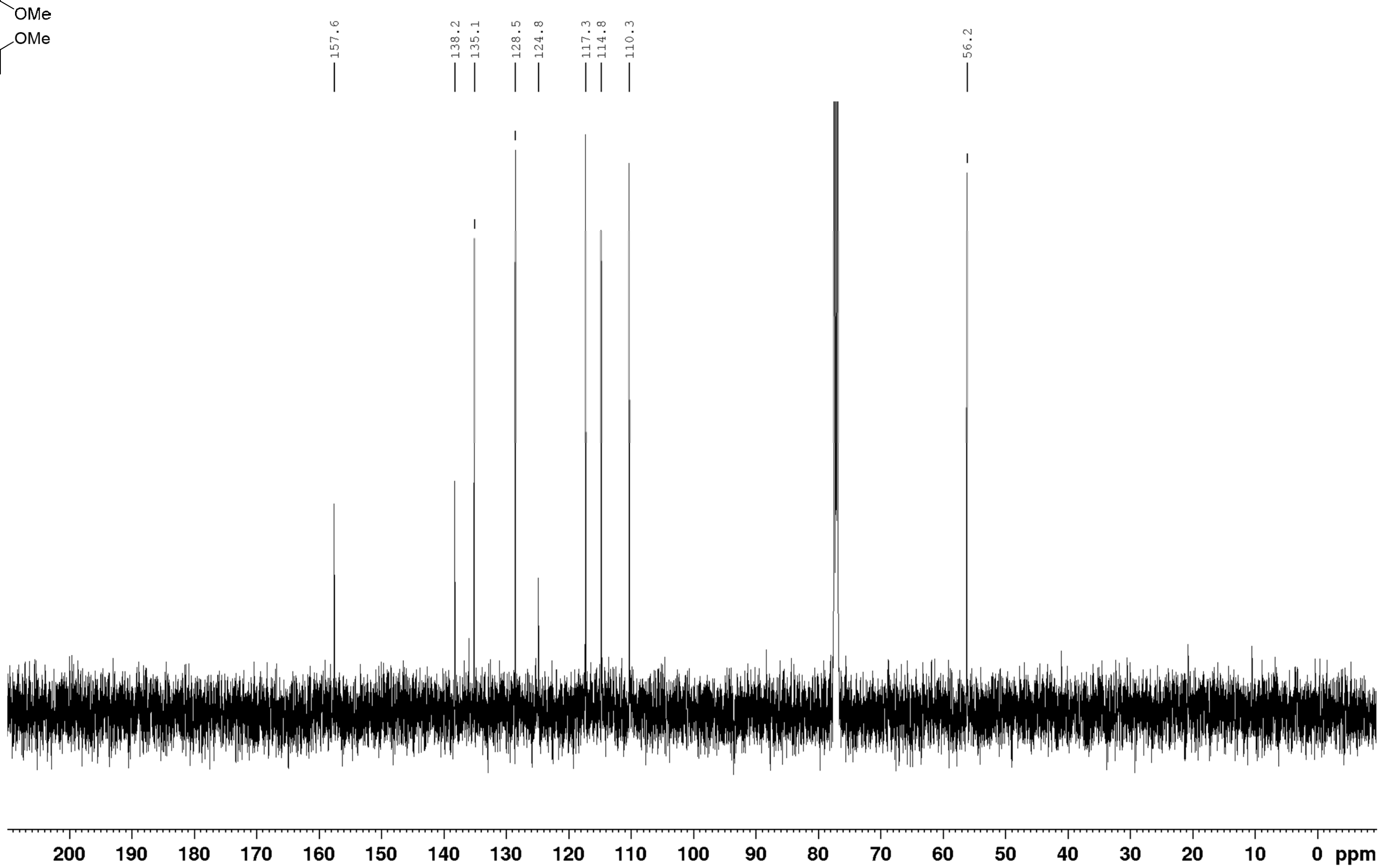

Figure S74. ^{1}H NMR (500 MHz, $CDCl_3$) of *rac*-2,2'-Dimethoxy-6,6'-bis(2-methylprop-1-en-1-yl)-1,1'-biphenyl (*rac*-**S13**).

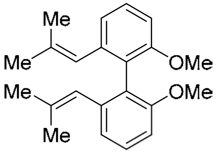

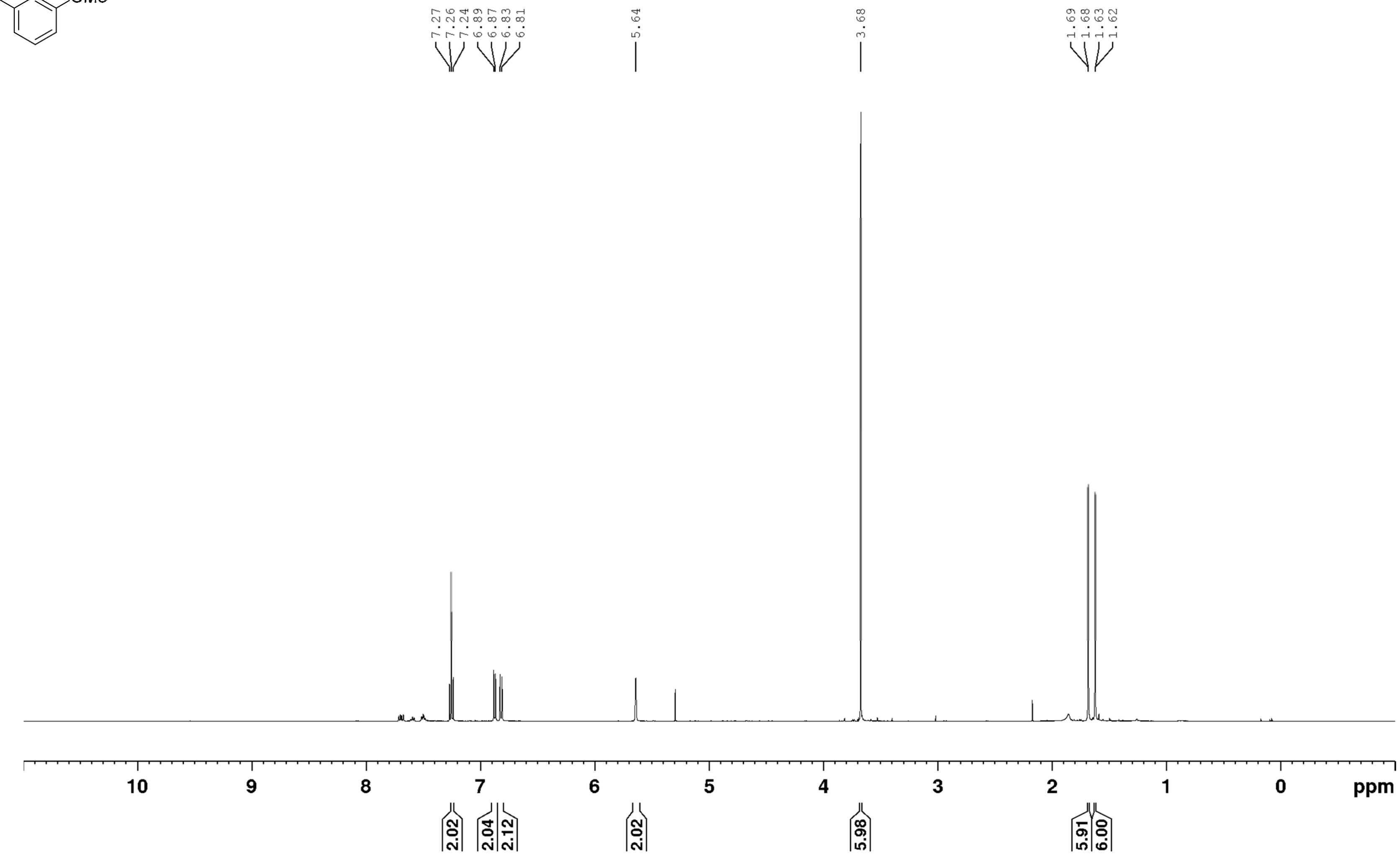

Figure S75. $^{13}C\{^{1}H\}$ NMR (126 MHz, $CDCl_3$) of *rac*-2,2'-Dimethoxy-6,6'-bis(2-methylprop-1-en-1-yl)-1,1'-biphenyl (*rac*-**S13**).

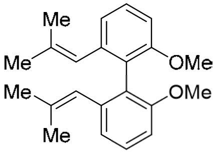

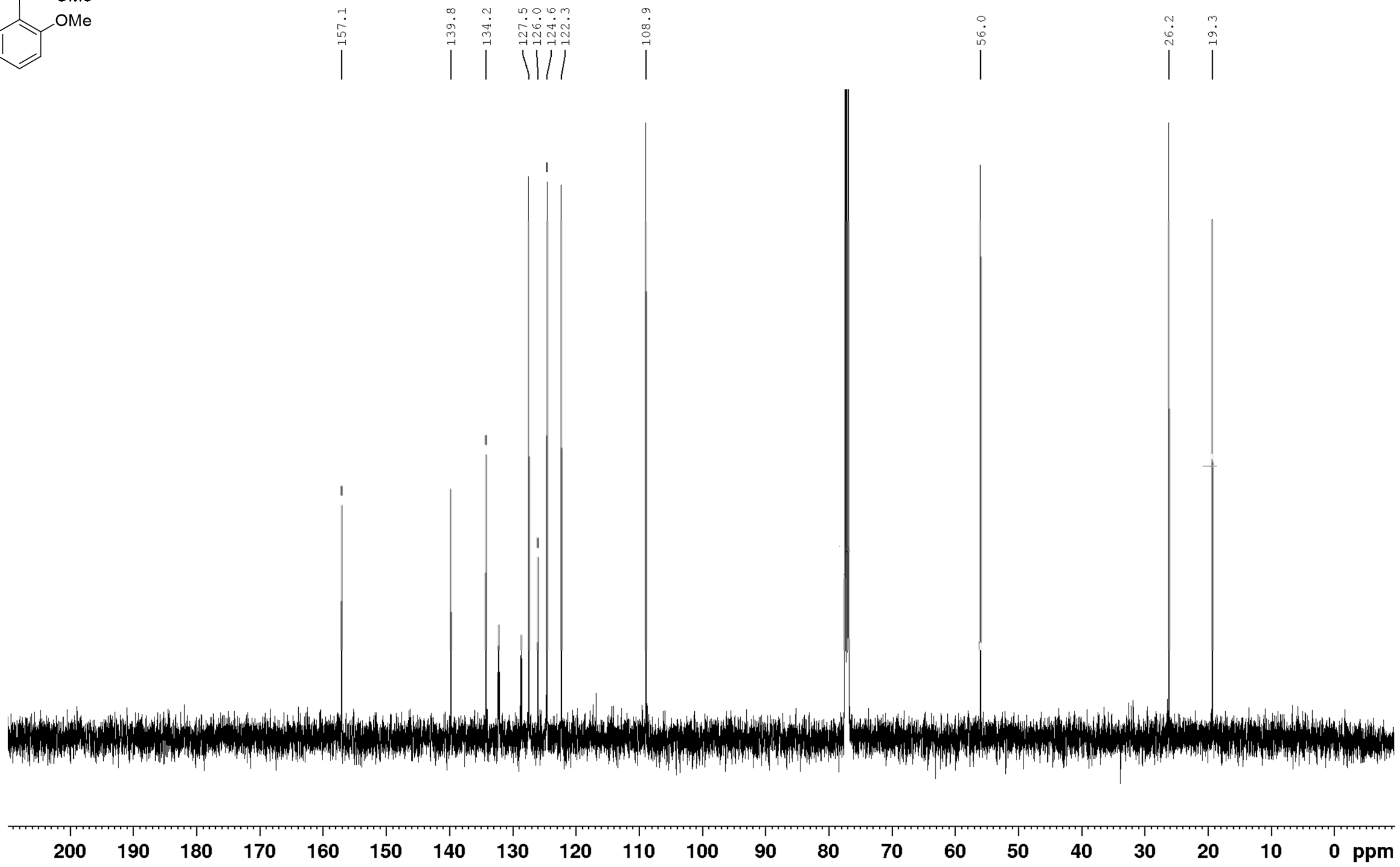

Figure S76. ^{1}H NMR (500 MHz, $CDCl_3$) of *rac*-2,2'-Diisobutyl-6,6'-dimethoxy-1,1'-biphenyl (*rac*-**S15**).

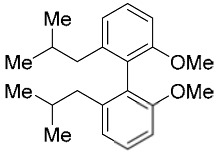

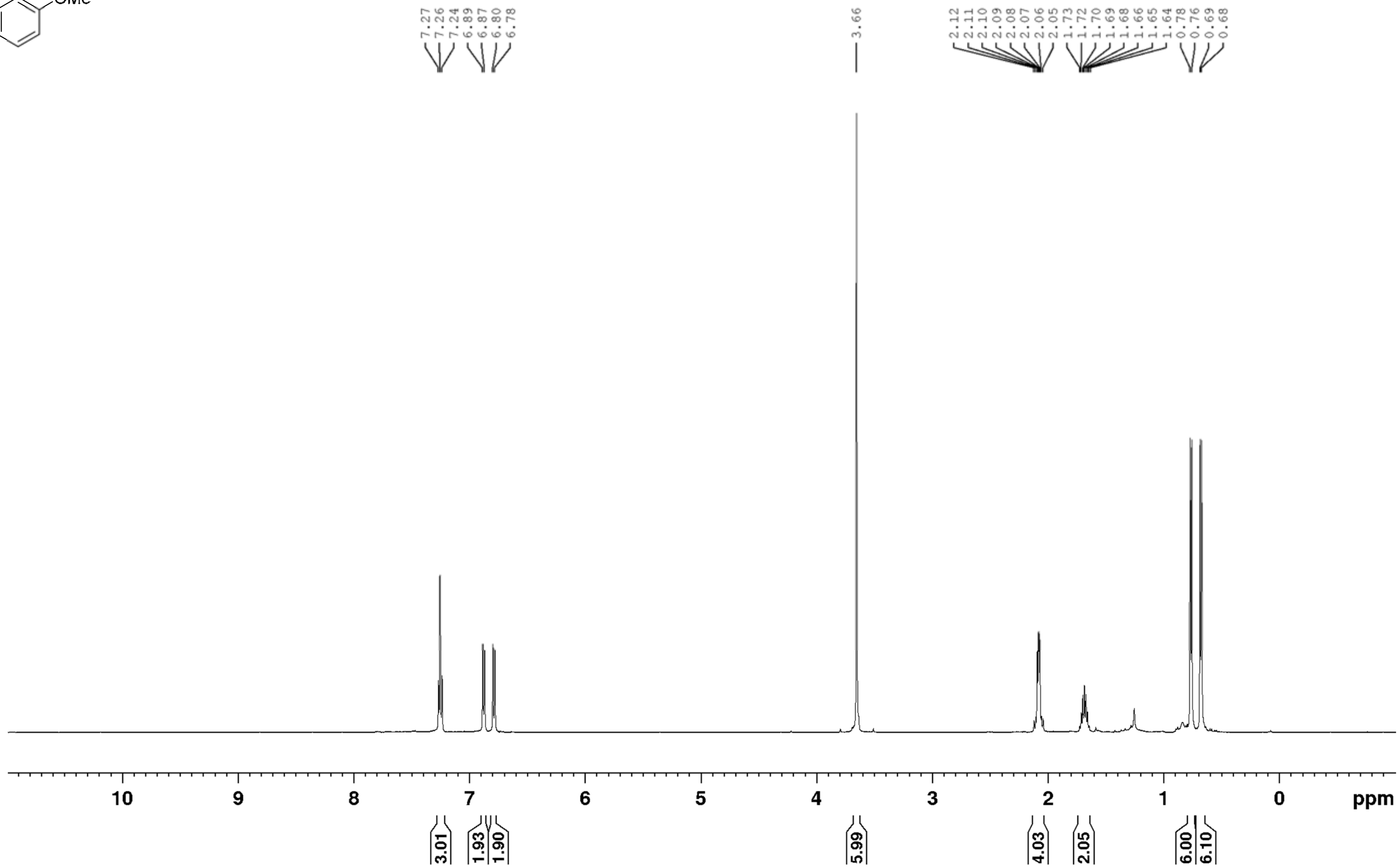

Figure S77. $^{13}C\{^1H\}$ NMR (126 MHz, $CDCl_3$) of *rac*-2,2'-Diisobutyl-6,6'-dimethoxy-1,1'-biphenyl (*rac*-**S15**).

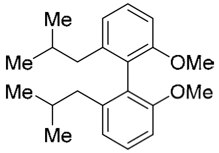

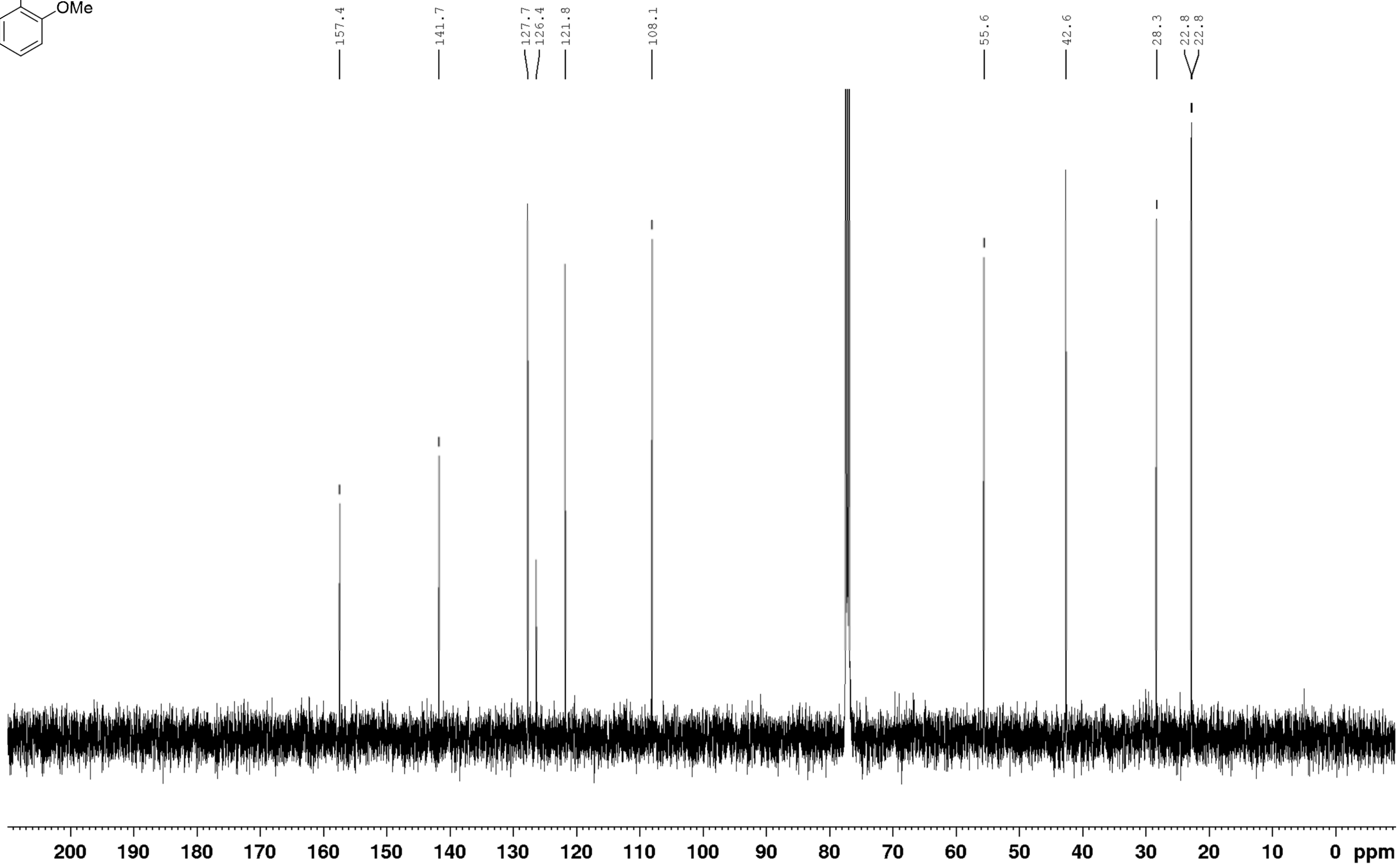

Figure S78. ^1H NMR (500 MHz, $CDCl_3$) of *rac*-6,6'-Diisobutyl-[1,1'-biphenyl]-2,2'-diol (*rac*-**S17**). (**tert*-buytl methylether)

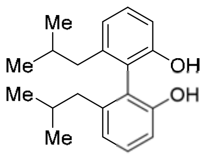

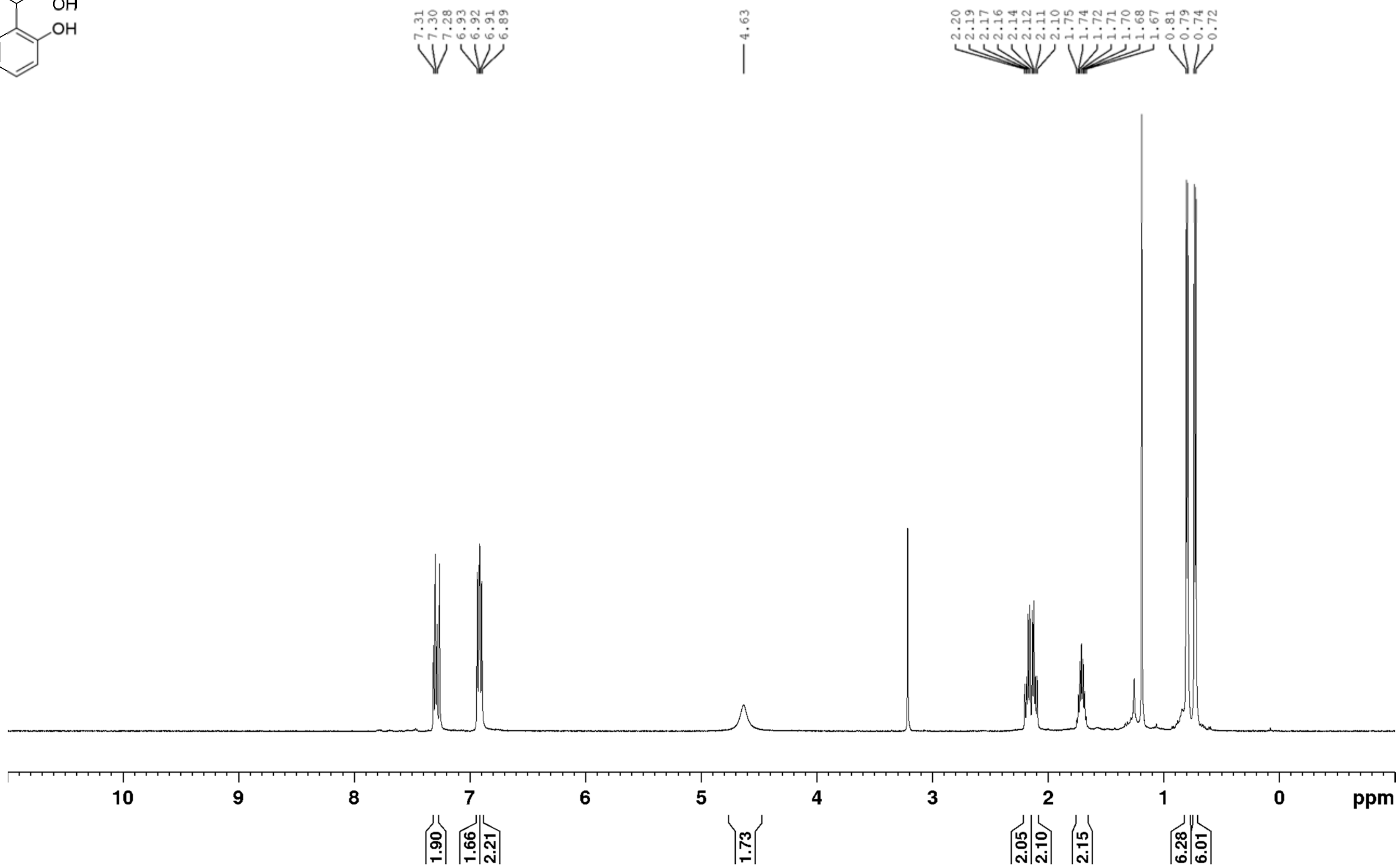

Figure S79. $^{13}C\{^{1}H\}$ NMR (126 MHz, $CDCl_3$) of *rac*-6,6'-Diisobutyl-[1,1'-biphenyl]-2,2'-diol (*rac*-**S17**). (**tert*-buytl methylether)

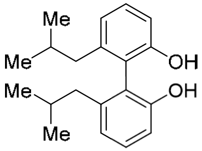

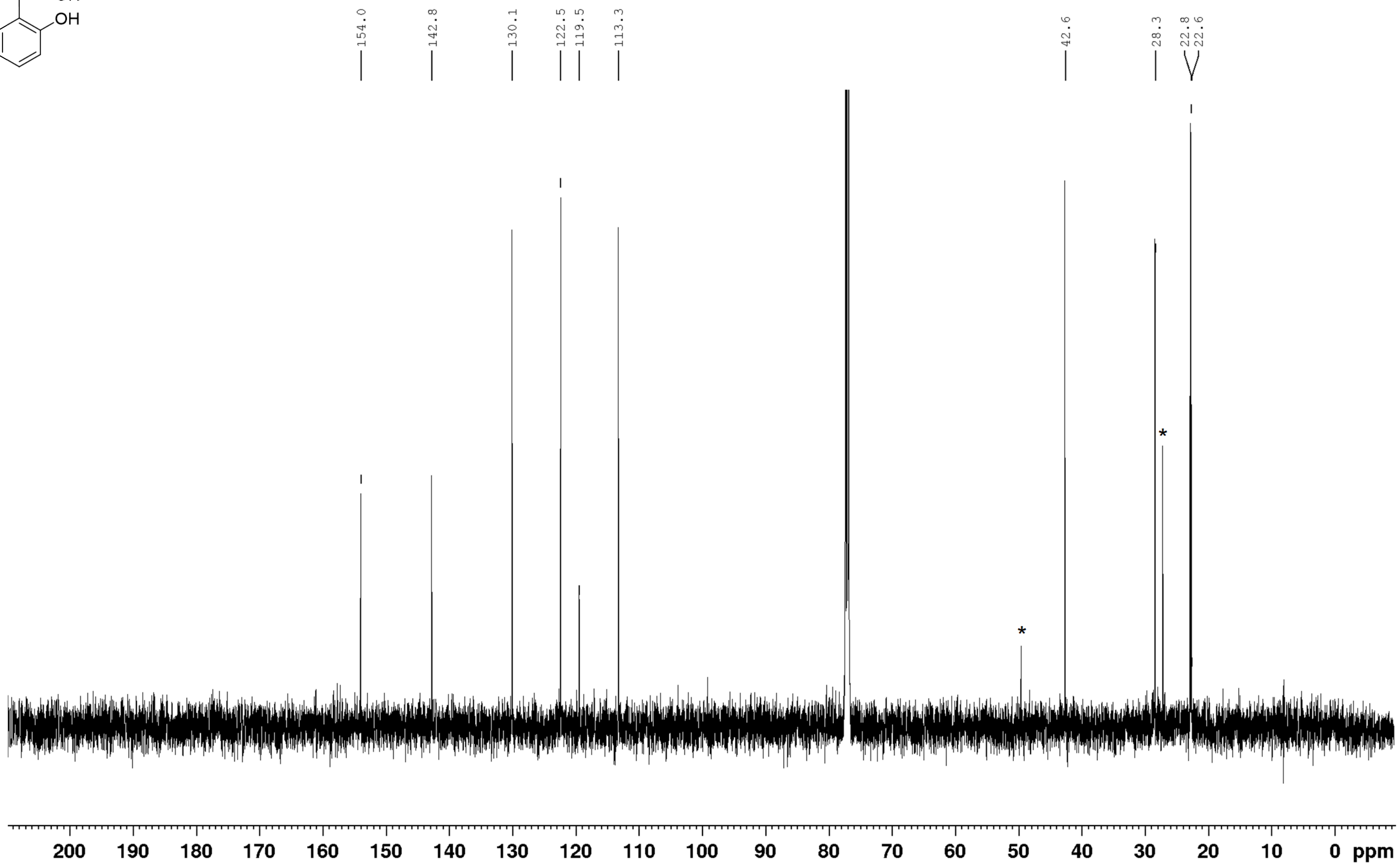

Figure S80. ^{1}H NMR (500 MHz, CD_2Cl_2) of (*S*)-[(2'-Methoxy-[1,1'-binaphthalen]-2-yl)oxy](methyl)diphenylsilane [(*S*)-**3ba**].

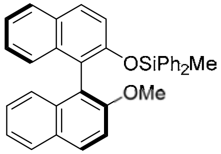

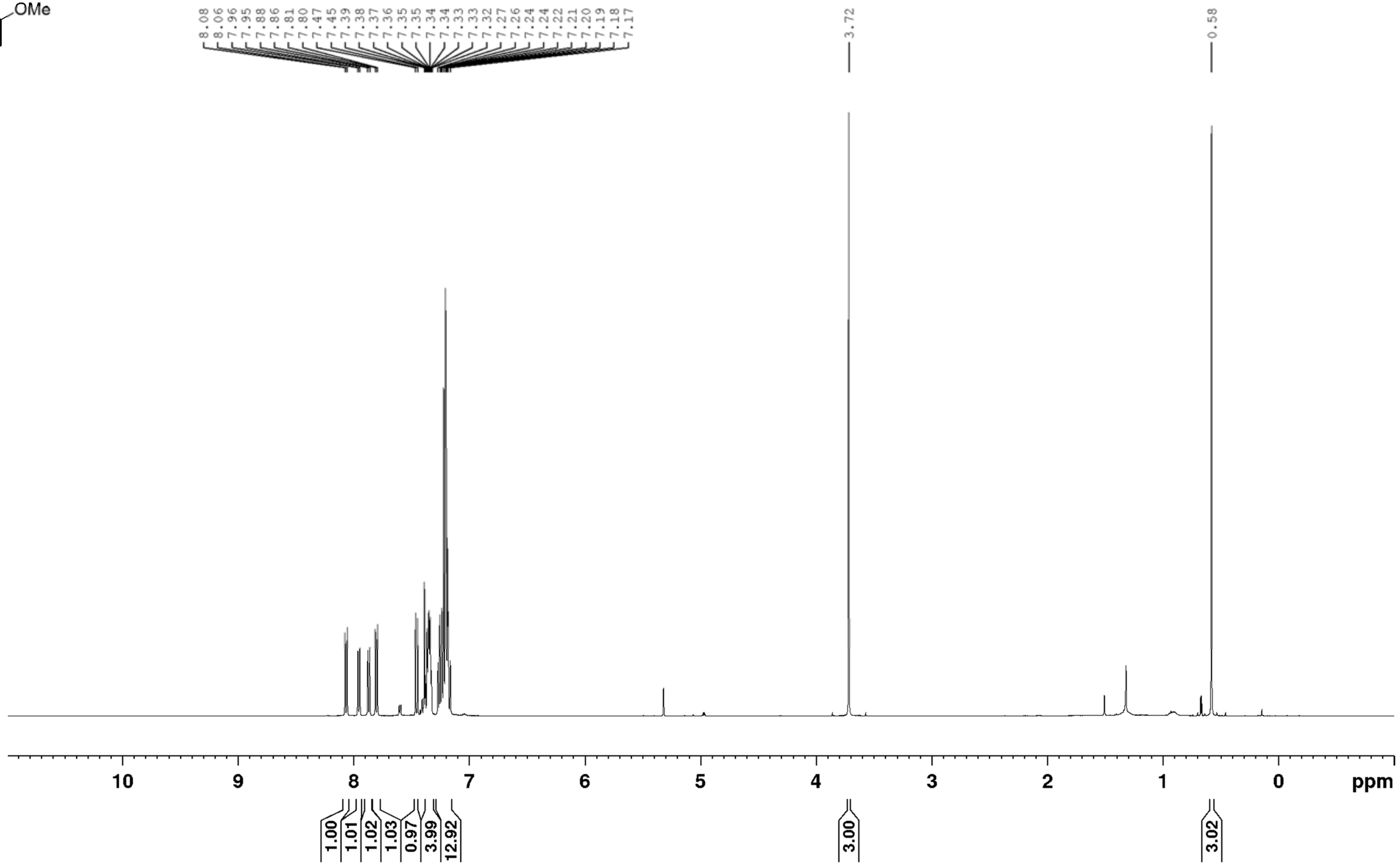

Figure S81. $^{13}C\{^{1}H\}$ NMR (126 MHz, CD_2Cl_2) of (*S*)-[(2'-Methoxy-[1,1'-binaphthalen]-2-yl)oxy](methyl)diphenylsilane [(*S*)-**3ba**].

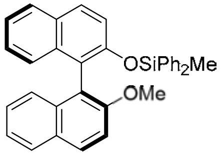

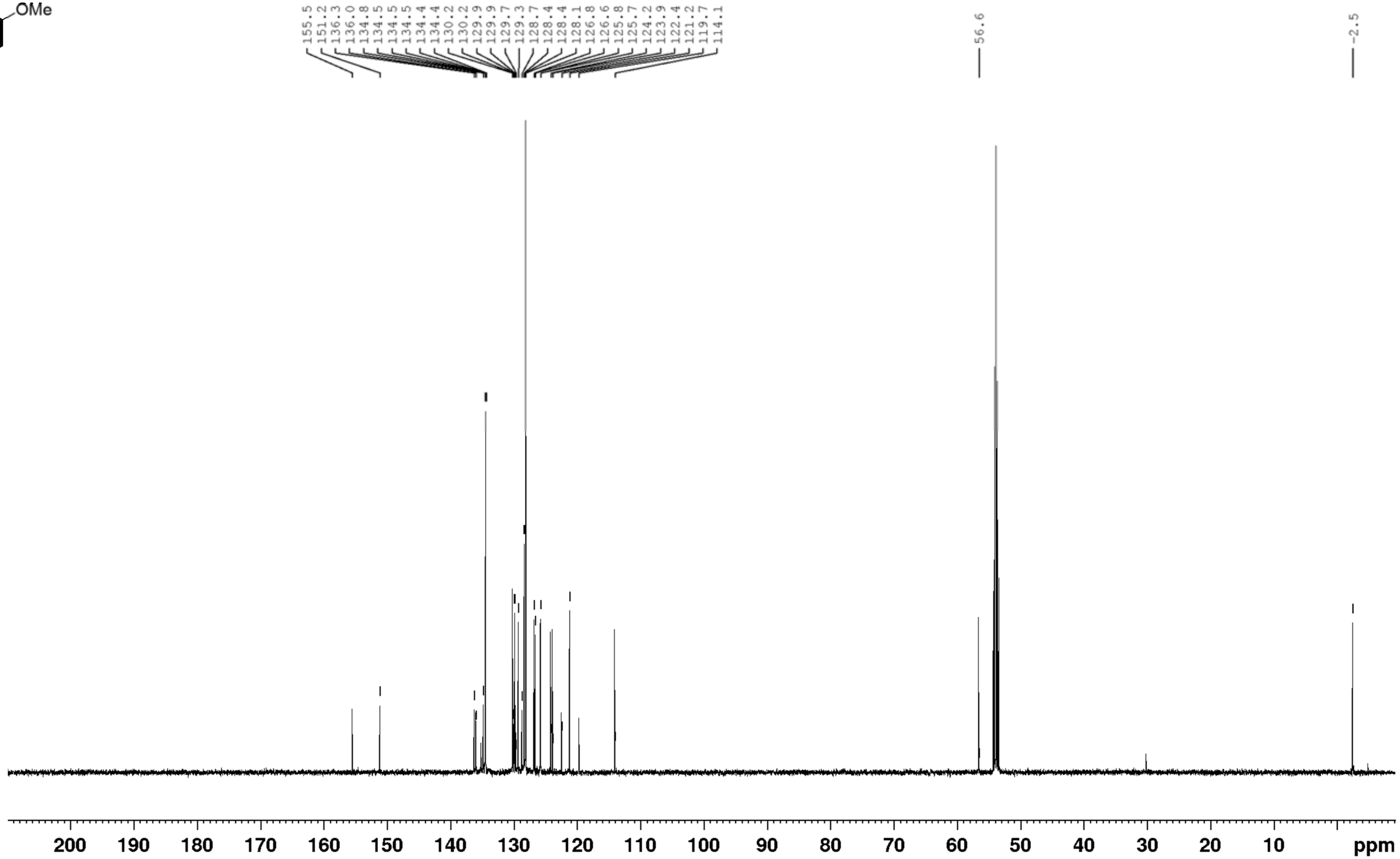

Figure S82. $^1H/^{29}Si$ HMQC NMR (500/99 MHz, CD_2Cl_2, optimized for J = 7 Hz) of (*S*)-[(2'-Methoxy-[1,1'-binaphthalen]-2-yl)oxy](methyl)-diphenylsilane [(*S*)-**3ba**].

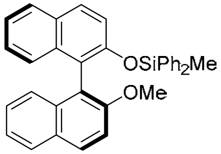

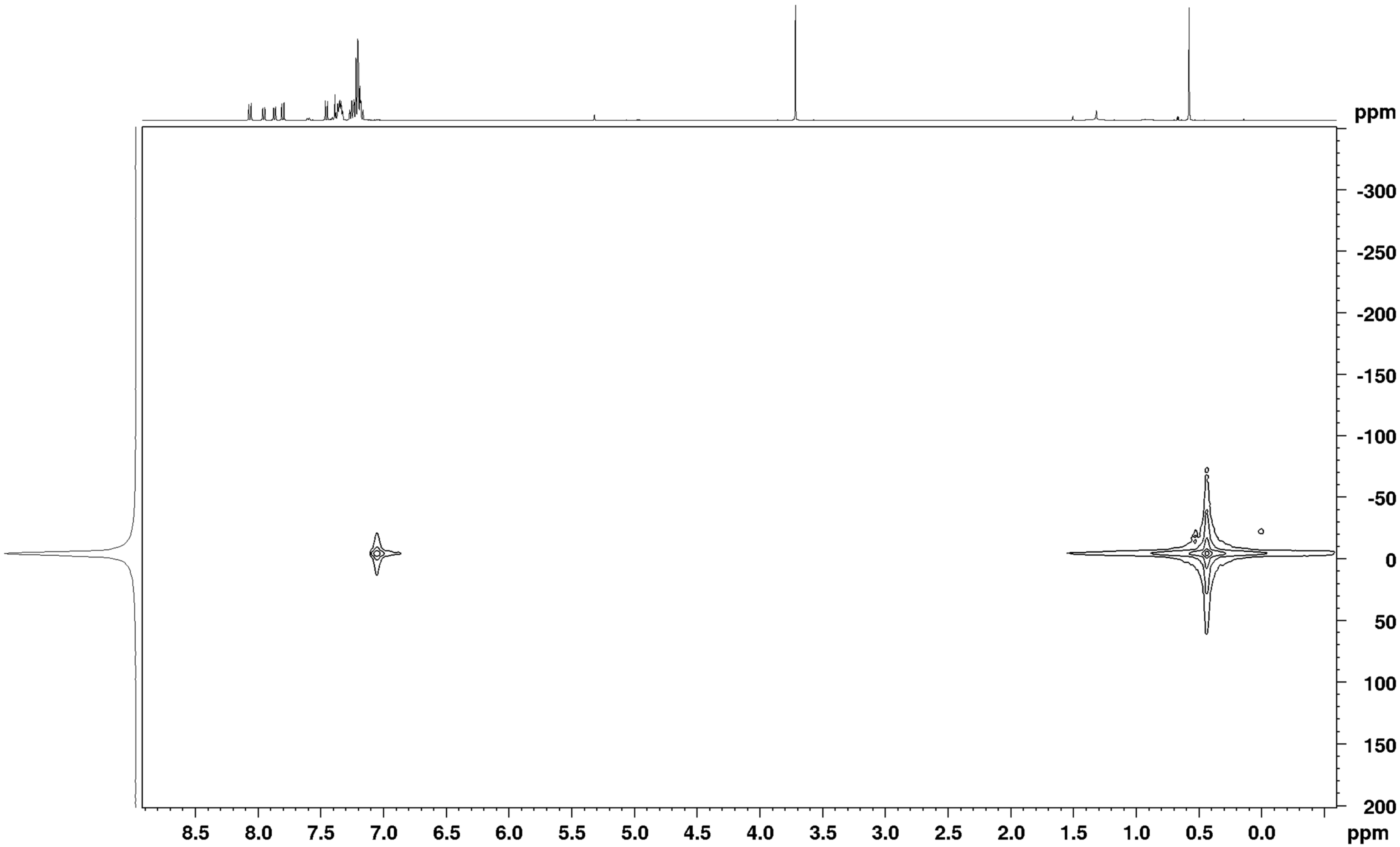

Figure S83. ^{1}H NMR (500 MHz, $CDCl_3$) of (*R*)-2'-Methoxy-[1,1'-binaphthalen]-2-ol [(*R*)-**1b**].

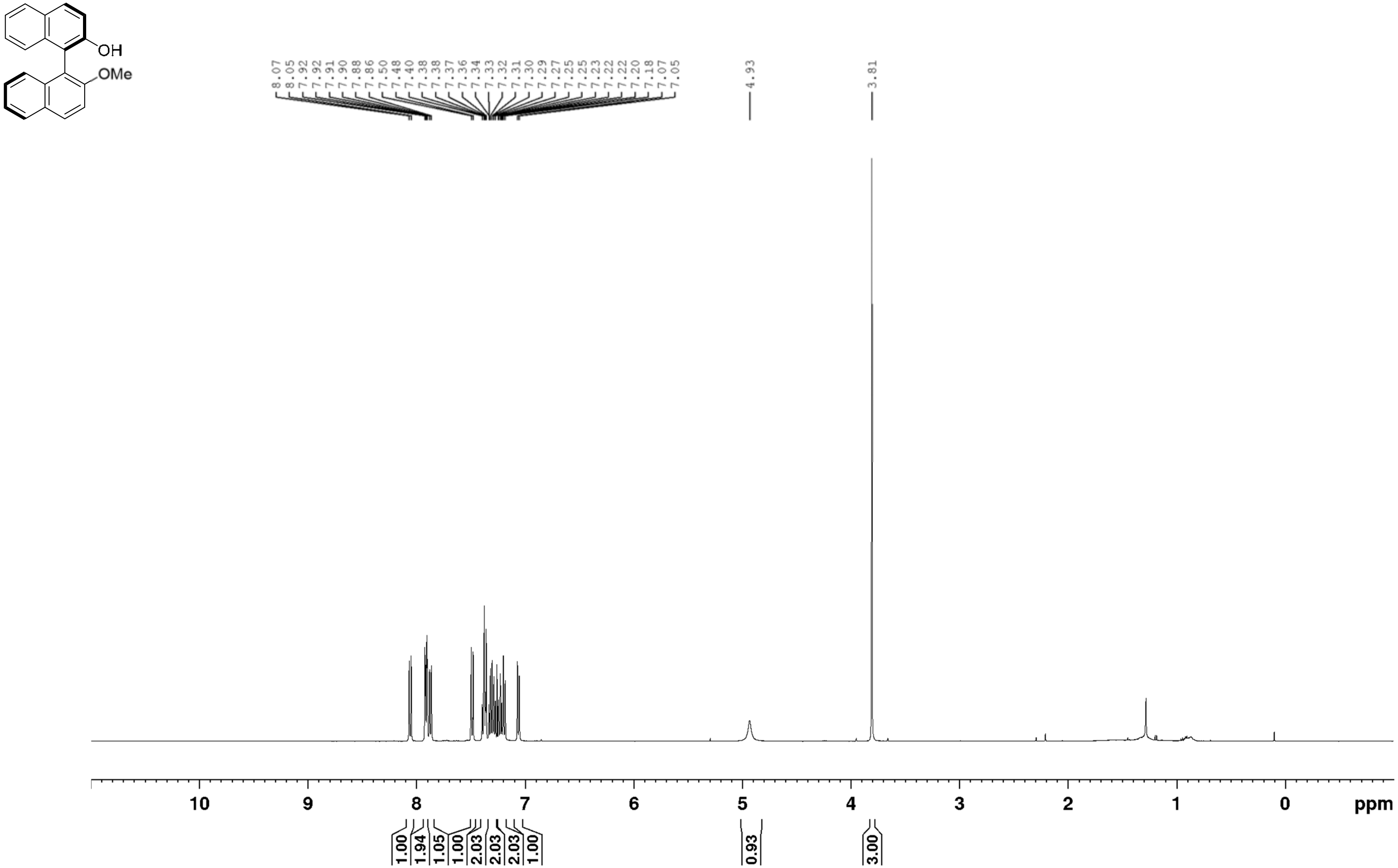

Figure S84. $^{13}C\{^{1}H\}$ NMR (126 MHz, $CDCl_3$) of (*R*)-2'-Methoxy-[1,1'-binaphthalen]-2-ol [(*R*)-**1b**].

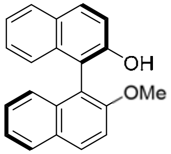

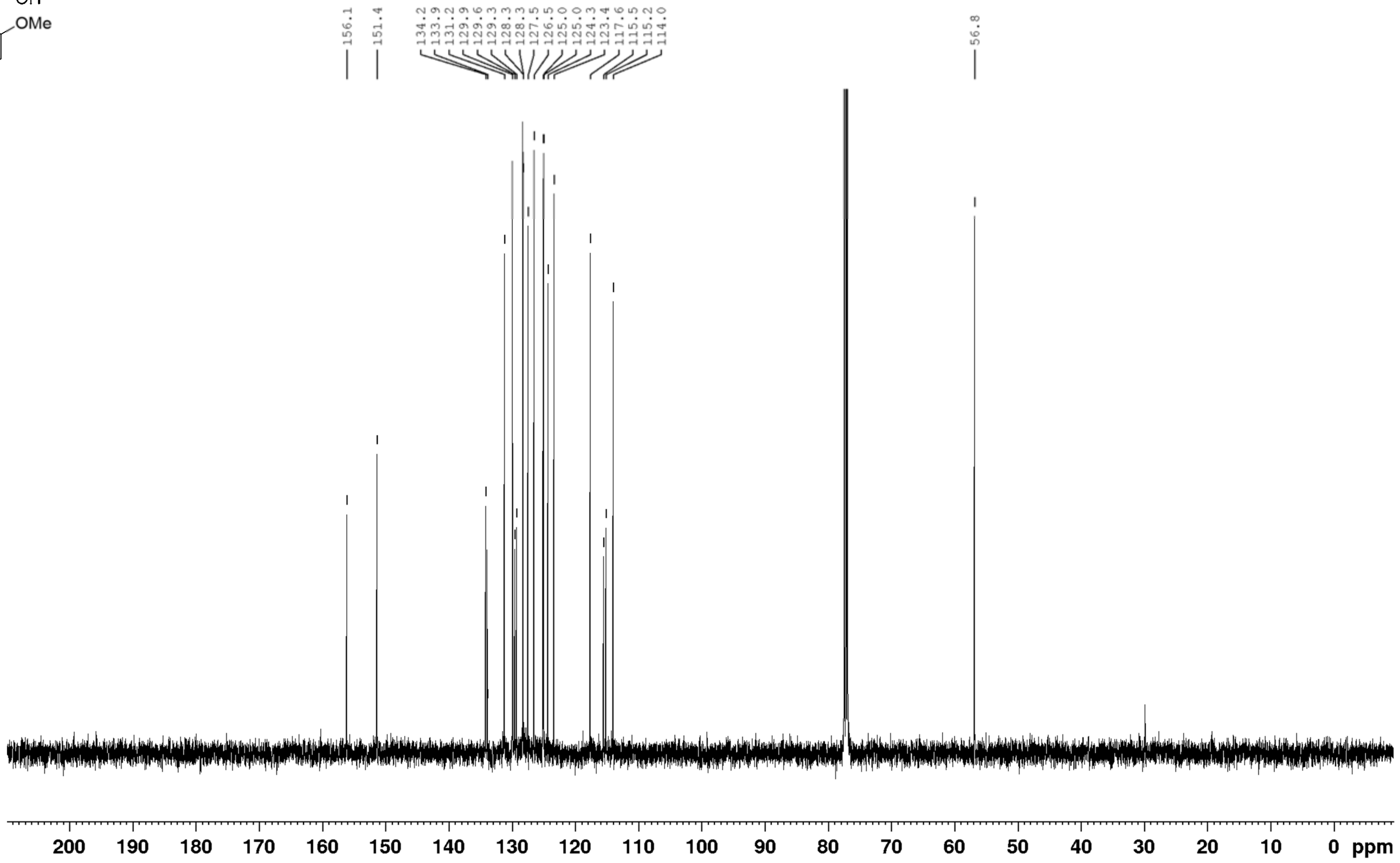

Figure S85. ^{1}H NMR (500 MHz, CD_2Cl_2) of (*S*)-[(2'-Isopropoxy-[1,1'-binaphthalen]-2-yl)oxy](methyl)diphenylsilane [(*S*)-**3ca**].

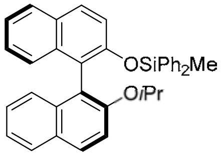

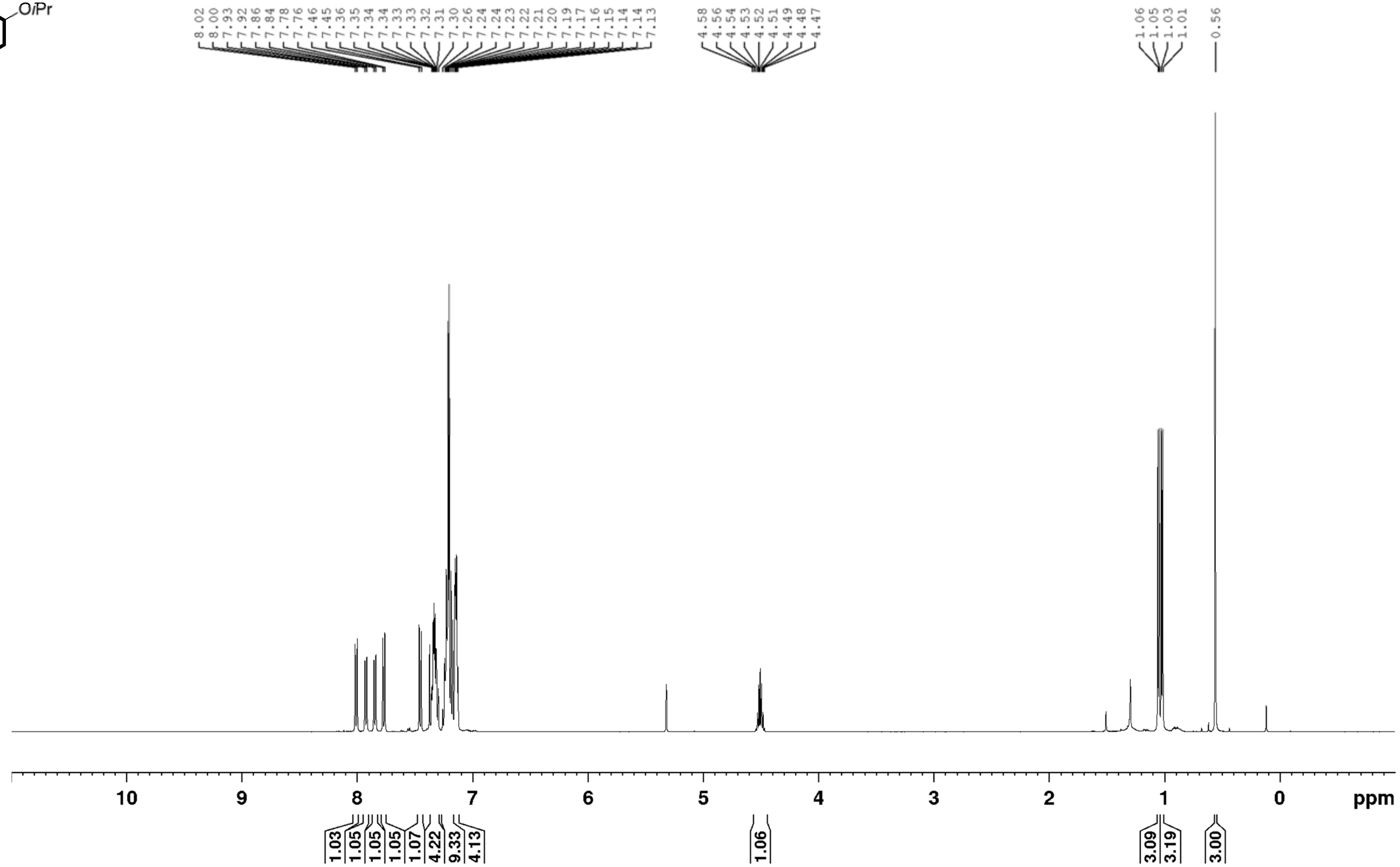

Figure S86. $^{13}C\{^{1}H\}$ NMR (126 MHz, CD_2Cl_2) of (*S*)-[(2'-Isopropoxy-[1,1'-binaphthalen]-2-yl)oxy](methyl)diphenylsilane [(*S*)-**3ca**].

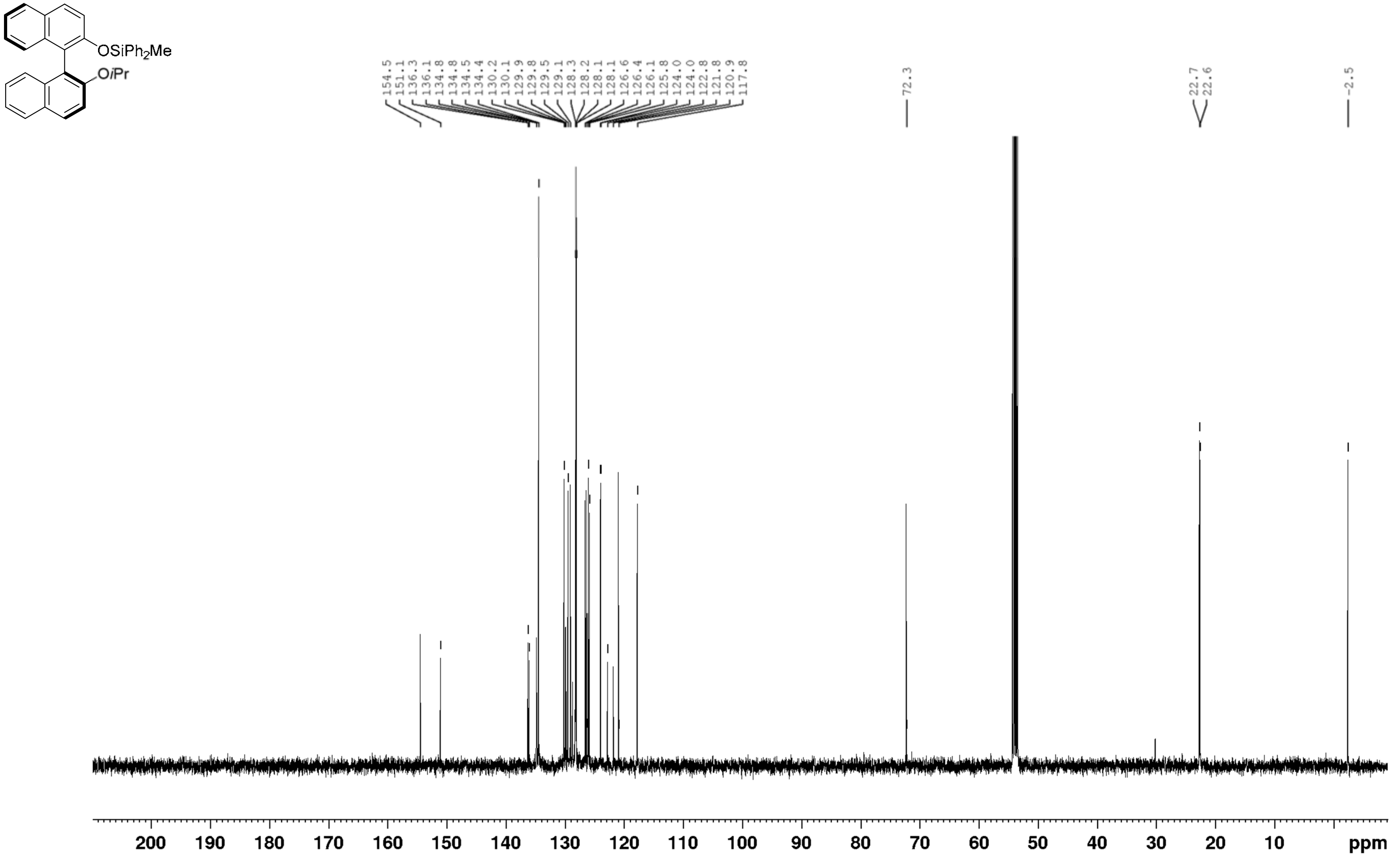

Figure S87. $^{1}H/^{29}Si$ HMQC NMR (500/99 MHz, CD_2Cl_2, optimized for J = 7 Hz) of (*S*)-[(2'-Isopropoxy-[1,1'-binaphthalen]-2-yl)oxy](methyl)-diphenylsilane [(*S*)-**3ca**].

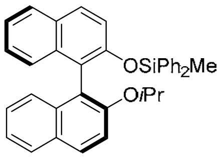

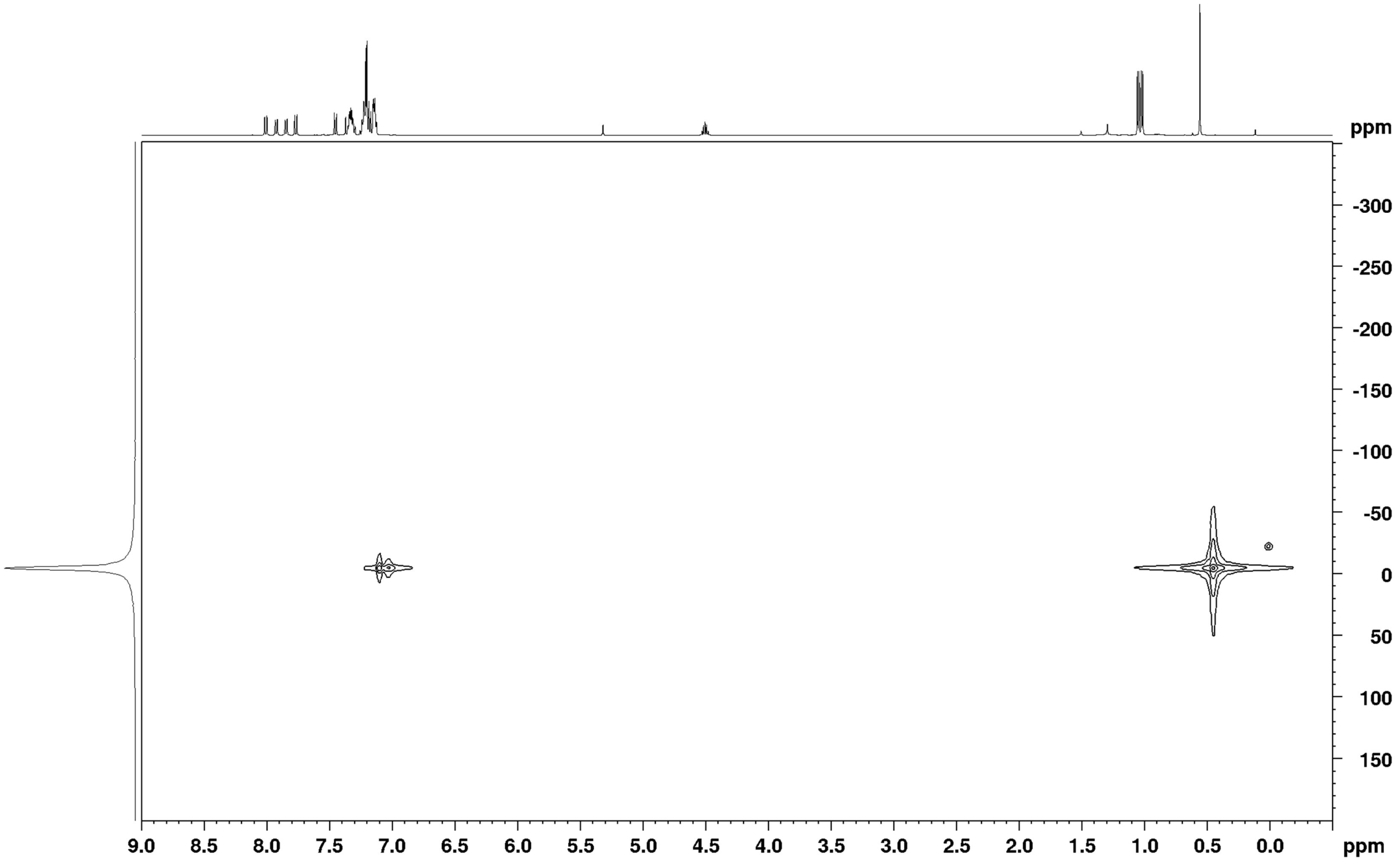

Figure S88. ^{1}H NMR (500 MHz, $CDCl_3$) of (*R*)-2'-Isopropoxy-[1,1'-binaphthalen]-2-ol [(*R*)-**1c**].

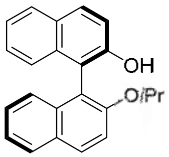

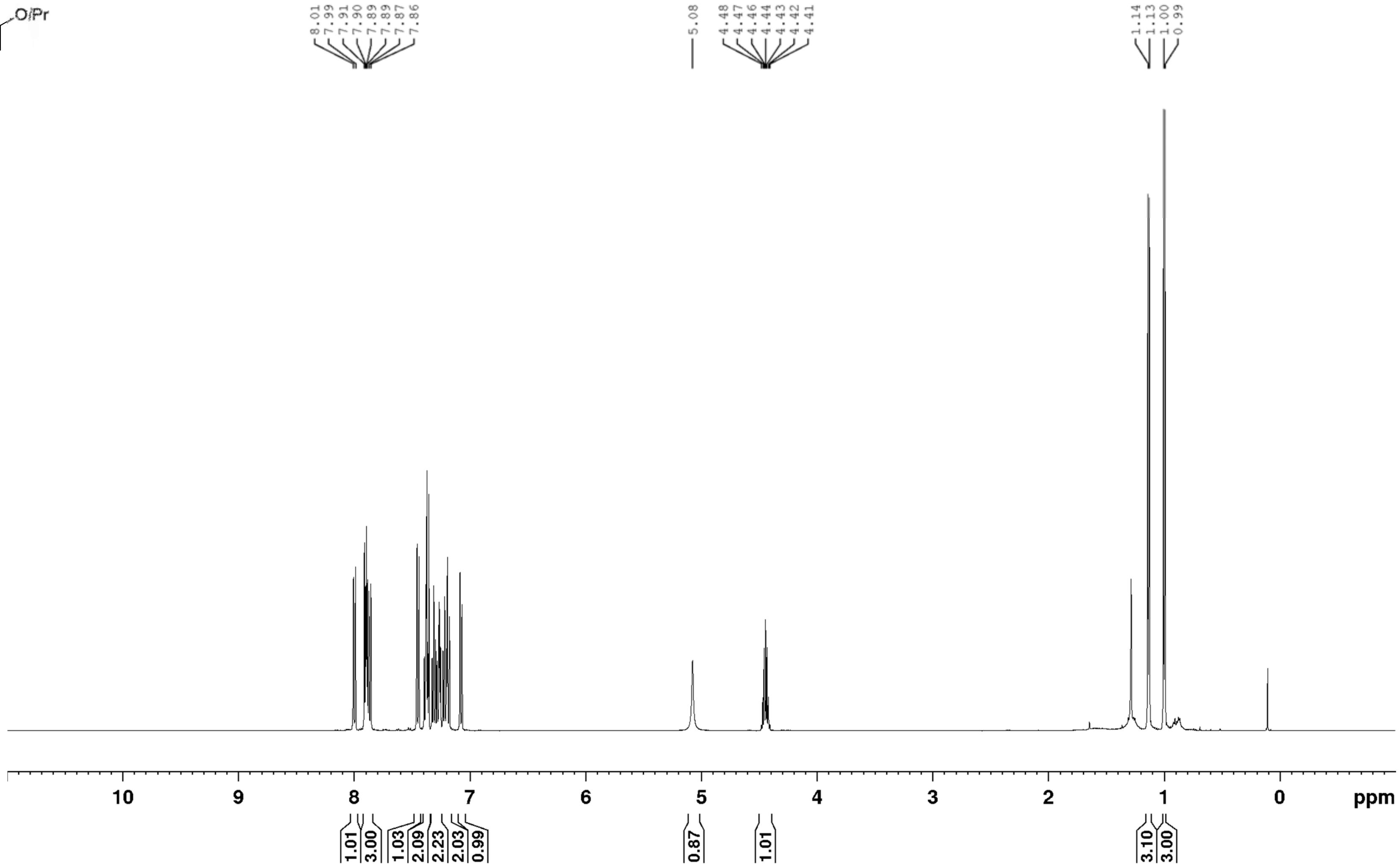

Figure S89. $^{13}C\{^{1}H\}$ NMR (126 MHz, $CDCl_3$) of (*R*)-2'-Isopropoxy-[1,1'-binaphthalen]-2-ol [(*R*)-**1c**].

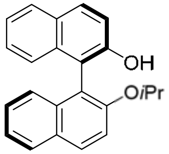

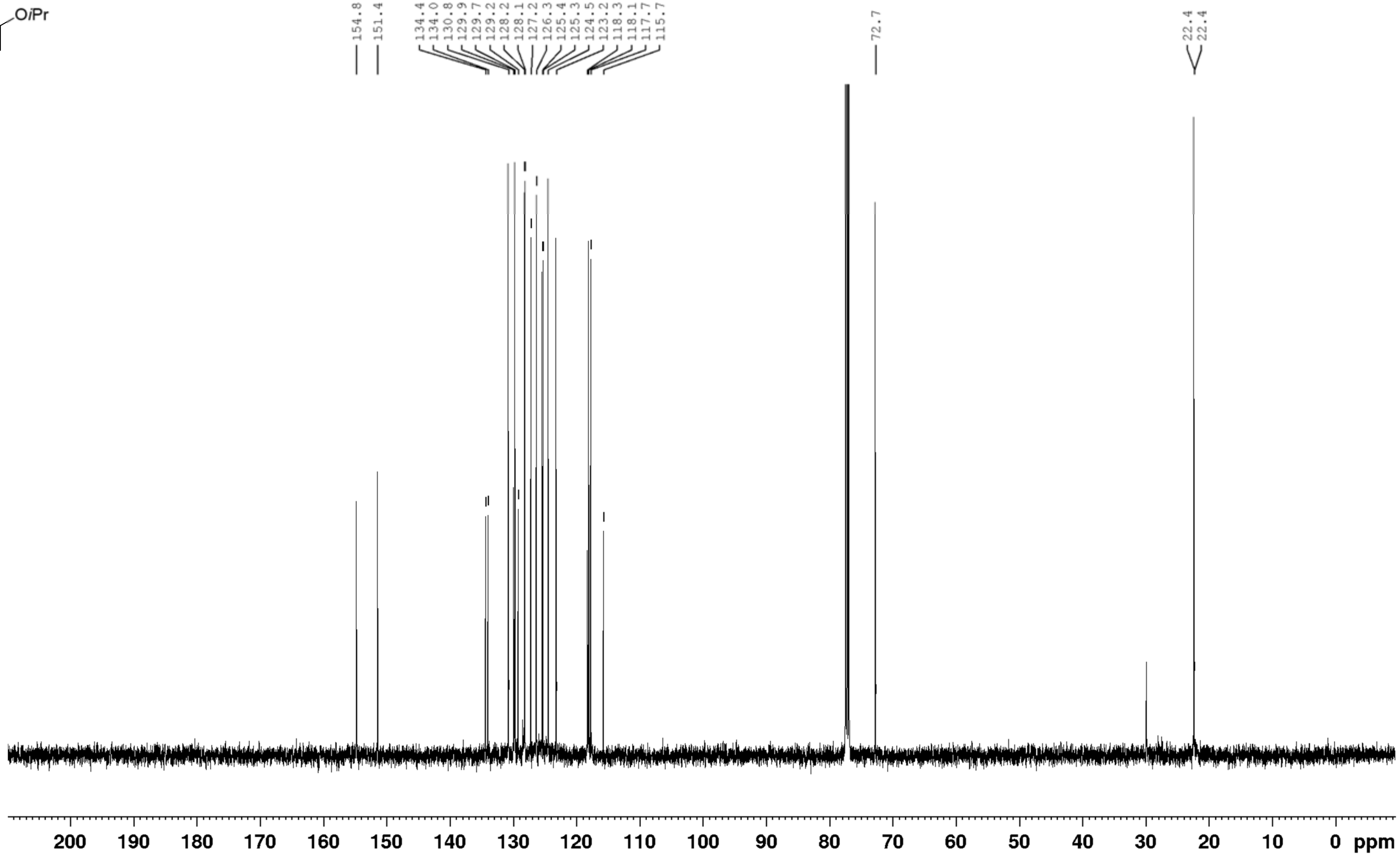

Figure S90. ^{1}H NMR (500 MHz, CD_2Cl_2) of (*S*)-[(2'-(Benzyloxy)-[1,1'-binaphthalen]-2-yl)oxy](methyl)diphenylsilane [(*S*)-**3da**].

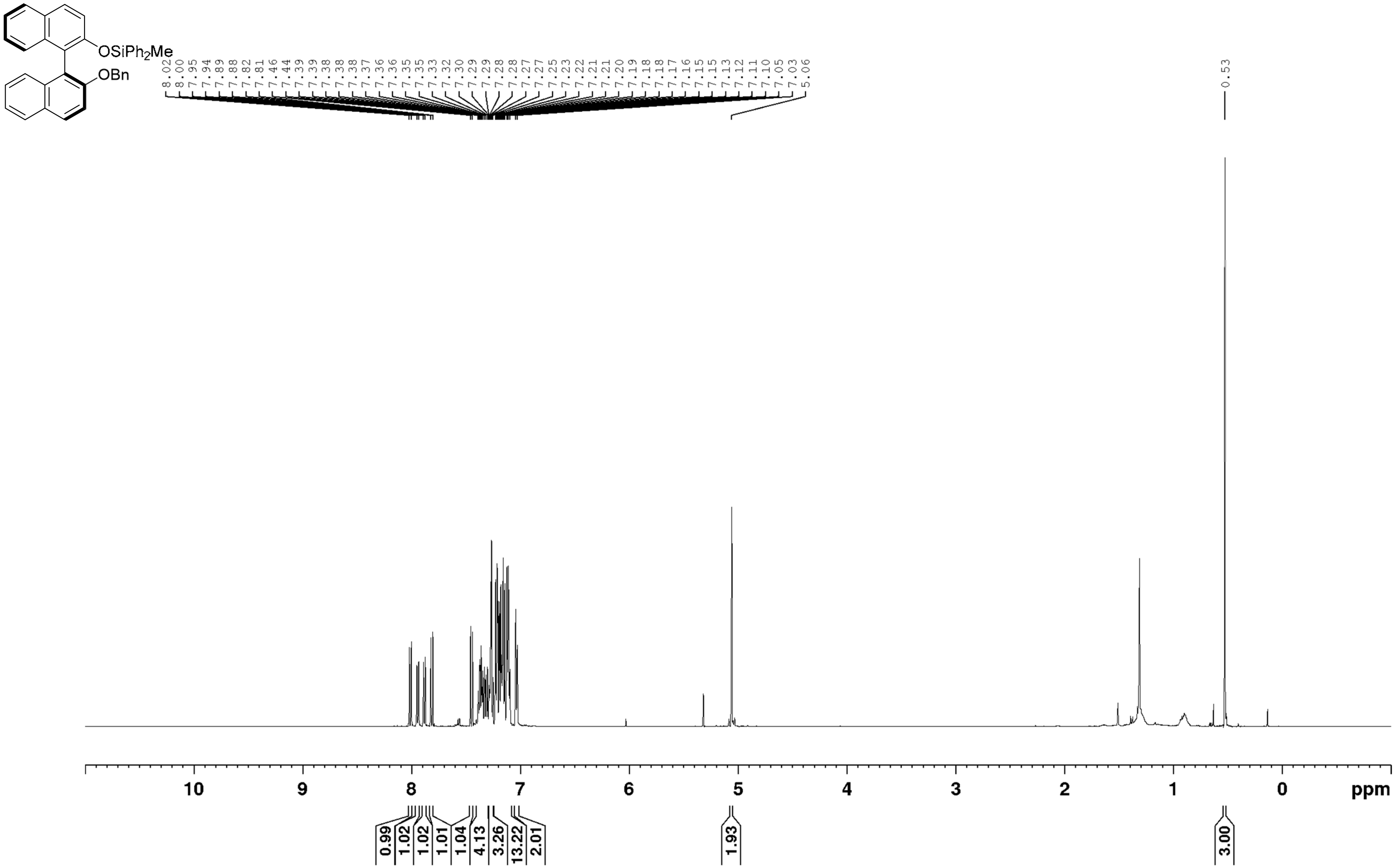

Figure S91. $^{13}C\{^{1}H\}$ NMR (126 MHz, CD_2Cl_2) of (*S*)-[(2'-(Benzyloxy)-[1,1'-binaphthalen]-2-yl)oxy](methyl)diphenylsilane [(*S*)-**3da**].

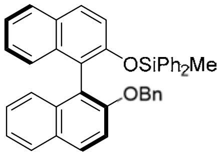

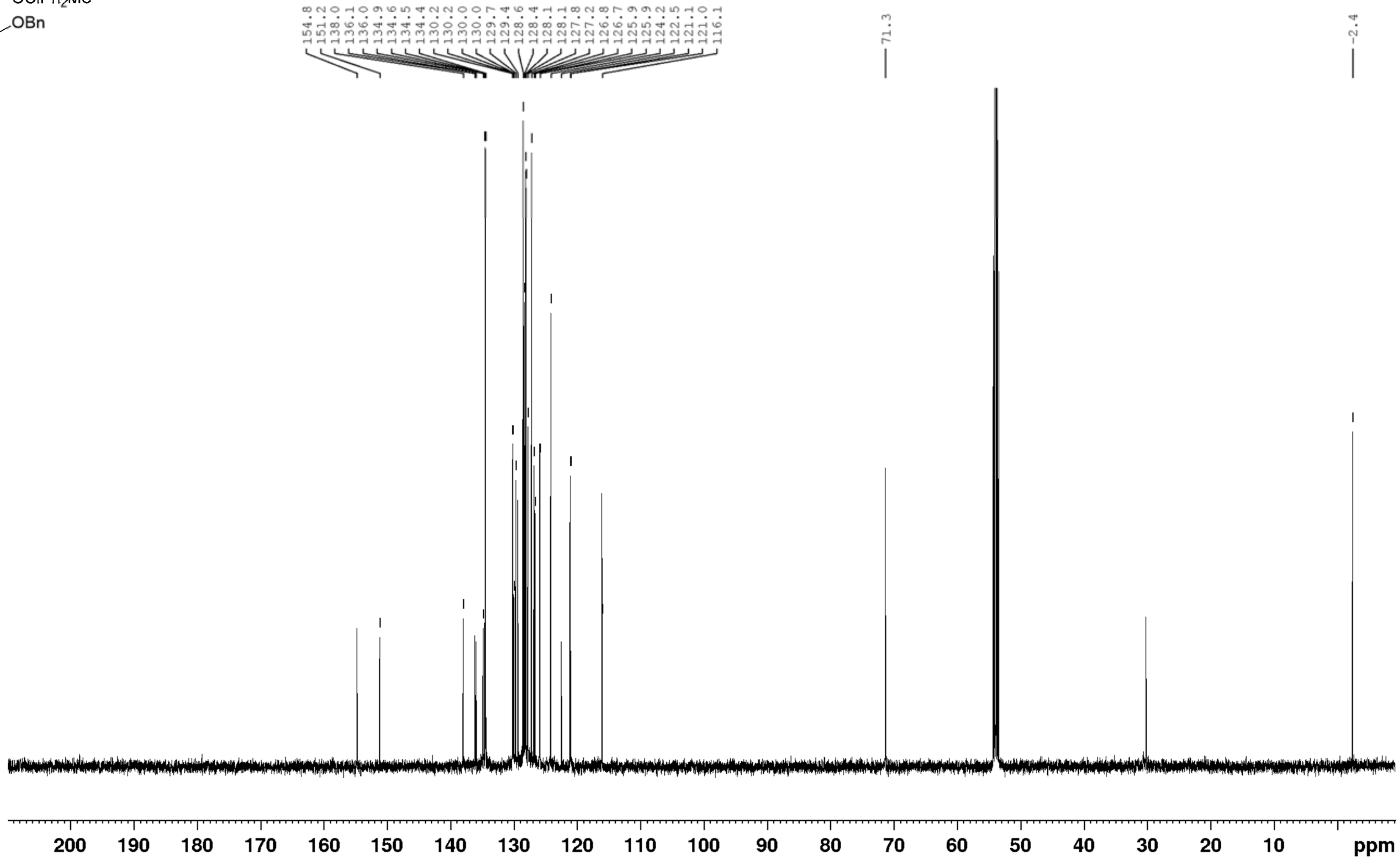

Figure S92. $^1H/^{29}Si$ HMQC NMR (500/99 MHz, CD_2Cl_2, optimized for J = 7 Hz) of (*S*)-[(2'-(Benzyloxy)-[1,1'-binaphthalen]-2-yl)oxy]-(methyl)diphenylsilane [(*S*)-**3da**].

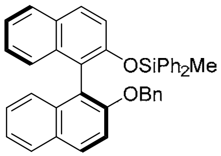

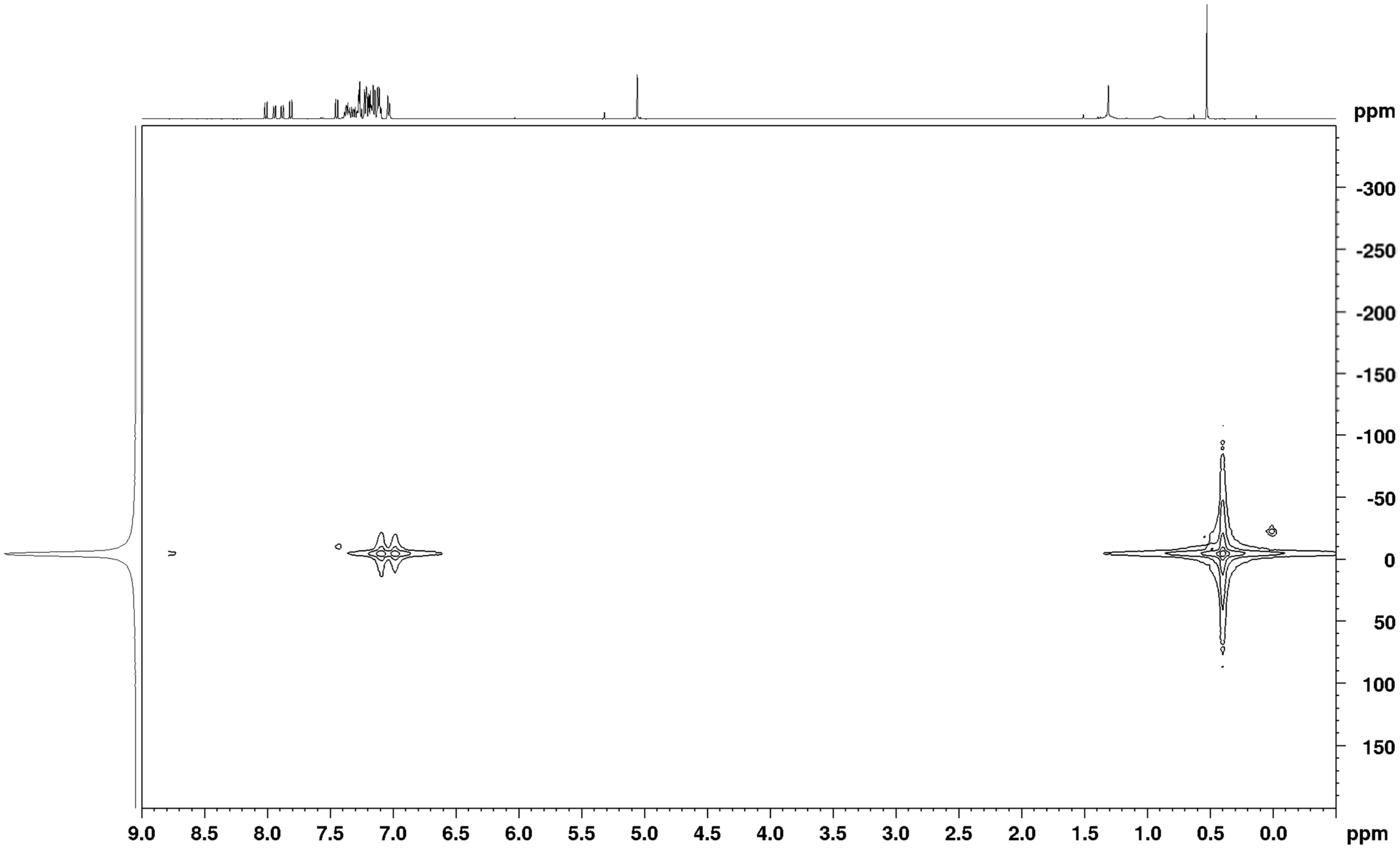

Figure S93. ^{1}H NMR (500 MHz, $CDCl_3$) of (*R*)-2'-(Benzyloxy)-[1,1'-binaphthalen]-2-ol [(*R*)-**1d**]. (*H grease)

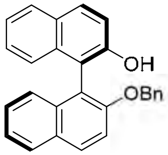

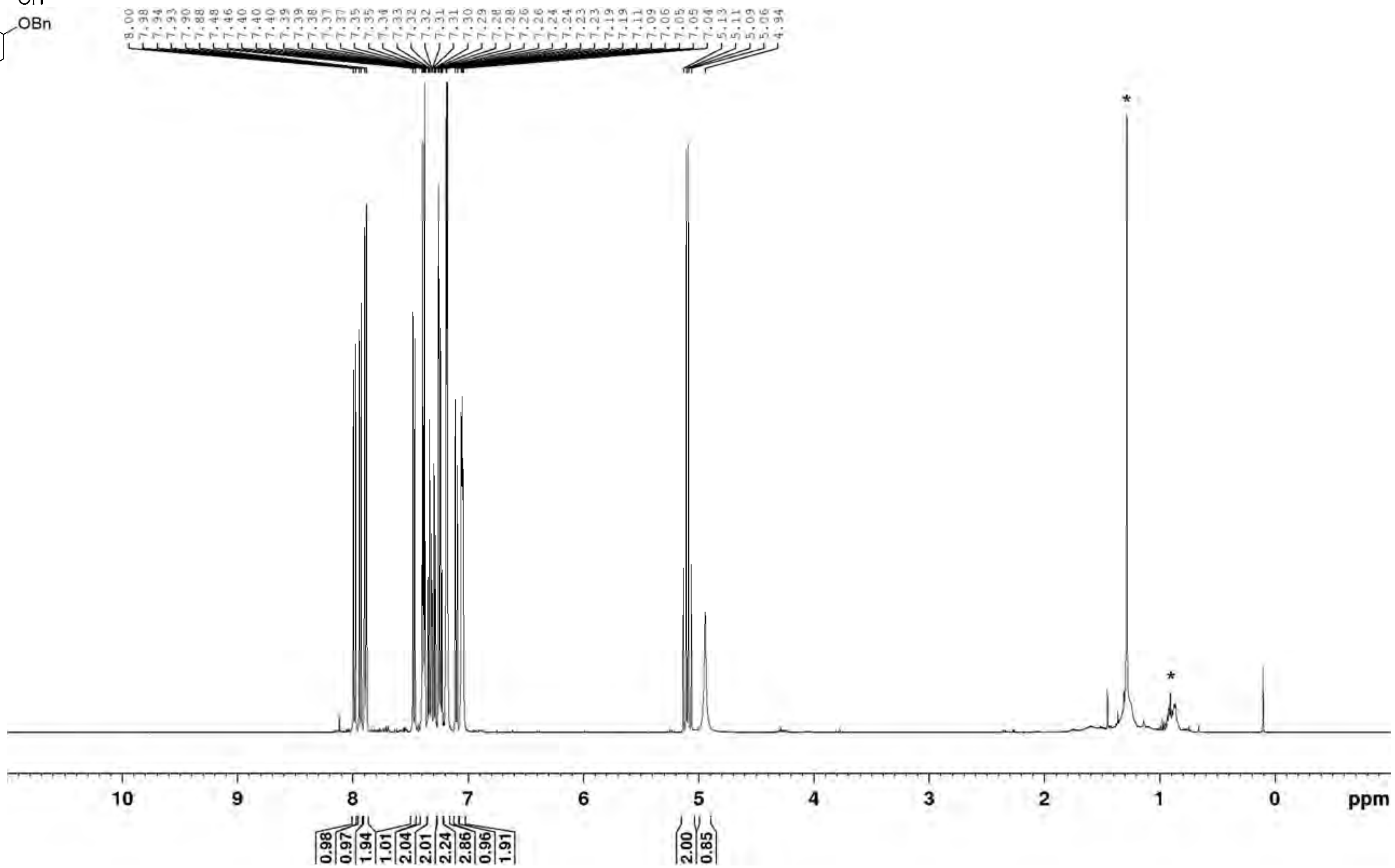

Figure S94. $^{13}C\{^{1}H\}$ NMR (126 MHz, $CDCl_3$) of (*R*)-2'-(Benzyloxy)-[1,1'-binaphthalen]-2-ol [(*R*)-**1d**]. (*H grease)

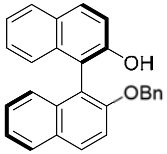

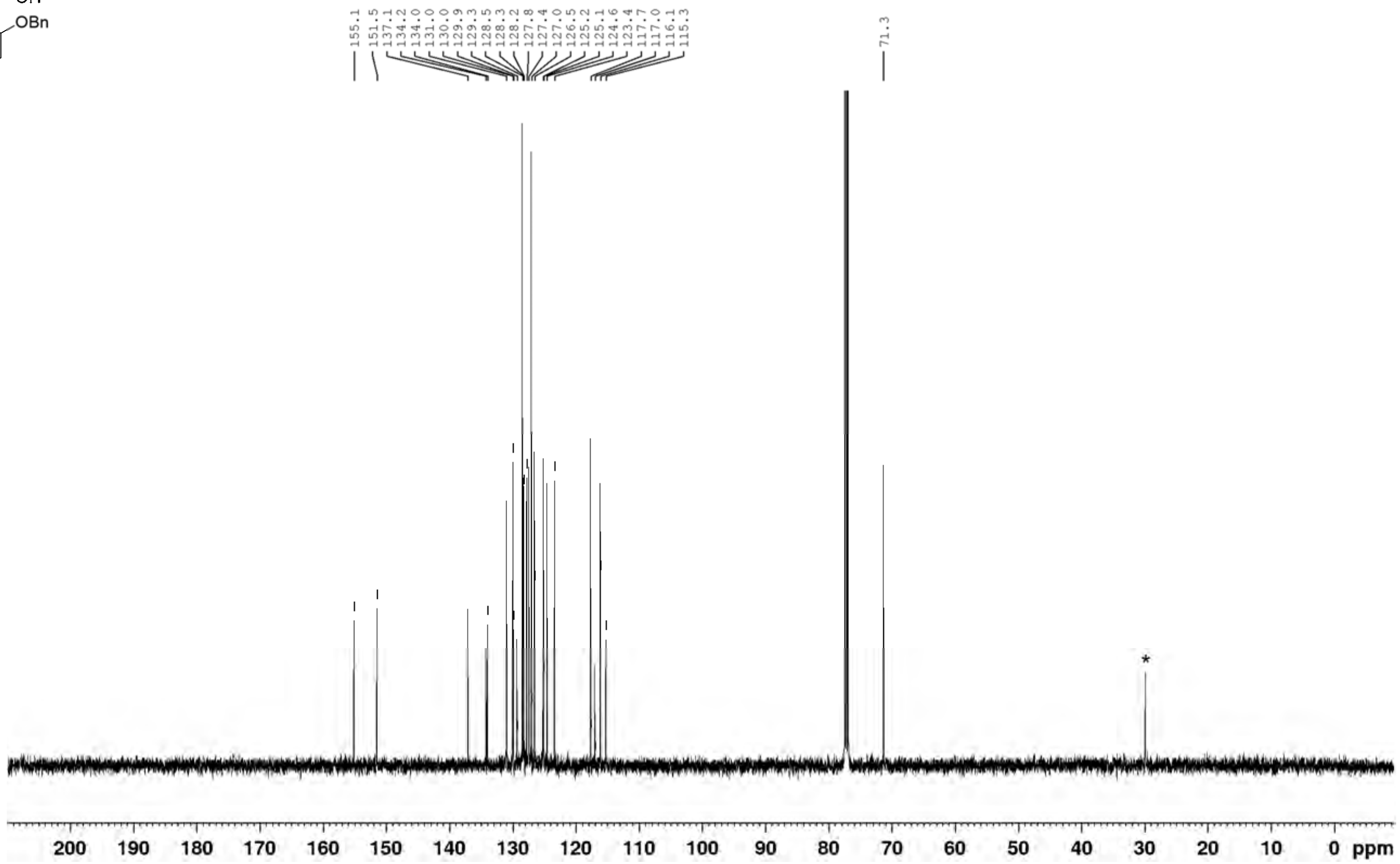

Figure S95. ^{1}H NMR (500 MHz, CD_2Cl_2) of (*S*)-[(2'-Isopropoxy-5,5',6,6',7,7',8,8'-octahydro-[1,1'-binaphthalen]-2-yl)oxy](methyl)diphenylsilane [(*S*)-**3ka**].

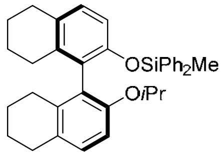

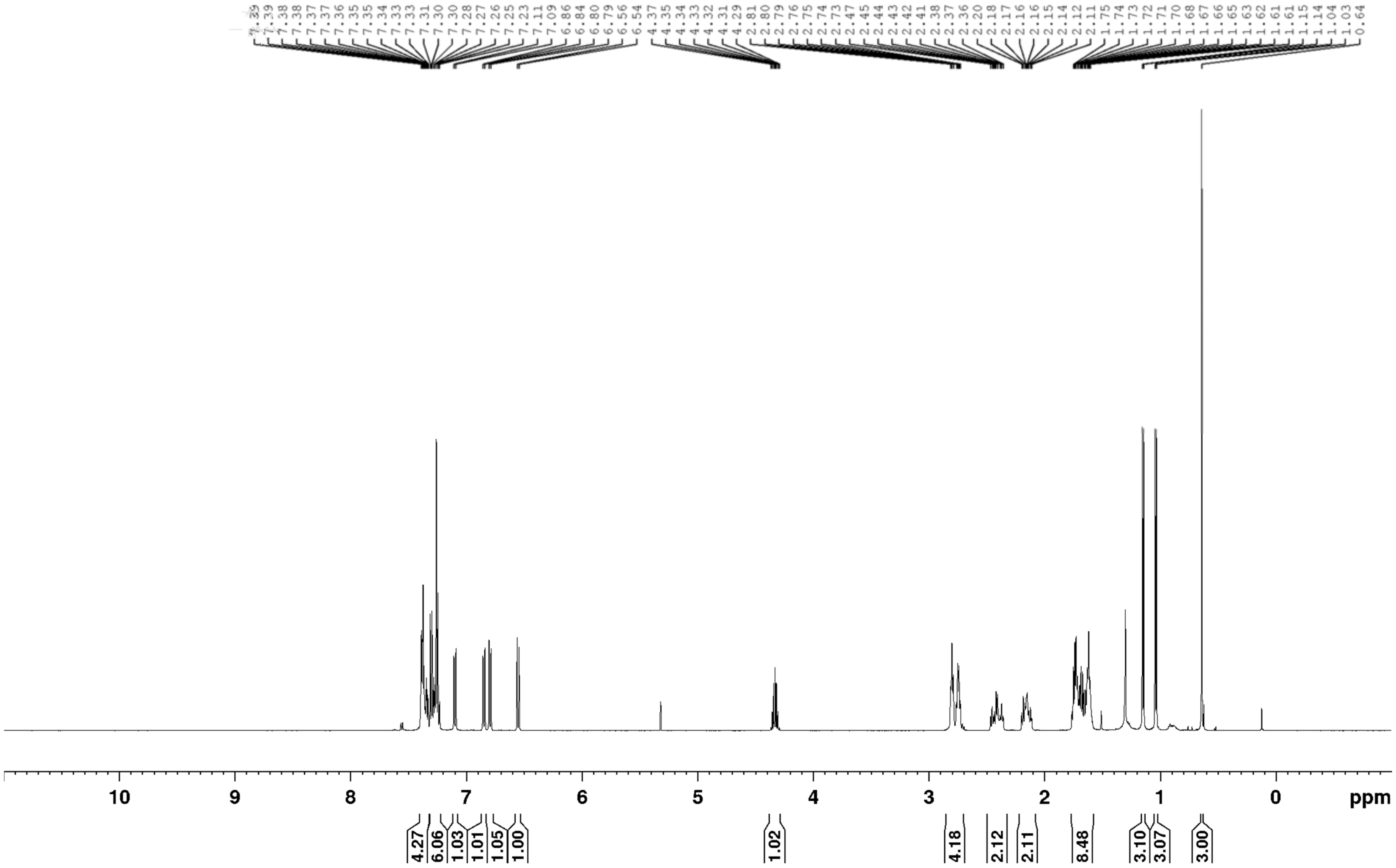

Figure S96. $^{13}C\{^{1}H\}$ NMR (126 MHz, CD_2Cl_2) of (*S*)-[(2'-Isopropoxy-5,5',6,6',7,7',8,8'-octahydro-[1,1'-binaphthalen]-2-yl)oxy](methyl)diphenylsilane [(*S*)-**3ka**].

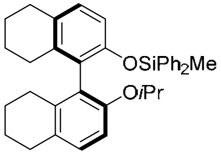

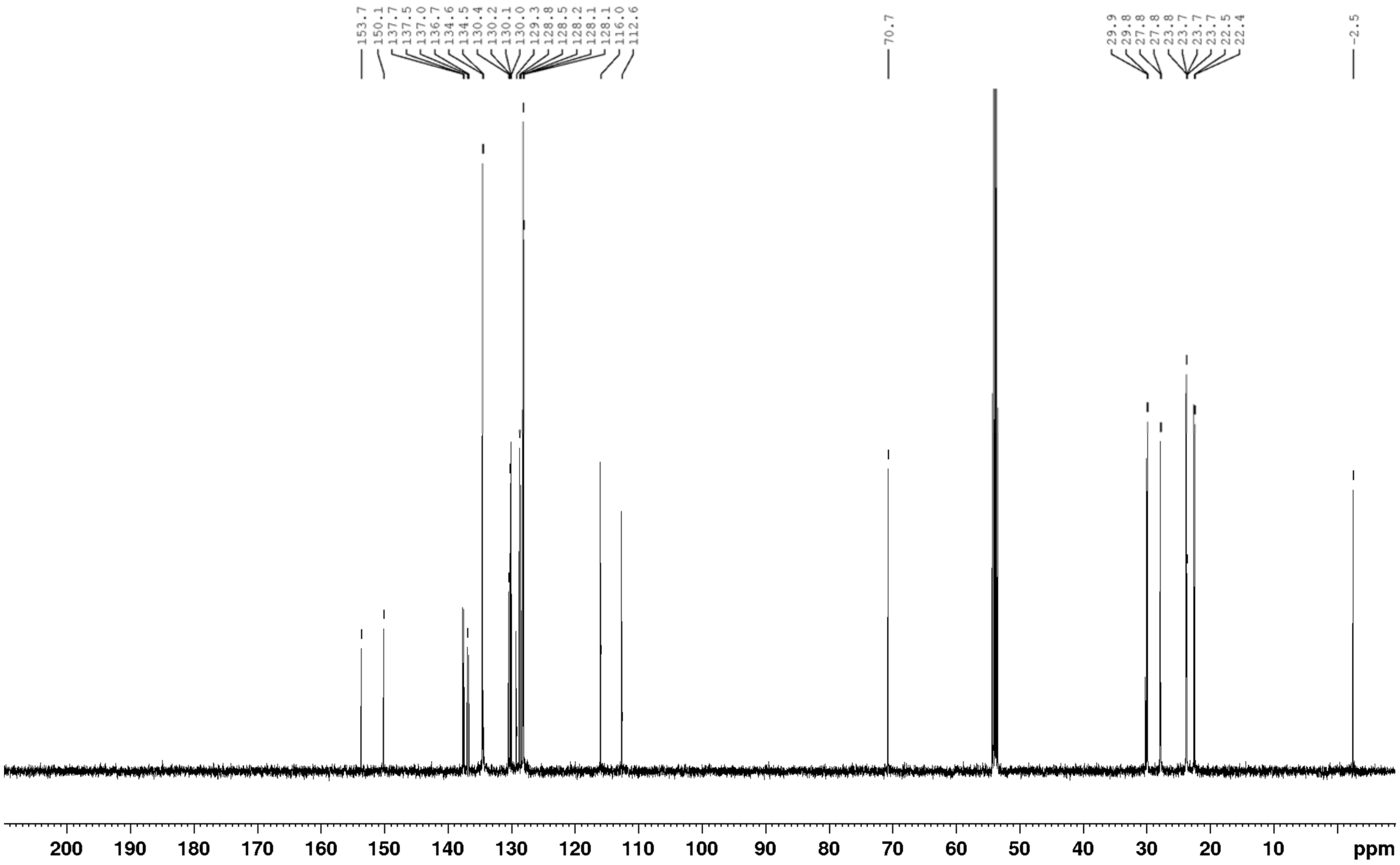

Figure S97. $^1H/^{29}Si$ HMQC NMR (500/99 MHz, CD_2Cl_2, optimized for *J* = 7 Hz) of (*S*)-[(2'-Isopropoxy-5,5',6,6',7,7',8,8'-octahydro-[1,1'-binaphthalen]-2-yl)oxy](methyl)diphenylsilane [(*S*)-**3ka**].

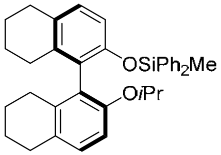

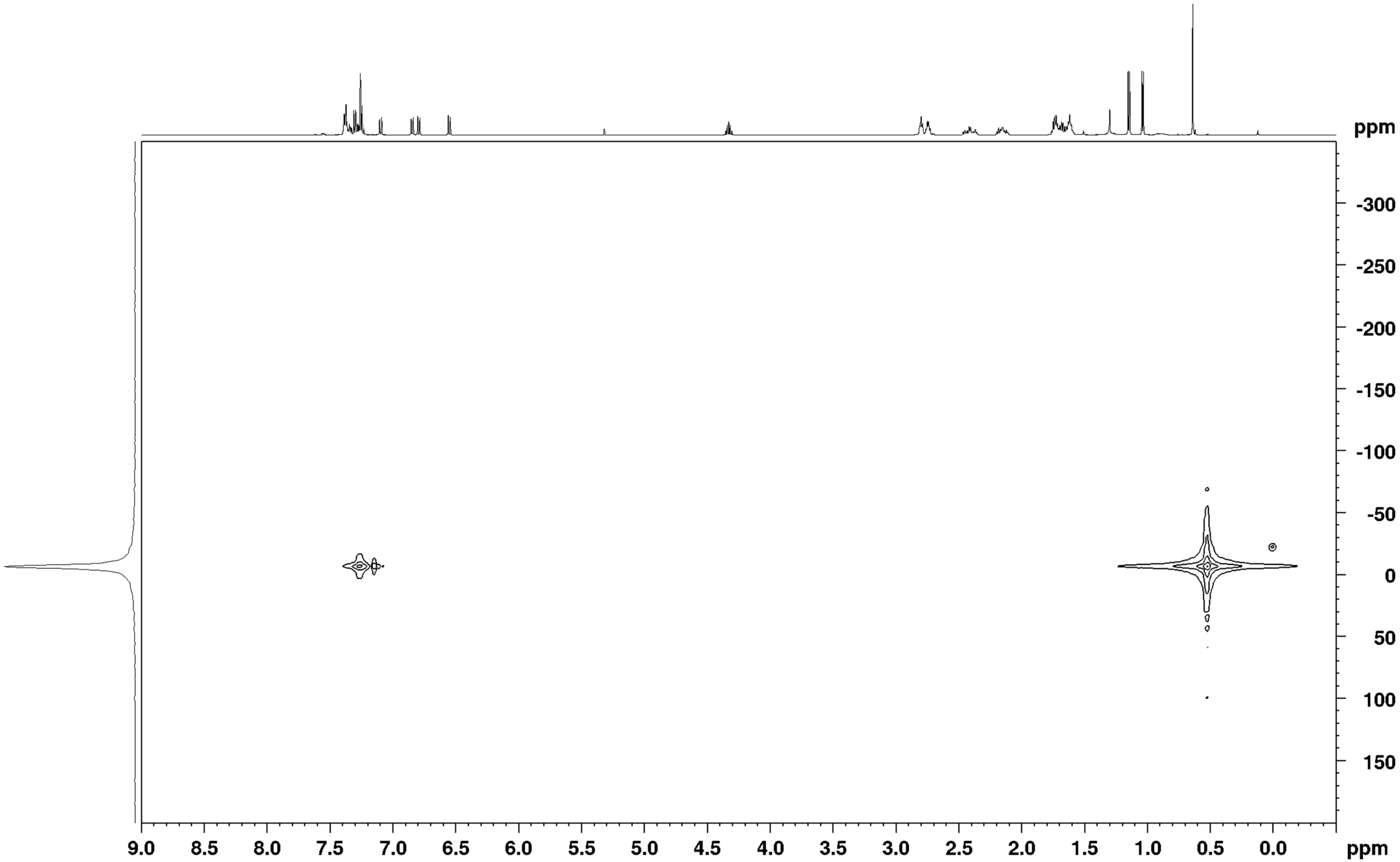

Figure S98. ^{1}H NMR (500 MHz, $CDCl_3$) of (*R*)-2'-Isopropoxy-5,5',6,6',7,7',8,8'-octahydro-[1,1'-binaphthalen]-2-ol [(*R*)-**1k**].

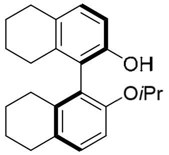

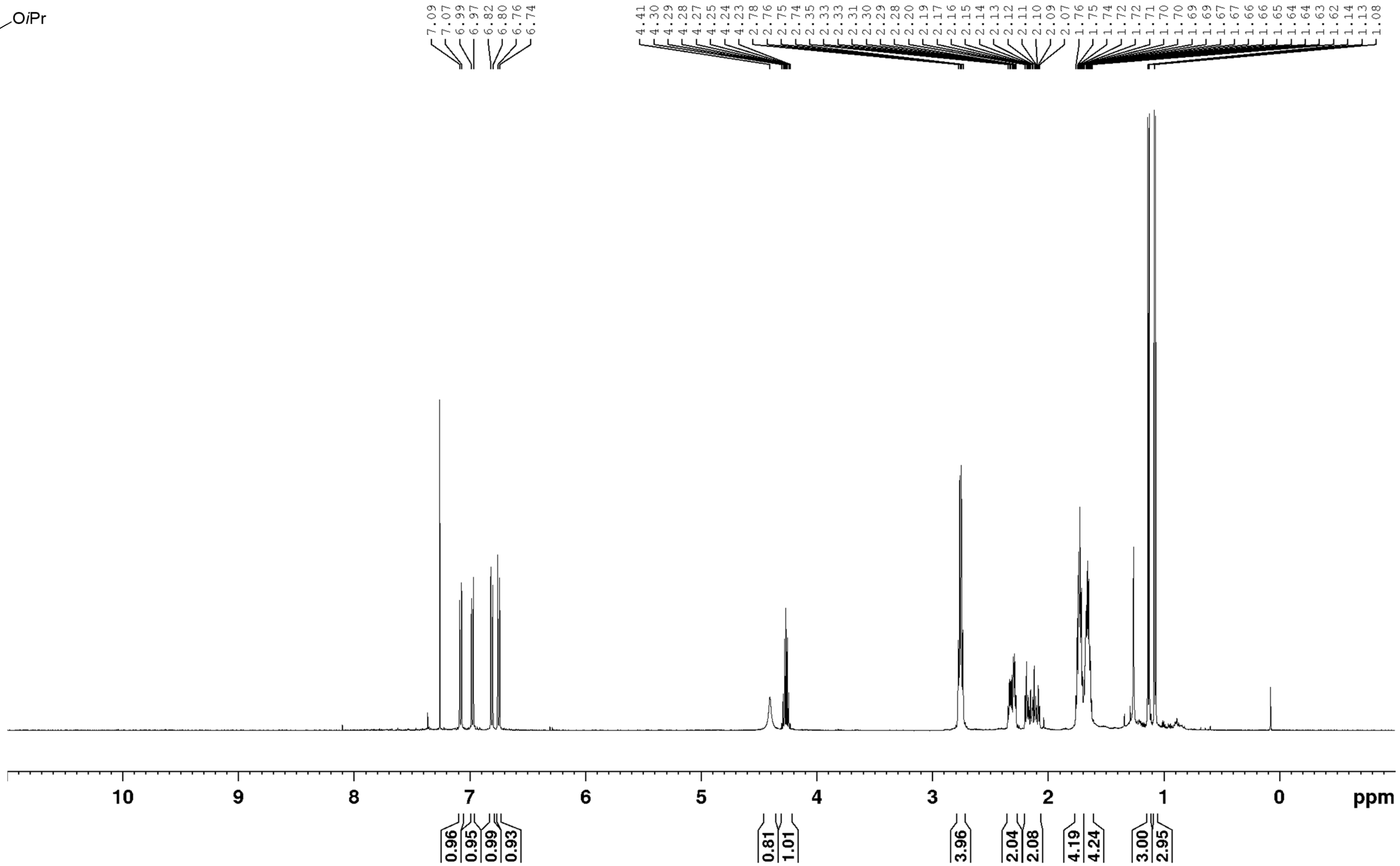

Figure S99. $^{13}C\{^{1}H\}$ NMR (126 MHz, $CDCl_3$) of (*R*)-2'-Isopropoxy-5,5',6,6',7,7',8,8'-octahydro-[1,1'-binaphthalen]-2-ol [(*R*)-**1k**].

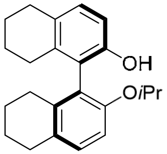

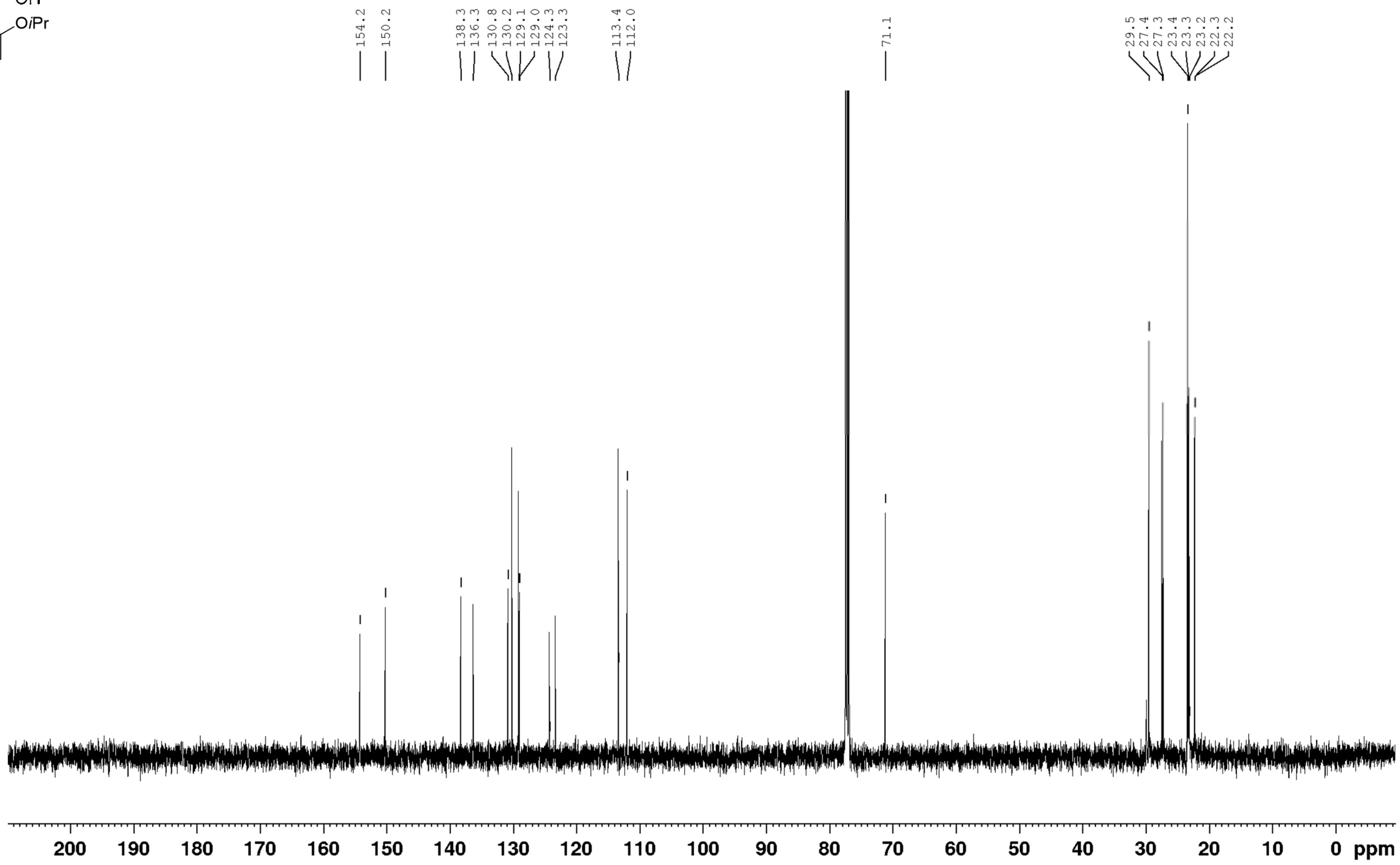

Figure S100. ^1H NMR (500 MHz, CD_2Cl_2) of (*S*)-[(2'-Isopropoxy-4,4'-dimethyl-[1,1'-binaphthalen]-2-yl)oxy](methyl)diphenylsilane [(*S*)-**3la**].

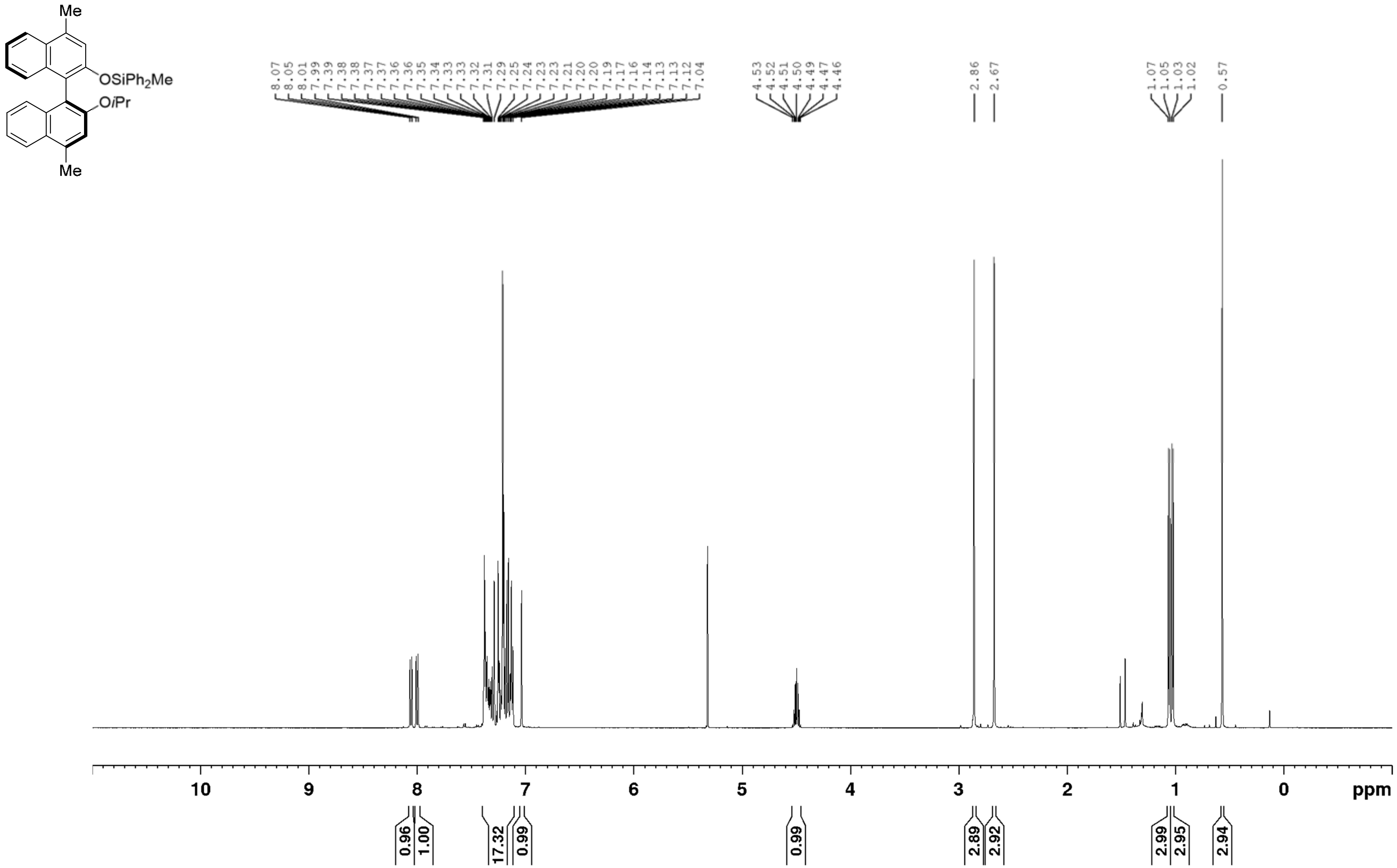

Figure S101. $^{13}C\{^{1}H\}$ NMR (126 MHz, CD_2Cl_2) of (*S*)-[(2'-Isopropoxy-4,4'-dimethyl-[1,1'-binaphthalen]-2-yl)oxy](methyl)diphenylsilane [(*S*)-**3la**].

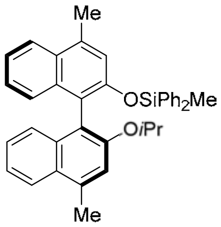

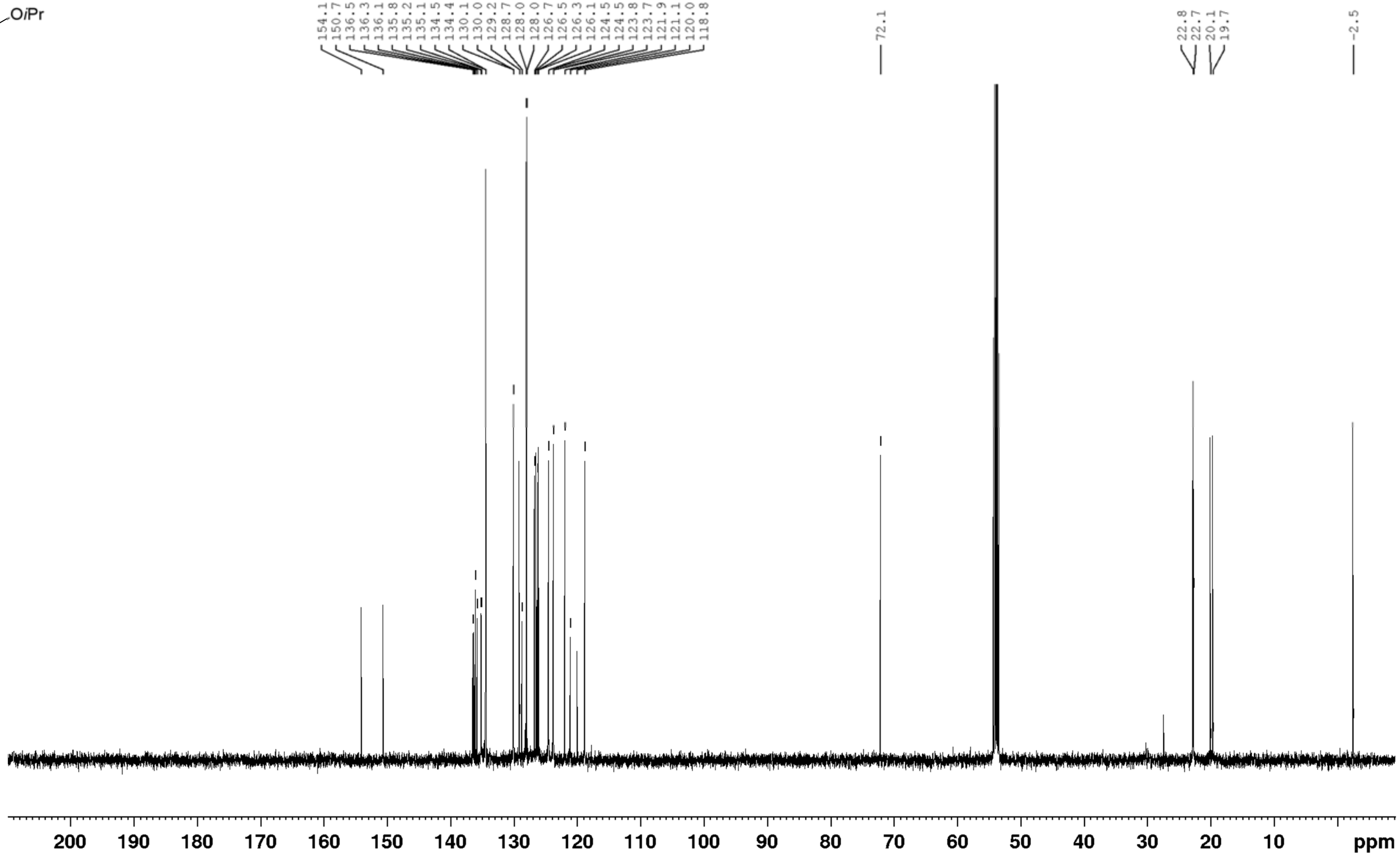

Figure S102. $^1H/^{29}Si$ HMQC NMR (500/99 MHz, CD_2Cl_2, optimized for J = 7 Hz) of (*S*)-[(2'-Isopropoxy-4,4'-dimethyl-[1,1'-binaphthalen]-2-yl)oxy](methyl)diphenylsilane [(*S*)-**3la**].

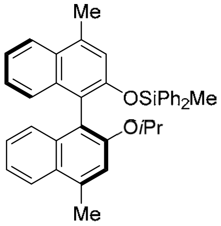

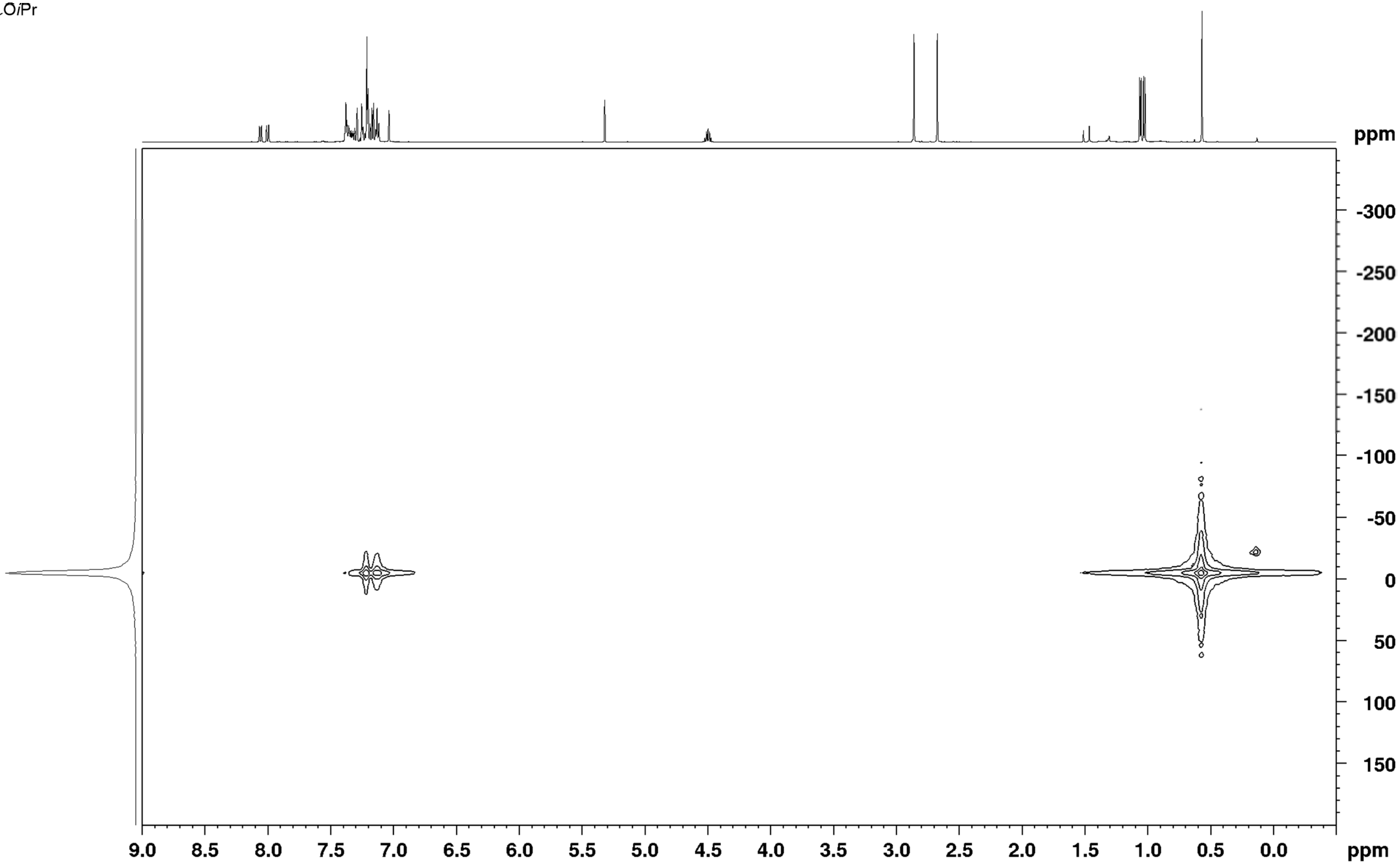

Figure S103. ^{1}H NMR (500 MHz, $CDCl_3$) of (*R*)-2'-Isopropoxy-4,4'-dimethyl-[1,1'-binaphthalen]-2-ol [(*R*)-**1l**].

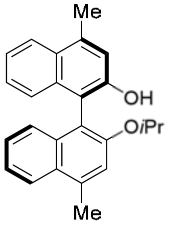

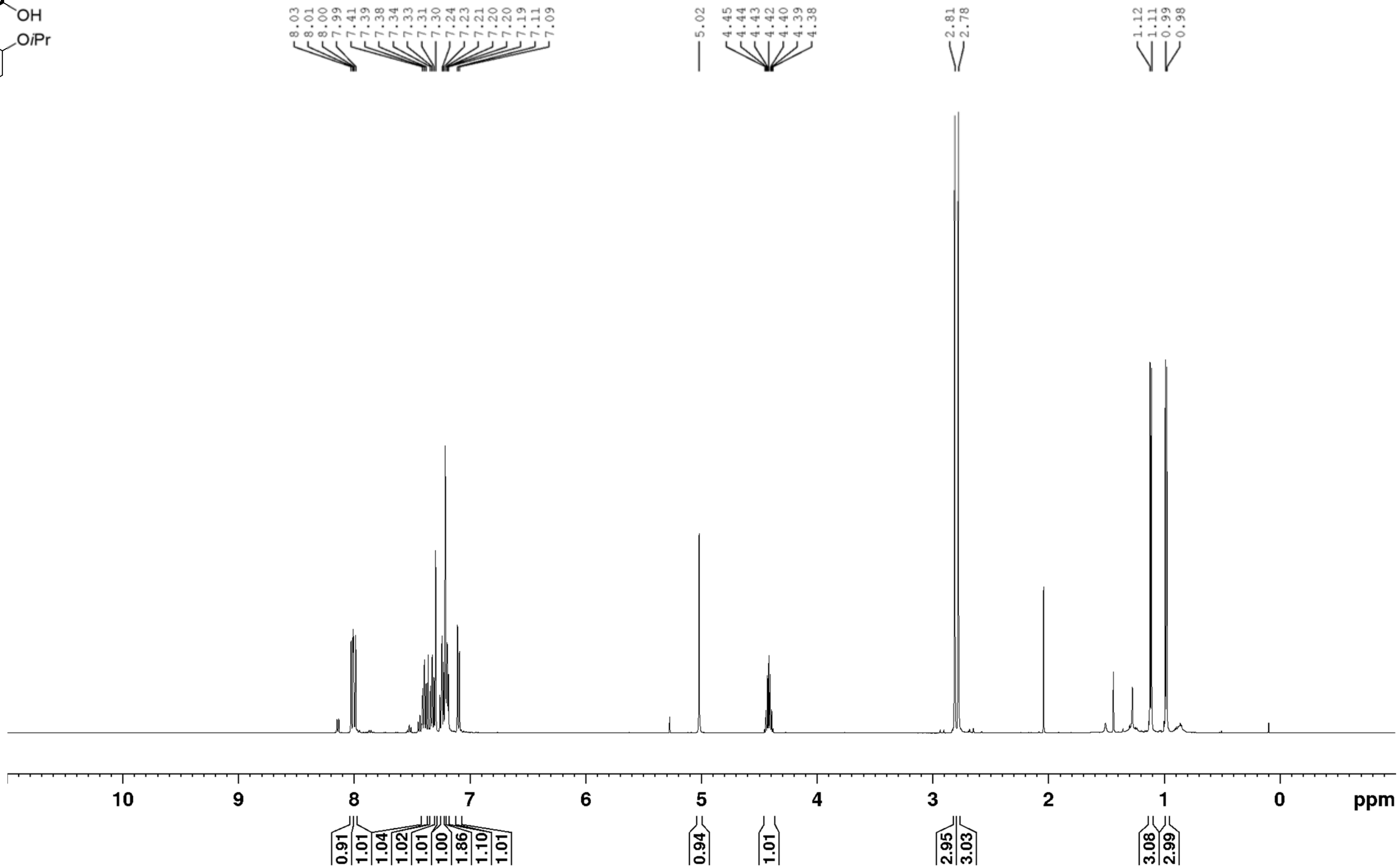

Figure S104. $^{13}C\{^1H\}$ NMR (126 MHz, $CDCl_3$) of (*R*)-2'-Isopropoxy-4,4'-dimethyl-[1,1'-binaphthalen]-2-ol [(*R*)-**1l**].

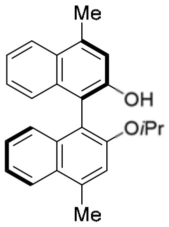

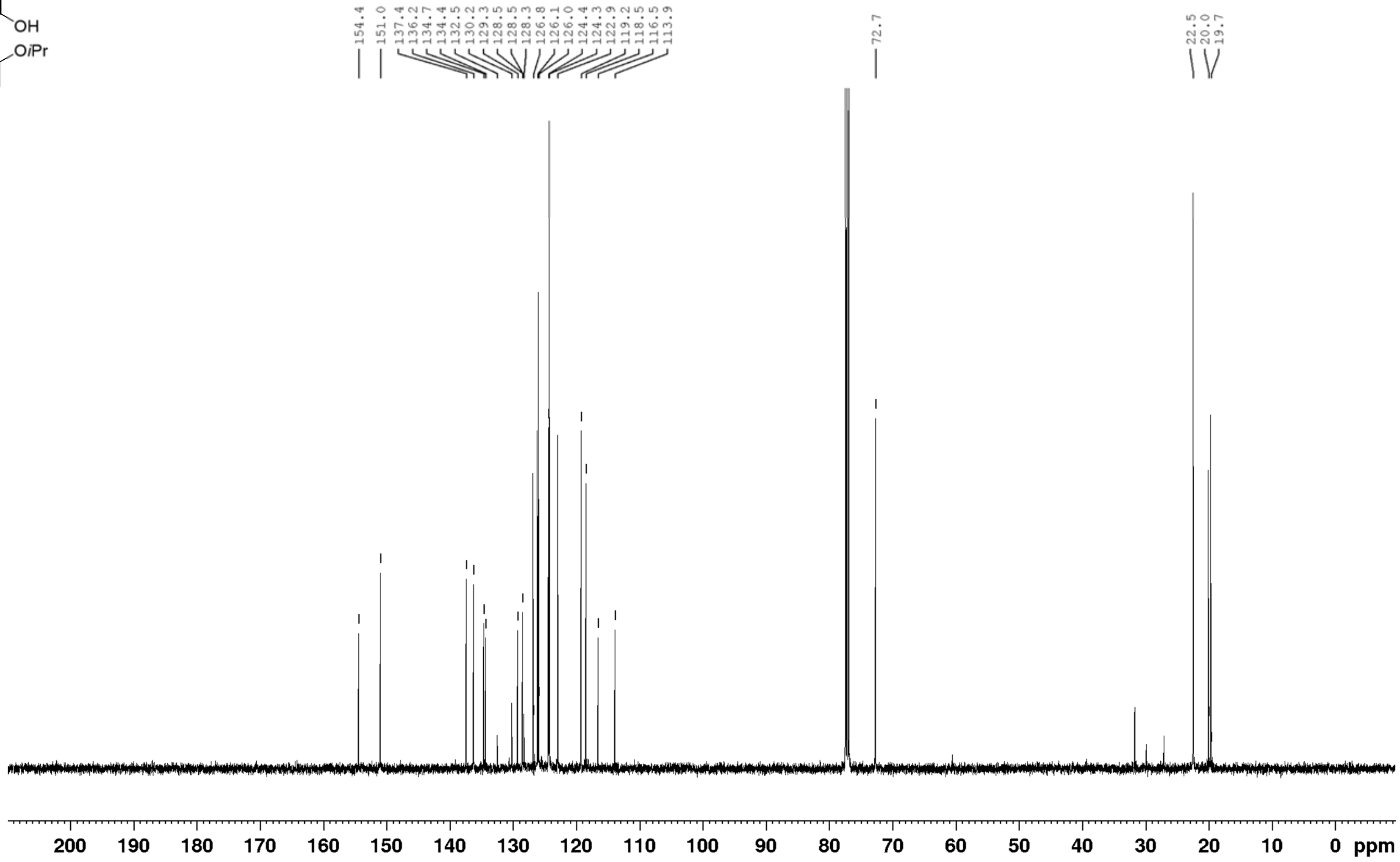

Figure S105. ^{1}H NMR (500 MHz, CD_2Cl_2) of (*S*)-[(2'-Isopropoxy-7,7'-dimethyl-[1,1'-binaphthalen]-2-yl)oxy](methyl)diphenylsilane [(*S*)-**3oa**].

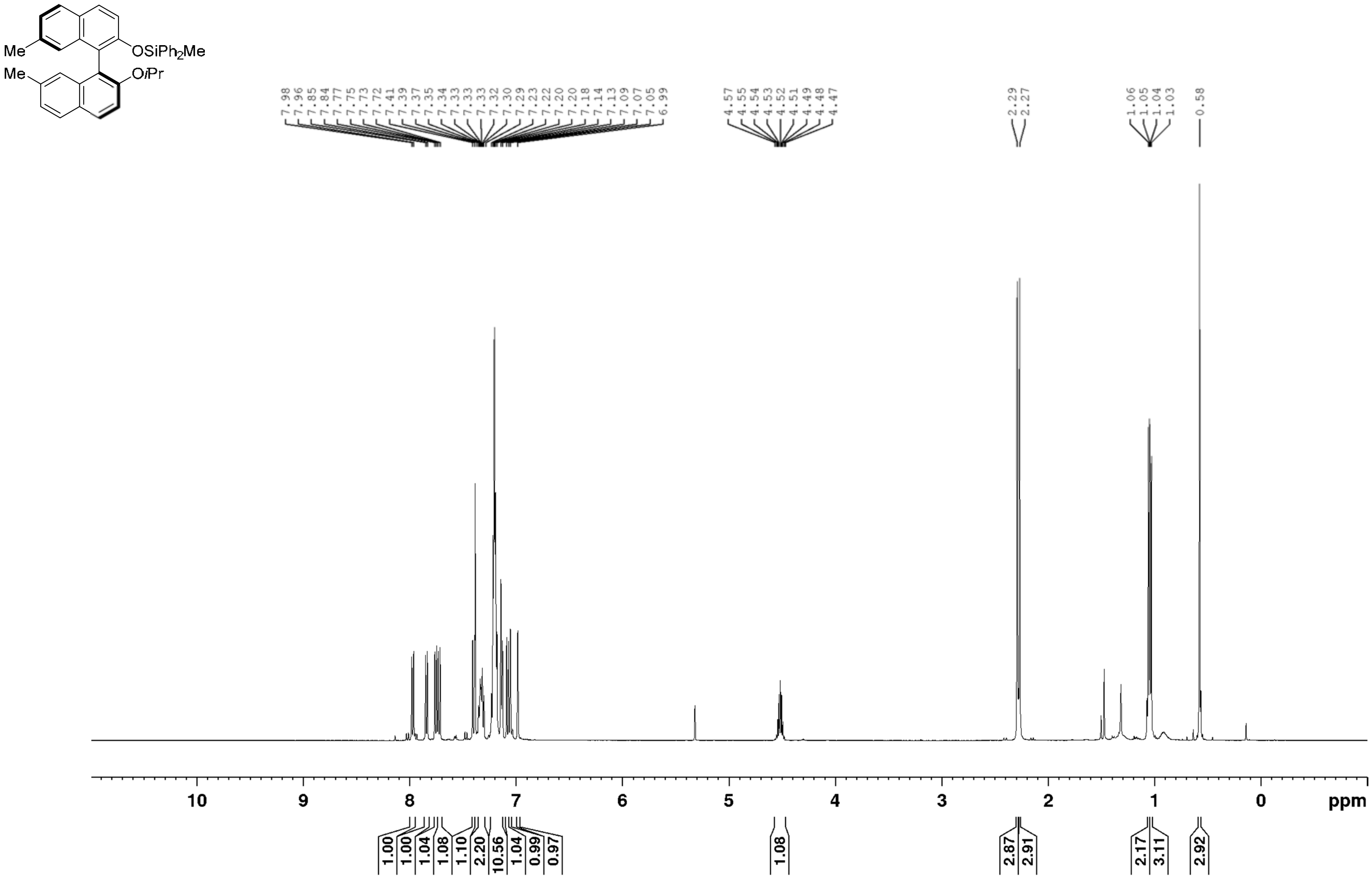

Figure S106. $^{13}C\{^{1}H\}$ NMR (126 MHz, CD_2Cl_2) of (*S*)-[(2'-Isopropoxy-7,7'-dimethyl-[1,1'-binaphthalen]-2-yl)oxy](methyl)diphenylsilane [(*S*)-**3oa**].

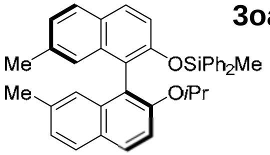

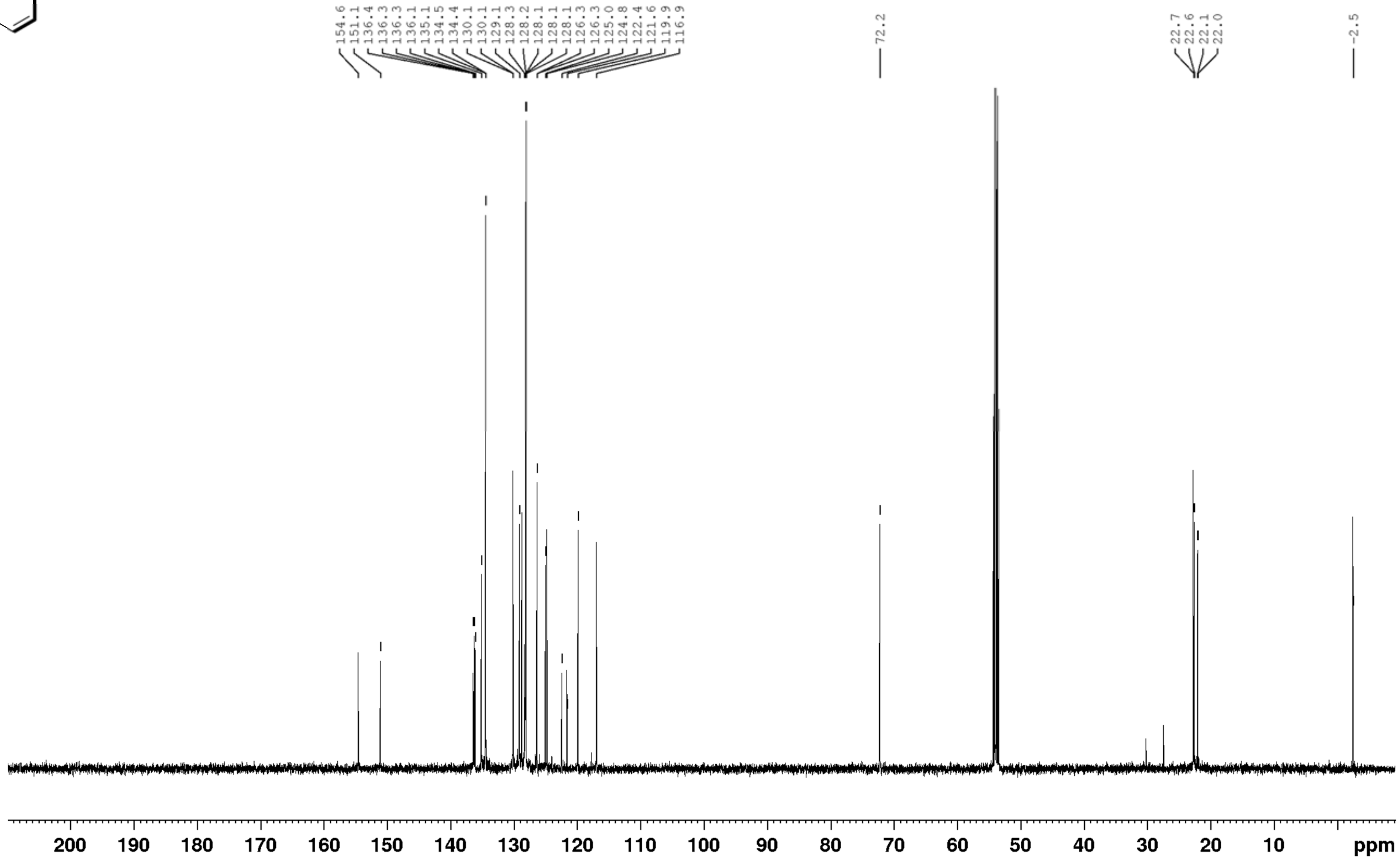

Figure S107. $^{1}H/^{29}Si$ HMQC NMR (500/99 MHz, CD_2Cl_2, optimized for J = 7 Hz) of (*S*)-[(2'-Isopropoxy-7,7'-dimethyl-[1,1'-binaphthalen]-2-yl)oxy](methyl)diphenylsilane [(*S*)-**3oa**].

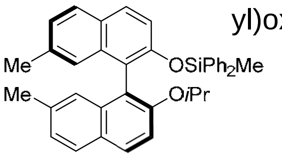

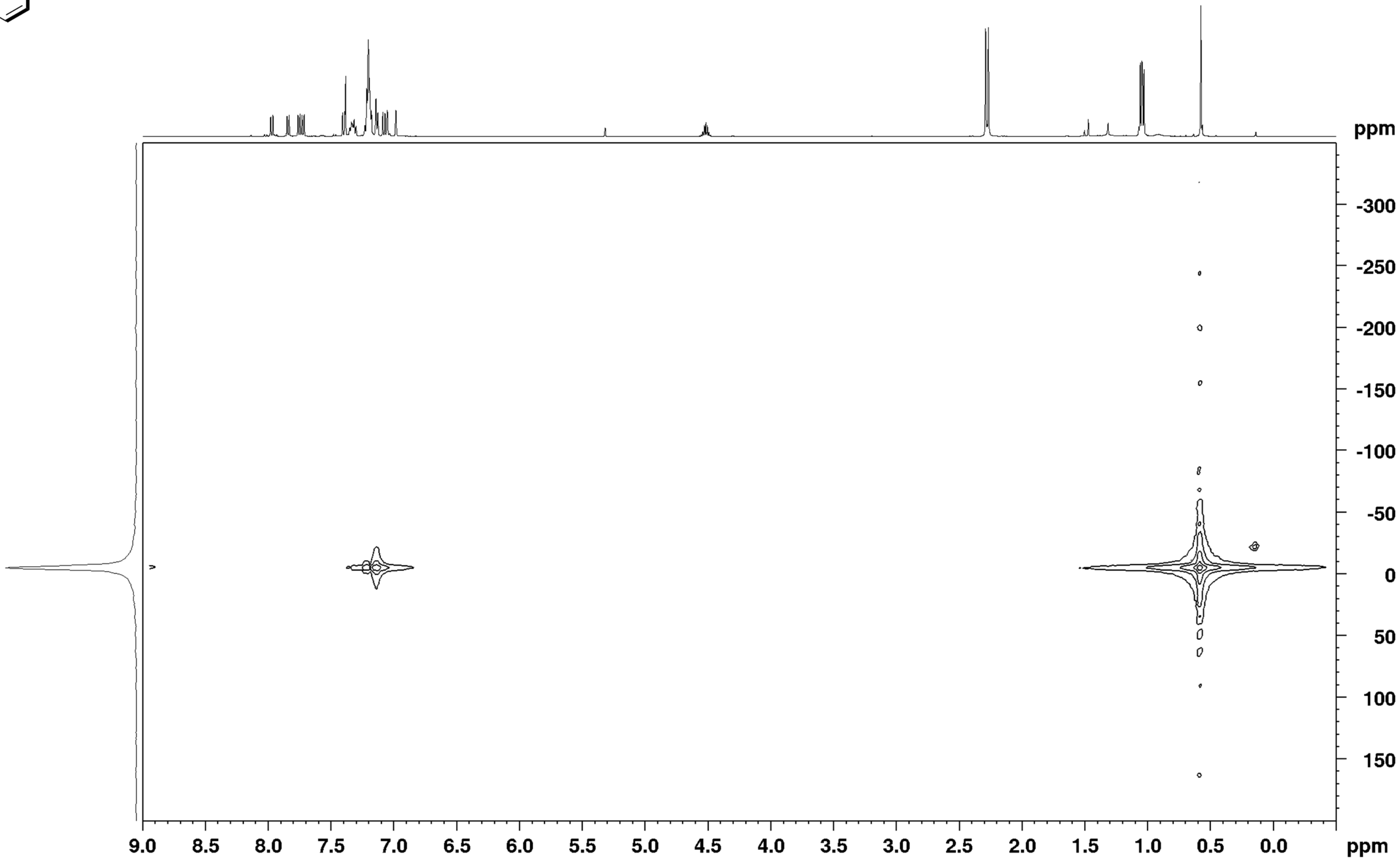

Figure S108. ^{1}H NMR (500 MHz, $CDCl_3$) of (*R*)-2'-Isopropoxy-7,7'-dimethyl-[1,1'-binaphthalen]-2-ol [(*R*)-**1o**].

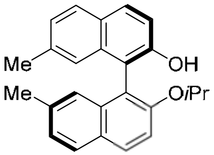

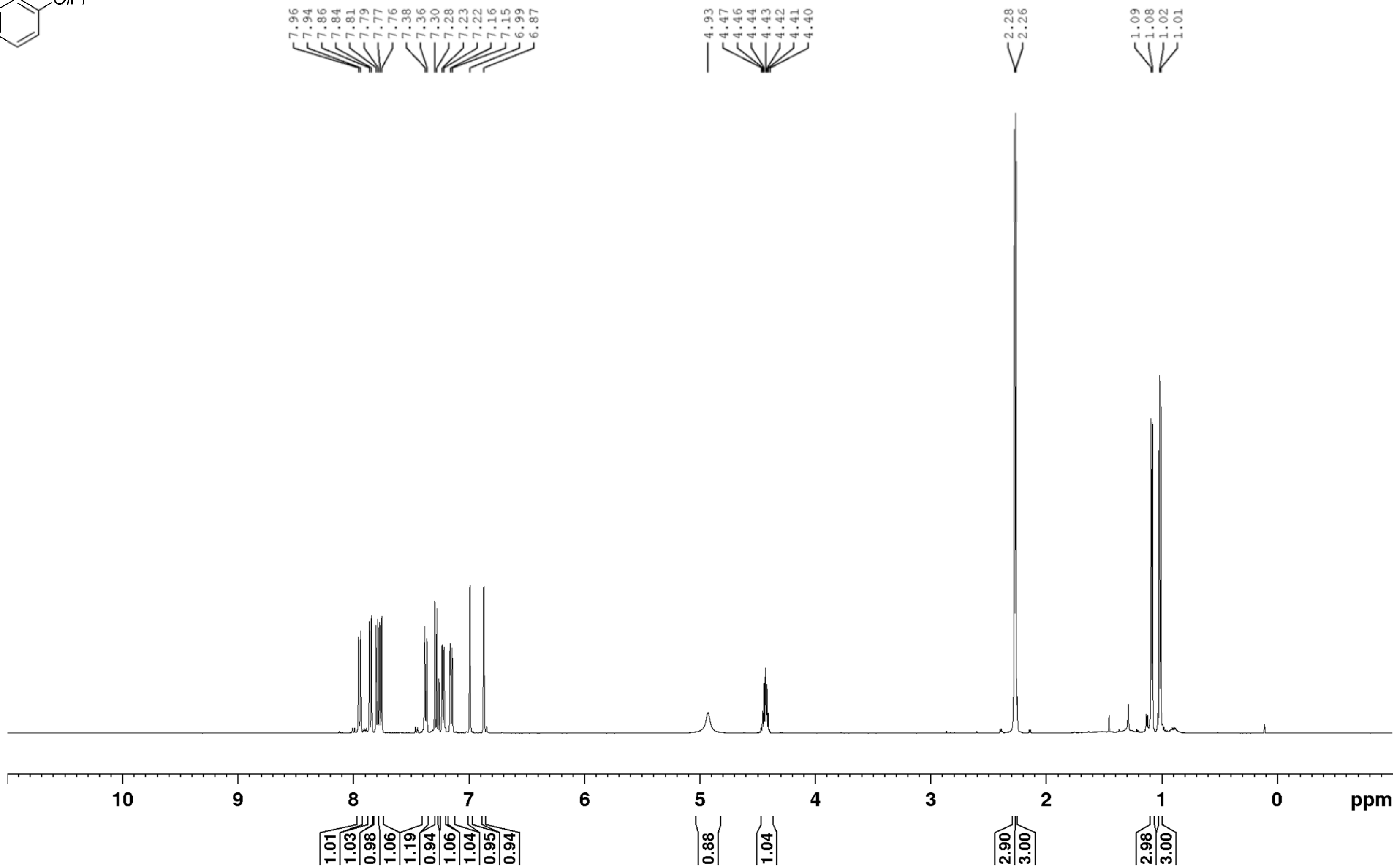

Figure S109. $^{13}C\{^{1}H\}$ NMR (126 MHz, $CDCl_3$) of (*R*)-2'-Isopropoxy-7,7'-dimethyl-[1,1'-binaphthalen]-2-ol [(*R*)-**1o**].

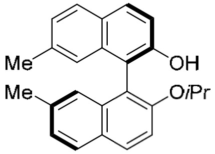

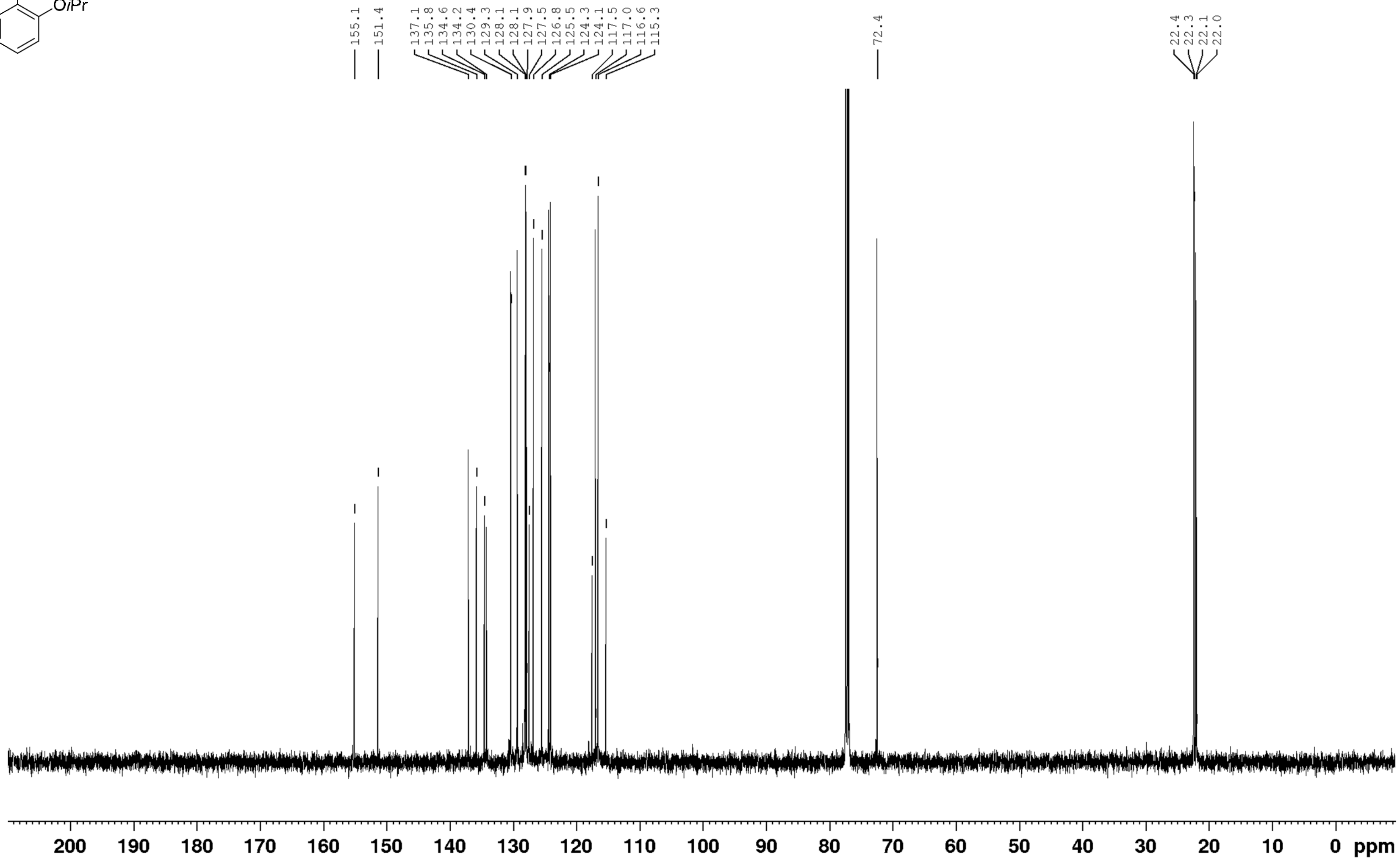

Figure S110. ^{1}H NMR (500 MHz, CD_2Cl_2) of (*S*)-[(2'-Isopropoxy-6,6'-dimethyl-[1,1'-binaphthalen]-2-yl)oxy](methyl)diphenylsilane [(*S*)-**3qa**].

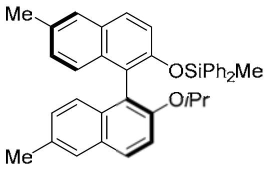

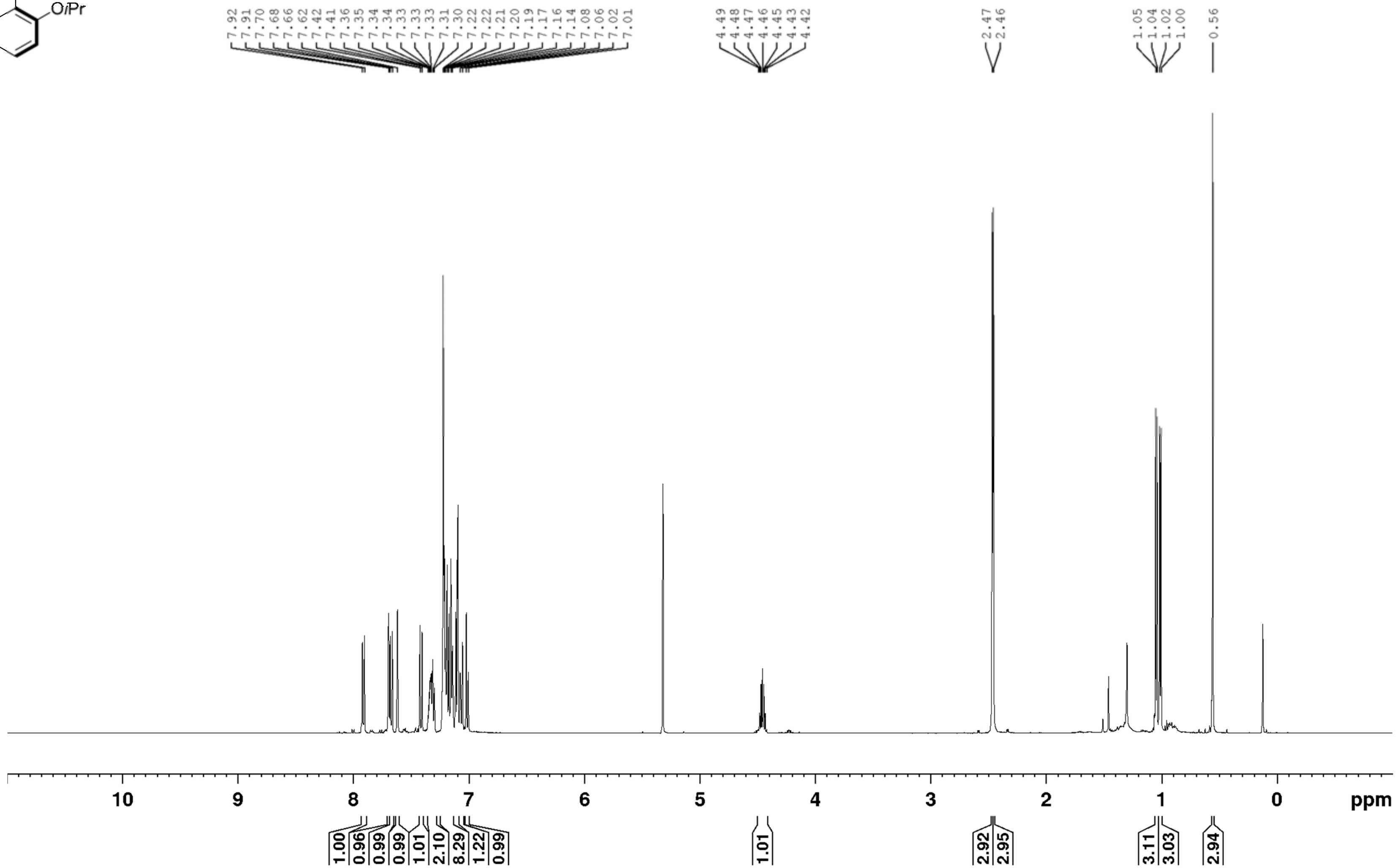

Figure S111. $^{13}C\{^{1}H\}$ NMR (126 MHz, CD_2Cl_2) of (*S*)-[(2'-Isopropoxy-6,6'-dimethyl-[1,1'-binaphthalen]-2-yl)oxy](methyl)diphenylsilane [(*S*)-**3qa**].

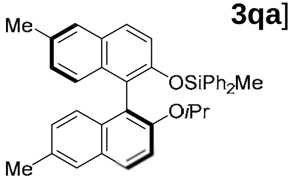

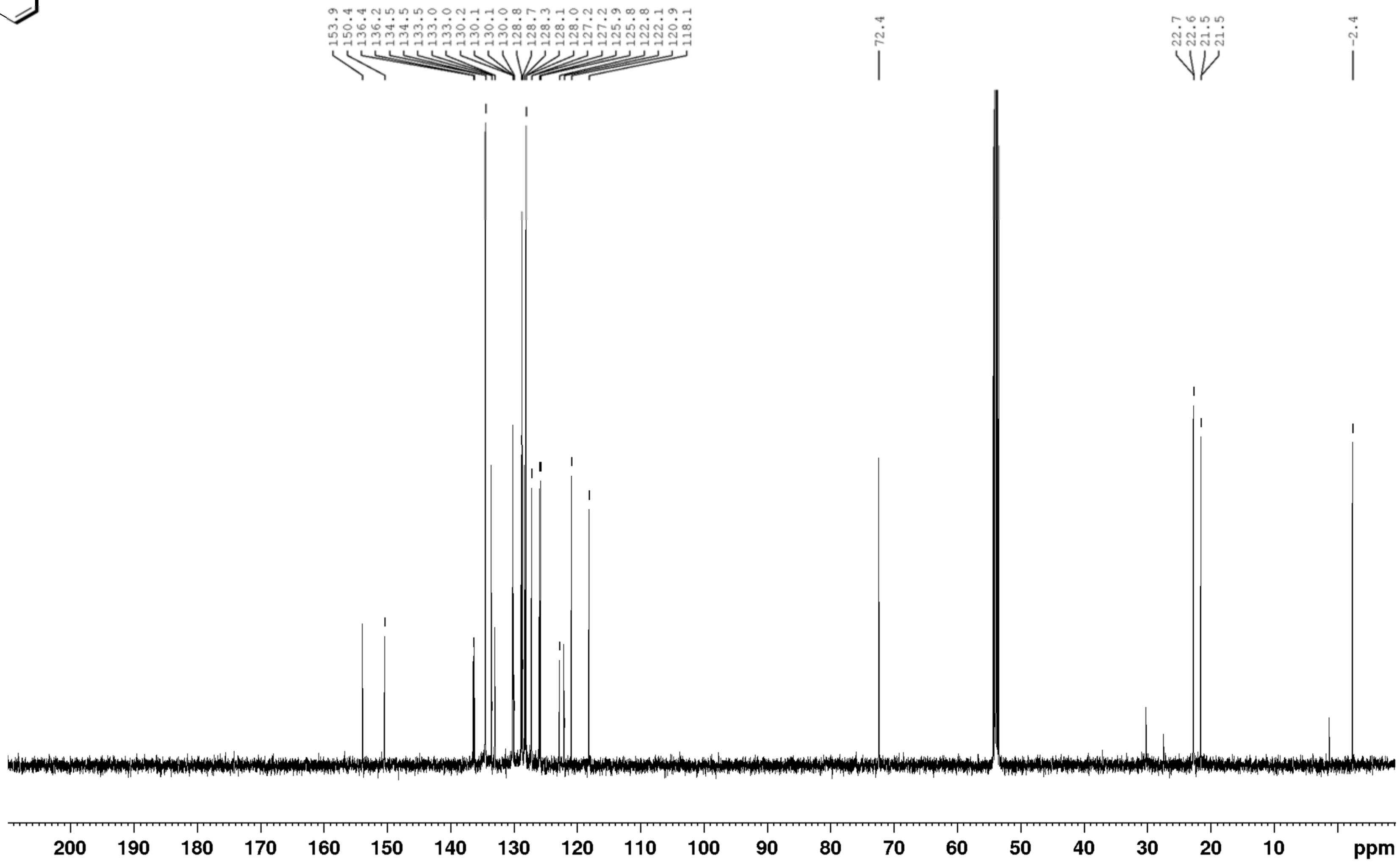

Figure S112. $^1H/^{29}Si$ HMQC NMR (500/99 MHz, CD_2Cl_2, optimized for J = 7 Hz) of (*S*)-[(2'-Isopropoxy-6,6'-dimethyl-[1,1'-binaphthalen]-2-yl)oxy](methyl)diphenylsilane [(*S*)-**3qa**].

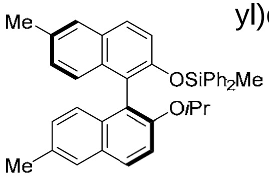

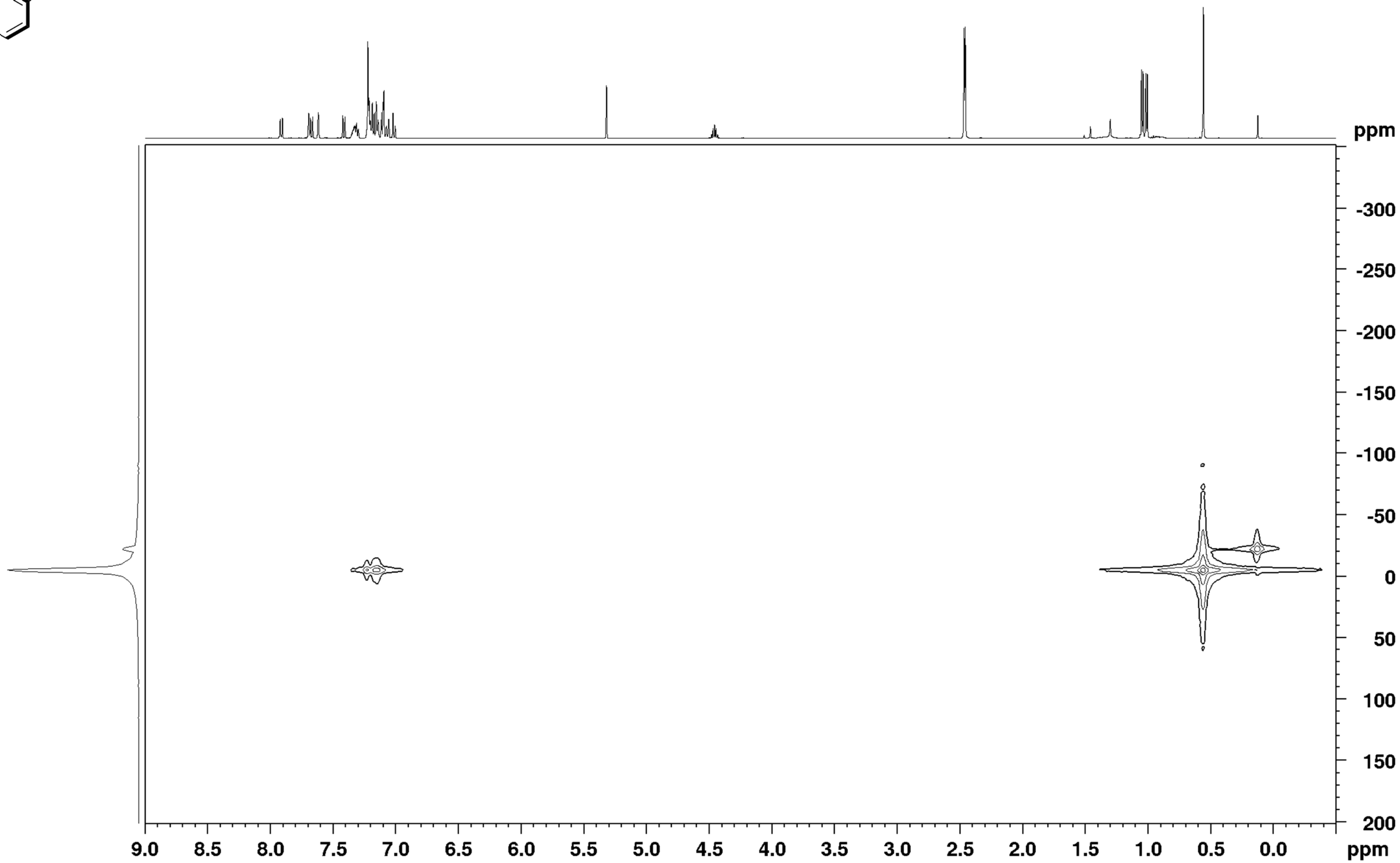

Figure S113. ^{1}H NMR (500 MHz, $CDCl_3$) of (*R*)-2'-Isopropoxy-6,6'-dimethyl-[1,1'-binaphthalen]-2-ol [(*R*)-**1q**].

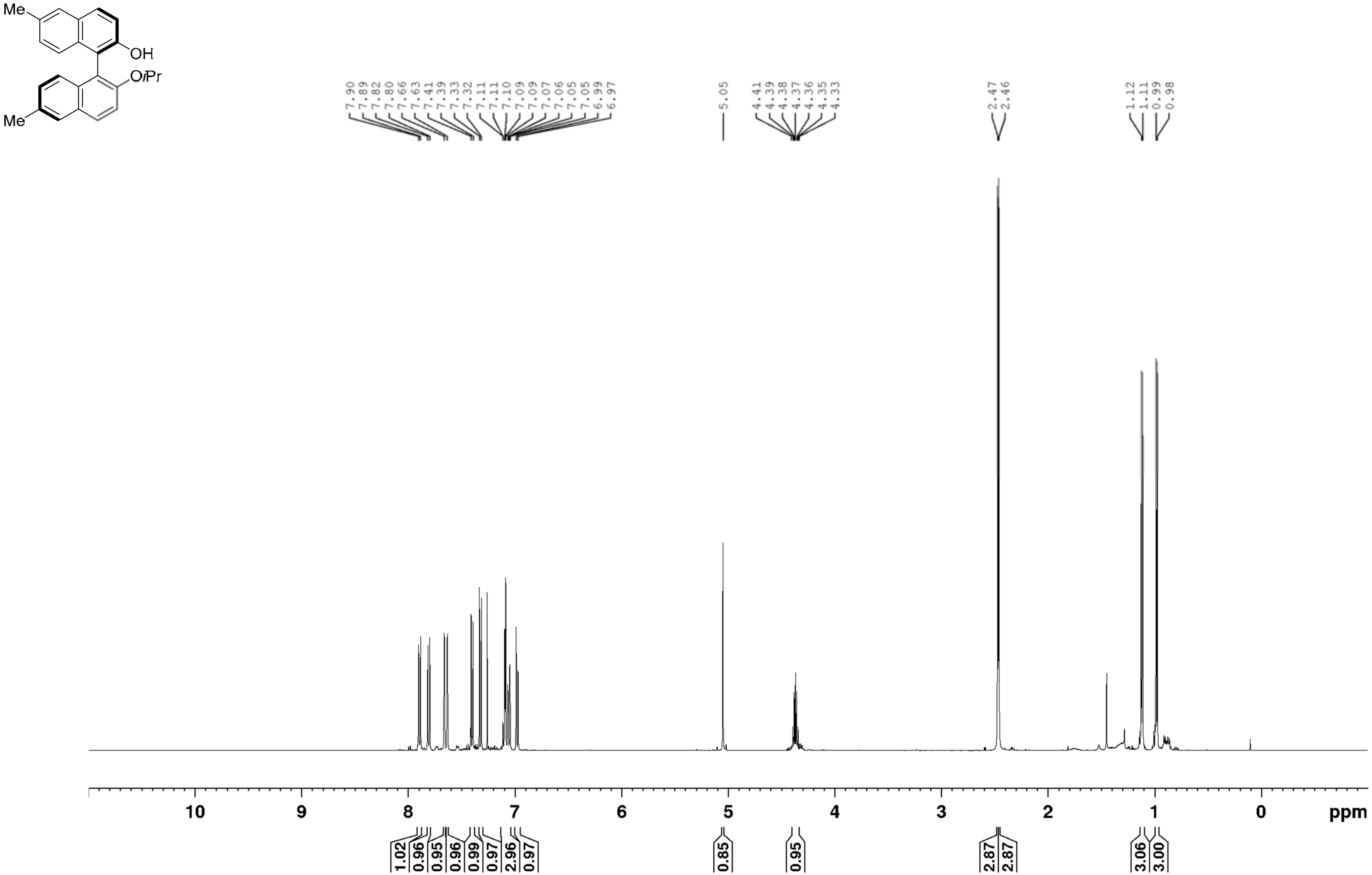

Figure S114. $^{13}C\{^{1}H\}$ NMR (126 MHz, $CDCl_3$) of (*R*)-2'-Isopropoxy-6,6'-dimethyl-[1,1'-binaphthalen]-2-ol [(*R*)-**1q**].

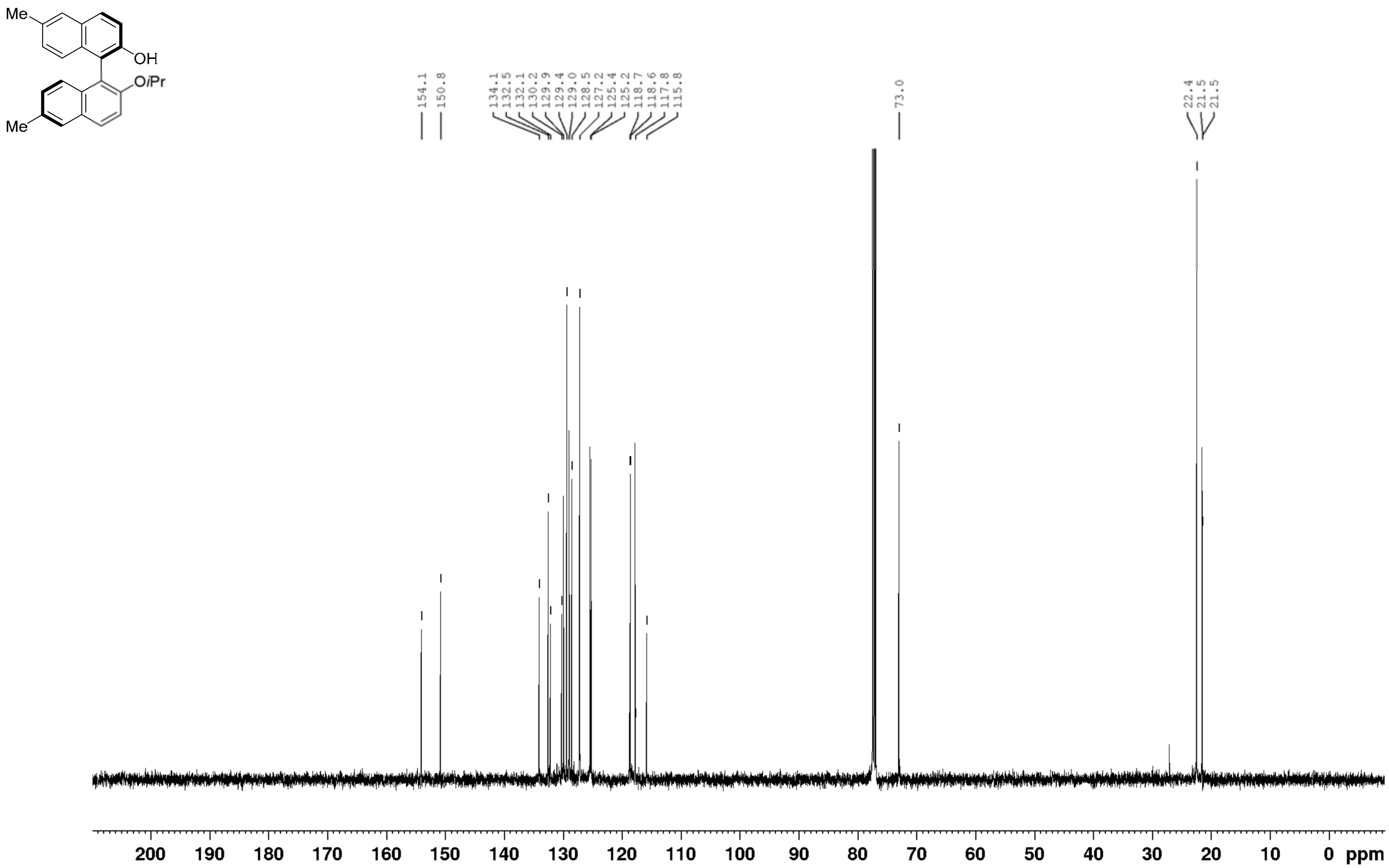

Figure S115. ^{1}H NMR (500 MHz, CD_2Cl_2) of (*S*)-[(2'-Isopropoxy-6,6'-diisopropyl-[1,1'-binaphthalen]-2-yl)oxy](methyl)diphenylsilane [(*S*)-**3ra**].

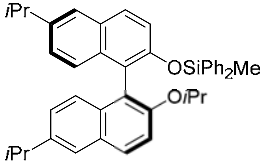

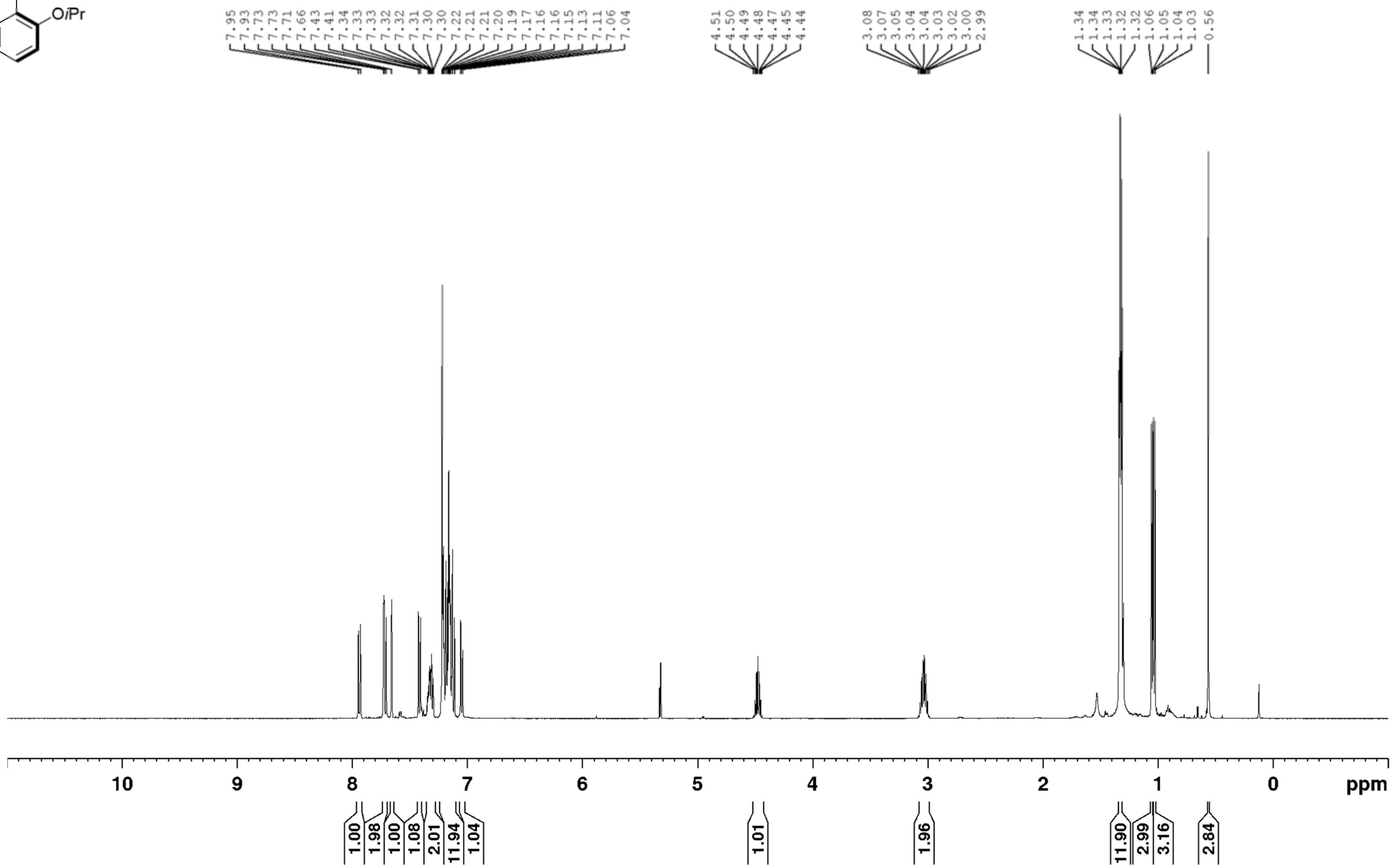

Figure S116. $^{13}C\{^{1}H\}$ NMR (126 MHz, CD_2Cl_2) of (*S*)-[(2'-Isopropoxy-6,6'-diisopropyl-[1,1'-binaphthalen]-2-yl)oxy](methyl)diphenylsilane [(*S*)-**3ra**].

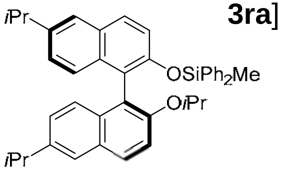

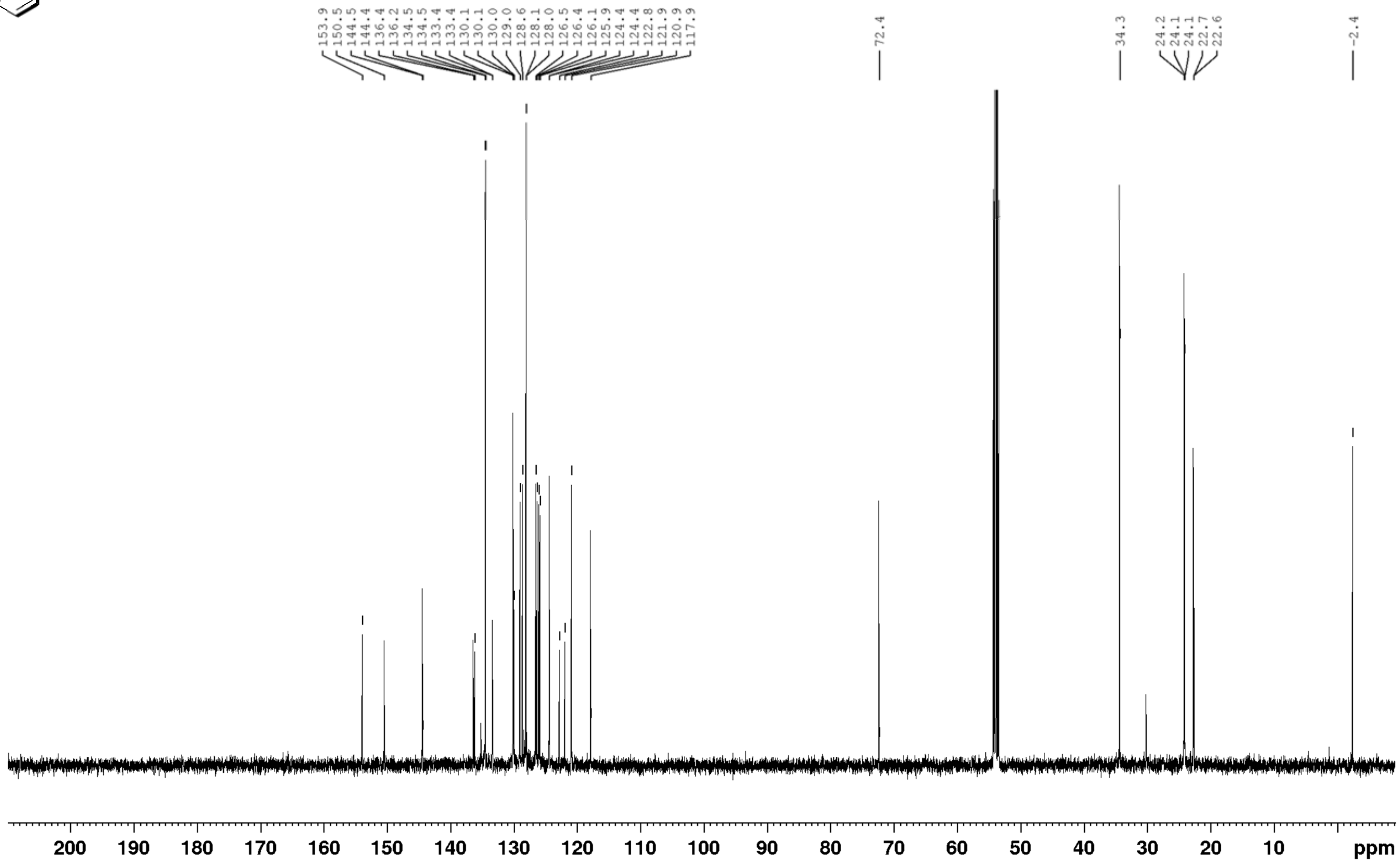

Figure S117. $^{1}H/^{29}Si$ HMQC NMR (500/99 MHz, CD_2Cl_2, optimized for *J* = 7 Hz) of (*S*)-[(2'-Isopropoxy-6,6'-diisopropyl-[1,1'-binaphthalen]-2-yl)oxy](methyl)diphenylsilane [(*S*)-**3ra**].

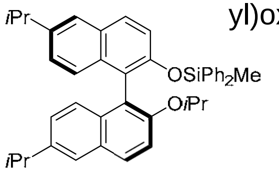

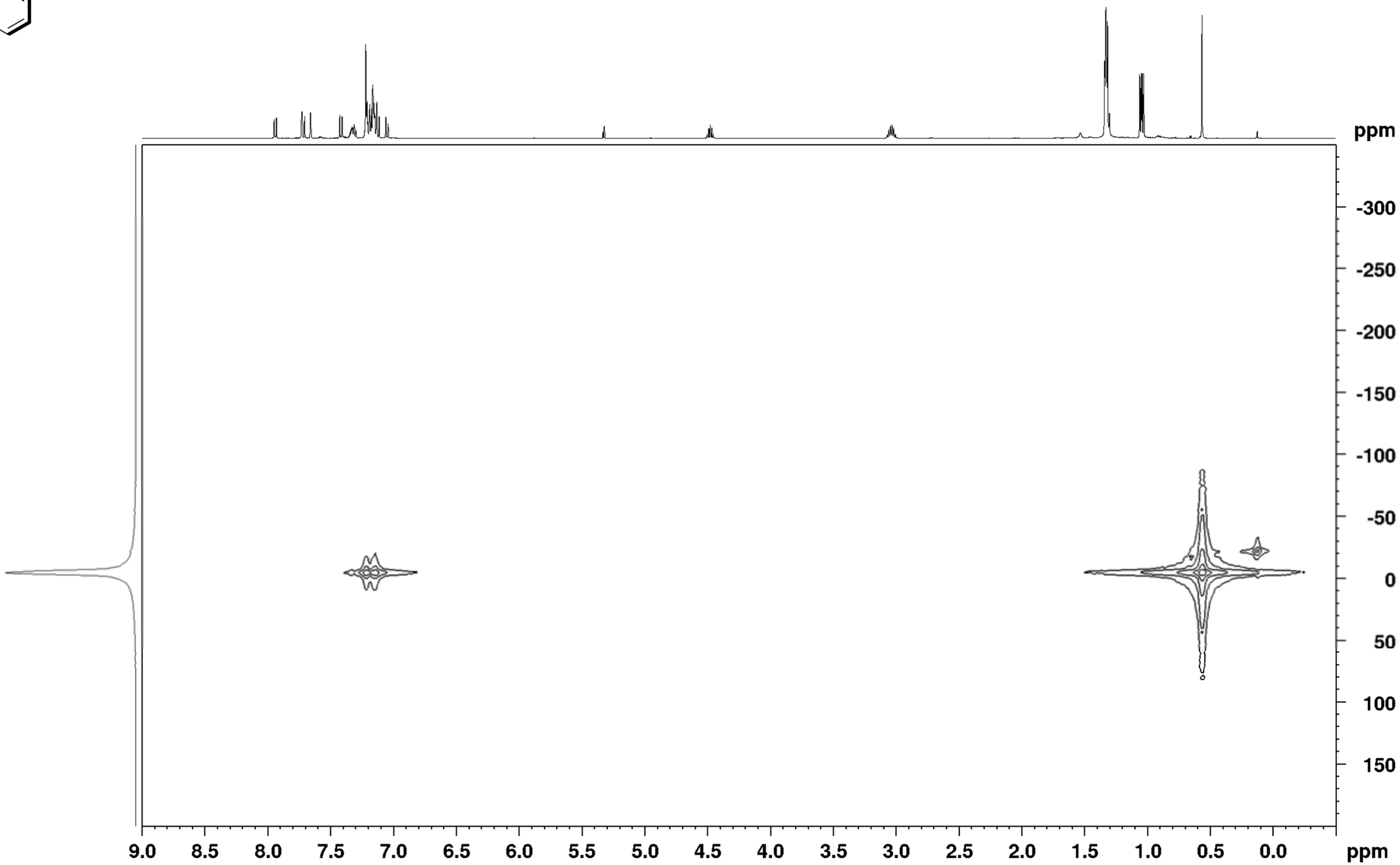

Figure S118. ^{1}H NMR (500 MHz, $CDCl_3$) of (*R*)-2'-Isopropoxy-6,6'-diisopropyl-[1,1'-binaphthalen]-2-ol [(*R*)-**1r**].

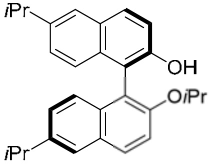

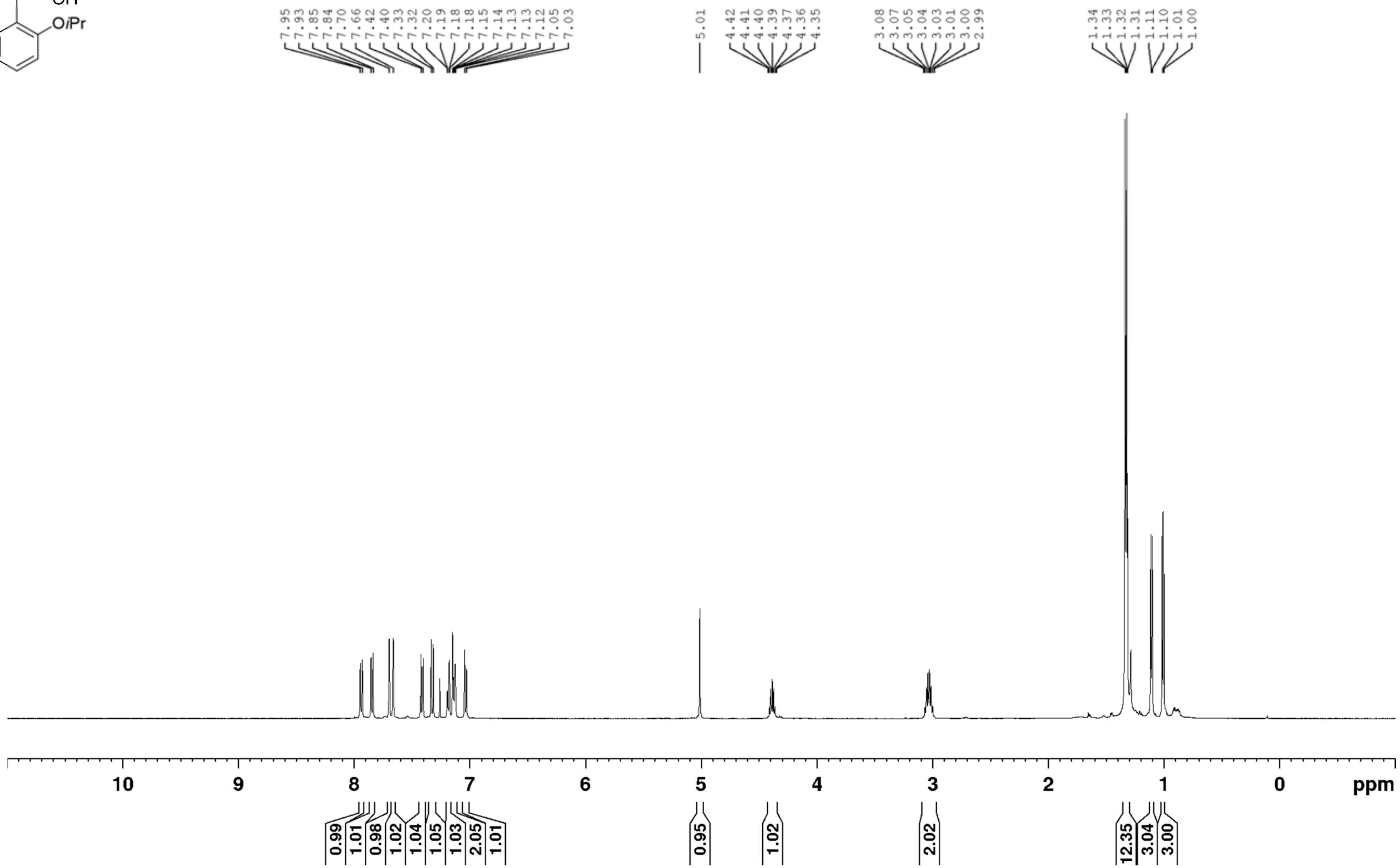

Figure S119. $^{13}C\{^{1}H\}$ NMR (126 MHz, $CDCl_3$) of (*R*)-2'-Isopropoxy-6,6'-diisopropyl-[1,1'-binaphthalen]-2-ol [(*R*)-**1r**].

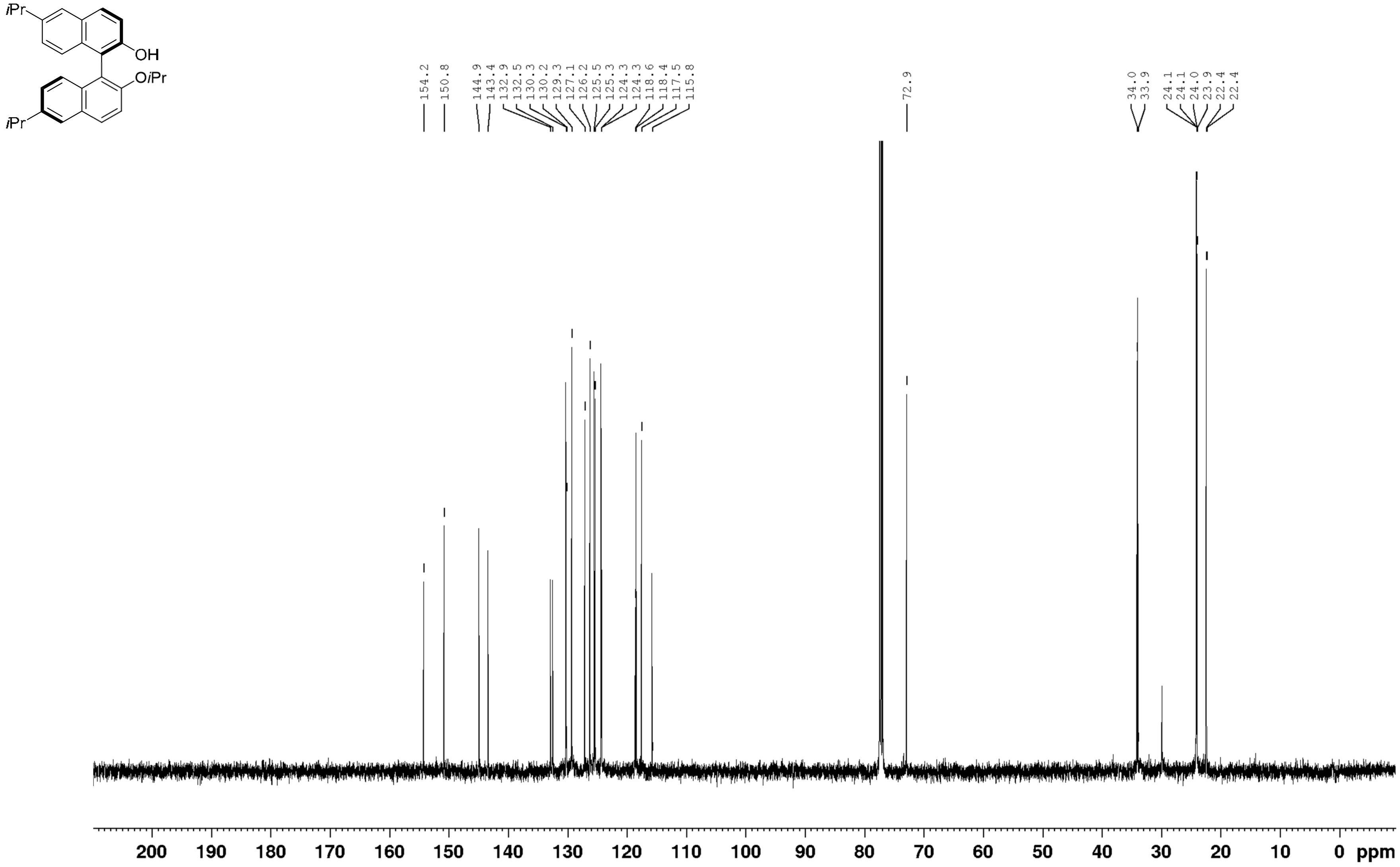

Figure S120. ^{1}H NMR (500 MHz, CD_2Cl_2) of (*S*)-[(2'-Isopropoxy-6,6'-diphenyl-[1,1'-binaphthalen]-2-yl)oxy](methyl)diphenylsilane [(*S*)-**3sa**].

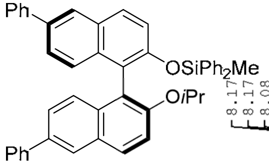

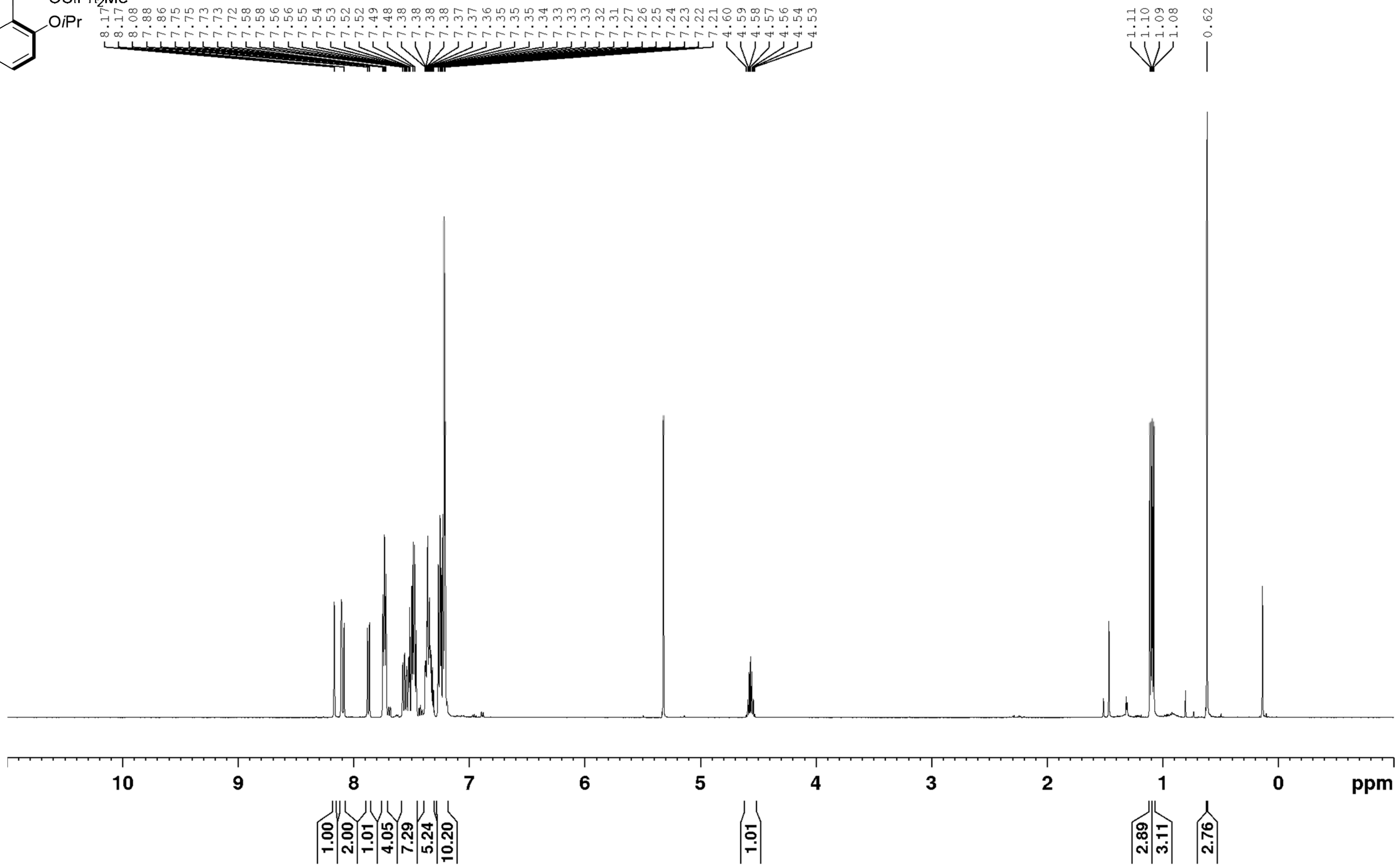

Figure S121. $^{13}C\{^{1}H\}$ NMR (126 MHz, CD_2Cl_2) of (*S*)-[(2'-Isopropoxy-6,6'-diphenyl-[1,1'-binaphthalen]-2-yl)oxy](methyl)diphenylsilane [(*S*)-**3sa**].

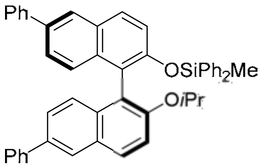

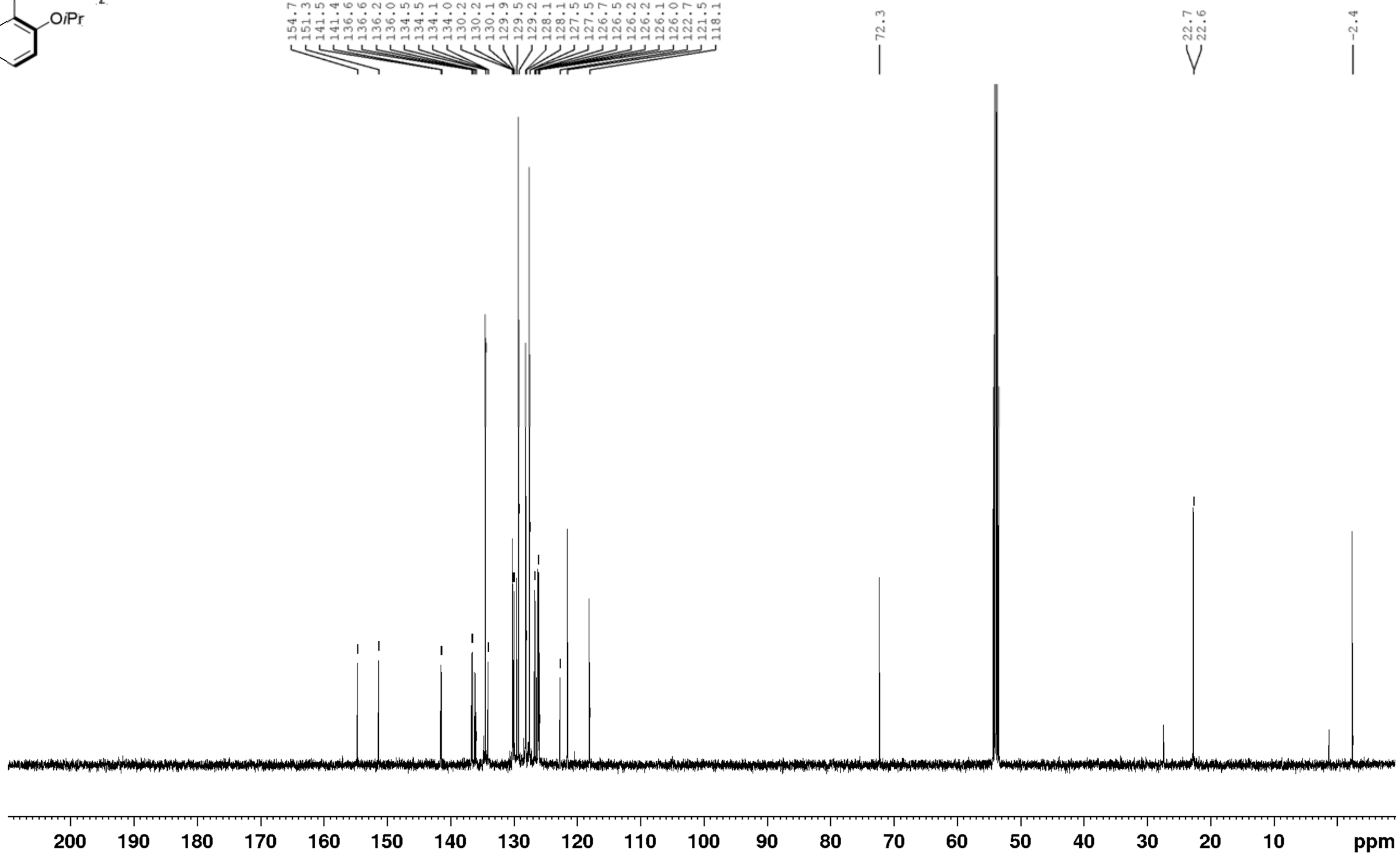

Figure S122. $^{1}H/^{29}Si$ HMQC NMR (500/99 MHz, CD_2Cl_2, optimized for J = 7 Hz) of (S)-[(2'-Isopropoxy-6,6'-diphenyl-[1,1'-binaphthalen]-2-yl)oxy](methyl)diphenylsilane [(S)-**3sa**].

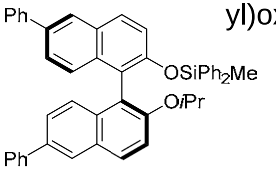

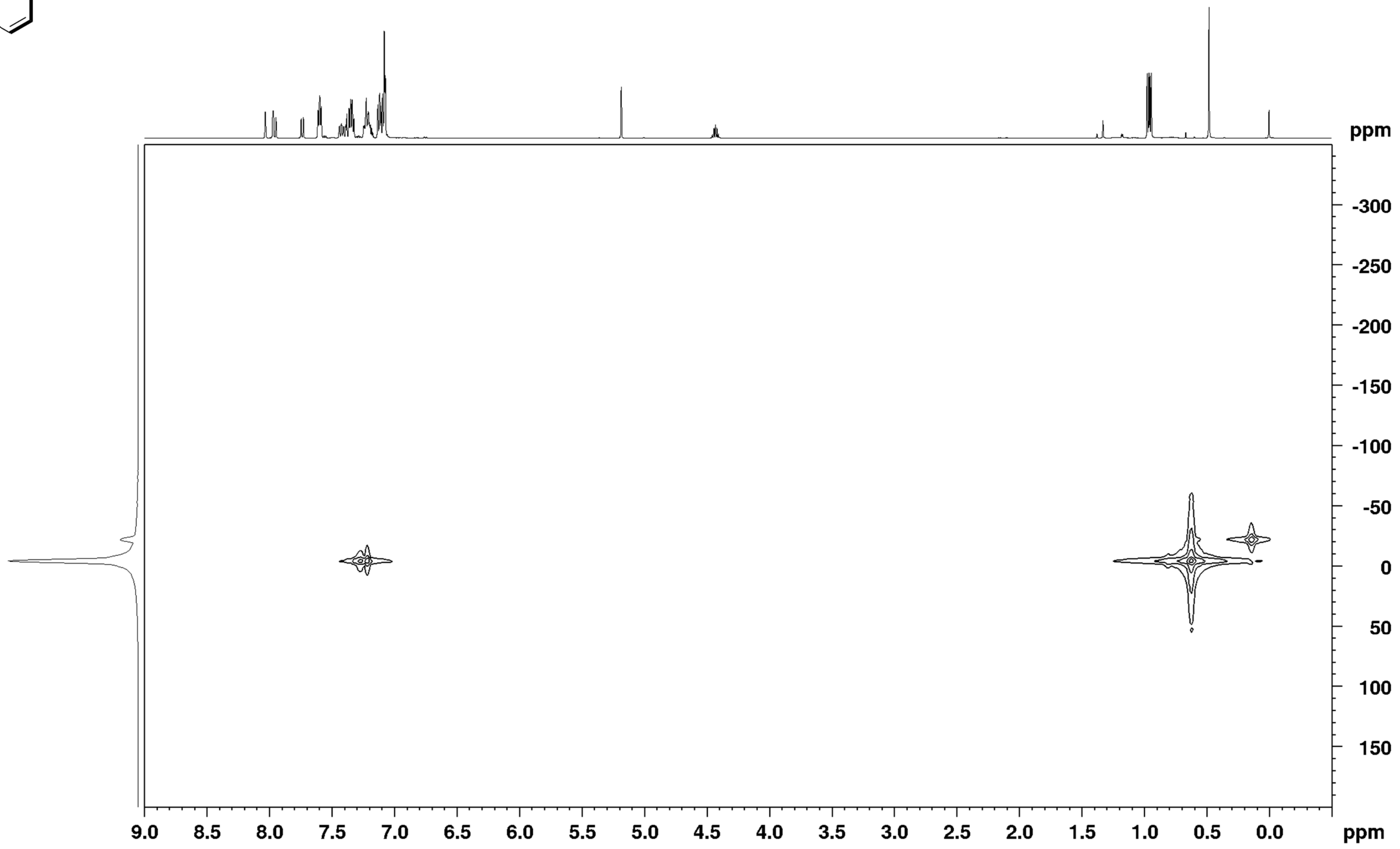

Figure S123. ^{1}H NMR (500 MHz, $CDCl_3$) of (*R*)-2'-Isopropoxy-6,6'-diphenyl-[1,1'-binaphthalen]-2-ol [(*R*)-**1s**].

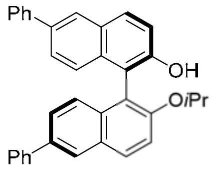

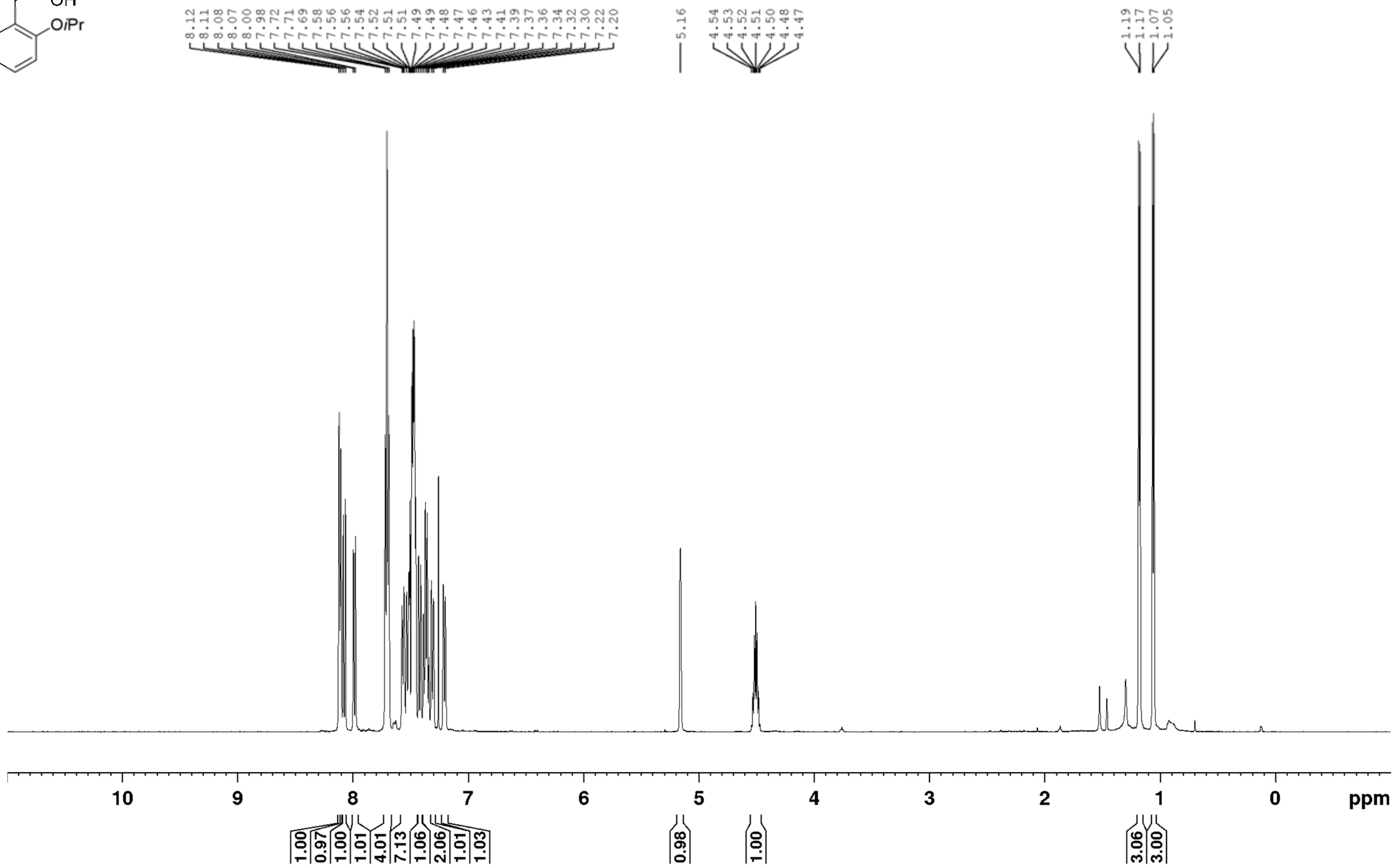

Figure S124. $^{13}C\{^{1}H\}$ NMR (126 MHz, $CDCl_3$) of (*R*)-2'-Isopropoxy-6,6'-diphenyl-[1,1'-binaphthalen]-2-ol [(*R*)-**1s**].

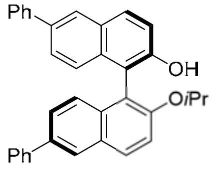

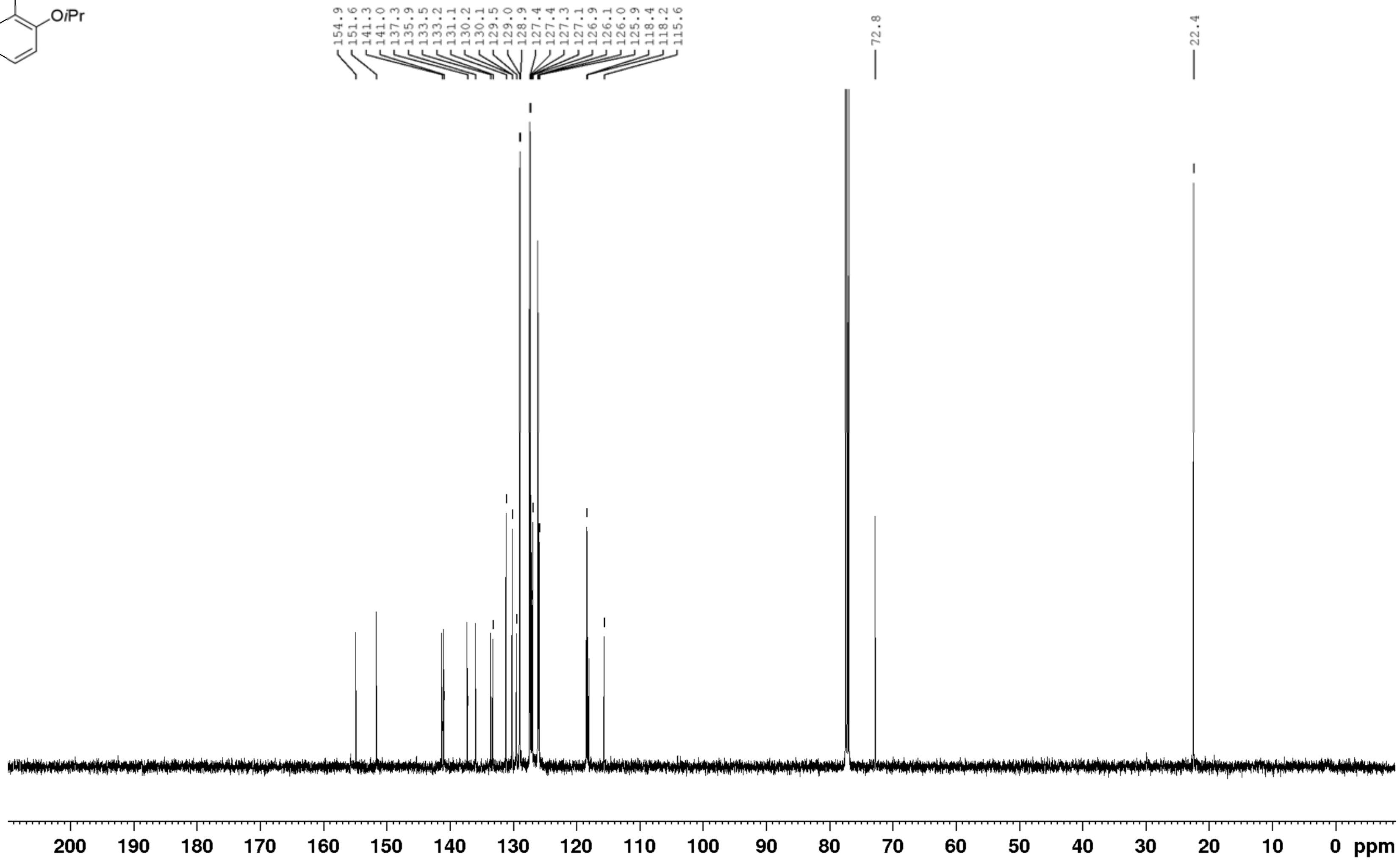

Figure S125. ^{1}H NMR (500 MHz, CD_2Cl_2) of *ent*-[(7'-Isopropoxy-2,2',3,3'-tetrahydro-1,1'-spirobi[inden]-7-yl)oxy](methyl)diphenyl-silane (*ent*-**3va**).

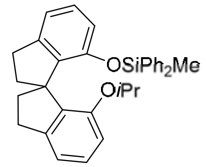

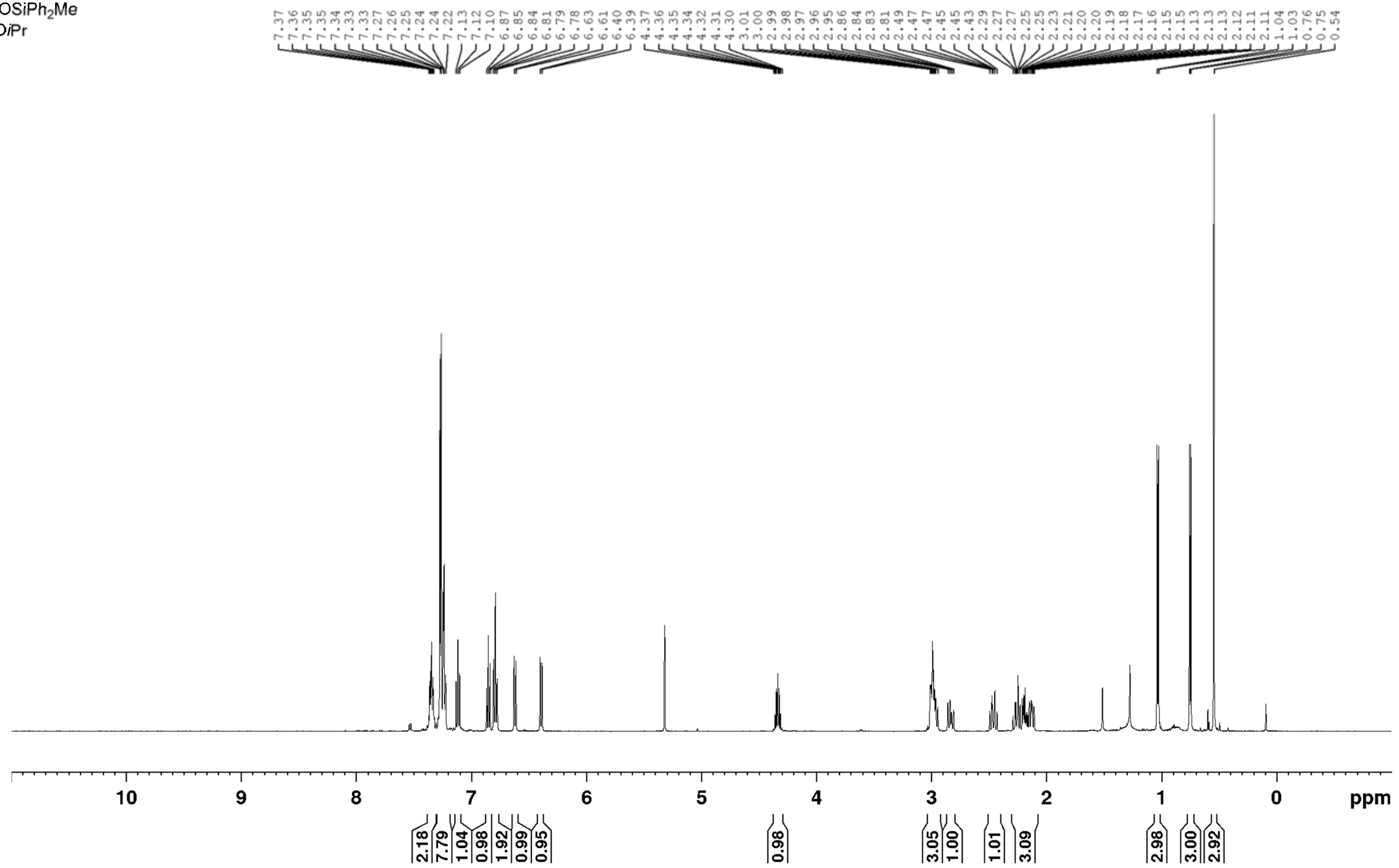

Figure S126. $^{13}C\{^{1}H\}$ NMR (126 MHz, CD_2Cl_2) of *ent*-[(7'-Isopropoxy-2,2',3,3'-tetrahydro-1,1'-spirobi[inden]-7-yl)oxy](methyl)diphenyl-silane (*ent*-**3va**).

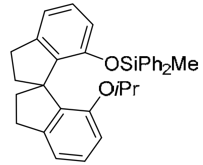

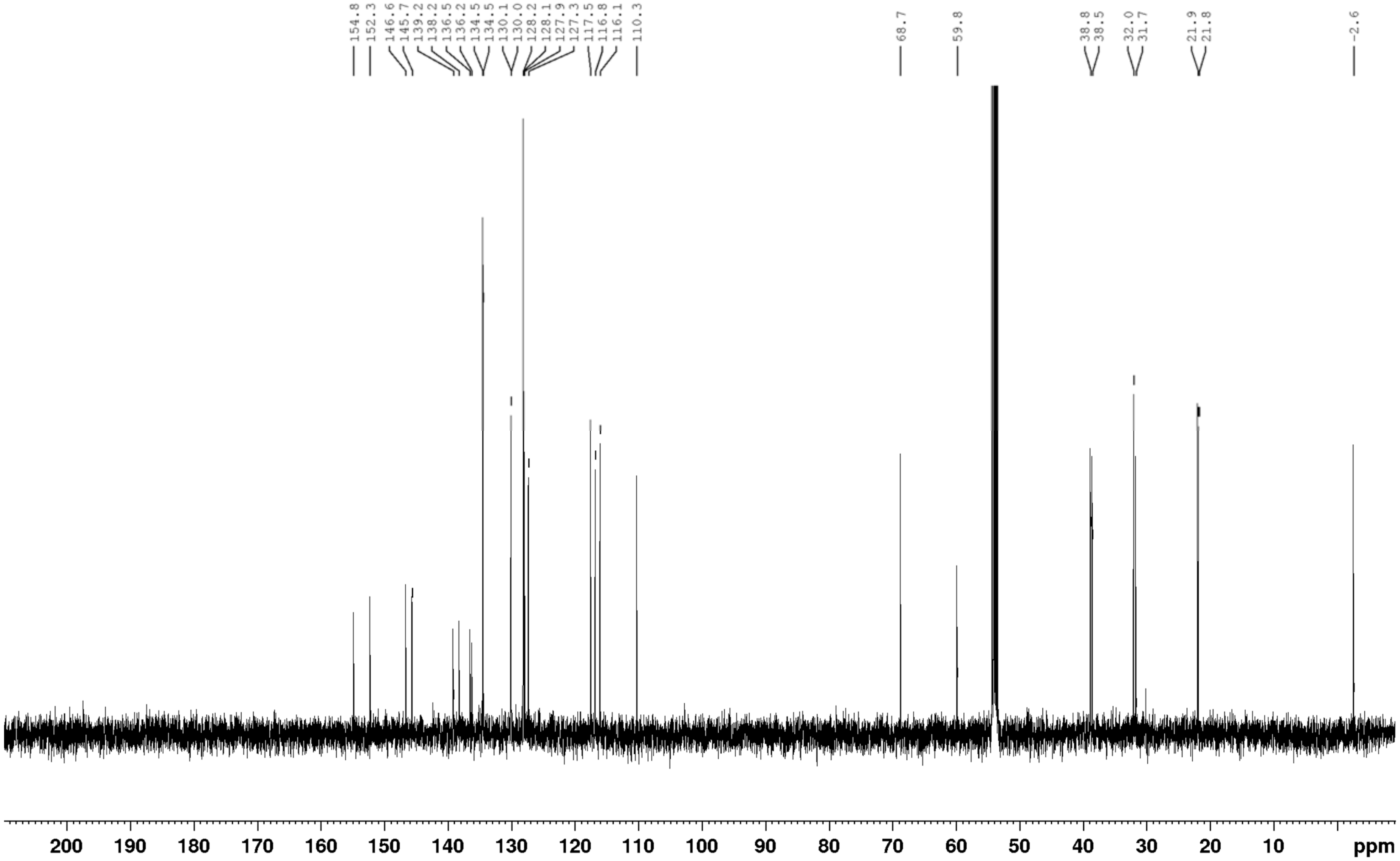

Figure S127. $^{1}H/^{29}Si$ HMQC NMR (500/99 MHz, CD_2Cl_2, optimized for J = 7 Hz) of *ent*-[(7'-Isopropoxy-2,2',3,3'-tetrahydro-1,1'-spirobi[inden]-7-yl)oxy](methyl)diphenyl-silane (*ent*-**3va**).

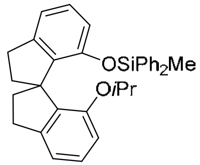

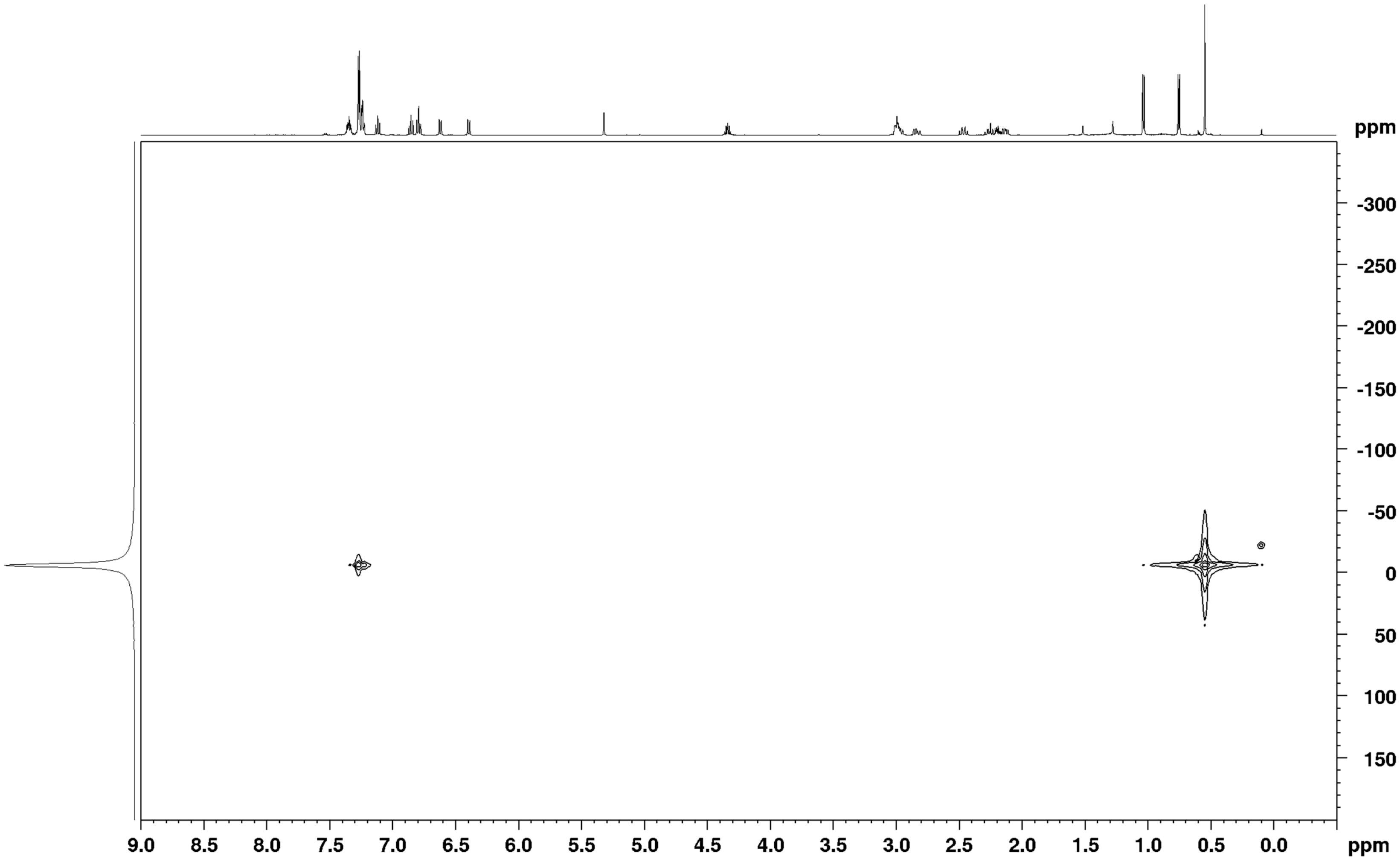

Figure S128. ^{1}H NMR (500 MHz, $CDCl_3$) of *ent*-7'-Isopropoxy-2,2',3,3'-tetrahydro-1,1'-spirobi[inden]-7-ol (*ent*-**1v**).

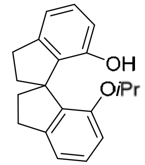

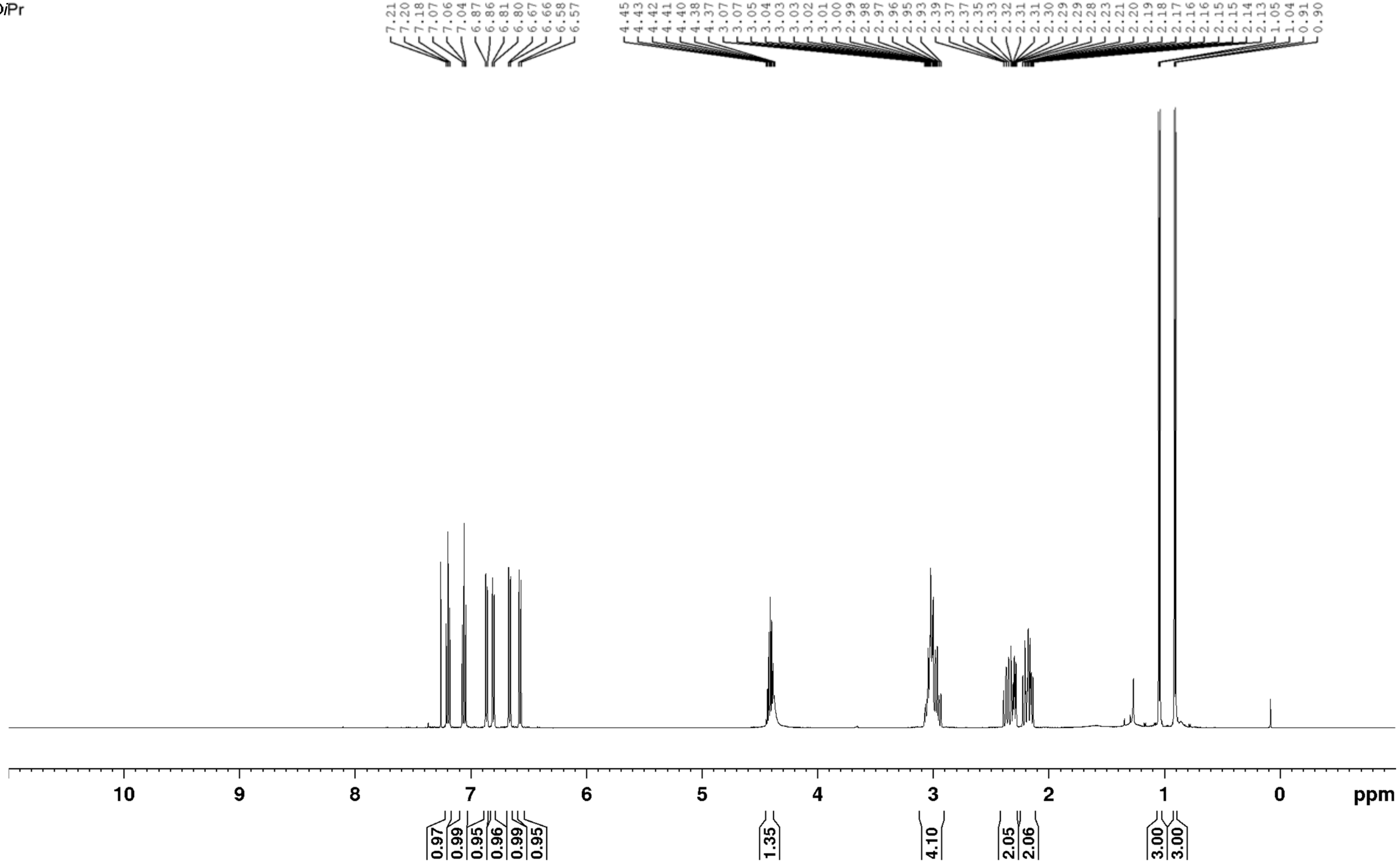

Figure S129. $^{13}C\{^{1}H\}$ NMR (126 MHz, $CDCl_3$) of *ent*-7'-Isopropoxy-2,2',3,3'-tetrahydro-1,1'-spirobi[inden]-7-ol (*ent*-**1v**).

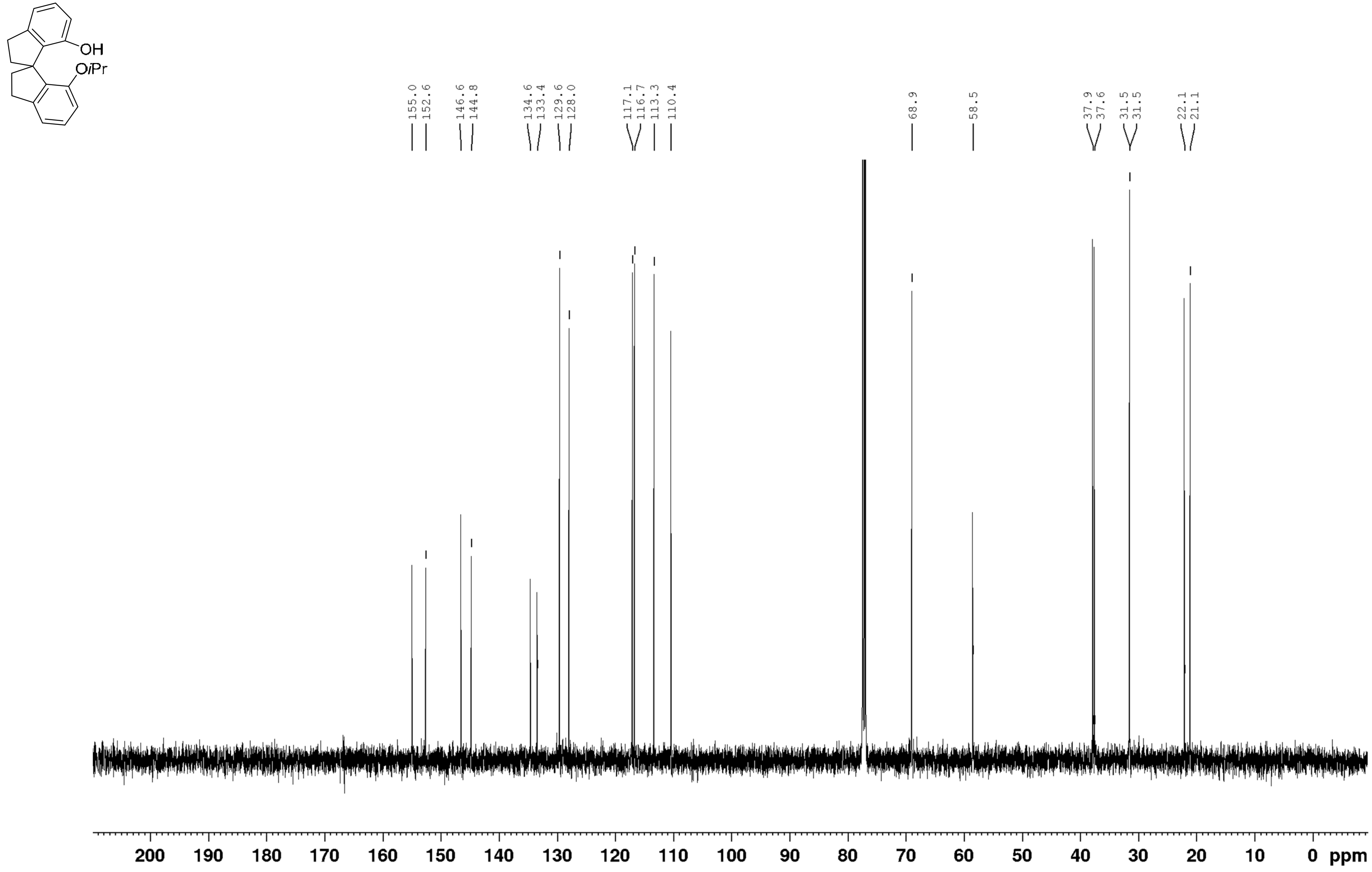

Figure S130. ^{1}H NMR (500 MHz, CD_2Cl_2) of (*S*)-[(2'-Isopropoxy-6,6'-dimethyl-[1,1'-biphenyl]-2-yl)oxy](methyl)diphenylsilane [(*S*)-**3wa**].

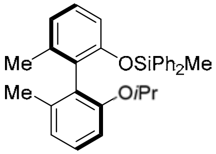

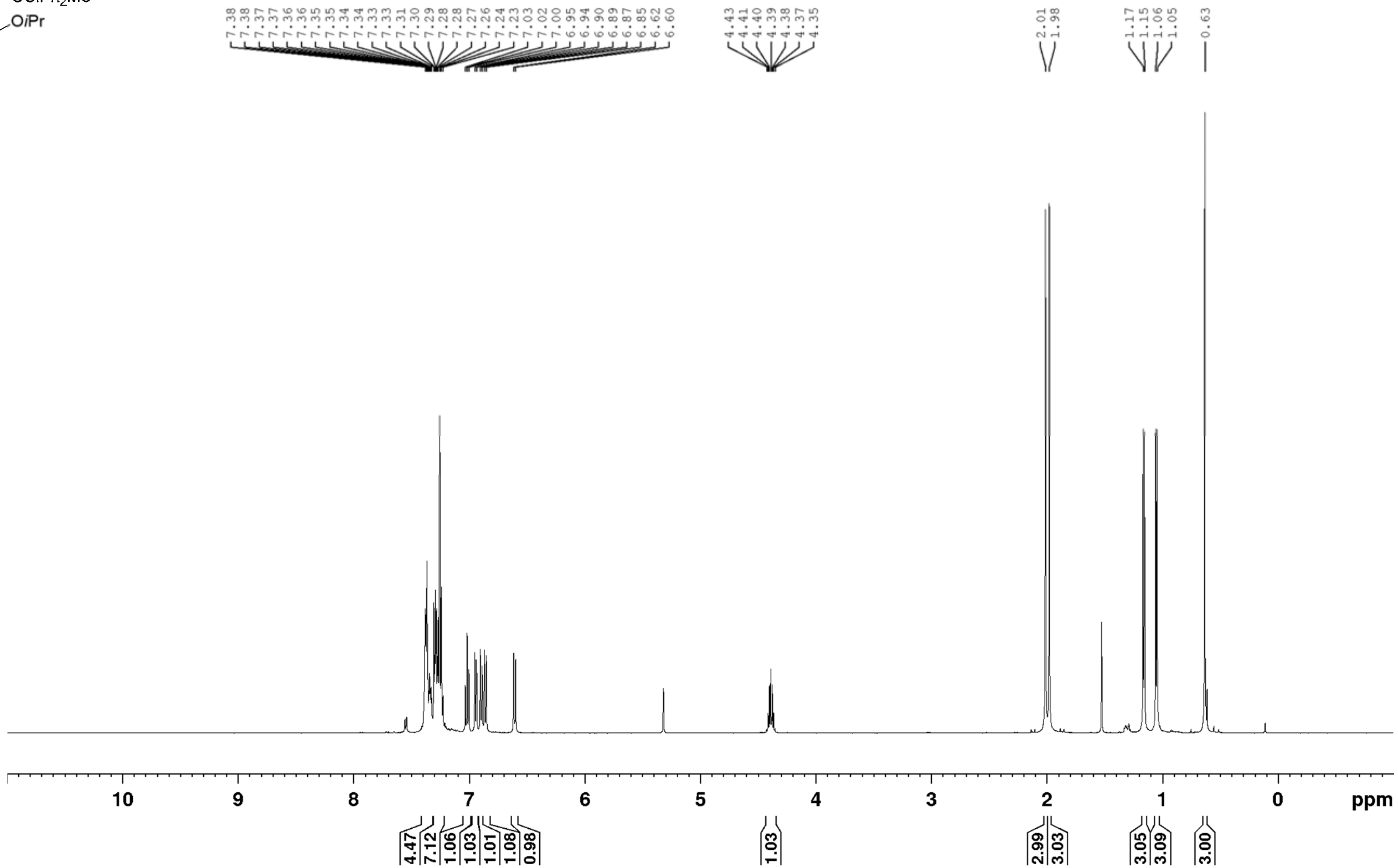

Figure S131. $^{13}C\{^{1}H\}$ NMR (126 MHz, CD_2Cl_2) of (*S*)-[(2'-Isopropoxy-6,6'-dimethyl-[1,1'-biphenyl]-2-yl)oxy](methyl)diphenylsilane [(*S*)-**3wa**].

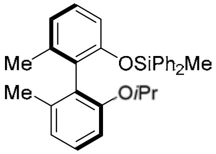

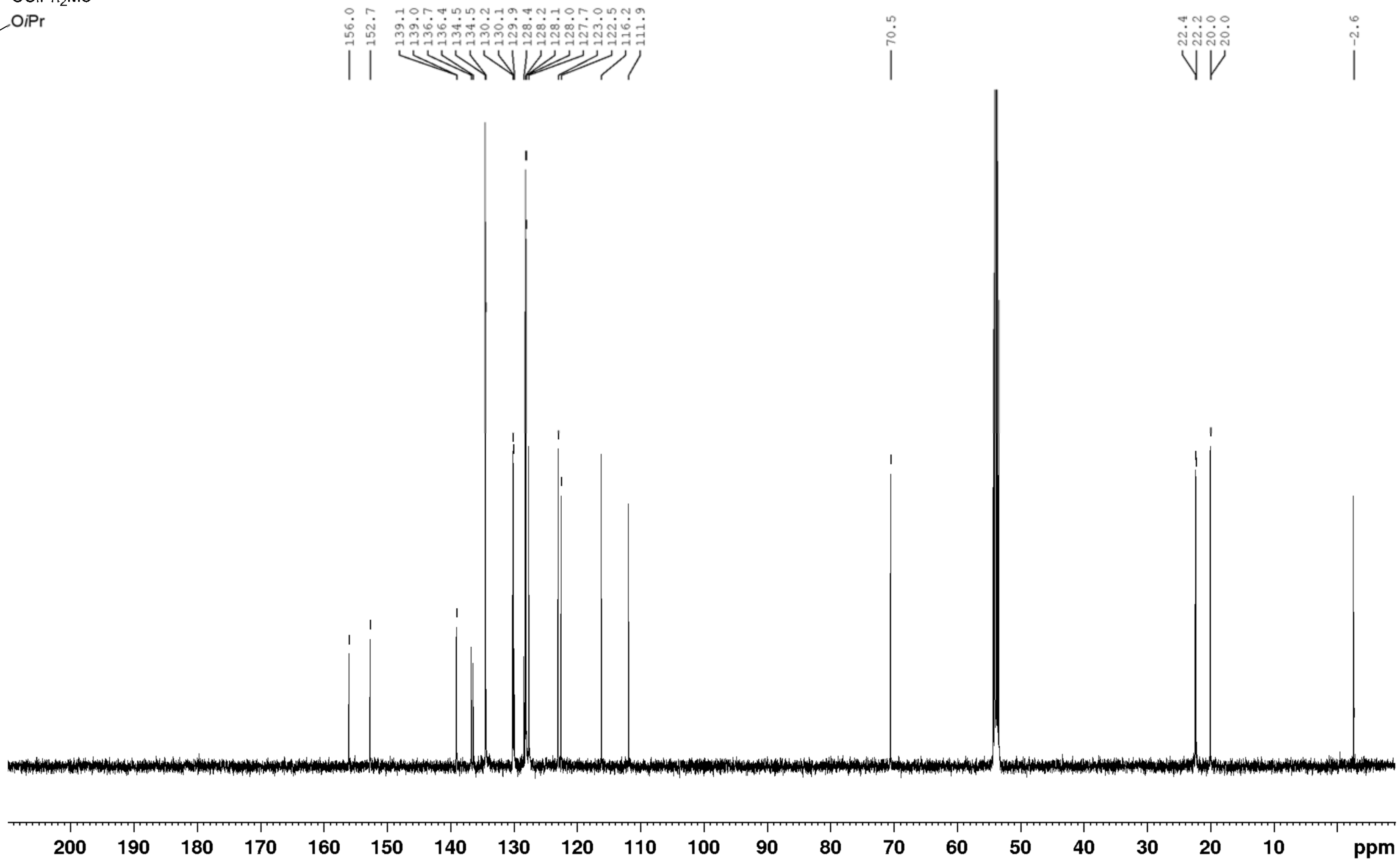

Figure S132. $^{1}H/^{29}Si$ HMQC NMR (500/99 MHz, CD_2Cl_2, optimized for J = 7 Hz) of (*S*)-[(2'-Isopropoxy-6,6'-dimethyl-[1,1'-biphenyl]-2-yl)oxy]-(methyl)diphenylsilane [(*S*)-**3wa**].

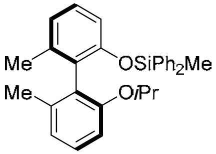

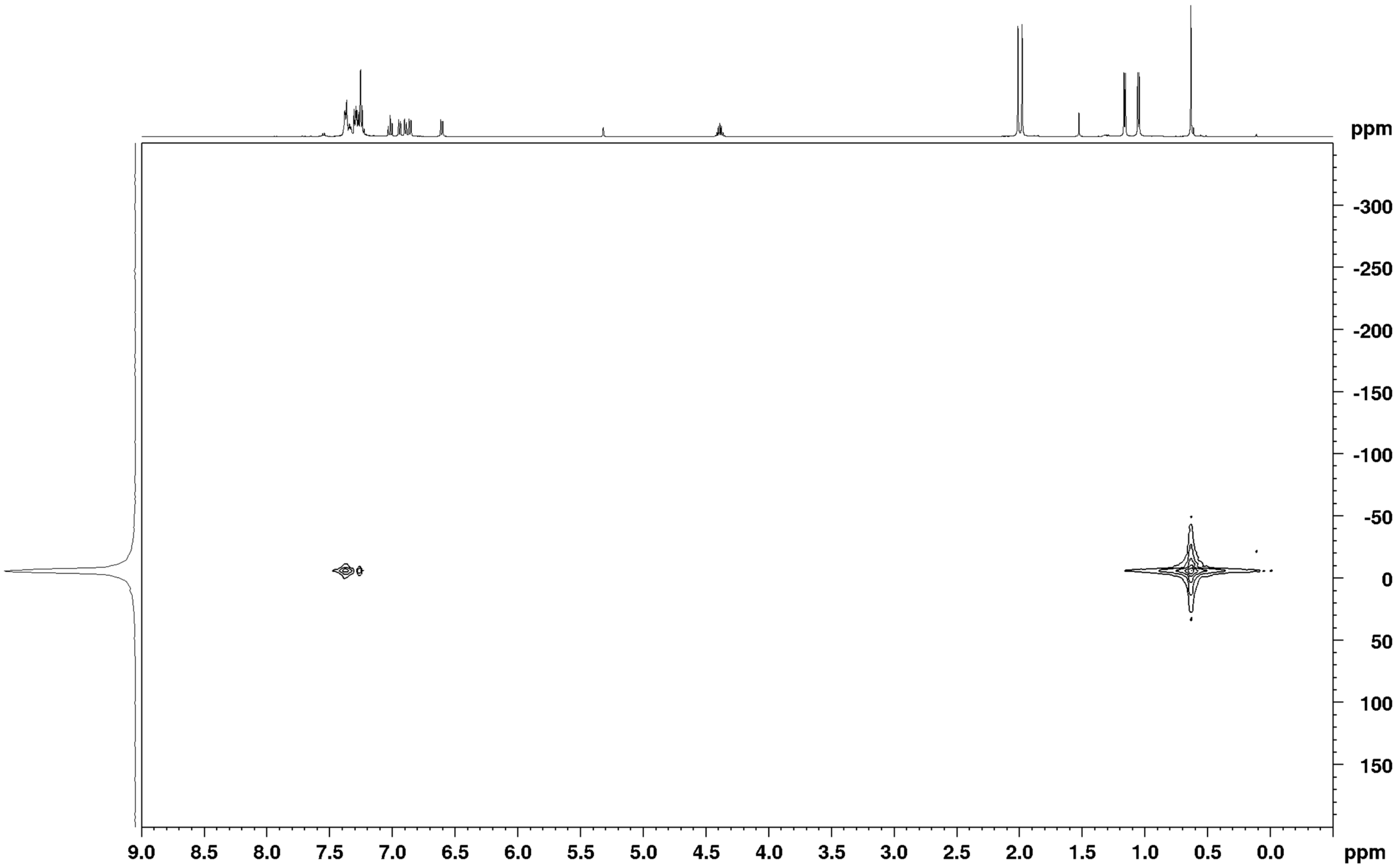

Figure S133. ^1H NMR (500 MHz, $CDCl_3$) of (*R*)-2'-Isopropoxy-6,6'-dimethyl-[1,1'-biphenyl]-2-ol [(*R*)-**1w**].

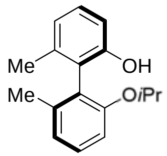

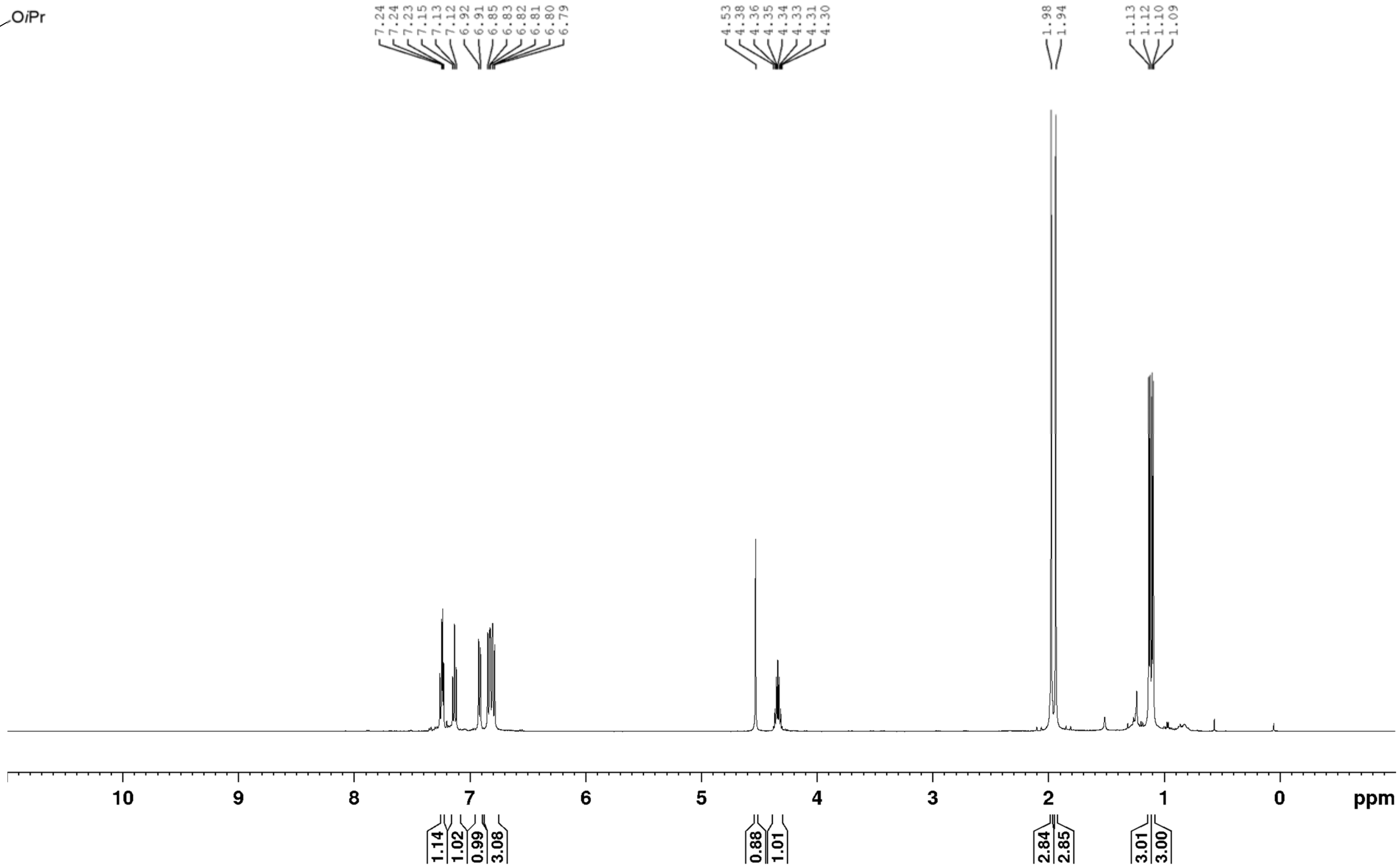

Figure S134. $^{13}C\{^{1}H\}$ NMR (126 MHz, $CDCl_3$) of (*R*)-2'-Isopropoxy-6,6'-dimethyl-[1,1'-biphenyl]-2-ol [(*R*)-**1w**].

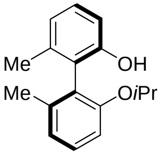

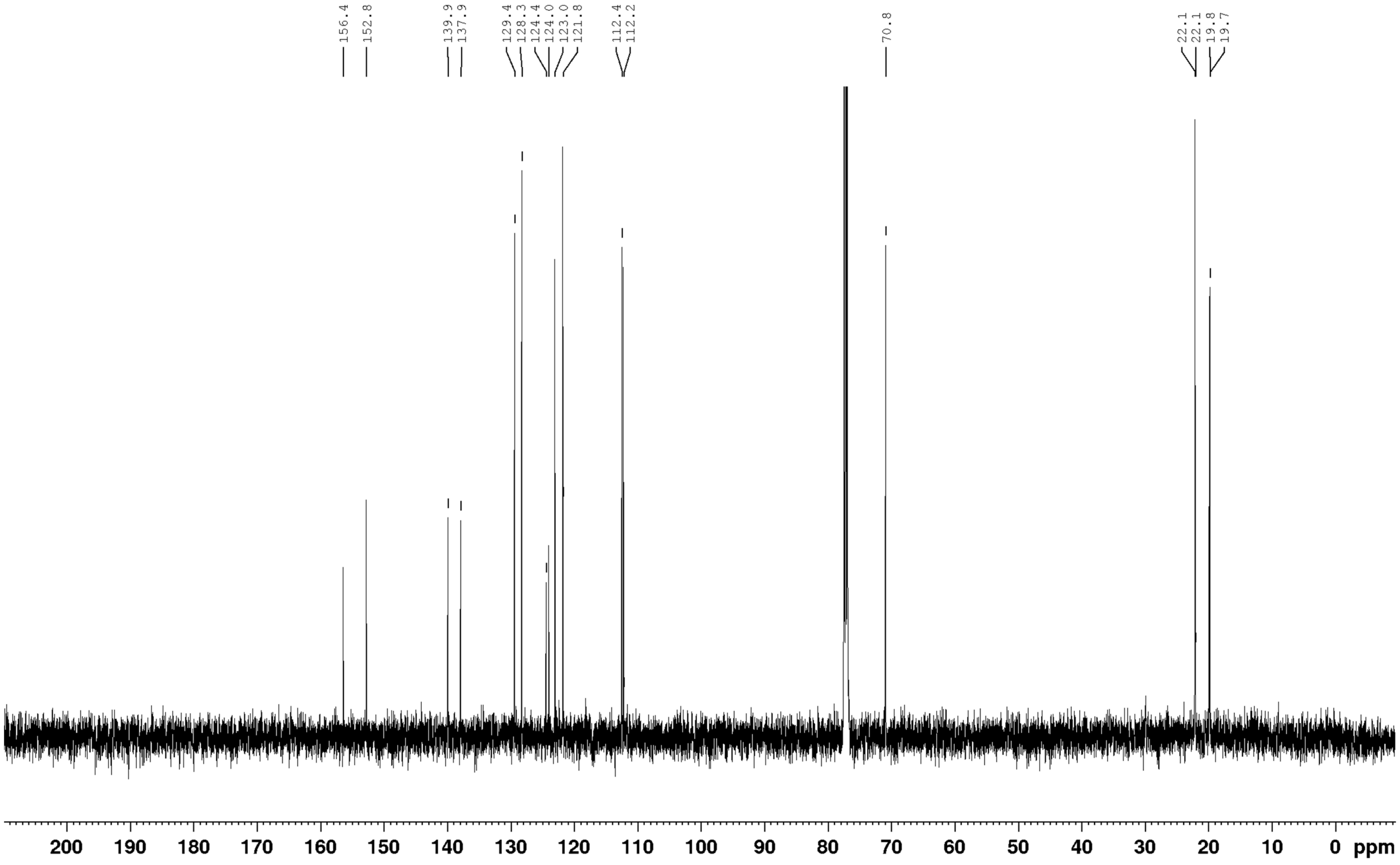

Figure S135. ^{1}H NMR (500 MHz, CD_2Cl_2) of (*S*)-[(2',6-Diethyl-6'-isopropoxy-[1,1'-biphenyl]-2-yl)oxy](methyl)diphenylsilane [(*S*)-**3xa**].

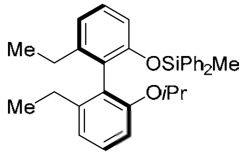

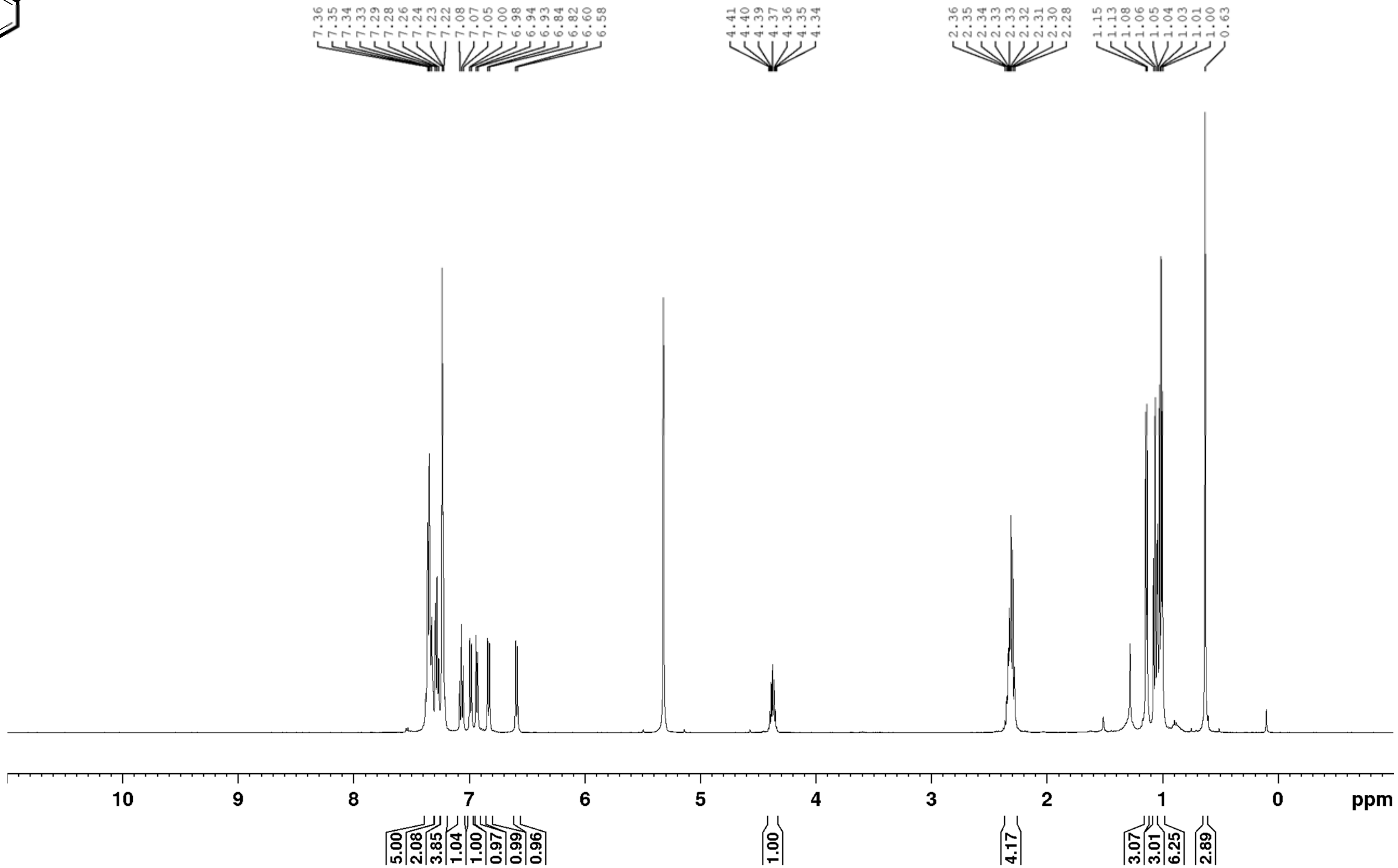

Figure S136. $^{13}C\{^{1}H\}$ NMR (126 MHz, CD_2Cl_2) of (*S*)-[(2',6-Diethyl-6'-isopropoxy-[1,1'-biphenyl]-2-yl)oxy](methyl)diphenylsilane [(*S*)-**3xa**].

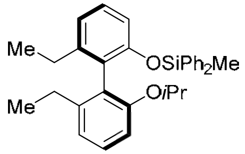

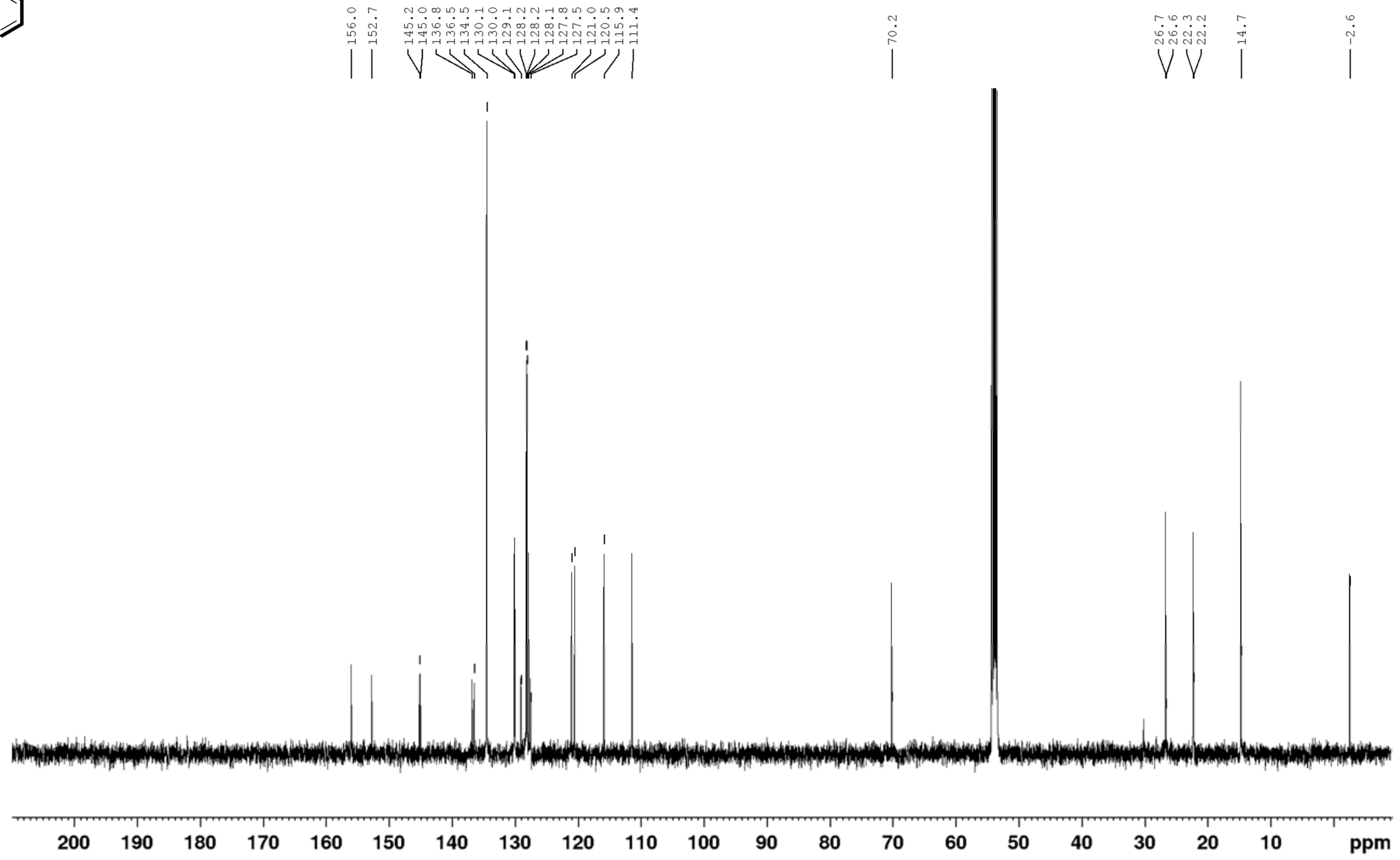

Figure S137. $^{1}H/^{29}Si$ HMQC NMR (500/99 MHz, CD_2Cl_2, optimized for J = 7 Hz) of (*S*)-[(2',6-Diethyl-6'-isopropoxy-[1,1'-biphenyl]-2-yl)oxy](methyl)diphenylsilane [(*S*)-**3xa**].

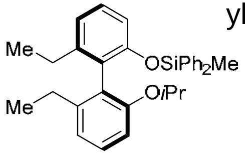

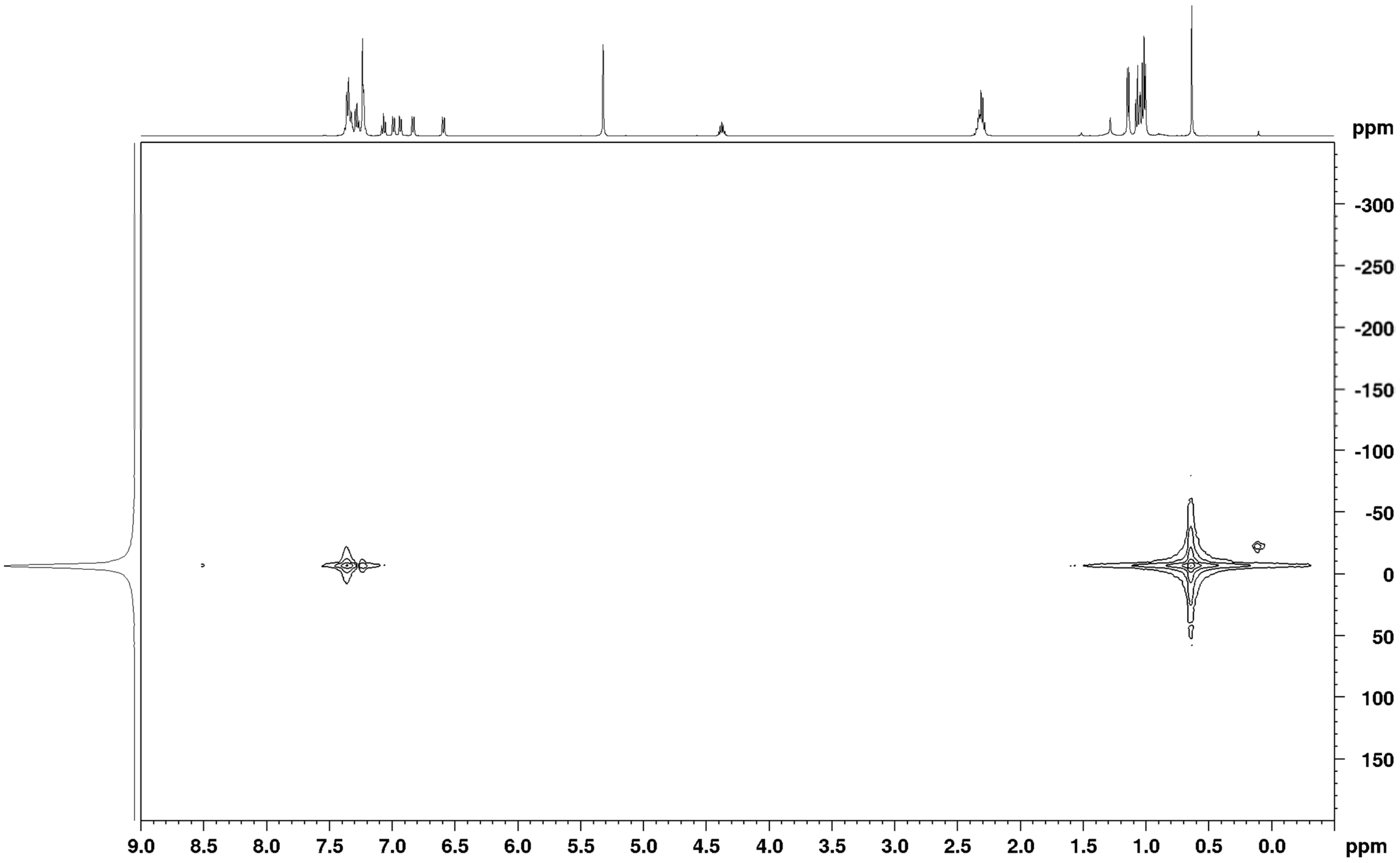

Figure S138. ^{1}H NMR (500 MHz, $CDCl_3$) of (*R*)-2',6-Diethyl-6'-isopropoxy-[1,1'-biphenyl]-2-ol [(*R*)-**1x**].

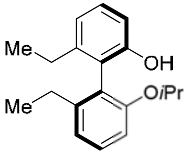

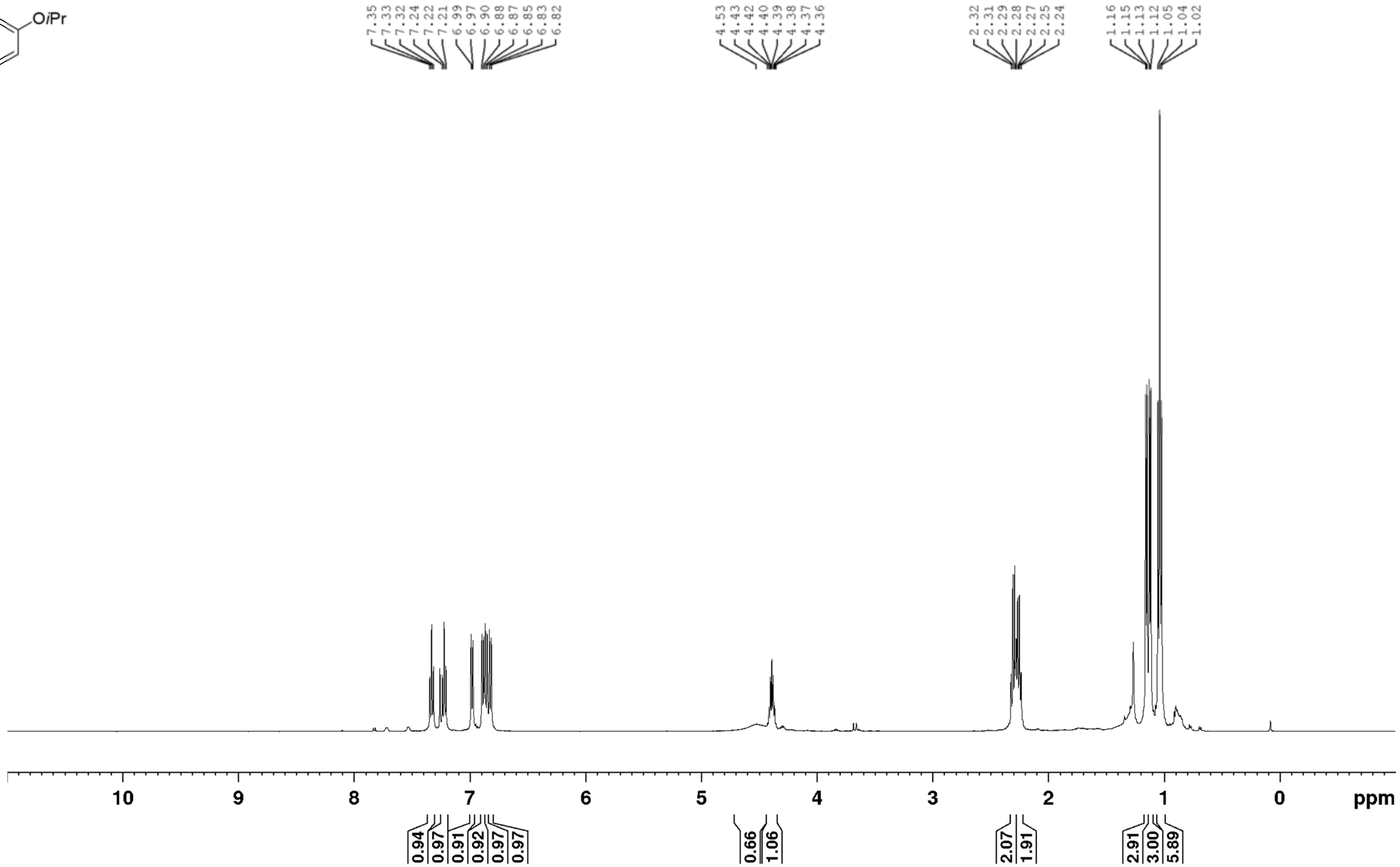

Figure S139. $^{13}C\{^{1}H\}$ NMR (126 MHz, $CDCl_3$) of (*R*)-2',6-Diethyl-6'-isopropoxy-[1,1'-biphenyl]-2-ol [(*R*)-**1x**].

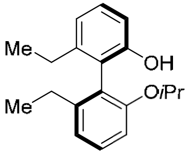

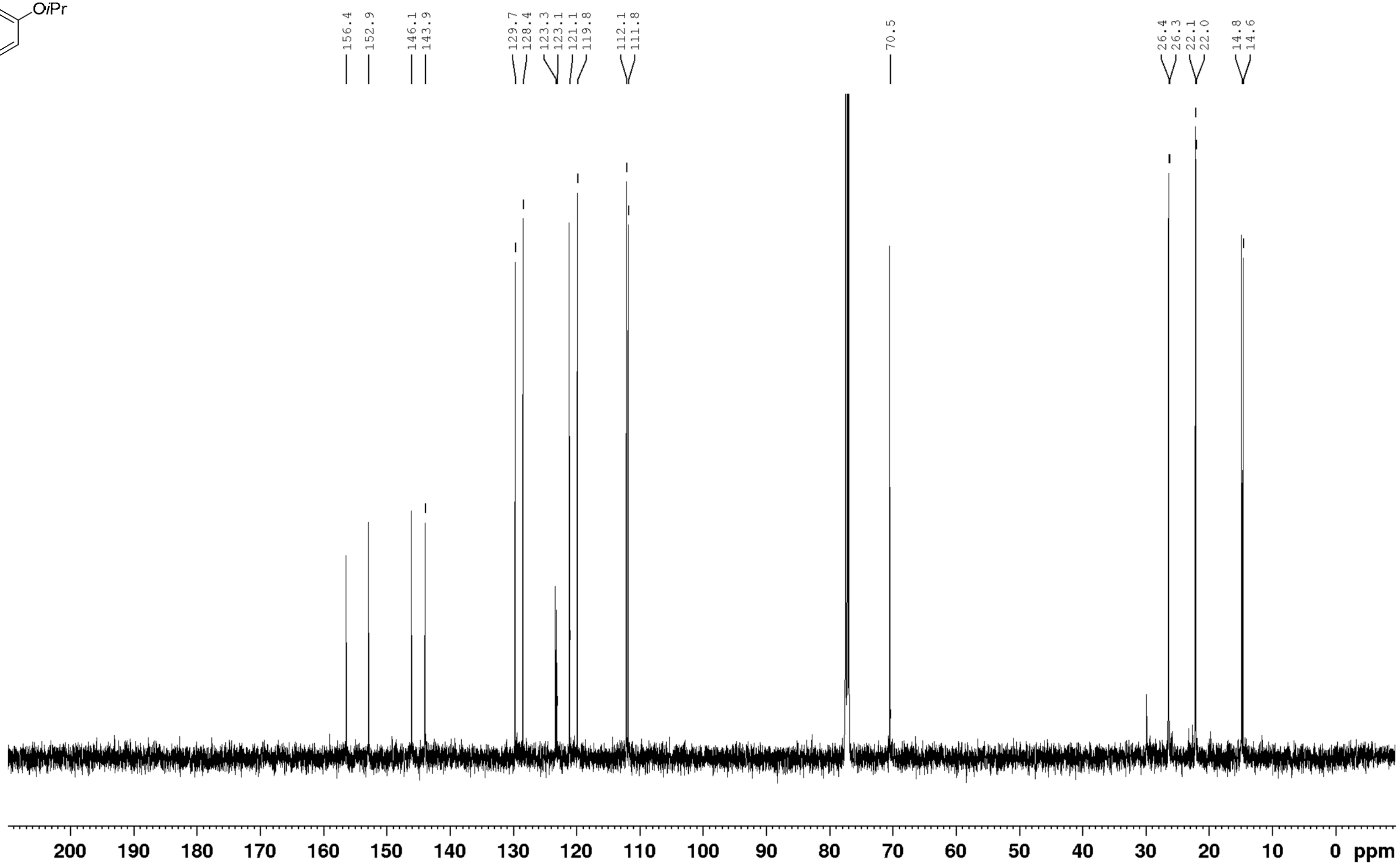

Figure S140. ^{1}H NMR (500 MHz, CD_2Cl_2) of (*S*)-[(2',6-Diisobutyl-6'-isopropoxy-[1,1'-biphenyl]-2-yl)oxy](methyl)diphenylsilane [(*S*)-**3ya**].

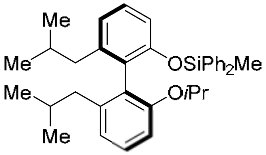

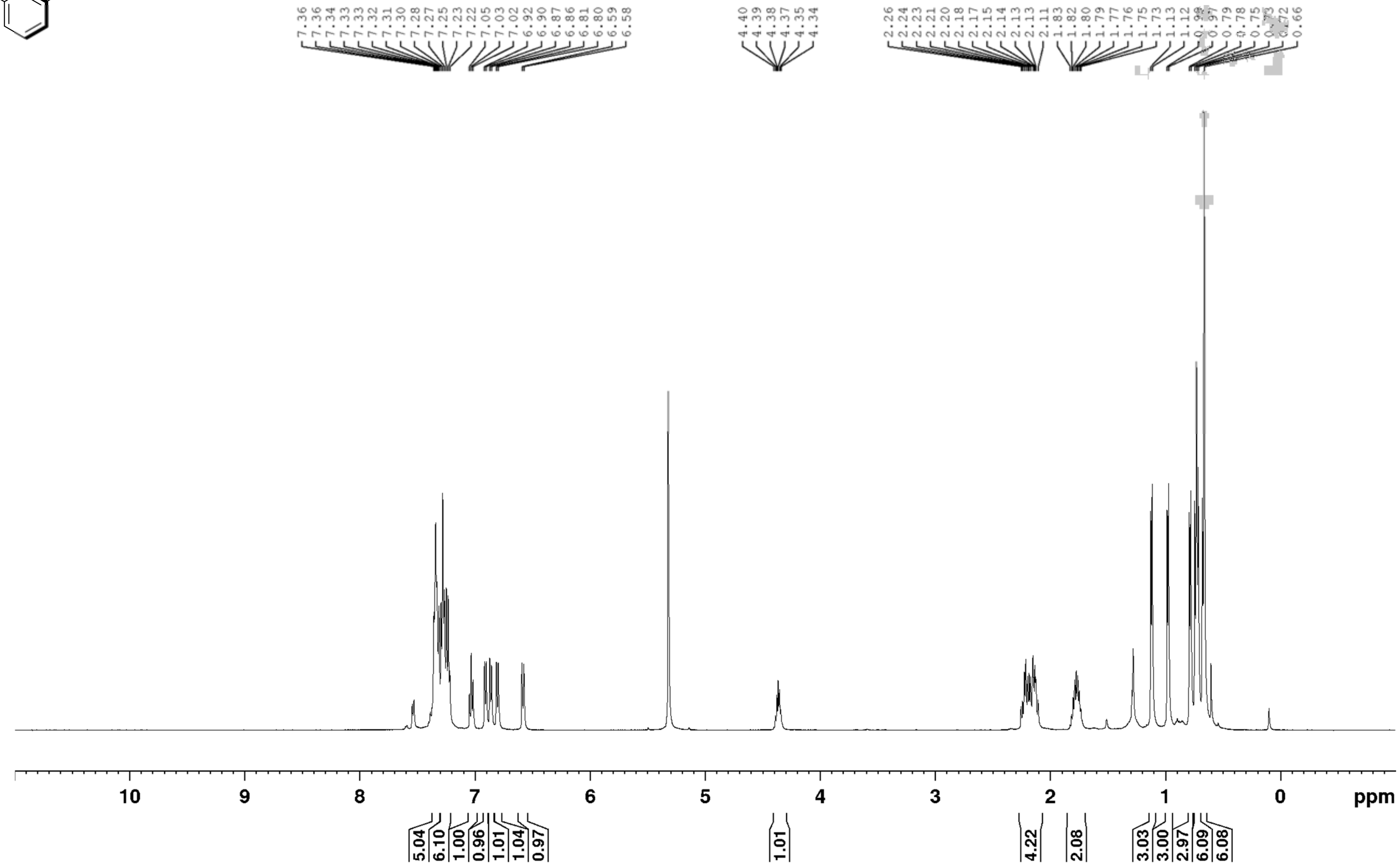

Figure S141. $^{13}C\{^{1}H\}$ NMR (126 MHz, CD_2Cl_2) of (*S*)-[(2',6-Diisobutyl-6'-isopropoxy-[1,1'-biphenyl]-2-yl)oxy](methyl)diphenylsilane [(*S*)-**3ya**].

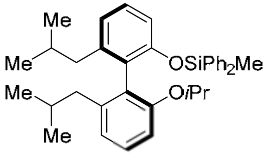

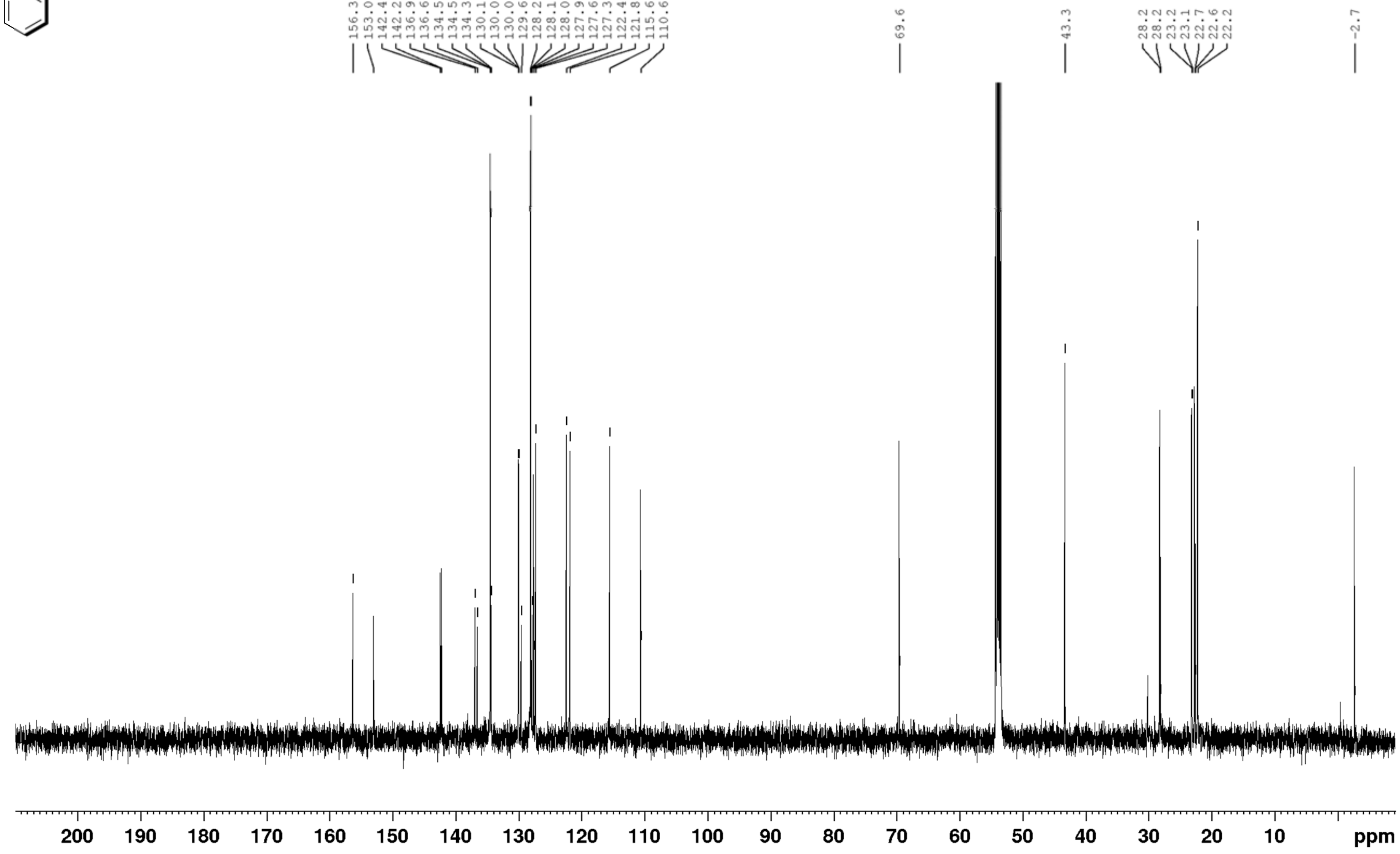

Figure S142. $^1H/^{29}Si$ HMQC NMR (500/99 MHz, CD_2Cl_2, optimized for J = 7 Hz) of (S)-[(2',6-Diisobutyl-6'-isopropoxy-[1,1'-biphenyl]-2-yl)oxy]-(methyl)diphenylsilane [(S)-**3ya**].

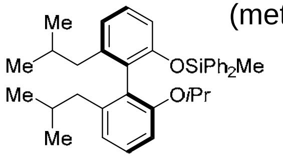

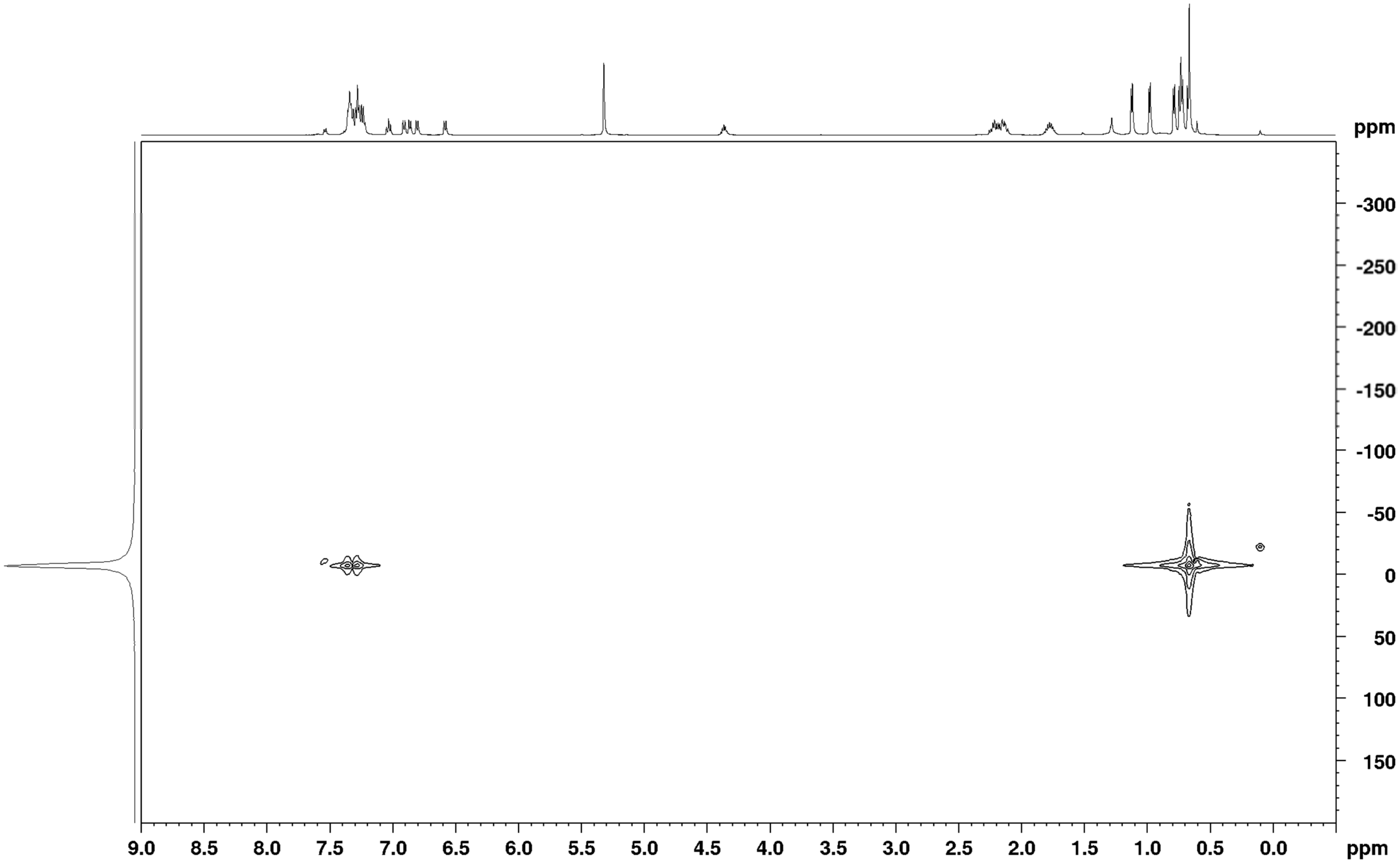

Figure S143. ^{1}H NMR (500 MHz, $CDCl_3$) of (*R*)-2',6-Diisobutyl-6'-isopropoxy-[1,1'-biphenyl]-2-ol [(*R*)-**1y**].

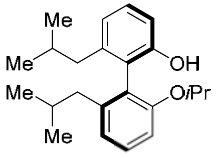

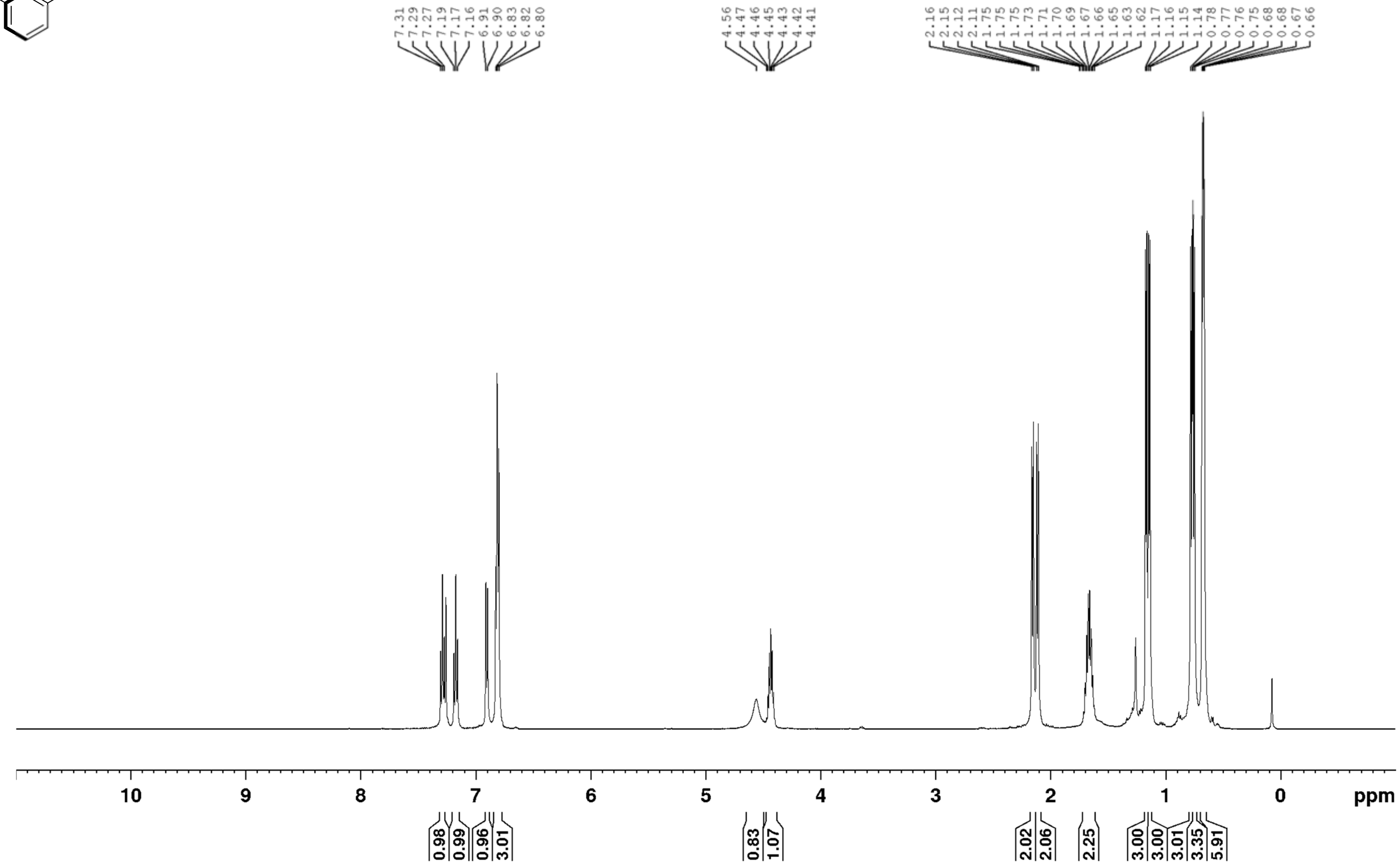

Figure S144. $^{13}C\{^1H\}$ NMR (126 MHz, $CDCl_3$) of (*R*)-2',6-Diisobutyl-6'-isopropoxy-[1,1'-biphenyl]-2-ol [(*R*)-**1y**].

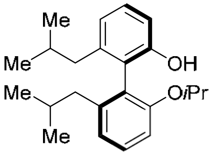

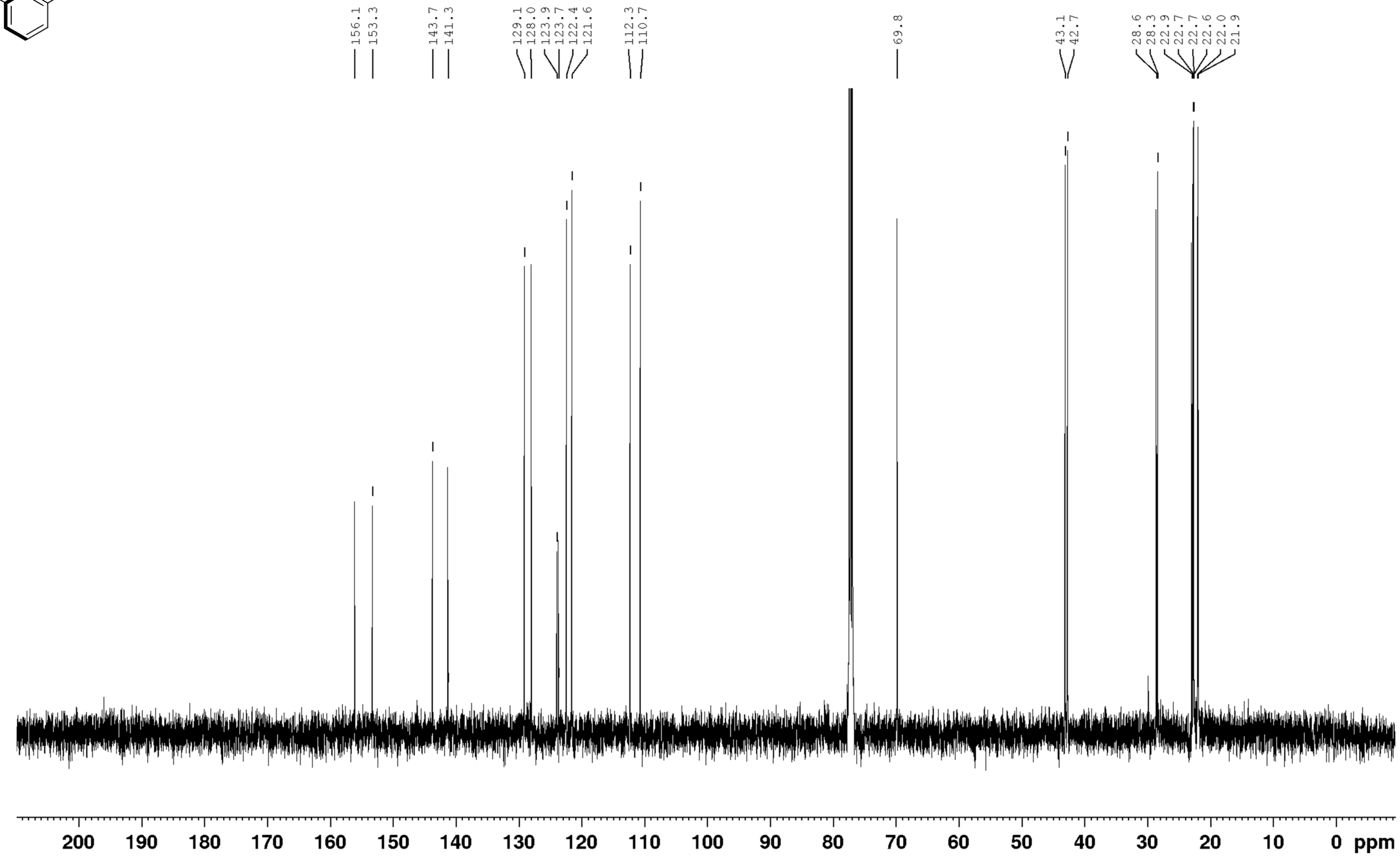

Figure S145. ^{1}H NMR (500 MHz, CD_2Cl_2) of (*S*)-[(6'-Isopropoxy-2',3',5,6-tetramethyl-[1,1'-biphenyl]-2-yl)oxy](methyl)diphenylsilane [(*S*)-**3za**].

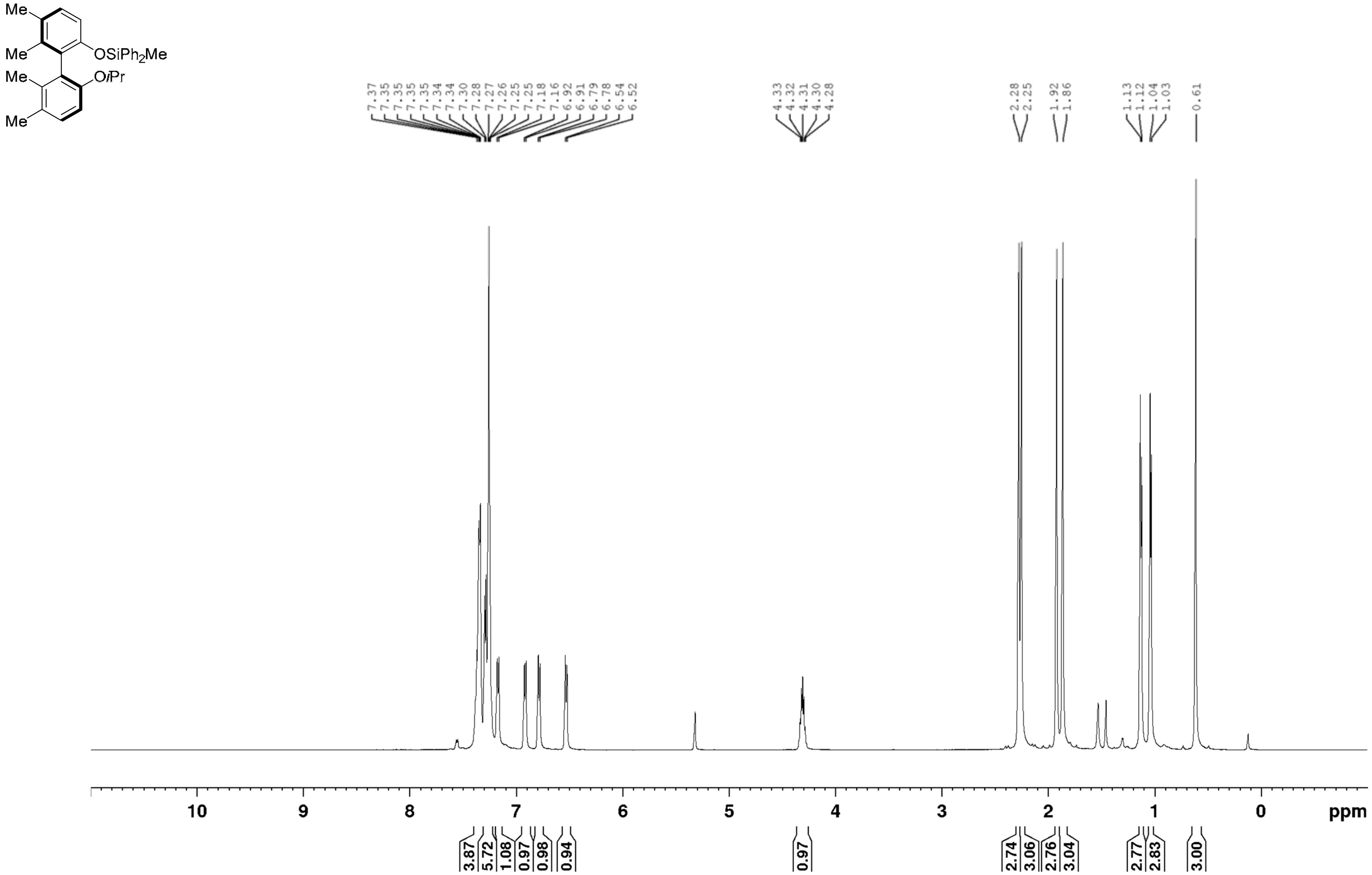

Figure S146. $^{13}C\{^1H\}$ NMR (126 MHz, CD_2Cl_2) of (*S*)-[(6'-Isopropoxy-2',3',5,6-tetramethyl-[1,1'-biphenyl]-2-yl)oxy](methyl)diphenylsilane [(*S*)-**3za**].

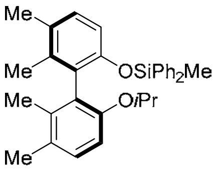

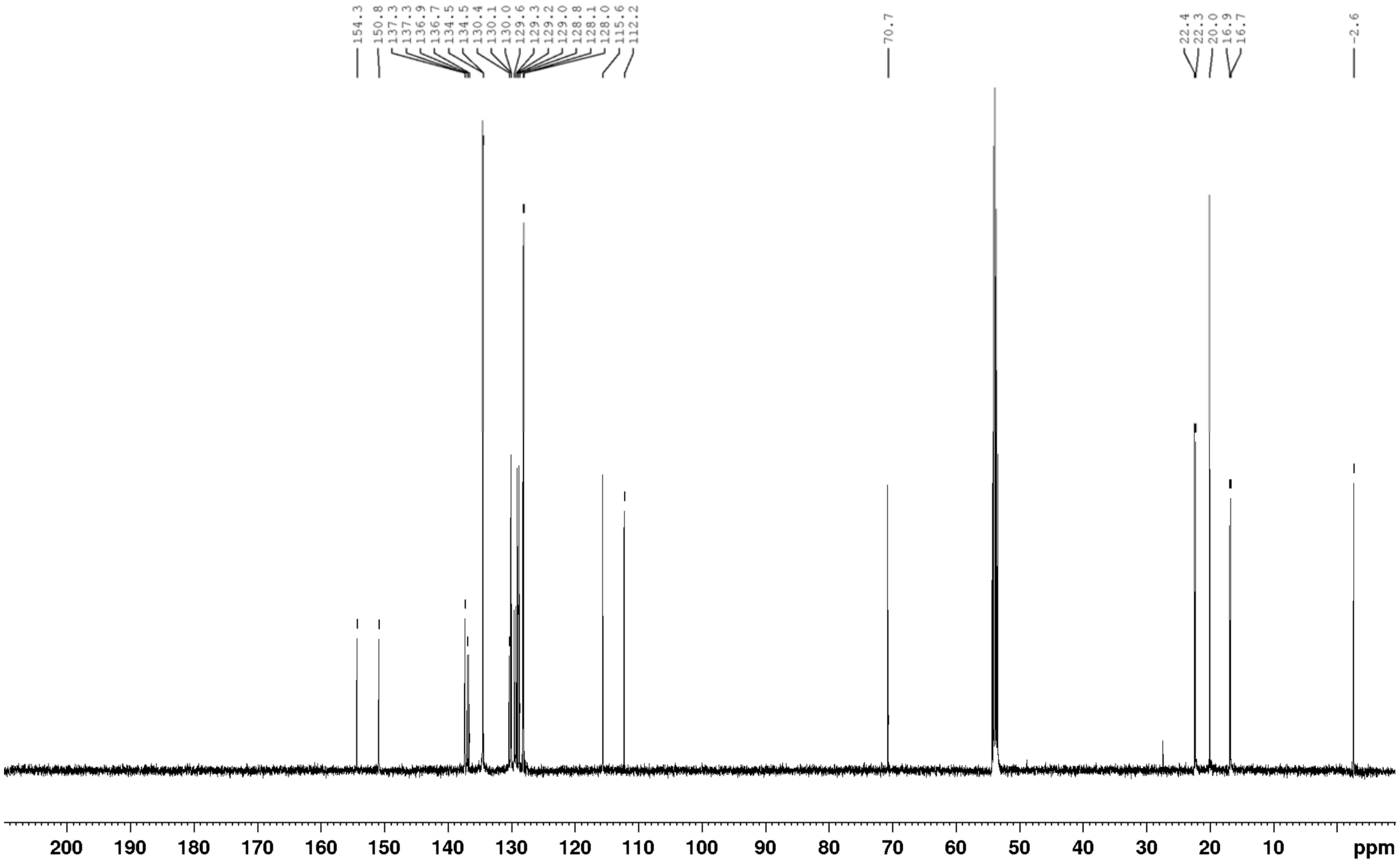

Figure S147. $^{1}H/^{29}Si$ HMQC NMR (500/99 MHz, CD_2Cl_2, optimized for J = 7 Hz) of (S)-[(6'-Isopropoxy-2',3',5,6-tetramethyl-[1,1'-biphenyl]-2-yl)oxy](methyl)diphenylsilane [(S)-**3za**].

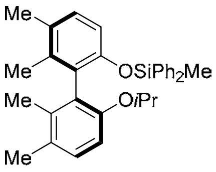

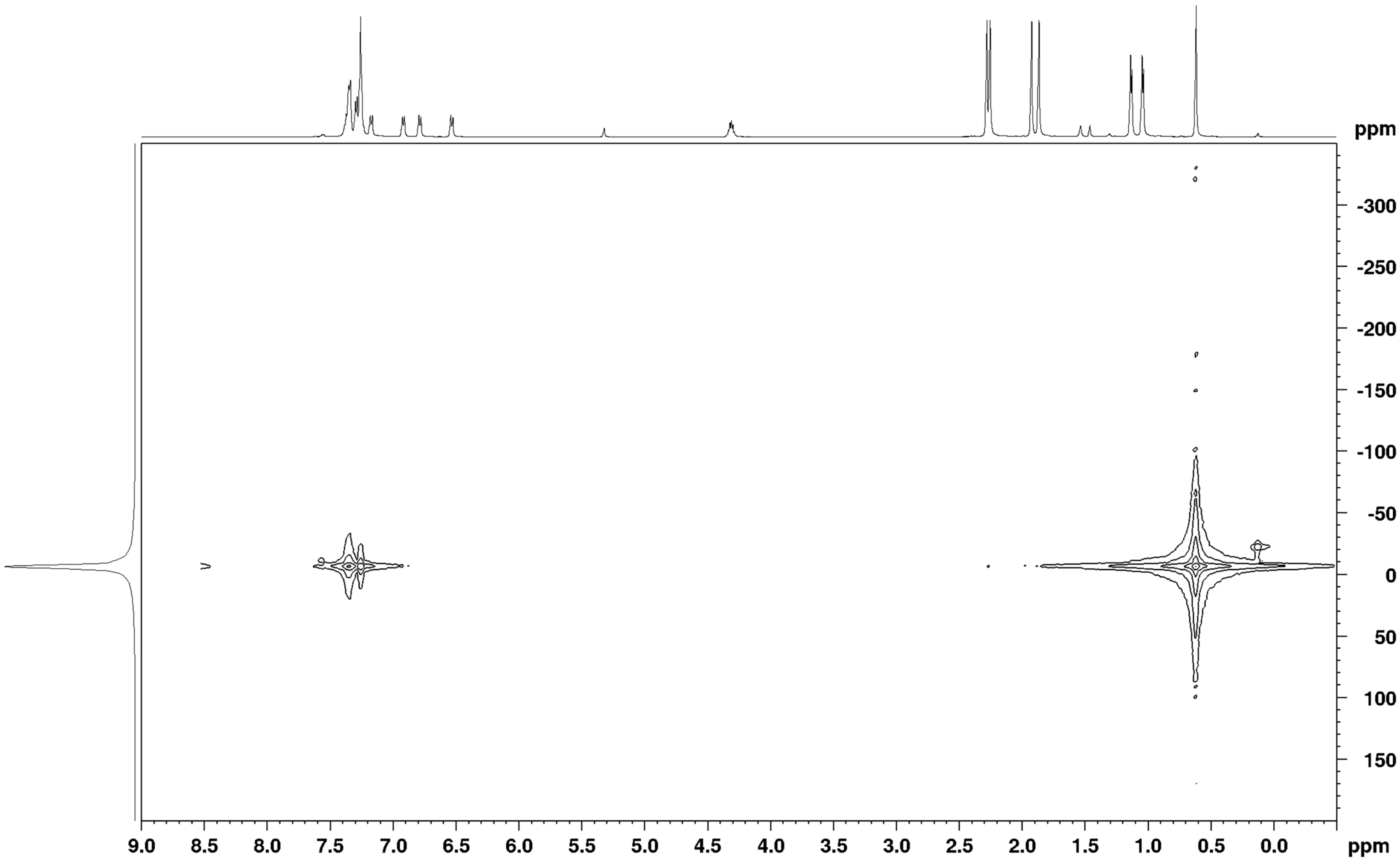

Figure S148. ^{1}H NMR (500 MHz, $CDCl_3$) of (*R*)-6'-Isopropoxy-2',3',5,6-tetramethyl-[1,1'-biphenyl]-2-ol [(*R*)-**1z**].

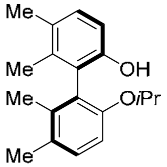

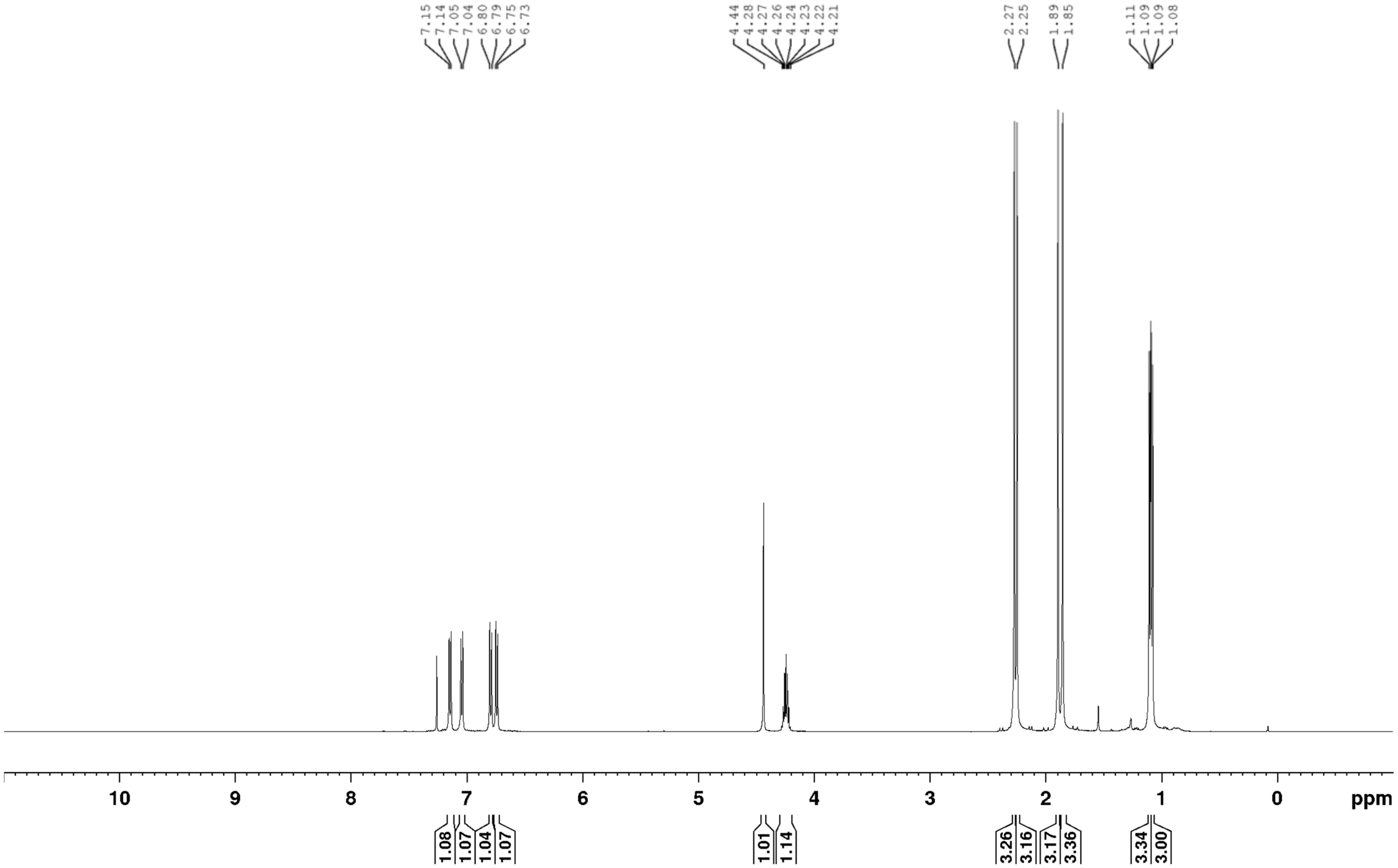

Figure S149. $^{13}C\{^{1}H\}$ NMR (126 MHz, $CDCl_3$) of (*R*)-6'-Isopropoxy-2',3',5,6-tetramethyl-[1,1'-biphenyl]-2-ol [(*R*)-**1z**].

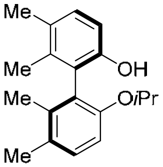

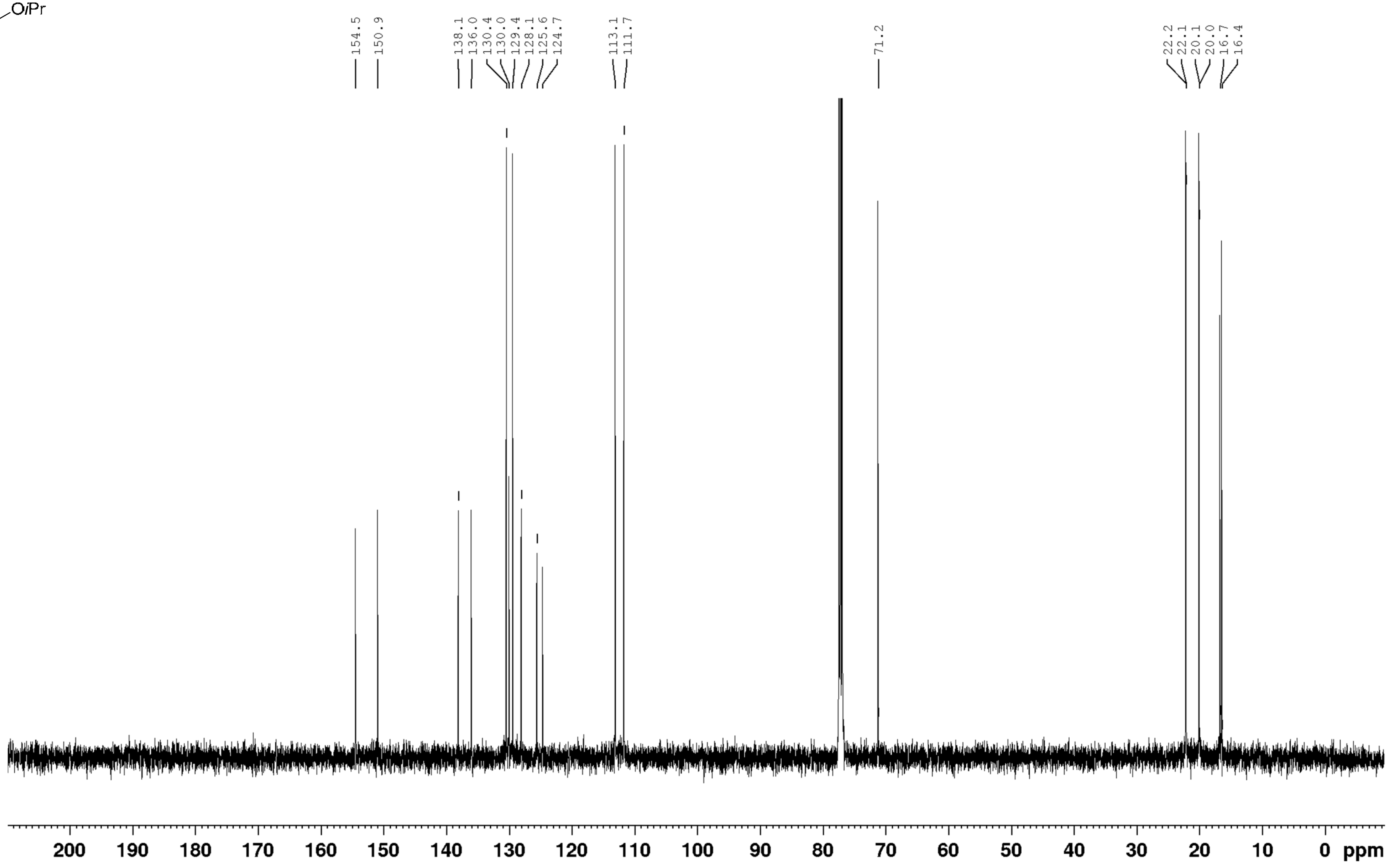

Figure S150. ^1H NMR (500 MHz, CD_2Cl_2) of (*S*)-[(2'-Isopropoxy-4,4',6,6'-tetramethyl-[1,1'-biphenyl]-2-yl)oxy](methyl)diphenylsilane [(*S*)-**3a'a**].

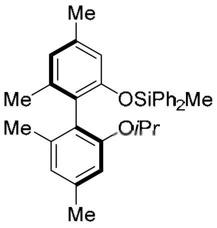

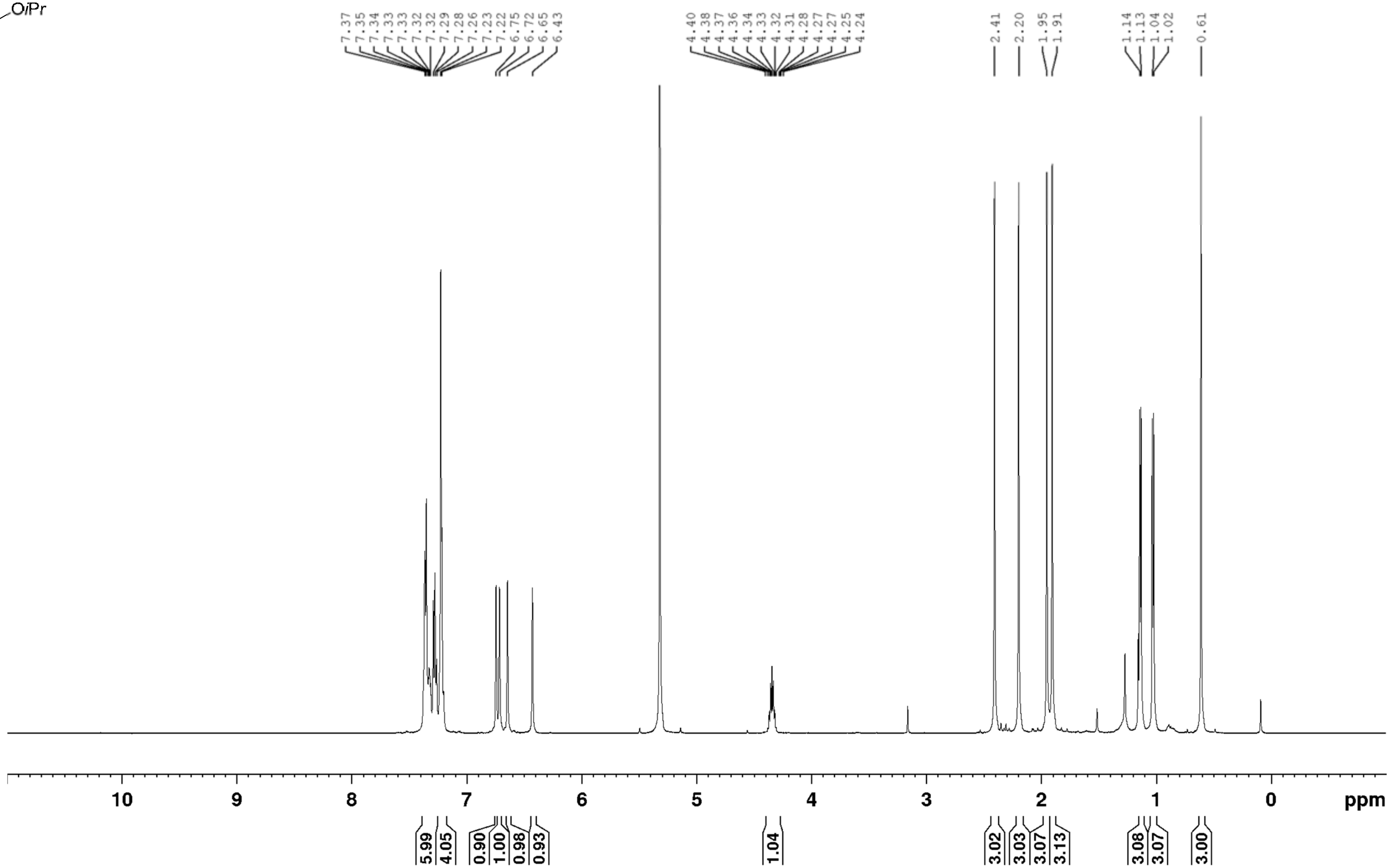

Figure S151. $^{13}C\{^{1}H\}$ NMR (126 MHz, CD_2Cl_2) of (*S*)-[(2'-Isopropoxy-4,4',6,6'-tetramethyl-[1,1'-biphenyl]-2-yl)oxy](methyl)diphenylsilane [(*S*)-**3a'a**].

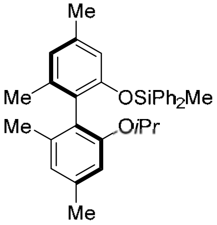

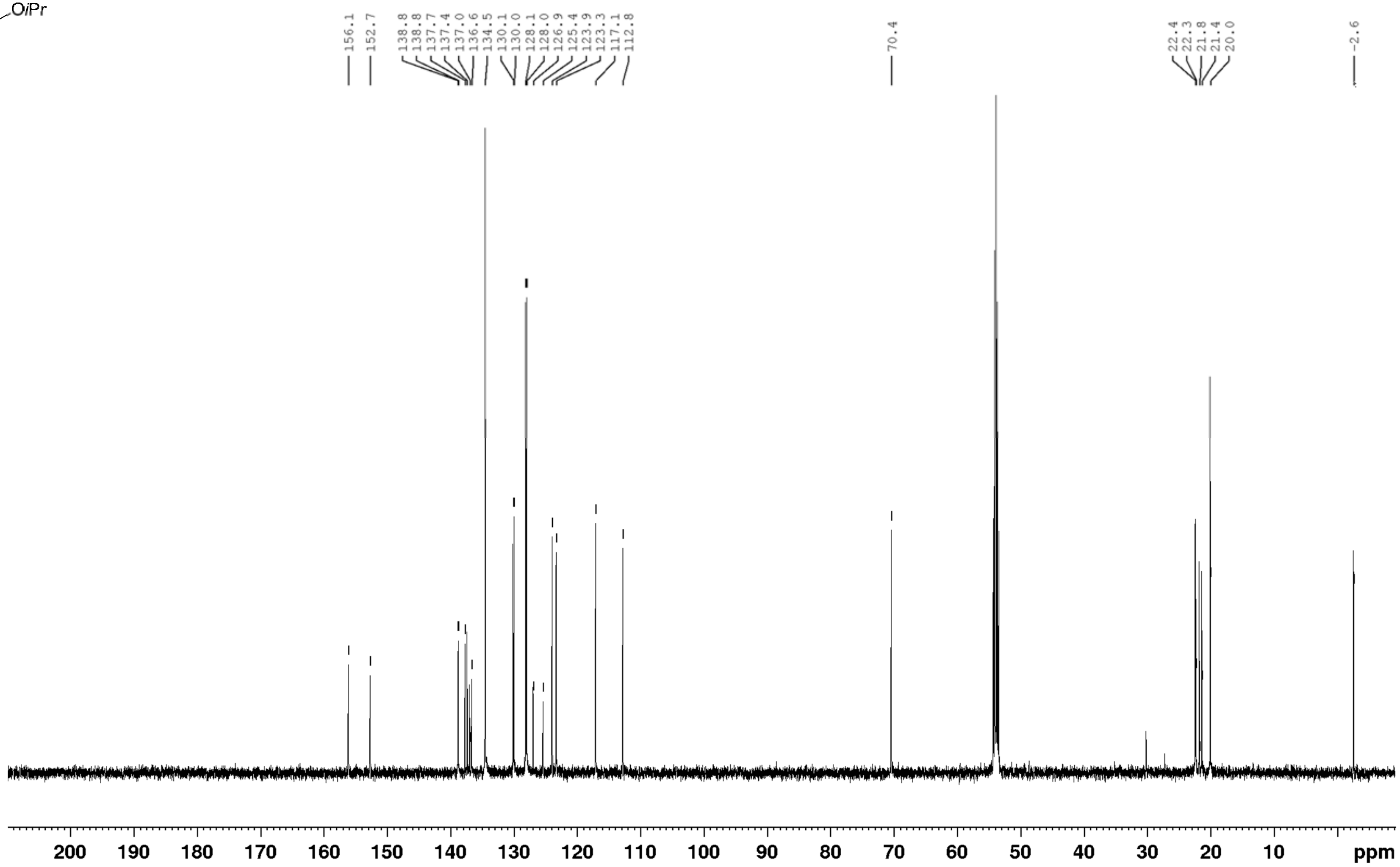

Figure S152. $^{1}H/^{29}Si$ HMQC NMR (500/99 MHz, CD_2Cl_2, optimized for J = 7 Hz) of (*S*)-[(2'-Isopropoxy-4,4',6,6'-tetramethyl-[1,1'-biphenyl]-2-yl)oxy](methyl)diphenylsilane [(*S*)-**3a'a**].

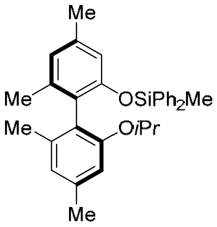

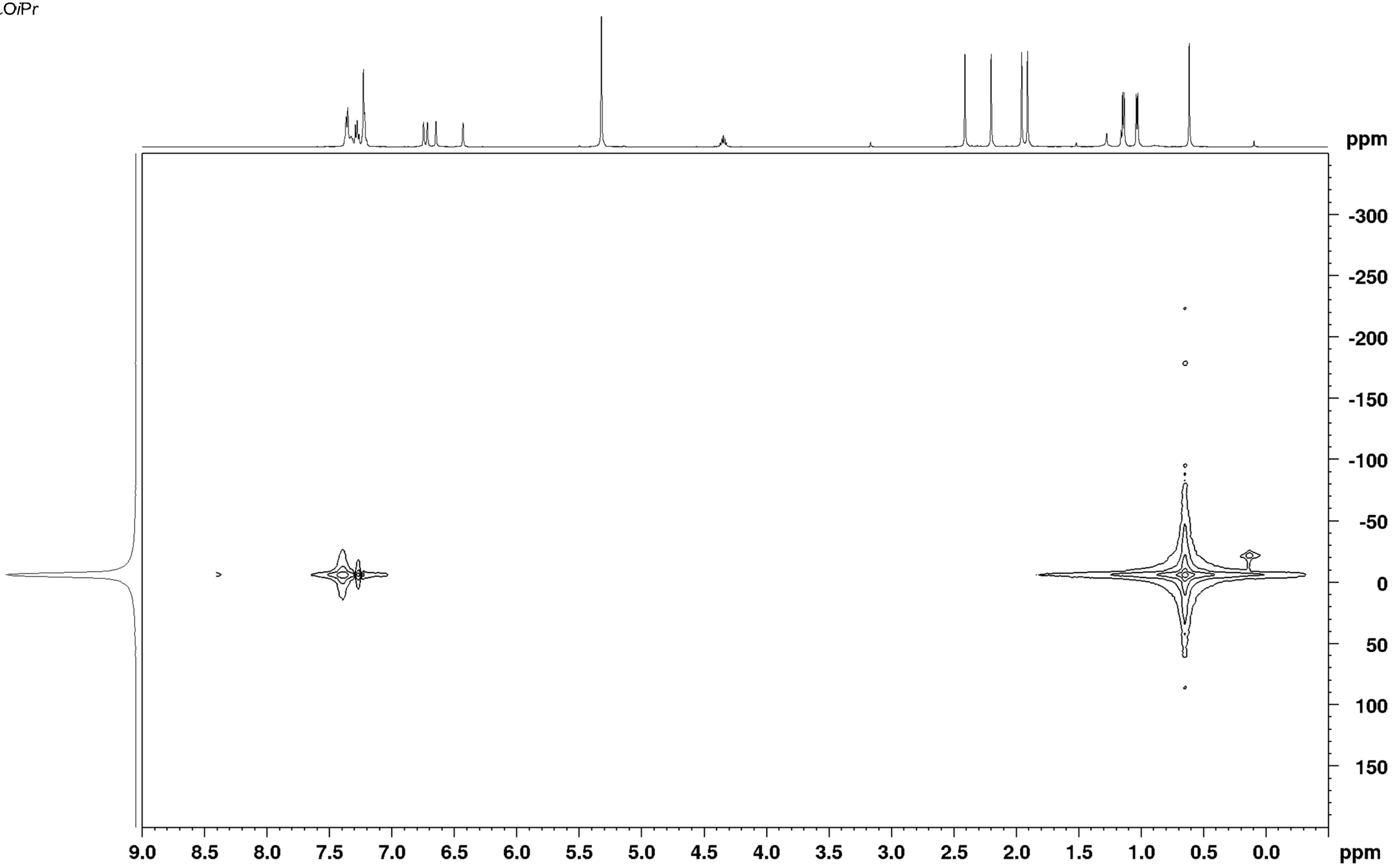

Figure S153. ^{1}H NMR (500 MHz, $CDCl_3$) of (*R*)-2'-Isopropoxy-4,4',6,6'-tetramethyl-[1,1'-biphenyl]-2-ol [(*R*)-**1a'**].

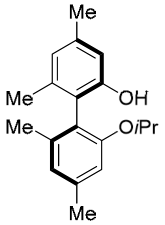

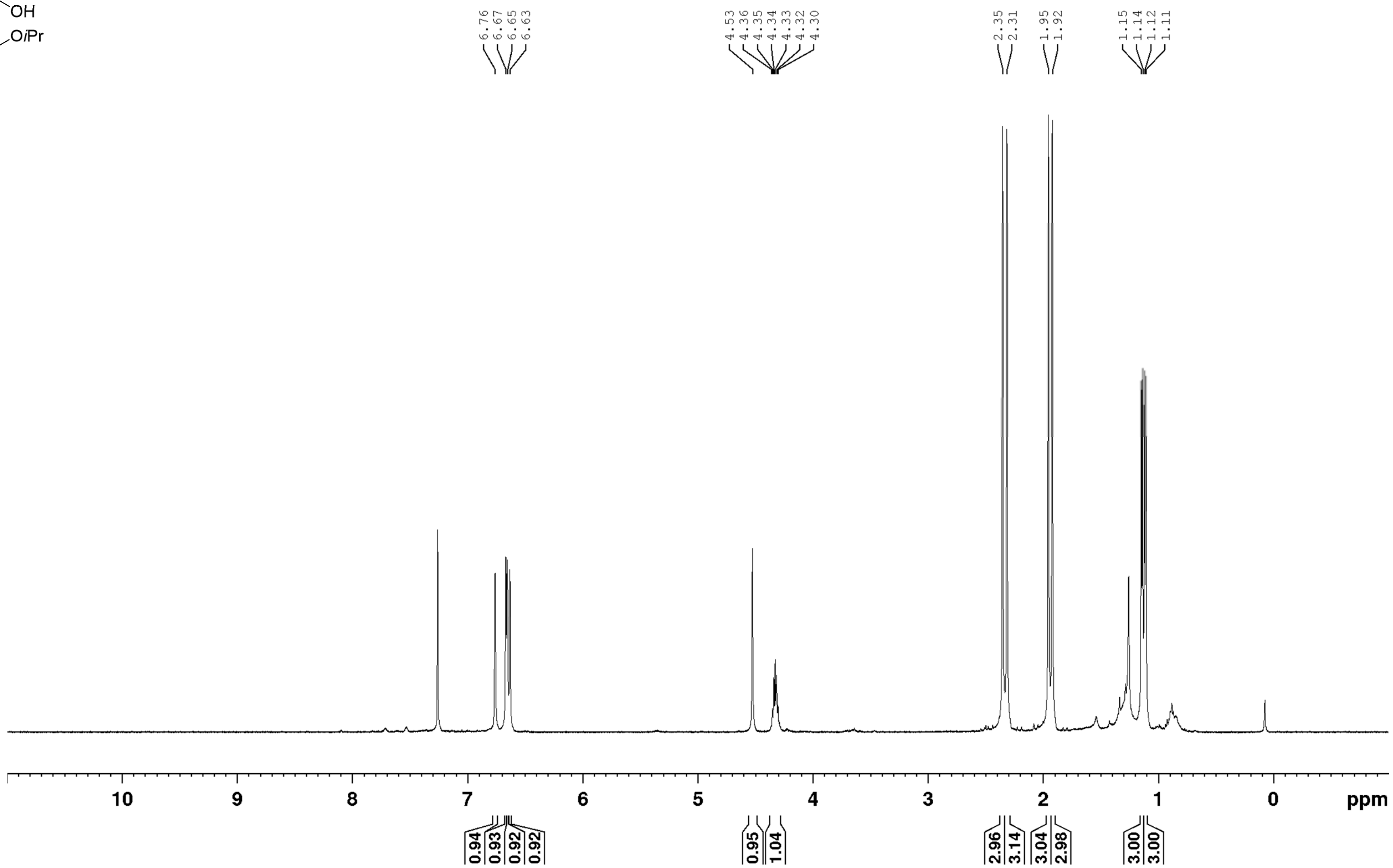

Figure S154. $^{13}C\{^{1}H\}$ NMR (126 MHz, $CDCl_3$) of (*R*)-2'-Isopropoxy-4,4',6,6'-tetramethyl-[1,1'-biphenyl]-2-ol [(*R*)-**1a'**].

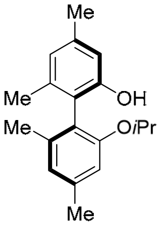

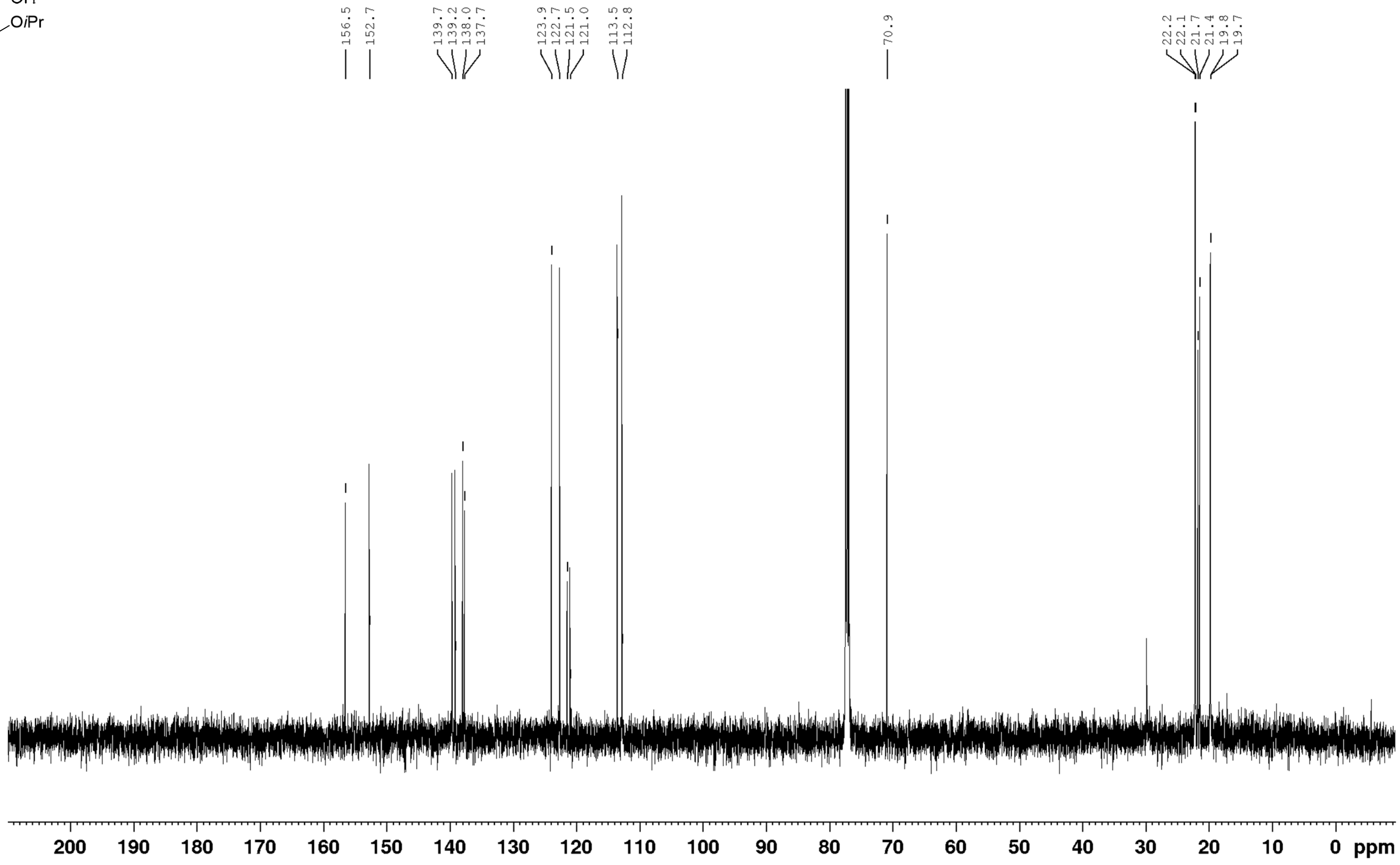

Figure S155. ^{1}H NMR (500 MHz, CD_2Cl_2) of (*S*)-[(14-Isopropoxy-5,6,7,8,9,10-hexahydrodibenzo[*a*,*c*][10]annulen-1-yl)oxy](methyl)diphenylsilane [(*S*)-**3b'a**].

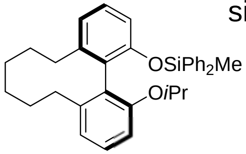

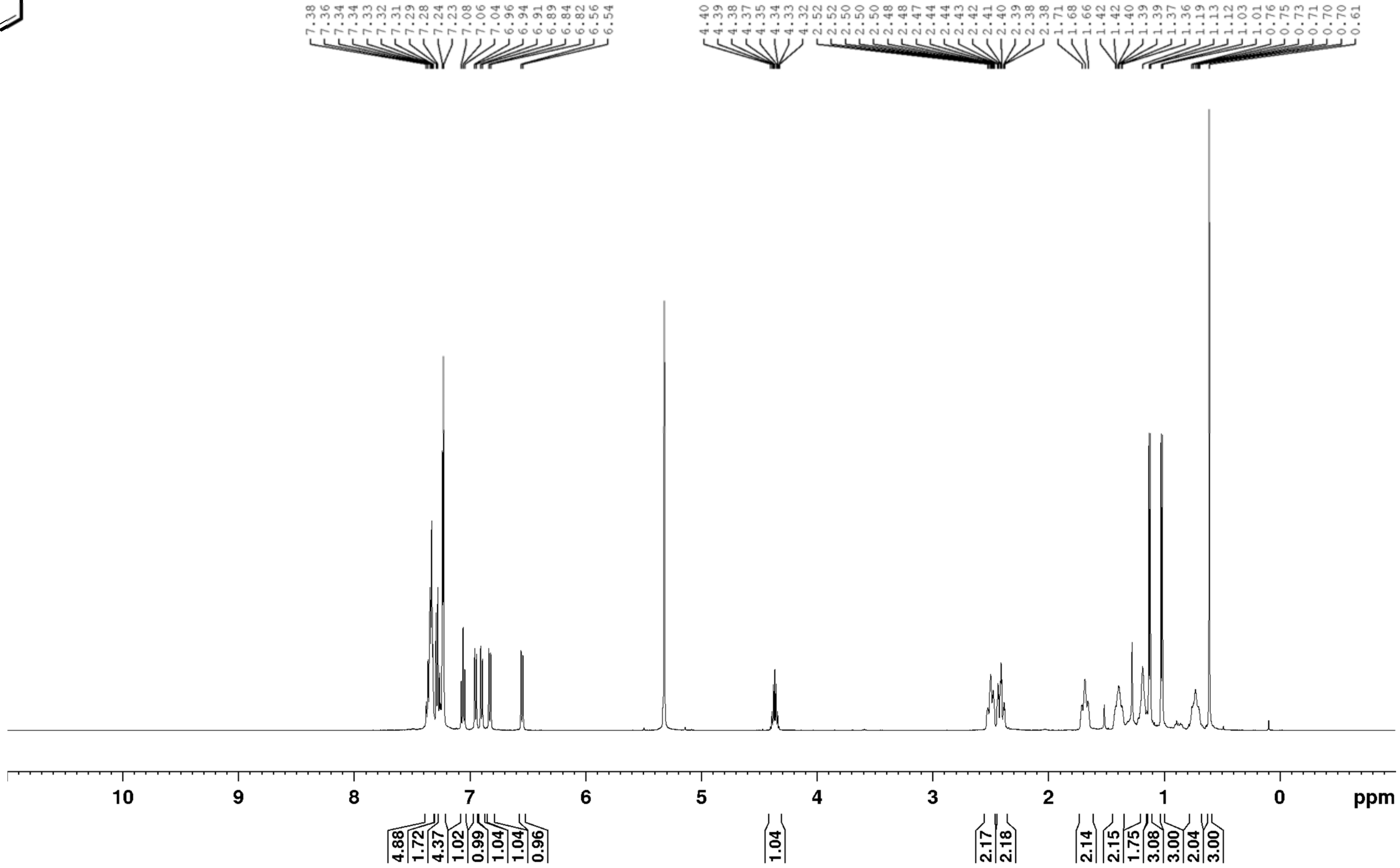

Figure S156. $^{13}C\{^{1}H\}$ NMR (126 MHz, CD_2Cl_2) of (*S*)-[(14-Isopropoxy-5,6,7,8,9,10-hexahydrodibenzo[*a*,*c*][10]annulen-1-yl)oxy](methyl)diphenylsilane [(*S*)-**3b'a**].

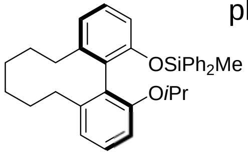

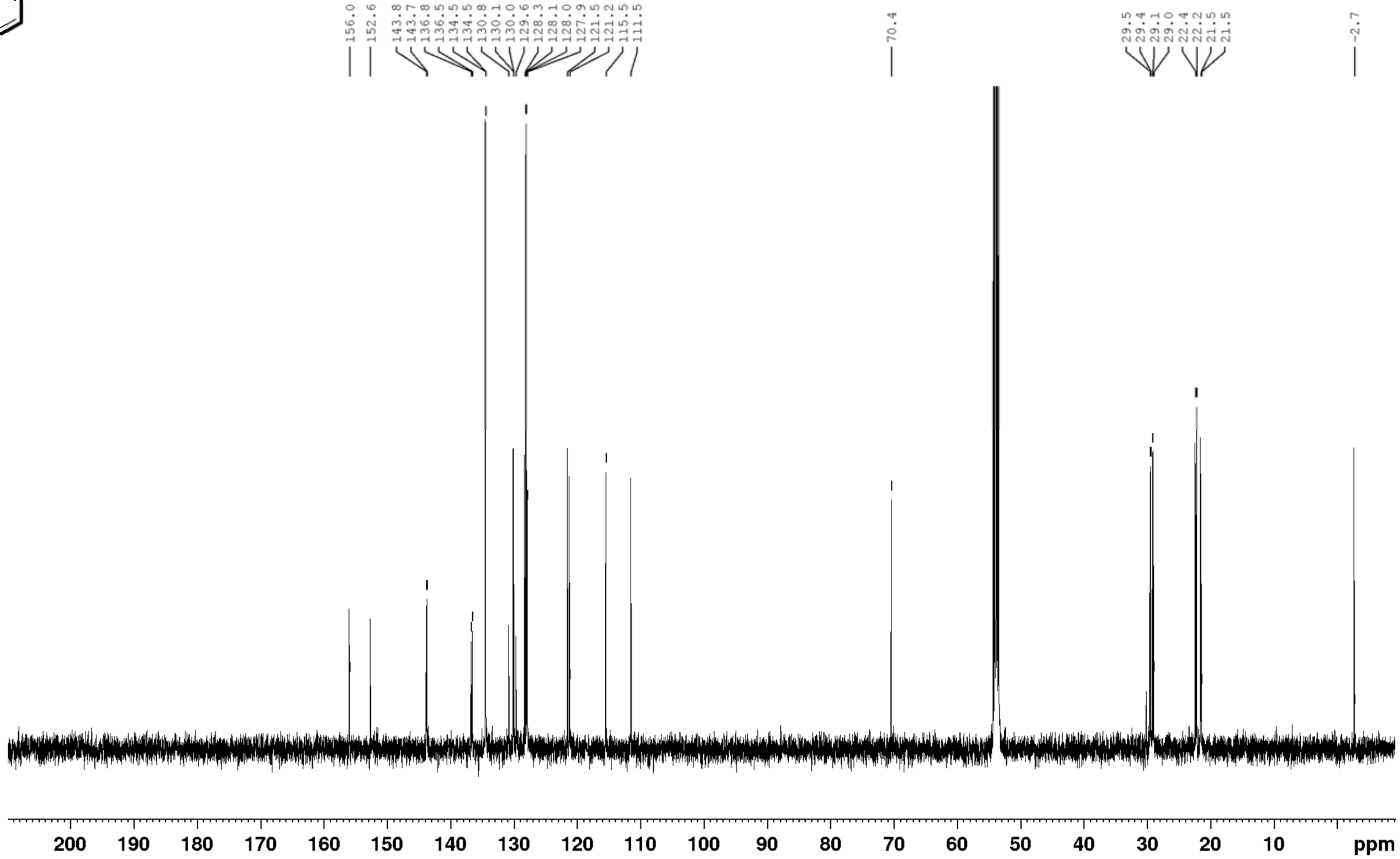

Figure S157. $^{1}H/^{29}Si$ HMQC NMR (500/99 MHz, CD_2Cl_2, optimized for J = 7 Hz) of (*S*)-[(14-Isopropoxy-5,6,7,8,9,10-hexahydrodibenzo-[*a*,*c*][10]annulen-1-yl)oxy](methyl)diphenylsilane [(*S*)-**3b’a**].

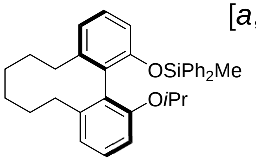

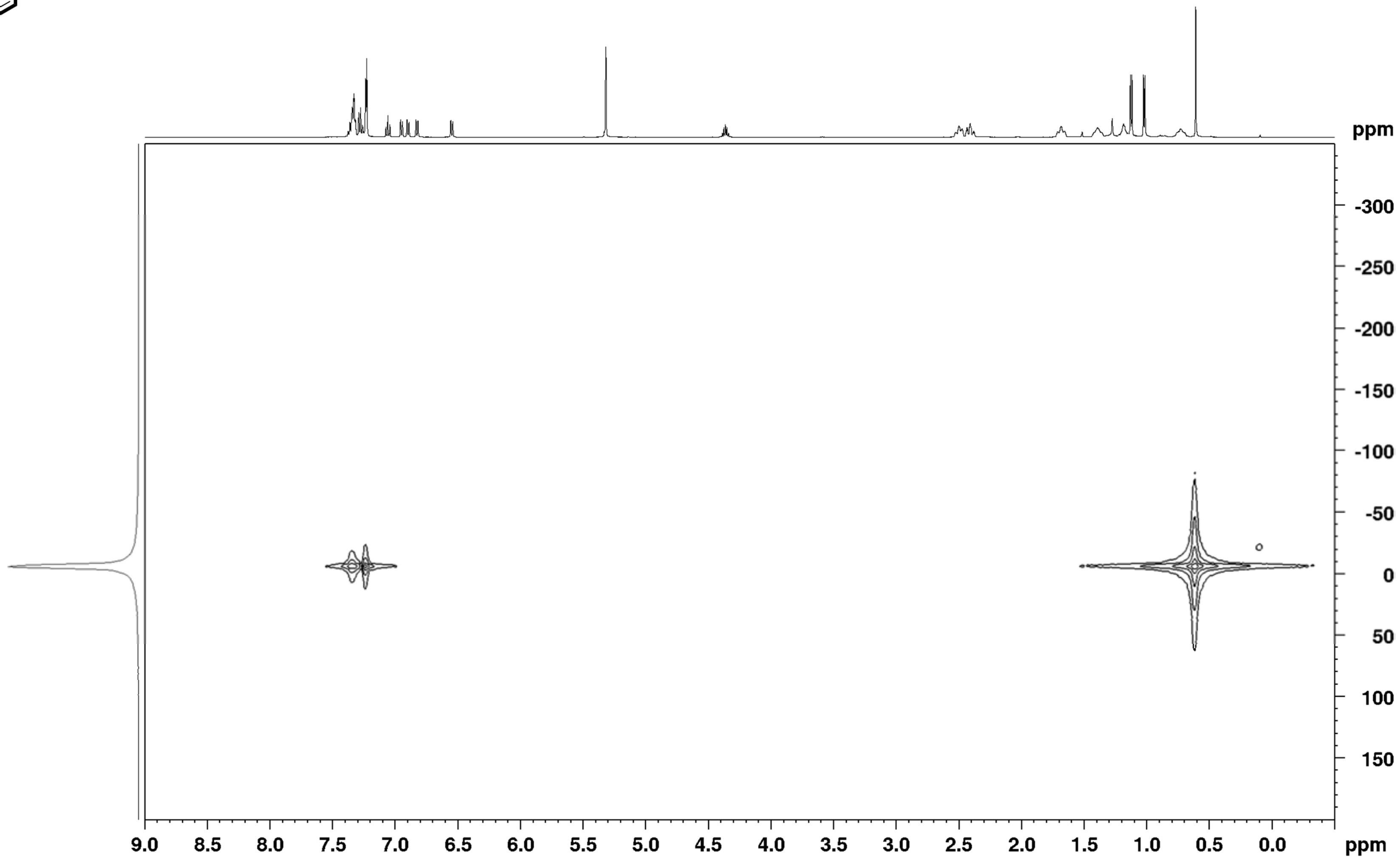

Figure S158. ^{1}H NMR (500 MHz, $CDCl_3$) of (*R*)-14-Isopropoxy-5,6,7,8,9,10-hexahydrodibenzo[*a*,*c*][10]annulen-1-ol [(*R*)-**1b'**].

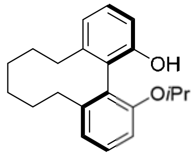

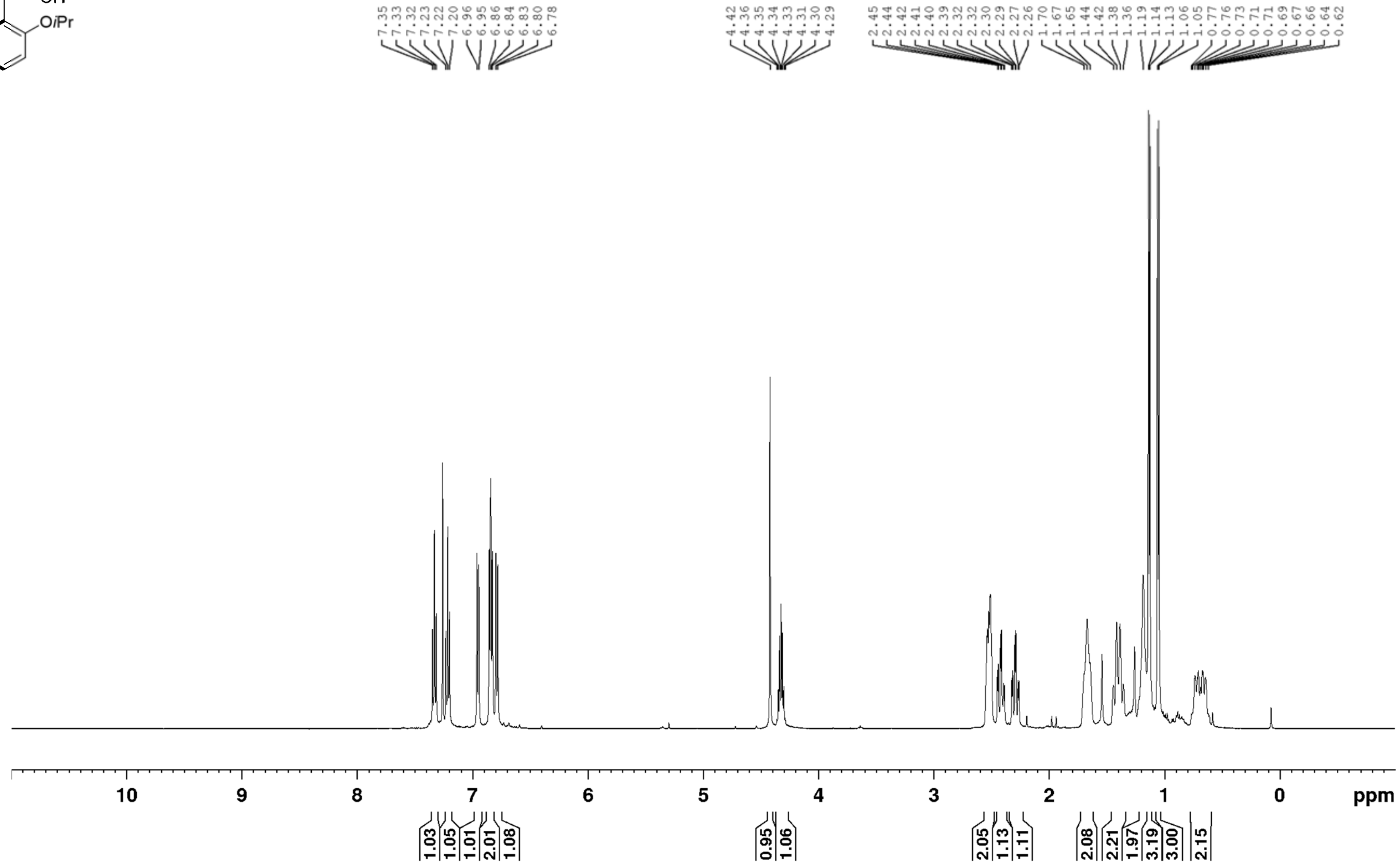

Figure S159. $^{13}C\{^{1}H\}$ NMR (126 MHz, $CDCl_3$) of (*R*)-14-Isopropoxy-5,6,7,8,9,10-hexahydrodibenzo[*a*,*c*][10]annulen-1-ol [(*R*)-**1b'**].

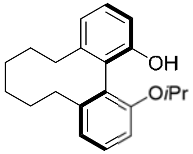

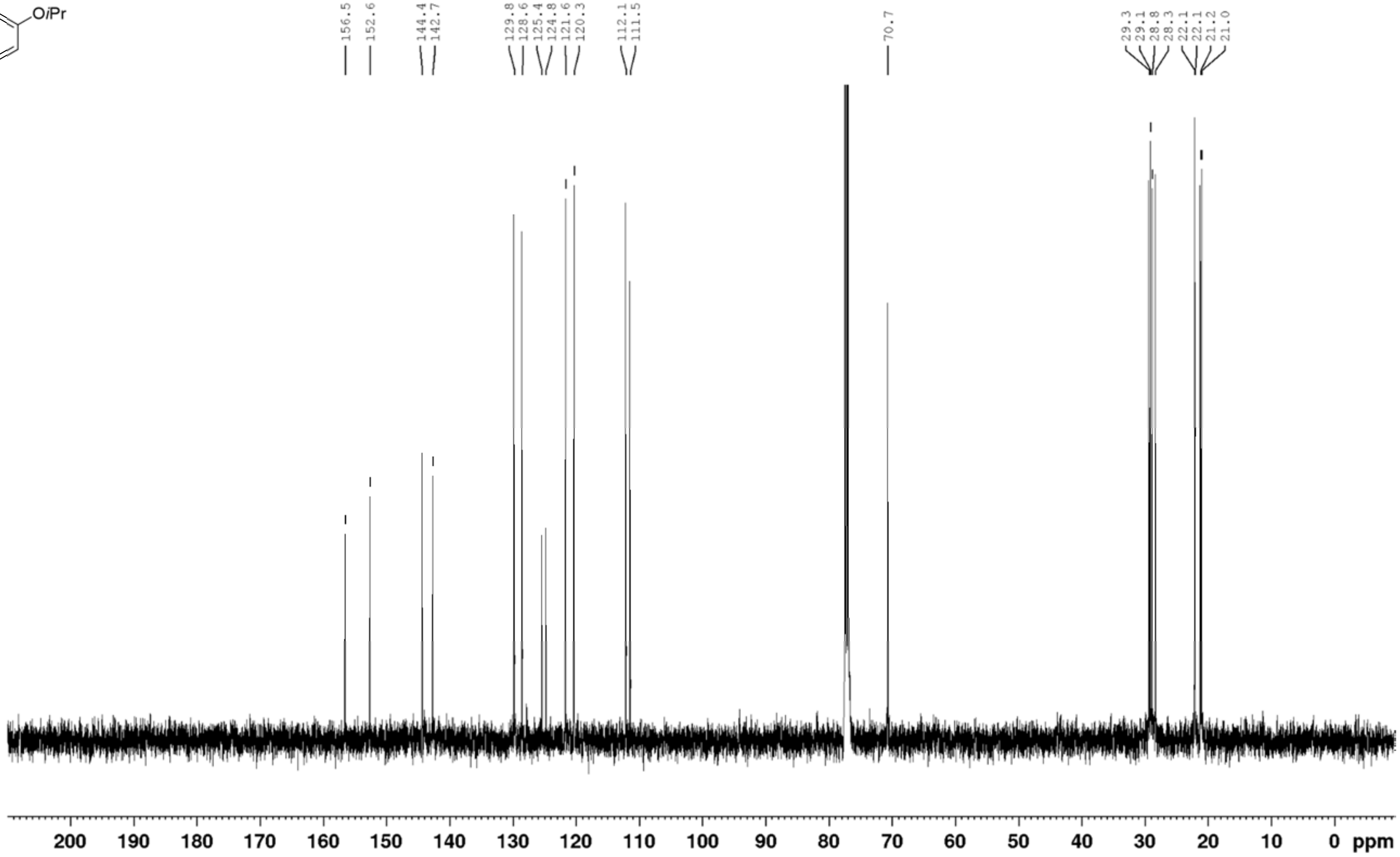

11 References

[1] Jones, B. A.; Balan, T.; Jolliffe, J. D., Campbell, C. D.; Smith, M. D. *Angew. Chem., Int. Ed.* **2019**, *58*, 4596–4600.

[2] Zhu, M.; Jiang, H.-J.; Sharanov, I.; Irran, E.; Oestreich, M. *Angew. Chem., Int. Ed.* **2023**, *62*, e202304475.

[3] Takayashi, M.; Ogasawara, K. *Tetrahedron: Asymmetry* **1997**, *8*, 3125–3130.

[4] Kahl, P.; Tkachenko, B. A.; Novikovsky, A. A.; Becker, J.; Dahl, J. E. P.; Carlson, R. M. K.; Fokin, A. A.; Schreiner, P. R. *Synthesis* **2014**, *46*, 787–798.

[5] Belokon, Y. N.; Bespalova, N. B.; Churkina, T. D.; Císařová, I.; Ezernitskaya, M. G.; Harutyunyan, S. R.; Hrdina, R.; Kagan, H. B.; Kočovský, P.; Kochetkov, K. A.; Larionov, O. V.; Lyssenko, K. A.; North, M.; Polášek, M.; Peregudov, A. S.; Prisyazhnyuk, V. V.; Vyskočil, Š. *J. Am. Chem. Soc.* **2003**, *125*, 12860–12871.

[6] Smrčina, M.; Vyskočil, Š.; Polívková, J.; Poláková, J.; Sejbal, J.; Hanus, V.; Polášek, M.; Verrier, H.; Kočovský, P. *Tetrahedron: Asymmetry* **1997**, *8*, 537–546.

[7] Ichinose, M.; Suematsu, H.; Yasutomi, Y.; Nishioka, Y.; Uchida, T.; Katsuki, T. *Angew. Chem., Int. Ed.* **2011**, *50*, 9884–9887.

[8] Handa, S.; Mathota Arachchige, Y. L. N.; Slaughter, L. M. *J. Org. Chem.* **2013**, *78*, 5694–5699.

[9] Endo, A.; Yanagisawa, A.; Abe, M.; Tohma, S.; Kan, T.; Fukuyama, T. *J. Am. Chem. Soc.* **2002**, *124*, 6552–6554.

[10] Ruzza, P.; Serra, P. A.; Fabbri, D.; Dettori, M. A.; Rocchitta, G.; Delogu, G. *Eur. J. Med. Chem.* **2017**, *126*, 1034–1038.

[11] Yoon, J.-M.; Lee, C.-Y.; Jo, Y.-I.; Cheon, C.-H. *J. Org. Chem.* **2016**, *81*, 8464–8469.

[12] Liu, T.; Liu, Y.; Xuan, W.; Cui, Y. *Angew. Chem., Int. Ed.* **2010**, *49*, 4121–4124.

[13] Heescher, C.; Schollmeyer, D.; Nubbemeyer, U. *Eur. J. Org. Chem.* **2013**, *20*, 4399–4404

[14] Wen, J.-F.; Hong, W.; Yuan, K.; Mak, T. C. W.; Wong, H. N. C. *J. Org. Chem.* **2003**, *68*, 8918–8931.

[15] Kang, H.; Herling, M. R.; Niederer, K. A.; Lee, Y. E.; Reddy, P. V. G.; Dey, S.; Allen, S. E.; Sung, P.; Hewitt, K.; Torruellas, C.; Kim, G. J.; Kozlowski, M. C. *J. Org. Chem.* **2018**, *83*, 14362–14383.

[16] Breit, B.; Reichert, S. *Org. Lett.* **2007**, *9*, 899–902.

[17] Zhang, P.; Yu, J.; Peng, F.; Wu, X.; Jie, J.; Liu, C.; Tian, H.; Yang, H.; Fu, H. *Chem. Eur. J.* **2016**, *22*, 17477–17484.

[18] Tuyet, T. M. T.; Harada, T.; Hashimoto, K.; Hatsuda, M.; Oku, A. *J. Org. Chem.* **2000**, *65*, 1335–1343.

[19] Lu, S.; Poh, S. B.; Zhao, Y. *Angew. Chem., Int. Ed.* **2014**, *53*, 11041–11045.
